# Systematic Review to Inform a World Health Organization (WHO) Clinical Practice Guideline: Benefits and Harms of Structured Exercise Programs for Chronic Primary Low Back Pain in Adults

**DOI:** 10.1007/s10926-023-10124-4

**Published:** 2023-11-22

**Authors:** Leslie Verville, Rachel Ogilvie, Cesar A. Hincapié, Danielle Southerst, Hainan Yu, André Bussières, Douglas P. Gross, Paulo Pereira, Silvano Mior, Andrea C. Tricco, Christine Cedraschi, Ginny Brunton, Margareta Nordin, Gaelan Connell, Jessica J. Wong, Heather M. Shearer, Joyce G. B. Lee, Dan Wang, Jill A. Hayden, Carol Cancelliere

**Affiliations:** 1 Institute for Disability and Rehabilitation Research and Faculty of Health Sciences, Ontario Tech University, Oshawa, Canada; 2 Department of Community Health and Epidemiology, Dalhousie University, Halifax, Canada; 3 EBPI-UWZH Musculoskeletal Epidemiology Research Group, University of Zurich and Balgrist University Hospital, Zurich, Switzerland; 4 Epidemiology, Biostatistics and Prevention Institute (EBPI), University of Zurich, Zurich, Switzerland; 5 University Spine Centre Zurich (UWZH), Balgrist University Hospital and University of Zurich, Zurich, Switzerland; 6 Département Chiropratique, Université du Québec à Trois-Rivières, Trois-Rivières (Québec), Canada; 7 School of Physical and Occupational Therapy, Faculty of Medicine and Health Sciences, McGill University, Montreal, Canada; 8 Department of Physical Therapy, University of Alberta, Edmonton, Canada; 9 Department of Neurosurgery, Centro Hospitalar Universitário São João, Faculty of Medicine, University of Porto, Porto, Portugal; 10 Department of Research and Innovation, Canadian Memorial Chiropractic College, Toronto, Canada; 11 Li Ka Shing Knowledge Institute, St. Michael’s Hospital, Unity Health Toronto, Toronto, Canada; 12 Epidemiology Division and Institute for Health Policy, Management, and Evaluation, Dalla Lana School of Public Health, University of Toronto, Toronto, Canada; 13 Queen’s Collaboration for Health Care Quality Joanna Briggs Institute Centre of Excellence, Queen’s University, Kingston, Canada; 14 Division of General Medical Rehabilitation, Geneva University and University Hospitals, Geneva, Switzerland; 15 Division of Clinical Pharmacology and Toxicology, Multidisciplinary Pain Centre, Geneva University Hospitals, Geneva, Switzerland; 16 EPPI-Centre, UCL Institute of Education, University College London, London, England, UK; 17 McMaster Midwifery Research Centre, McMaster University, Hamilton, Canada; 18 Department of Orthopedic Surgery and Environmental Medicine, NYU Grossman School of Medicine, New York University, New York, United States; 19 Bloorview Research Institute, Holland Bloorview Kids Rehabilitation Hospital, Toronto, Canada

**Keywords:** Low back pain, Systematic review, Meta-analysis, Exercise, Rehabilitation

## Abstract

**Purpose:**

Evaluate benefits and harms of structured exercise programs for chronic primary low back pain (CPLBP) in adults to inform a World Health Organization (WHO) standard clinical guideline.

**Methods:**

We searched for randomized controlled trials (RCTs) in electronic databases (inception to 17 May 2022). Eligible RCTs targeted structured exercise programs compared to placebo/sham, usual care, or no intervention (including comparison interventions where the attributable effect of exercise could be isolated). We extracted outcomes, appraised risk of bias, conducted meta-analyses where appropriate, and assessed certainty of evidence using GRADE.

**Results:**

We screened 2503 records (after initial screening through Cochrane RCT Classifier and Cochrane Crowd) and 398 full text RCTs. Thirteen RCTs rated with overall low or unclear risk of bias were synthesized. Assessing individual exercise types (predominantly very low certainty evidence), pain reduction was associated with aerobic exercise and Pilates vs. no intervention, and motor control exercise vs. sham. Improved function was associated with mixed exercise vs. usual care, and Pilates vs. no intervention. Temporary increased minor pain was associated with mixed exercise vs. no intervention, and yoga vs. usual care. Little to no difference was found for other comparisons and outcomes. When pooling exercise types, exercise vs. no intervention probably reduces pain in adults (8 RCTs, SMD = − 0.33, 95% CI − 0.58 to − 0.08) and functional limitations in adults and older adults (8 RCTs, SMD = − 0.31, 95% CI − 0.57 to − 0.05) (moderate certainty evidence).

**Conclusions:**

With moderate certainty, structured exercise programs probably reduce pain and functional limitations in adults and older people with CPLBP.

**Supplementary Information:**

The online version contains supplementary material available at 10.1007/s10926-023-10124-4.

## Introduction

Exercise therapy or structured exercise programs are widely used to manage low back pain (LBP). Exercise therapy is defined as “a series of specific movements with the aim of training or developing the body by a routine practice or as physical training to promote good physical health” [1] with a goal to reduce pain and functional limitations. Exercise therapies are prescribed or planned by health practitioners and include conducting postures, movements, and/or activities (e.g., strengthening, stretching, aerobic exercise) at varying dosages (duration, frequency, intensity) [2]. For people with chronic primary LBP (CPLBP), exercise therapy may improve musculoskeletal function, while also benefiting most other body systems and mental wellbeing [3]. In turn, this may reduce pain and functional limitations, and improve emotional and psychological wellbeing [2]. Exercise therapy is accessible globally.

Hayden and colleagues published a Cochrane review (2021) (literature search date ending 28 April 2018) to assess the impact of exercise therapy on pain and functional limitations for the management of chronic LBP in adults compared to placebo, no treatment, or usual care (pooled together), or other conservative treatments (249 randomized controlled trials (RCTs); 24,486 participants) [2] and a network meta-analysis comparing different types of exercise treatments [4]. They concluded with moderate certainty that exercise reduces pain and functional limitations when compared to no treatment, usual care, or sham, but not when compared to other conservative treatments [2].

To develop clinical practice guideline recommendations for the management of CPLBP in adults, the WHO commissioned the current systematic review to update the evidence and expand the aims of Hayden et al.’s previously published Cochrane review [2] by assessing additional important outcomes, conducting additional subgroup analyses, and disaggregating pairwise findings by exercise type (compared to no treatment, placebo/sham, or usual care).

The objectives of this systematic review of RCTs were to determine: (1) the benefits and harms of structured exercise programs compared to placebo/sham, usual care, or no intervention for the management of CPLBP in adults, including older adults (aged ≥ 60 years); and (2) whether the benefits and harms of structured exercise programs vary by age, gender/sex, presence of leg pain, race/ethnicity, or national economic development of the countries where the RCTs were conducted.

## Methods

This systematic review was conducted as part of a series of reviews to inform a WHO clinical practice guideline on the management of CPLBP in adults. The development of this guideline was ongoing at the time of submission of this manuscript. The review was conducted in collaboration with the Cochrane ‘exercise treatment for chronic low back pain’ collaborative review team, led by Prof. Jill Hayden [5]. The methods are detailed in the methodology article of this series [6].

Briefly, we updated and expanded the scope of the previously published Cochrane review [2]. The current review differs from Hayden et al.’s in the following ways: 1) we updated the literature search to include RCTs published from 28 April 2018 through 17 May 2022; 2) we assessed additional outcomes identified as critical by the WHO Guideline Development Group (GDG); 3) we conducted additional subgroup analyses (e.g., age, gender/sex); 4) we analyzed and reported the results separately for different exercise types, specifically comparing the effects of each exercise intervention to its respective comparator; 5) we did not assess ‘other conservative treatment’ comparisons (e.g., exercise vs. manual therapy); 6) we excluded RCTs of multimodal interventions where the specific effects of exercise could not be isolated; 7) we excluded RCTs judged to have high risk of bias in our primary analyses (although included all RCTs, irrespective of risk of bias in a supplementary analysis); and 8) the eligibility criteria for the population of interest differed to some degree. For example, we did not exclude RCTs of participants who had specific pathologies (e.g., disc herniation, lumbar spinal stenosis, and spondylolisthesis) provided all other eligibility criteria were satisfied. We also did not exclude RCTs of surgical populations if time since surgery was at least 12 months and participants had no history of fusion and/or disc replacement surgery.

We registered our review protocol with PROSPERO (International Prospective Register of Systematic Reviews) (CRD42022314576) on 7 March 2022.

In collaboration with the Cochrane review team, we modified the original search strategy using a detailed search optimization process [7]. The updated strategy was approved by a Cochrane musculoskeletal (MSK) literature search specialist. We searched MEDLINE (Ovid), CENTRAL (Cochrane Library, Wiley), and Embase (Elsevier) with no date or language restrictions up until 17 May 2022 (see Online Resource 1). Retrieved citations were de-duplicated against the search results of the previous Cochrane review update.

We included RCTs that compared structured exercise programs to placebo/sham, usual care, and no intervention (including comparison interventions where the attributable effect of exercise could be isolated, i.e., exercise + medication vs. same medication alone) in adults (aged ≥ 20 years) with CPLBP. Eligible interventions included all types of exercise with no exclusions based on setting, mode of delivery (e.g., in-person vs. telehealth, group vs. individual, home vs. clinic or community) or degree of personalization (standardized vs. individualized). Individuals may have been given verbal or written exercise instructions (e.g., handbook). Eligible exercise interventions, considered as separate exercise types, included, but were not limited to aerobic exercise; muscle strength training; stretching, flexibility or mobilizing exercises; yoga; core strengthening; motor control exercise; functional restoration exercise (not including multimodal programs of exercise with other interventions, such as psychological supports); Pilates; Tai Chi; Qigong; and mixed exercise therapies (i.e., two or more types of exercise in which one did not clearly predominate).

In addition to the main critical outcomes assessed for all reviews in this series (pain, function, health-related quality of life (HRQoL), harms, psychological functioning, and social participation including work), we also assessed additional critical outcomes requested by the WHO GDG for this review – the change in use of medications, burden related to the intervention or comparator (e.g., ease of access to the intervention, time burden of the intervention), performance-based physical functioning, and falls (older adults only aged ≥ 60 years). We reported outcomes based on post-intervention follow-up intervals including: (1) immediate term (closest to 2 weeks after the intervention period); (2) short term (closest to 3 months after the intervention period); (3) intermediate term (closest to 6 months after the intervention period); (4) long term (closest to 12 months after the intervention period); and (5) extra-long term (more than 12 months after the intervention period).

We assessed between-group differences to determine the magnitude of the effect of an intervention and to assess its effectiveness [8, 9] (details in the methodology article in this series) [6]. Briefly, we considered a mean difference (MD) of ≥ 10% of the scale range or ≥ 10% difference in risk for dichotomous outcomes to be a minimally important difference (MID) [10, 11]. If the standardized mean difference (SMD) was calculated, SMD ≥ 0.2 was considered a MID [12].

Pairs of reviewers independently screened studies for eligibility, and critically appraised risk of bias (ROB) using the Cochrane ROB 1 tool [13], modified from the Cochrane Back and Neck Methods Guidelines [14]. One reviewer extracted data for all included RCTs, which was then verified by a second reviewer. Any disagreements were resolved by consensus between paired reviewers or with a third reviewer, when necessary. Forms and guidance for screening, risk of bias assessment, and data extraction were adapted from those developed by Hayden et al. in the conduct of the ‘exercise for chronic low back pain’ collaborative review, in which members of our team participated [5]. The forms were completed using DistillerSR Inc. [15]—a web-based electronic systematic review software application.

In our primary synthesis, our analyses were conducted according to exercise type (e.g., aerobic exercise, yoga). In addition to the subgroup analyses conducted for all reviews in this series (age, gender/sex, presence of leg pain, race/ethnicity, and national economic development of country where RCT was conducted), we aimed to perform subgroup analyses according to exercise dosage and intensity, and to conduct a sensitivity analysis by removing RCTs rated as unclear ROB.

We conducted random-effects meta-analyses and narrative synthesis where meta-analysis was not appropriate [16], and graded the certainty of evidence using Grading of Recommendations Assessment, Development and Evaluation (GRADE) [17]. The comparisons involving no intervention and interventions where the attributable effect of exercise could be isolated were combined in meta-analyses. Meta-analyses were conducted using R statistical packages [18, 19], and GRADE Evidence Profiles and GRADE Summary of Findings tables were developed using GRADEpro software [20].

Following completion of our primary synthesis, the WHO commissioned a supplementary evidence synthesis to further inform the formulation of recommendations by the GDG. In the supplementary evidence synthesis, we synthesized the 13 RCTs (judged as low or unclear ROB) included in our primary evidence synthesis along with 55 additional RCTs originally excluded from our synthesis due to high ROB. These studies were identified as having been published in the period 28 April 2018 (search end date of Hayden’s previously published Cochrane review [2]) to 17 May 2022. We included all 13 trials from the primary synthesis (from database inception through 17 May 2022) in this supplementary synthesis since no differences in the magnitude or directions of the effect estimates were observed in a sensitivity analysis where RCTs published on or before 28 April 2018 were excluded.

In the supplementary evidence synthesis (see Online Resource 8), we included RCTs that compared *any structured exercise program or exercise type* to the same comparisons as in our primary synthesis. The outcomes assessed were pain, function, and harms only. The key differences between the primary and supplementary evidence syntheses are summarized (Table 1).

**Table 1:** Differences between the primary and supplementary evidence syntheses

Evidence synthesis component	Primary evidence synthesis	Supplementary evidence synthesis
Search period	Database inception through 17 May 2022 (for low or unclear ROB RCTs)	Database inception through 17 May 2022 (for low or unclear ROB RCTs) + 28 April 2018 through 17 May 2022 for high ROB RCTs^a^
Inclusion criteria based on ROB	Low or unclear ROB	Low, unclear or high ROB
Outcomes	Pain, function, harms, health-related quality of life, psychological functioning, social participation (+ change in use of medications, burden related to treatment, performance-based physical functioning, and falls in adults aged ≥ 60 years)	Pain, function, harms
Sub-group comparisons	Age, national economic development, exercise type, ROB judgement (low vs. unclear), gender/sex, presence of leg pain, race/ethnicity	Age, national economic development, exercise type, ROB judgement (low vs. not low)^b^

ROB: risk of bias

^a^Randomized controlled trials (RCTs) published on or before 28 April 2018 were included in the supplementary synthesis since no differences in the magnitude or direction of effect estimates were observed in sensitivity analyses when these RCTs were excluded

^b^Other subgroups were not analyzed (i.e., gender/sex, presence of leg pain, race/ethnicity) as the primary synthesis did not demonstrate varied findings

The WHO was provided with the primary and supplementary evidence syntheses to support the GDG in formulation of recommendations. The GDG may have also considered other aligned evidence when formulating its recommendations (currently under development).

## Results

**Fig. 1:**
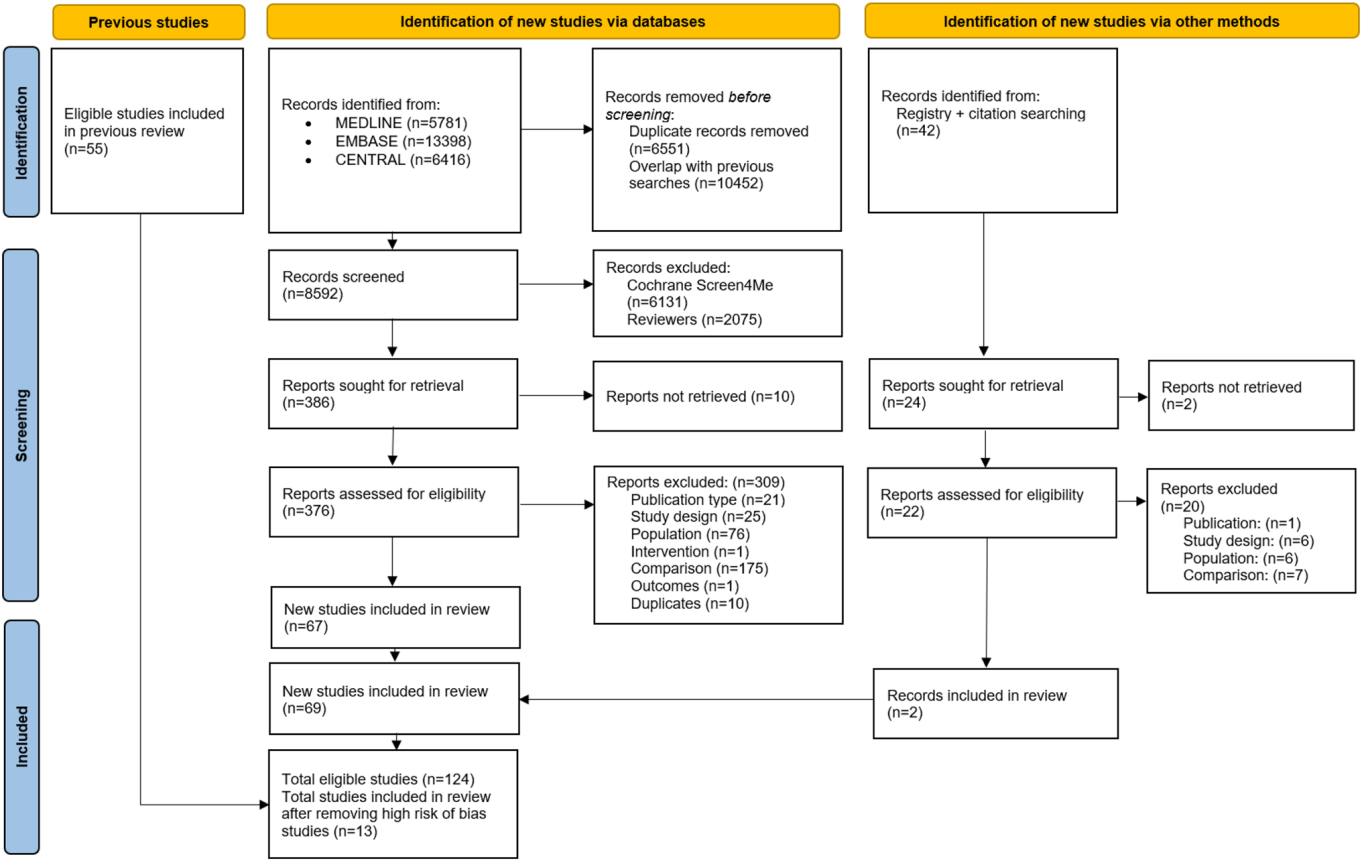
Flow diagram of literature search

Our electronic search strategy identified 8592 new citations (Fig. 1), the Cochrane RCT Classifier/known assessments and Cochrane Crowd first excluded 6131 non-RCTs (RCT Classifier/known assessments: 3281, Cochrane Crowd: 2850). We subsequently screened 2503 records and 398 full-text reports. Of these, 69 new RCTs were eligible. We included an additional 55 RCTs from the published review [2], which totalled 124 RCTs. Of these, 111 were excluded from the primary analyses due to an overall high ROB rating (see Online Resource 2). Therefore, we included 13 RCTs (*n* = 1362 participants) in our synthesis [21–33] ranging from 45 to 313 participants per trial, predominantly from healthcare settings (see Online Resources 3, 4).

Regarding unpublished RCTs, we identified 185 RCTs (registrations and published protocols) in the WHO ICTRP. Of these, 14 authors could not be contacted because an email address could not be located. Thus, 171 authors were contacted and 164 received our invitation to respond to a REDCap survey [34, 35] consisting of our specific queries. Of these, 32% (53/164) responded; 19 reported that their RCT would not meet our inclusion criteria; 26 reported their RCT was ongoing; and 8 provided citations, which we confirmed were already included in our review. Thus, we did not include any unpublished RCTs in our review.

The 13 included RCTs were conducted in high-income economies [36]: Australia (1 RCT) [22], Germany (1 RCT) [28], Japan (1 RCT) [24], Netherlands (1 RCT) [30], United Kingdom (1 RCT) [32], and the United States (1 RCT) [33]; upper-middle income economies: Brazil (3 RCTs) [23, 25, 26] and Turkey (1 RCT) [31]; and lower-middle income economies: India (1 RCT) [21] and Iran (2 RCTs) [27, 29]. The mean age ranged from 37 to 74 years; two RCTs assessed older adults (*n* = 252) [24, 33]. The percentage of females within the RCTs ranged from 27 to 84%. None of the RCTs included adults who all had leg pain in addition to back pain. Two RCTs reported on adults without leg pain [23, 29]. In one RCT, adults had CPLBP either with or without non-radicular leg pain [30]; and in another adults had CPLBP either with or without unclassified (radicular vs. non-radicular) leg pain [33]. The presence of leg pain was not classified in nine RCTs [21–28, 31, 32]. Where reported by authors, the mean duration of CPLBP ranged from 7 months to ≥ 15 years.

The RCTs assessed aerobic exercise [26, 28]; core strengthening exercise [27]; muscle strength training [23, 29]; mixed exercise [21, 24, 30, 31, 33]; Pilates [25]; stretching, flexibility or mobilizing exercises [29]; yoga [32]; and motor control exercise [22]. We did not identify any low or unclear ROB trials that assessed the other exercise types: functional restoration, Tai Chi, Qigong, or hydrotherapy/aquatic exercise. The number of exercise sessions delivered ranged from 8 to 30, with the duration of each session ranging from 15 to 105 min. Exercise was compared to interventions where the attributable effects of exercise could be isolated, sham, or usual care, and most RCTs assessed pain and function in the immediate term (Table 2). The outcomes were assessed in the immediate term (13 RCTs) [21–33], short term (3 RCTs) [24, 28, 31], intermediate term (8 RCTs) [22–25, 30–33], and long term (4 RCTs) [22, 23, 30, 32] (Table 2). The RCTs were rated as overall unclear ROB (11, 85%), or low ROB (2, 15%) (Online Resource 2). The agreement on ROB judgements was high (weighted overall kappa score 0.92).

**Table 2:** Number of included RCTs by comparison and outcome

Outcome assessed (post-intervention)	Follow-up			
	Immediate (2 weeks)	Short (3 months)	Intermediate (6 months)	Long (12 months)
*Exercise versus no intervention*^*a*^* (9 RCTs*^*b*^*, 10 exercise groups)*				
Pain	**10**^**c**^	*2*	**5**^**c**^	*3*
Function	**10**^**c**^	*2*	**5**^**c**^	*2*
Health-related quality of life	**3**^**c**^	*2*	*2*^*c*^	*1*
Psychological functioning	**3**^**c**^	–	**3**^**c**^	*1*
Social participation	–	–	–	–
Change in medication use	–	–	–	–
Treatment-related burden	–	–	–	–
Performance-based physical functioning	*1*^*c*^	–	*1*^*c*^	–
Falls	–	–	–	–
Harms	**5**^**c**^			
Number of RCTs per exercise type: aerobic = 2, core strengthening = 1, general strength training = 2, mixed exercise = 3^c^, Pilates = 1, stretching/flexibility/mobilizing exercise = 1				
*Exercise versus sham*				
Pain	*1*	–	*1*	*1*
Function	*1*	–	*1*	*1*
Health-related quality of life	–	–	–	–
Psychological functioning	–	–	–	–
Social participation	–	–	–	–
Change in medication use	–	–	–	–
Treatment-related burden	–	–	–	–
Performance-based physical functioning	–	–	–	–
Falls	–	–	–	–
Harms	1			
Number of RCTs per exercise type: motor control = 1				
*Exercise versus usual care*				
Pain	**3**^**c**^	*1*^*c*^	*2*^*c*^	*1*
Function	**3**^**c**^	*1*^*c*^	*2*^*c*^	*1*
Health-related quality of life	*2*^*c*^	*1*^*c*^	*2*^*c*^	*1*
Psychological functioning	*2*^*c*^	*1*^*c*^	*2*^*c*^	*1*
Social participation	–	–	–	–
Change in medication use	–	–	–	–
Treatment-related burden	–	–	–	–
Performance-based physical functioning	–	–	–	–
Falls	–	–	–	–
Harms	1			
Number of RCTs per exercise type: mixed exercise = 2^c^, yoga = 1				

Bold values: majority of studies are in this category, italic values: some studies

^a^Included comparison interventions where the attributable effect of exercise could be isolated (i.e., combined exercise with treatment B versus treatment B alone)

^b^One RCT reported two intervention groups: 1) hamstring static stretching + physiotherapy vs. physiotherapy, 2) hamstring strengthening in lengthened position + physiotherapy vs. physiotherapy

^c^One RCT included adults aged ≥ 60 years

### Certainty of Evidence

The certainty of the evidence ranged from very low (for outcomes assessed with the individual exercise types) to moderate (for outcomes assessed after pooling exercise types). Certainty of evidence was downgraded due to ROB, inconsistency, indirectness, and/or imprecision of the effect estimates (see Online Resources 5, 6 and 7). For results reported as a MD, lower or negative values refer to reduced pain, functional limitations, depression, or fear avoidance; higher or positive values refer to improved HRQoL and self-efficacy.

### Aerobic Exercise Versus Comparison Interventions With Isolated Exercise Effects

The certainty of evidence was very low for all outcomes. It is uncertain whether aerobic exercise reduces ***pain**** (scale 0 to 10, 0* = *no pain)* in the immediate (2 RCTs; MD = − 1.33, 95% confidence interval (CI) − 2.27 to − 0.40) (plot 1.1.1.1) [26, 28], or short term (1 RCT; MD = − 1.26, 95% CI − 2.51 to − 0.01) (plot 1.1.1.2) [28]. It is uncertain whether aerobic exercise makes little or no difference to ***functional limitations**** (scale 0 to 100, 0* = *no functional limitations)* in the immediate (2 RCTs; MD = − 1.30, 95% CI − 3.89 to 1.29) (plot 1.1.2.1), [26, 28] or short term (1 RCT; MD = 0.90, 95% CI − 5.66 to 7.46) (plot 1.1.2.2) [28]. It is uncertain whether aerobic exercise makes little or no difference to ***HRQoL**** (scale 0 to 100, 0* = *poor quality of life; PCS* = *physical component summary; MCS* = *mental component summary)* in the immediate and short terms (immediate: ***PCS:*** MD = 3.50, 95% CI − 0.05 to 7.05; ***MCS:*** MD = − 1.20, 95% CI − 5.22 to 2.82; plot 1.1.3.1.1 and 1.1.3.1.2; short term: ***PCS:*** MD = 3.70, 95% CI 0.05 to 7.35; ***MCS:*** MD = 2.20, 95% CI − 3.15 to 7.55; plot 1.1.3.2.1 and 1.1.3.2.2) [28]. It is uncertain whether aerobic exercise makes little or no difference to ***adverse events/harms*** (1 RCT) (no plot, narrative synthesis). Authors reported no adverse events [28].

### Core Strengthening Exercise Versus Comparison Interventions With Isolated Exercise Effects

The certainty of evidence was very low for all outcomes and based on one RCT [27]. In the immediate term, it is uncertain whether core strengthening reduces ***pain**** (scale 0 to 10, 0* = *no pain)* (MD = − 0.56, 95% CI − 0.94 to − 0.19) (plot 2.1.1.1), or ***functional limitations**** (scale 0 to 24, 0* = *no functional limitations)* (MD = − 1.7, 95% CI − 2.42 to − 0.98) (plot 2.1.2.1). It is uncertain whether core strengthening exercise makes little or no difference to ***adverse events/harms*** (no plot, narrative synthesis).

### Muscle Strength Training Versus Comparison Interventions With Isolated Exercise Effects

The certainty of evidence was very low for all outcomes. It is uncertain whether muscle strength training makes little or no difference to ***pain**** (scale 0 to 10, 0* = *no pain)* in the immediate (2 RCTs; MD = − 0.39, 95% CI − 1.16 to 0.38) (plot 3.1.1.1) [23, 29], intermediate (1 RCT; MD = − 0.40, 95% CI − 1.67 to 0.87) (plot, 3.1.1.2) [23], or long term (1 RCT; MD = − 0.10, 95% CI − 1.32 to 1.12) (plot, 3.1.1.3) [23]. It is uncertain whether muscle strength training makes little or no difference to ***function**** (benefit indicated by lower values)* in the immediate (2 RCTs; standardized mean difference (SMD) = 0.05, 95% CI − 0.34 to 0.45) (plot 3.1.2.1) [23, 29]; or intermediate (1 RCT; MD = − 0.60, 95% CI − 3.20 to 2.00) (plot 3.1.2.2) [23], and long terms (1 RCT; MD = − 0.20, 95% CI − 2.73 to 2.33) (plot 3.1.2.3) (*scale 0 to 24, 0* = *no functional limitations*) [23].

### Mixed Exercise Versus Comparison Interventions With Isolated Exercise Effects

#### All Adults

Due to very low certainty evidence, it is uncertain whether mixed exercise makes little or no difference to ***pain*** in the immediate (2 RCTs; SMD = − 0.01, 95% CI − 0.32 to 0.31; *benefit indicated by lower values*) (plot 4.1.1.1) [31, 33], short (1 RCT; MD = − 0.10, 95% CI − 1.34 to 1.14; *scale 0 to 10, 0* = *no pain*) (plot 4.1.1.2) [31], intermediate (2 RCTs; SMD = 0.03, 95% CI − 0.23 to 0.29; *benefit indicated by lower values*) (plot 4.1.1.3) [31, 33], or long term (1 RCT; MD = 8.88, 95% CI − 0.36 to 18.13; *scale 0 to 100, 0* = *no pain*) (no plot, narrative synthesis) [30]. Mixed exercise may make little or no difference to ***function*** in the immediate term (2 RCTs; SMD = − 0.15, 95% CI − 0.48 to 0.18; *benefit indicated by lower values*; low certainty evidence) (plot 4.1.2.1) [31, 33]. Due to very low certainty evidence, it is uncertain whether mixed exercise makes little or no difference to function in the short (1 trial; MD = − 1.25, 95% CI − 2.79 to 0.29; *scale 0 to 9, 0* = *no functional limitations*) (plot 4.1.2.1) [31], intermediate (2 trials; SMD = − 0.09, 95% CI − 0.42 to 0.24; *benefit indicated by lower values*) (plot 4.1.2.2) [31, 33], or long term (1 trial; MD = 1.62, 95% CI − 0.06 to 3.31; *scale 0 to 24, 0* = *no functional limitations*) (no plot, narrative synthesis) [30]. Due to very low certainty evidence from one RCT [31], it is uncertain whether mixed exercise makes little or no difference to ***HRQoL**** (scale 0 to 3, 0* = *poor quality of life)* in the immediate (MD = 0.24, 95% CI − 0.06 to 0.54) (plot 4.1.3.1), short (MD = 0.17, 95% CI − 0.07 to 0.41) (plot 4.1.3.2), or intermediate term (MD = 0.19, 95% CI − 0.09 to 0.47) (plot 4.1.3.3); or ***depression**** (scale 0 to 63, 0* = *no depression)* in the long term (MD = − 0.09, 95% CI − 2.11 to 1.93) (no plot, narrative synthesis). It is uncertain whether mixed exercise makes little or no difference to ***adverse events/harms*** (2 RCTs; odds ratio (OR) 4.24, 95% CI 0.69 to 25.95; very low certainty evidence) (plot 4.1.5) [30, 33]. Adverse events were mainly minor and included back and knee pain.

#### Older Adults

Due to very low certainty evidence from 1 RCT [33], in older adults, it is uncertain if mixed exercise makes little or no difference to ***pain**** (benefit indicated by lower values)* in the immediate (SMD = − 0.10, 95% CI − 0.44 to 0.23) (plot 4.1.6.1.1), or intermediate term (SMD = − 0.01, 95% CI − 0.39 to 0.40) (plot 4.1.6.1.2); ***function**** (benefit indicated by lower values)* in the immediate (SMD = − 0.01, 95% CI − 0.29 to 0.27) (plot 4.1.6.2.1), or intermediate term (SMD = 0.03, 95% CI − 0.24 to 0.31) (plot 4.1.6.2.2); ***depression**** (scale 0 to 30, 0* = *no depression)* in the immediate (MD = − 0.11, 95% CI − 1.87 to 1.66) (plot 4.1.4.1) or intermediate term (MD = 0.14, 95% CI − 1.92 to 2.20) (plot 4.1.4.2); ***self-efficacy**** (scale 10–100, benefit indicated by higher values)* in the immediate (between-group difference change score = 2.1, standard error (SE) 3.1, *p* = 0.50), or intermediate term (between-group difference change score = − 0.8, SE 3.2, *p* = 0.80) (narrative synthesis); ***catastrophizing and fear avoidance**** (benefit indicated by lower values)* in the immediate and intermediate terms (no plots, narrative synthesis); ***performance-based physical functioning*** in the immediate (between-group difference change scores: *usual pace gait speed:* 0.02 m/second, *p* = 0.29; *chair raise time:* − 0.8 s, *p* = 0.008; *stair climb time*: − 0.0 s, *p* = 0.99) or intermediate term (between-group difference change scores: *usual pace gait speed:* 0.00 m/second, *p* = 0.92; *chair raise time:* 0.1 s, *p* = 0.88; *stair climb time:* − 0.6 s, *p* = 0.61) (no plots, narrative synthesis); or ***harms*** (OR = 3.06, 95% CI 0.31 to 29.93) (plot 4.1.6.3). One participant experienced increased back pain. Authors reported no substantial intervention-associated adverse events.

Due to very low certainty evidence from 1 RCT [33], in older adults in the immediate term, it is uncertain whether mixed exercise worsens ***HRQoL PCS**** (scale 0 to 100, 0* = *poor quality of life)* (MD = − 6.56, 95% CI − 13.03 to − 0.10) (plot 4.1.3.3.1). Mixed exercise may make little or no difference to ***HRQoL MCS*** in the immediate (MD = − 1.05, 95% CI − 4.38 to 2.28) (plot 4.1.3.1.2); or intermediate term (***PCS:*** MD = − 2.31, 95% CI − 9.33 to 4.70; ***MCS:*** MD = − 0.83, 95% CI − 8.67 to 7.00) (plot 4.1.3.3.2).

### Mixed Exercise vs. Usual Care

#### All Adults

For outcomes that are based on RCTs of older adults only, results are reported under *older adults* below*.*

Due to very low certainty evidence, it is uncertain whether mixed exercise makes little or no difference to ***pain**** (scale 0 to 10, 0* = *no pain)* in the immediate (2 RCTs; MD = − 0.12, 95% CI − 0.91 to 0.68) (plot 4.2.1.1) [21, 24], short (1 RCT; MD = − 0.30, 95% CI − 1.66 to 1.06) (plot 4.2.1.2) [24], or intermediate term (1 RCT; MD = 0.00, 95% CI − 1.26 to 1.26) ( plot 4.2.1.3) [24]. It is uncertain whether mixed exercise reduces ***functional limitations**** (benefit indicated by lower values)* in the immediate term (2 RCTs; SMD = − 0.62, 95% CI − 0.96 to − 0.28) (plot 4.2.2.1) [21, 24].

#### Older Adults

Due to very low certainty evidence from one RCT of older adults [24], it is uncertain whether mixed exercise makes little or no difference to ***pain**** (scale 0 to 10, 0* = *no pain)* in the immediate (MD = − 0.80, 95% CI − 2.42 to 0.82) (plot 4.2.1.1), short (MD = − 0.30, 95% CI − 1.66 to 1.06) (plot 4.2.1.2), or intermediate term (MD = 0.00, 95% CI − 1.26 to 1.26) (plot 4.2.1.3); ***HRQoL**** (scale 0 to 1, 0* = *poor quality of life)* in the immediate (MD = 0.05, 95% CI − 0.01 to 0.11) (plot 4.2.3.1), short (MD = 0.04, 95% CI − 0.00 to 0.08) (plot 4.2.3.2), or intermediate term (MD = 0.05, 95% CI − 0.00 to 0.10) (plot 4.2.3.3); or ***self-efficacy**** (scale 0 to 60, 0* = *poor self-efficacy)* in the immediate (MD = 3.00, 95% CI − 2.39 to 8.39) (plot 4.2.4.1), short (MD = 3.00, 95% CI − 1.63 to 7.63) (plot 4.2.4.2), or intermediate term (MD = 4.00, 95% CI − 3.81 to 11.81) (plot 4.2.4.3). It is uncertain whether mixed exercise reduces ***functional limitations*** in the immediate term (SMD = − 0.86, 95% CI − 1.45 to − 0.27; *benefit indicated by lower values*) (plot 4.2.2.1). It is uncertain whether mixed exercise makes little or no difference to functional limitations *(scale 0 to 24, 0* = *no functional limitations)* in the short (MD = − 2.30, 95% CI − 4.92 to 0.32) (plot 4.2.2.2), or intermediate term (MD = − 2.50, 95% CI − 5.19 to 0.19) (plot 4.2.2.3).

### Pilates Exercises Versus Comparison Interventions With Isolated Exercise Effects

Due to very low certainty evidence from one RCT [25], it is uncertain whether Pilates reduces ***pain**** (scale 0 to 10, 0* = *no pain)* in the immediate term (MD = − 2.10, 95% CI − 3.07 to − 1.13) (plot 5.1.1.1), or makes little or no difference to pain in the intermediate term (MD = − 0.80, 95% CI − 1.75 to 0.15) ( plot 5.1.1.2). It is uncertain whether Pilates reduces ***functional limitations**** (scale 0 to 24, 0* = *no disability)* in the immediate (MD = − 3.50, 95% CI − 5.48 to − 1.52) (plot 5.1.2.1), or intermediate term (MD = − 2.20, 95% CI − 4.35 to − 0.05) (plot 5.1.2.2).

Due to very low certainty evidence from one RCT [25], it is uncertain whether Pilates makes little or no difference to ***fear avoidance**** (scale 17–68, benefit indicated by lower values)* in the immediate (MD = − 1.80, 95% CI − 5.12 to 1.52) (plot 5.1.3.1), or intermediate term (MD = − 0.80, 95% CI − 3.86 to 2.26) (plot 5.1.3.2); or to ***harms****:* authors reported no adverse events (no plot, narrative synthesis).

### Stretching, Flexibility Or Mobilizing Exercises Versus Comparison Interventions With Isolated Exercise Effects

Due to very low certainty evidence from one RCT [29], in the immediate term, it is uncertain whether stretching, flexibility or mobilizing exercise makes little or no difference to ***pain**** (scale 0 to 10, 0* = *no pain)* (MD = − 0.18, 95% CI − 1.61 to 1.25) (plot 6.1.1.1) or ***function**** (scale 0 to 100, 0* = *no disability)* (MD = − 3.97, 95% CI − 13.14 to 5.19) (plot 6.1.2.1).

### Yoga Versus Usual Care

The evidence is based on one RCT [32] and is very low certainty for all outcomes and time points. The results in this section are narratively synthesized (no forest plots).

It is uncertain whether yoga makes little or no difference to ***pain**** (scale 0 to 100, 0* = *no pain)* in the immediate (between-group difference in means = − 2.42, 95% CI − 4.97 to 0.12), intermediate (between-group difference in means = − 1.74, 95% CI − 4.32 to 0.84), or long term (between-group difference in means = − 0.73, 95% CI − 3.30 to 1.84); or ***HRQoL**** (scale 0 to 100, 0* = *poor quality of life)* in the immediate (between-group difference in means: ***PCS:*** 1.36, 95% CI − 0.70 to 3.41; ***MCS:*** 2.02, 95% CI − 0.31 to 4.35), intermediate (between-group difference in means: ***PCS:*** 1.24, 95% CI − 0.83 to 3.33; ***MCS:*** 2.02, 95% CI − 0.34 to 4.37), or long term (between-group difference in means: ***PCS:*** 0.80, 95% CI − 1.28 to 2.87; ***MCS:*** 0.42, 95% CI − 1.92 to 2.77). It is uncertain whether yoga reduces ***functional limitations**** (scale 0 to 24, 0* = *no disability)* in the immediate (between-group difference in means = − 2.17, 95% CI − 3.31 to − 1.03), intermediate (between-group difference in means = − 1.48, 95% CI − 2.62 to − 0.03), or long term (between-group difference in means = − 1.57, 95% CI − 2.71 to − 0.42). It is uncertain whether yoga improves ***self-efficacy**** (scale 0 to 60, 0* = *poor self-efficacy)* in the immediate (between-group difference in means = 2.96, 95% CI 0.35 to 5.58), or intermediate term (between-group difference in means = 3.33, 95% CI 0.68 to 5.97); or whether yoga makes little or no difference to self-efficacy in the long term (between-group difference in means = 1.75, 95% CI − 0.87 to 4.38). It is uncertain whether yoga increases ***minor adverse events/harms*** (i.e., increased pain) (OR 25.77, 95% CI 1.50 to 441.85) (plot 7.1.1); or whether yoga makes little or no difference to ***serious adverse events/harms*** (OR 0.51, 95% CI 0.05 to 5.70) (plot 7.1.2). Authors reported one participant in the yoga group experienced severe pain (typically does after physical activity).

### Motor Control Exercise Versus Sham

The evidence is based on one RCT [22] and is very low certainty for all outcomes. It is uncertain whether motor control exercise reduces ***pain**** (scale 0 to 10, 0* = *no pain)* in the immediate (MD = − 1.00, 95% CI − 1.85 to − 0.15) (plot 8.1.1.1) or long term (MD = − 1.30, 95% CI − 2.13 to − 0.47) (plot 8.1.1.3), or whether it makes little or no difference to pain in the intermediate term (MD = − 0.60, 95% CI − 1.46 to 0.26) (plot 8.1.1.2). It is uncertain whether motor control exercise reduces ***functional limitations**** (scale 0 to 24, 0* = *no disability)* in the immediate term (MD = − 2.30, 95% CI − 4.26 to − 0.34) (plot 8.1.2.1); or whether it makes little or no difference in the intermediate (MD = − 1.90, 95% CI − 4.06 to 0.26) (plot 8.1.2.2), or long term (MD = − 0.90, 95% CI − 3.15 to 1.35) (plot 8.1.2.3). It is uncertain whether motor control exercise makes little or no difference to ***harms*** (OR = 1.52, 95% CI 0.25 to 9.36) (plot 8.1.3). Authors reported all adverse events were temporary exacerbations of pain.

### Pooled Analysis of All Exercise Types Versus Comparison Interventions With Isolated Exercise Effects

We conducted a post hoc analysis by pooling all exercise types since only 1–3 RCTs were identified for each exercise type and none on their own showed a clear benefit. To be included in this analysis, data from two or more of the eight exercise types had to be available per comparison, outcome, and time point. Otherwise, findings of the individual eight exercise types have been reported in the eight previous comparisons.

**Fig. 2:**
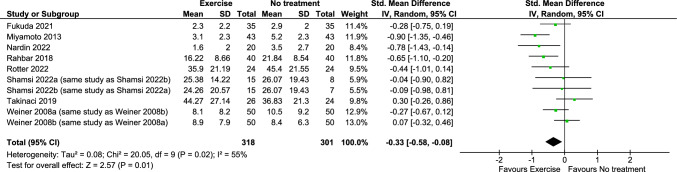
Any exercise versus comparison interventions where the attributable effect of exercise could be isolated for pain in the immediate term (closest to 2 weeks)

Exercise probably reduces ***pain**** (benefit indicated by lower values)* in the immediate term (8 RCTs; SMD = − 0.33, 95% CI − 0.58 to − 0.08; moderate certainty evidence) (Fig. 2) (plot 9.1.1.1) [23, 25–29, 31, 33]. Due to very low certainty evidence, it is uncertain whether exercise makes little or no difference to ***pain**** (scale 0 to 10, 0* = *no pain)* in the short (2 RCTs; MD = − 0.68, 95% CI − 1.82 to 0.46) (plot 9.1.1.2) [28, 31], or long term (1 RCT; between-group MD = 8.88, 95% CI − 0.36 to 18.13; scale 0 to 100, 0 = no pain) (no plot, narrative synthesis) [30]. Exercise may make little or no difference to ***pain**** (benefit indicated by lower values)* in the intermediate term (4 RCTs; SMD = − 0.08, 95% CI − 0.29 to 0.13; low certainty evidence) (plot 9.1.1.3) [23, 25, 31, 33].

**Fig. 3:**
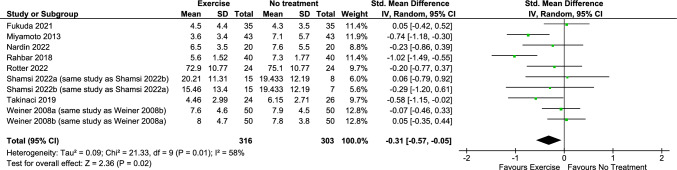
Any exercise versus comparison interventions where the attributable effect of exercise could be isolated for pain in the immediate term (closest to 2 weeks)

Exercise probably reduces ***functional limitations**** (benefit indicated by lower values)* in the immediate term (8 RCTs; SMD = − 0.31, 95% CI − 0.57 to − 0.05; moderate certainty evidence) (Fig. 3) (plot 9.1.2.1) [23, 25–29, 31, 33]. Due to very low certainty evidence, it is uncertain whether exercise makes little or no difference to function in the short (2 RCTs; SMD = − 0.26, 95% CI − 0.67 to 0.14; *benefit indicated by lower values*) (plot 9.1.2.2) [28, 31], or long term (1 RCT; between-group MD = 1.62, 95% CI − 0.06 to 3.31; *scale 0 to 24, 0* = *no functional limitations*) (no plot, narrative synthesis) [30]. Exercise may make little or no difference to function in the intermediate term (4 RCTs; SMD = − 0.16, 95% CI − 0.39 to 0.07; *benefit indicated by lower values;* low certainty evidence) (plot 9.1.2.3) [23, 25, 31, 33].

In the immediate term, it is uncertain whether exercise makes little or no difference to ***HRQoL**** (scale 0 to 100, 0* = *poor quality of life)* for the ***PCS*** (2 RCTs; MD = − 2.31, 95% CI − 10.36 to 5.75; very low certainty evidence) (plot 9.1.3.1.1) [28, 33]. Exercise may make little or no difference for the ***MSC*** (2 RCTs; MD = − 1.11, 95% CI − 3.67 to 1.45; low certainty evidence) (plot 9.1.3.1.2) [28, 33]. It is uncertain whether exercise makes little or no difference to ***harms*** (2 RCTs; OR = 4.24, 95% CI 0.69 to 25.95; very low certainty evidence) (plot 9.1.4) [30, 33]. Harms were minor adverse events including back and knee pain.

### Subgroup, Sensitivity and Supplementary Evidence Analyses

For the primary evidence synthesis, we did not conduct subgroup analysis for exercise dosage or intensity because there were too few RCTs (1–3) per comparison with little variation in dosage or intensity between RCTs. Additionally, we did not conduct sensitivity analyses removing the overall unclear ROB RCTs as most were given this rating (11/13, 85%).

In the supplementary evidence synthesis (see Online Resource 8), our findings aligned with our primary synthesis, except the certainty of evidence was lower (due to including RCTs rated as having high overall ROB). The supplementary evidence synthesis included 68 RCTs (13 identified from our primary synthesis [21–33], and 55 identified for the supplementary synthesis [37–91]. The 68 RCTs included a total of 4195 participants (ranging from 14 to 313 participants per RCT). The trials were conducted in high to upper-middle income economies: Australia (3 RCTs) [22, 72, 90], Brazil (7 RCTs) [23, 25, 26, 47, 48, 74, 77], Canada (2 RCTs) [64, 67], China (8 RCTs) [53, 54, 68–71, 83, 88], France (1 RCT) [78], Germany (3 RCTs) [28, 49, 50], Italy (1 RCT) [45], Japan (1 RCT) [24], Malaysia (1 RCT) [37], Netherlands (1 RCT) [29], South Korea (4 RCTs) [65, 66, 76, 86], Thailand (1 RCT) [59], Turkey (1 RCT) [31], United Kingdom (1 RCT) [32], and the United States (2 RCTs) [33, 46]; and low to lower-middle income economies: Egypt (2 RCTs) [37, 52], India (5 RCTs) [21, 51, 55, 79, 87], Iran (18 RCTs) [27, 29, 40, 42–44, 56–58, 62, 63, 72, 75, 81, 81, 83, 84, 89], Nigeria (2 RCTs) [59, 91], and Pakistan (4 RCTs) [39, 41, 61, 80]. The mean age of participants ranged from 20.4 to 74.3 years; nine RCTs with 524 participants total assessed older adults aged ≥ 60 years [24, 33, 45, 48, 54, 72, 76, 89, 90].

In the subgroup and/or sensitivity analyses conducted in both the primary and supplementary evidence syntheses, for all comparisons and outcomes, subgroup differences could not be explained and/or the differences between subgroups would likely not result in different recommendations for different subgroups. This was mostly due to the low or very low certainty evidence and the absence of or unimportant differences between the intervention and comparison groups (see Online Resources 7 and 8).

## Discussion

The evidence regarding the benefits and harms of structured exercise programs for CPLBP in adults is based on 13 RCTs deemed as low or unclear ROB with a total of 1362 participants. Of these, two RCTs (n = 252) assessed adults aged ≥ 60 years. The eight exercise types assessed were aerobic exercise, core strengthening, muscle strengthening, mixed exercise, Pilates, stretching/flexibility/mobilizing exercise, yoga, and motor control exercise. Most of the RCTs (11, 85%) were rated as unclear overall ROB (concerns primarily with performance and detection bias). The certainty for the evidence related to individual exercise types was low or very low. Compared to no intervention, pain reduction was associated with aerobic exercise in the immediate and short terms, and Pilates in the immediate term, and motor control exercise vs. sham in the immediate and long terms. Improved function was associated with mixed exercise vs. usual care, and Pilates vs. no intervention in the immediate term. Temporary increased minor pain was associated with mixed exercise vs. no intervention, and yoga vs. usual care; no harms were reported with Pilates vs. no intervention. Little to no differences were found for other comparisons and outcomes.

When pooling all exercise types together based on the 13 RCTs, we found moderate certainty evidence indicating that in the immediate term, exercise (including aerobic, motor control, Pilates, yoga, core strengthening, and mixed exercise) improves pain in adults, and function in adults and older adults. Little or no difference was found between groups for the other outcomes (HRQoL, depression, self-efficacy, catastrophizing, fear avoidance, and performance-based physical functioning in older adults). Taken together, the findings from our primary synthesis, supplementary synthesis, and the work by Hayden et al. [2, 4] are consistent.

Our systematic review has several strengths. First, our international team had clinical and methodological expertise regarding LBP, systematic reviews, evidence syntheses, and answering important public health questions from the WHO. Second, our review process involved conducting comprehensive literature searches without any language restrictions. Third, during the screening and ROB assessments, a core team member (with the most expertise and reliability in screening and ROB evaluations) was involved in each screening and ROB pair. Fourth, our ROB assessments did not rely on summary scores or the number of items at ROB. Instead, we created supplementary guidance forms based on the ROB 1 criteria [13, 14], which allowed reviewers to consider critical flaws in the studies [6]. Our use of these forms resulted in high agreement on ROB judgements. Fifth, we maintained transparency throughout the review process, providing detailed ROB assessments and footnotes for grading the certainty of the evidence (see Online Resources 2, 5, 8). These notes give readers a better understanding of our judgements and allow them to reach their own conclusions.

Our review has some limitations. One limitation is that we did not search the grey literature, which could introduce publication bias as studies published in peer-reviewed journals tend to report larger intervention effects than those in the grey literature [92]. We tried to mitigate this by searching for unpublished RCTs in the WHO ICTRP registry and contacting authors of unpublished RCTs. Moreover, unpublished studies are known to represent a small proportion of studies and rarely impact results and conclusions [93]. However, it may be important to include such studies in limited scenarios or where there are potential conflicts of interest in published research [93].

We identified several key gaps in the evidence across different exercise comparisons: 1) lack of studies examining the effects of exercise on anxiety symptoms and social participation (including work); 2) inability to assess whether the benefits or harms of exercise interventions vary by gender/sex or race/ethnicity; 3) insufficient studies to evaluate the impact of leg pain/symptoms on exercise benefits or harms, as well as differences in higher versus lower income countries; 4) inability to examine the influence of intervention-level characteristics, such as exercise specificity, tailored approaches, supervision level, and group versus individual delivery, on benefits and harms; 5) limited evidence on the benefits or harms of specific exercise types in older adults, including aerobic exercise, core strengthening, muscle strength training, Pilates, stretching, flexibility or mobilizing exercises, yoga, and motor control exercises; 6) few studies assessing the impact of exercise on quality of life and psychological outcomes (depression, fear avoidance, catastrophizing, self-efficacy, anxiety), with comparatively less evidence available for older individuals; 7) limited understanding of the effects of exercise in vulnerable populations, such as older adults and those in low-income settings, who are more likely to experience persistent disability from low back pain. Additionally, exercise's effects are modest, suggesting a need for multifaceted interventions.

## Conclusion

When assessing individual exercise types, based on low or very low certainty evidence, pain reduction was associated with aerobic exercise, Pilates and motor control exercise; improved function was associated with mixed exercise and Pilates. A temporary increase in minor pain was associated with mixed exercise and yoga. Little to no difference was found for other comparisons and outcomes. When pooling exercise types, based on moderate certainty evidence, exercise was shown to be beneficial in improving pain and function in adults and older adults. Exercise prescription should be considered based on patient preferences, availability of exercise type, costs, and other contextual factors. Harms should be further investigated systematically.

### Supplementary Information

Below is the link to the electronic supplementary material.

Supplementary file1 (DOCX 30939 KB)

## Data Availability

The datasets generated during and/or analysed during the primary analysis of the current study are available from the corresponding author on reasonable request.
