# Supplementary material for: Systematic Review to Inform a World Health Organization (WHO) Clinical Practice Guideline: Benefits and Harms of Structured Exercise Programs for Chronic Primary Low Back Pain in Adults
Source: J Occup Rehabil. 2023 Nov 22;33(4):636–50. doi: 10.1007/s10926-023-10124-4 (PMC10684665; doi:10.1007/s10926-023-10124-4)
Supplement: Supplementary file 1 — Supplementary file1 (DOCX 30939 KB) [file 10926_2023_10124_MOESM1_ESM.docx]

Systematic review to inform a World Health Organization (WHO) clinical practice guideline: Benefits and harms of structured exercise programs for chronic primary low back pain in adults

Verville L, Ogilvie R, Hincapié CA, Southerst D, Yu H, Bussières A, Gross DP, Pereira P, Mior S, Tricco AC, Cedraschi C, Brunton G, Nordin M, Connell G, Wong JJ, Shearer HM, Lee JGB, Wang D, Hayden JA, Cancelliere C

Corresponding authors:

Carol Cancelliere

Institute of Disability and Rehabilitation Research and Faculty of Health Sciences, Ontario Tech University, Oshawa, Ontario, Canada

Email: [carolina.cancelliere@ontariotechu.ca](mailto:carolina.cancelliere@ontariotechu.ca)

Cesar A. Hincapié

EBPI-UWZH Musculoskeletal Epidemiology Research Group, University of Zurich and Balgrist University Hospital, Zurich, Switzerland

Epidemiology, Biostatistics and Prevention Institute (EBPI), University of Zurich, Zurich, Switzerland

University Spine Centre Zurich (UWZH), Balgrist University Hospital and University of Zurich, Zurich, Switzerland

Email: [cesar.hincapie@uzh.ch](mailto:cesar.hincapie@uzh.ch)

**Online Resource 1.** Literature search strategies

**MEDLINE (Ovid)**

| 1 | randomized controlled trial.pt. |
| --- | --- |
| 2 | controlled clinical trial.pt. |
| 3 | pragmatic clinical trial.pt. |
| 4 | random*.ti,ab. |
| 5 | placebo.ab,ti. |
| 6 | drug therapy.fs. |
| 7 | trial.ab,ti. |
| 8 | groups.ab,ti. |
| 9 | or/1-8 |
| 10 | (animals not (humans and animals)).sh. |
| 11 | 9 not 10 |
| 12 | exp Back Pain/ |
| 13 | Intervertebral Disc Displacement/ |
| 14 | exp Sciatic Neuropathy/ |
| 15 | exp Spondylosis/ |
| 16 | (back ache* or backache* or back disorder* or back pain*).tw,kw,kf. |
| 17 | coccydynia.tw,kw,kf. |
| 18 | ((disc? or disk?) adj1 (degenerat* or displace* or hernia* or prolapse* or slipped)).tw,kw,kf. |
| 19 | dorsalgia.tw,kw,kf. |
| 20 | (lumb* adj4 pain).tw,kw,kf. |
| 21 | lumbago.tw,kw,kf. |
| 22 | (sciatic neuropathy or sciatica or ischialgia).tw,kw,kf. |
| 23 | (spondylosis or spondylolysis or spondylolisthesis).tw,kw,kf. |
| 24 | or/12-23 |
| 25 | exp Exercise/ |
| 26 | exp Exercise Therapy/ |
| 27 | exp Exercise Movement Techniques/ |
| 28 | Physical Therapy Modalities/ |
| 29 | exp Recreation/ |
| 30 | Recreation Therapy/ |
| 31 | exp Physical Fitness/ |
| 32 | exercis*.tw,kw,kf. |
| 33 | (kinesiotherapy or recreation*).tw,kw,kf. |
| 34 | McKenzie.tw,kw,kf. |
| 35 | Alexander.tw,kw,kf. |
| 36 | William.tw,kw,kf. |
| 37 | Feldenkrais.tw,kw,kf. |
| 38 | (McGill adj5 (method or technique)).tw,kw,kf. |
| 39 | (training adj2 (strength* or physical or fitness or core or ergonomic* or musc* or spine or spinal or balance or stabil*)).tw,kw,kf. |
| 40 | ((core or musc*) adj2 (strengthen* or stabiliz* or stabilis* or stability or endurance or condition*)).tw,kw,kf. |
| 41 | functional restoration.tw,kw,kf. |
| 42 | pilates*.tw,kw,kf. |
| 43 | (yoga or hatha or ashtanga or bikram or iyengar or kripalu or kundalini or sivananda or vinyasa or raja or radja or bhakti or jnana or kriya or karma or yama or niyama or asana or pranayama or pratyahara or dharana or dhyana or samadhi or bandha or mudra or yin).tw,kw,kf. |
| 44 | aerobic*.tw,kw,kf. |
| 45 | (high intensity interval training or hiit).tw,kw,kf. |
| 46 | (walk* or run or running or jog or jogging or sport* or cycling or biking or swim* or dance or dancing or gymnastic* or boxing or kickboxing or stretch*).tw,kw,kf. |
| 47 | (aquacise or aquacize or aquasize or aquafit* or zumba or barre).tw,kw,kf. |
| 48 | (tai chi or tai ji or taiji or taijiquan or taijizhang).tw,kw,kf. |
| 49 | eldoa.tw,kw,kf. |
| 50 | (glad adj5 (hip? or knee? or osteoarthritis)).tw,kw,kf. |
| 51 | (otago adj5 (program* or balance or strength or training)).tw,kw,kf. |
| 52 | (bone fit or bonefit).tw,kw,kf. |
| 53 | walk tall.tw,kw,kf. |
| 54 | (dynamic neuromuscular stabili?ation or dns).tw,kw,kf. |
| 55 | active rehabilitation.tw,kw,kf. |
| 56 | or/25-55 |
| 57 | Alexander Disease/ |
| 58 | Williams Syndrome/ |
| 59 | or/57-58 |
| 60 | 56 not 59 |
| 61 | 11 and 24 and 60 |

**Embase (Embase Elsevier.com)**

| 1 | 'randomized controlled trial'/de |
| --- | --- |
| 2 | 'controlled clinical trial'/exp |
| 3 | 'controlled study'/de |
| 4 | double blind procedure'/de |
| 5 | 'single blind procedure'/de |
| 6 | 'crossover procedure'/de |
| 7 | 'placebo'/de |
| 8 | 'randomization'/de |
| 9 | random*:ti,ab |
| 10 | placebo$:ti,ab |
| 11 | allocat*:ti,ab |
| 12 | assign*:ti,ab |
| 13 | blind*:ti,ab |
| 14 | ('cross-over' OR crossover):ti,ab |
| 15 | (compare OR compared OR comparing OR comparison OR comparative):ti,ab |
| 16 | (controlled NEAR/7 (study OR design OR trial)):ti,ab |
| 17 | ((singl* OR doubl* OR trebl* OR tripl*) NEAR/7 (blind* OR mask*)):ti,ab |
| 18 | trial:ti,ab |
| 19 | #1 OR #2 OR #3 OR #4 OR #5 OR #6 OR #7 OR #8 OR #9 OR #10 OR #11 OR #12 OR #13 OR #14 OR #15 OR #16 OR #17 OR #18 |
| 20 | animal'/exp OR 'invertebrate'/exp OR 'animal experiment'/de OR 'animal model'/de OR 'animal tissue'/de OR 'animal cell'/de OR 'nonhuman'/de |
| 21 | human'/de OR 'normal human'/de OR 'human cell'/de |
| 22 | #20 AND #21 |
| 23 | #20 NOT #22 |
| 24 | #19 NOT #23 |
| 25 | 'backache'/exp |
| 26 | 'intervertebral disk hernia'/exp |
| 27 | 'ischialgia'/exp |
| 28 | 'sciatic neuropathy'/exp |
| 29 | 'sciatica'/exp |
| 30 | 'spondylosis'/exp |
| 31 | ('back ache*' OR backache* OR 'back disorder*' OR 'back pain*'):ti,ab,kw |
| 32 | coccydynia:ti,ab,kw |
| 33 | ((disc$ OR disk$) NEAR/1 (degenerat* OR displace* OR hernia* OR prolapse* OR slipped)):ti,ab,kw |
| 34 | dorsalgia:ti,ab,kw |
| 35 | (lumb* NEAR/4 pain):ti,ab,kw |
| 36 | lumbago:ti,ab,kw |
| 37 | ('sciatic neuropathy' OR sciatica OR ischialgia):ti,ab,kw |
| 38 | (spondylosis OR spondylolysis OR spondylolisthesis):ti,ab,kw |
| 39 | #25 OR #26 OR #27 OR #28 OR #29 OR #30 OR #31 OR #32 OR #33 OR #34 OR #35 OR #36 OR #37 OR #38 |
| 40 | 'exercise'/exp |
| 41 | 'fitness'/de |
| 42 | 'kinesiotherapy'/exp |
| 43 | 'physical activity'/exp |
| 44 | 'recreation'/exp |
| 45 | 'recreational therapy'/exp |
| 46 | exercis*:ti,ab,kw |
| 47 | (kinesiotherapy OR recreation*):ti,ab,kw |
| 48 | McKenzie:ti,ab,kw |
| 49 | Alexander:ti,ab,kw |
| 50 | William:ti,ab,kw |
| 51 | Feldenkrais:ti,ab,kw |
| 52 | (McGill NEAR/5 (method OR technique)):ti,ab,kw |
| 53 | (training NEAR/2 (strength* OR physical OR fitness OR core OR ergonomic* OR musc* OR spine OR spinal OR balance OR stabil*)):ti,ab,kw |
| 54 | ((core OR musc*) NEAR/2 (strengthen* OR stabiliz* OR stabilis* OR stability OR endurance or condition*)):ti,ab,kw |
| 55 | 'functional restoration':ti,ab,kw |
| 56 | pilates*:ti,ab,kw |
| 57 | (yoga OR hatha OR ashtanga OR bikram OR iyengar OR kripalu OR kundalini OR sivananda OR vinyasa OR raja OR radja OR bhakti OR jnana OR kriya OR karma OR yama OR niyama OR asana OR pranayama OR pratyahara OR dharana OR dhyana OR samadhi OR bandha OR mudra OR yin):ti,ab,kw |
| 58 | aerobic*:ti,ab,kw |
| 59 | ('high intensity interval training' OR hiit):ti,ab,kw |
| 60 | (walk* OR run OR running OR jog OR jogging OR sport* OR cycling OR biking OR swim* OR dance OR dancing OR gymnastic* OR boxing OR kickboxing OR stretch*):ti,ab,kw |
| 61 | (aquacise OR aquacize OR aquasize OR aquafit* OR zumba OR barre):ti,ab,kw |
| 62 | ('tai chi' OR 'tai ji' OR taiji OR taijiquan OR taijizhang):ti,ab,kw |
| 63 | eldoa:ti,ab,kw |
| 64 | (glad NEAR/5 (hip$ OR knee$ OR osteoarthritis)):ti,ab,kw |
| 65 | (otago NEAR/5 (program* OR balance OR strength OR training)):ti,ab,kw |
| 66 | ('bone fit' OR bonefit):ti,ab,kw |
| 67 | 'walk tall':ti,ab,kw |
| 68 | ('dynamic neuromuscular stabili?ation' OR dns):ti,ab,kw |
| 69 | 'active rehabilitation':ti,ab,kw |
| 70 | #40 OR #41 OR #42 OR #43 OR #44 OR #45 OR #46 OR #47 OR #48 OR #49 OR #50 OR #51 OR #52 OR #53 OR #54 OR #55 OR #56 OR #57 OR #58 OR #59 OR #60 OR #61 OR #62 OR #63 OR #64 OR #65 OR #66 OR #67 OR #68 OR #69 |
| 71 | 'alexander disease'/exp |
| 72 | 'williams beuren syndrome'/exp |
| 73 | #71 OR #72 |
| 74 | #70 NOT #73 |
| 75 | #24 AND #39 AND #74 |

**CENTRAL (Cochrane Library)**

| 1 | MeSH descriptor: [Back Pain] explode all trees |
| --- | --- |
| 2 | MeSH descriptor: [Intervertebral Disc Displacement] explode all trees |
| 3 | MeSH descriptor: [Sciatic Neuropathy] explode all trees |
| 4 | MeSH descriptor: [Spondylosis] explode all trees |
| 5 | ("back ache*" or backache* or "back disorder*" or "back pain*"):ti,ab,kw |
| 6 | coccydynia:ti,ab,kw |
| 7 | ((disc* or disk*) near/1 (degenerat* or displace* or hernia* or prolapse* or slipped)):ti,ab,kw |
| 8 | dorsalgia:ti,ab,kw |
| 9 | (lumb* near/4 pain):ti,ab,kw |
| 10 | lumbago:ti,ab,kw |
| 11 | ("sciatic neuropathy" or sciatica or ischialgia):ti,ab,kw |
| 12 | (spondylosis or spondylolysis or spondylolisthesis):ti,ab,kw |
| 13 | or #1-#12 |
| 14 | MeSH descriptor: [Exercise] explode all trees |
| 15 | MeSH descriptor: [Exercise Therapy] explode all trees |
| 16 | MeSH descriptor: [Exercise Movement Techniques] explode all trees |
| 17 | MeSH descriptor: [Physical Therapy Modalities] this term only |
| 18 | MeSH descriptor: [Recreation] explode all trees |
| 19 | MeSH descriptor: [Recreation Therapy] this term only |
| 20 | MeSH descriptor: [Physical Fitness] explode all trees |
| 21 | exercis*:ti,ab,kw |
| 22 | (kinesiotherapy or recreation*):ti,ab,kw |
| 23 | McKenzie:ti,ab,kw |
| 24 | Alexander:ti,ab,kw |
| 25 | William:ti,ab,kw |
| 26 | Feldenkrais:ti,ab,kw |
| 27 | (McGill near/5 (method or technique)):ti,ab,kw |
| 28 | (training near/2 (strength* or physical or fitness or core or ergonomic* or musc* or spine or spinal or balance or stabil*)):ti,ab,kw |
| 29 | ((core or musc*) near/2 (strengthen* or stabiliz* or stabilis* or stability or endurance or condition*)):ti,ab,kw |
| 30 | "functional restoration":ti,ab,kw |
| 31 | pilates*:ti,ab,kw |
| 32 | (yoga or hatha or ashtanga or bikram or iyengar or kripalu or kundalini or sivananda or vinyasa or raja or radja or bhakti or jnana or kriya or karma or yama or niyama or asana or pranayama or pratyahara or dharana or dhyana or samadhi or bandha or mudra or yin):ti,ab,kw |
| 33 | aerobic*:ti,ab,kw |
| 34 | ("high intensity interval training" or hiit):ti,ab,kw |
| 35 | (walk* or run or running or jog or jogging or sport* or cycling or biking or swim* or dance or dancing or gymnastic* or boxing or kickboxing or stretch*):ti,ab,kw |
| 36 | (aquacise or aquacize or aquasize or aquafit* or zumba or barre):ti,ab,kw |
| 37 | ("tai chi" or "tai ji" or taiji or taijiquan or taijizhang):ti,ab,kw |
| 38 | eldoa:ti,ab,kw |
| 39 | (glad near/5 (hip* or knee* or osteoarthritis)):ti,ab,kw |
| 40 | (otago near/5 (program* or balance or strength or training)):ti,ab,kw |
| 41 | ("bone fit" or bonefit):ti,ab,kw |
| 42 | "walk tall":ti,ab,kw |
| 43 | ("dynamic neuromuscular stabili?ation" or dns):ti,ab,kw |
| 44 | "active rehabilitation":ti,ab,kw |
| 45 | or #14-#44 |
| 46 | MeSH descriptor: [Alexander Disease] this term only |
| 47 | MeSH descriptor: [Williams Syndrome] this term only |
| 48 | or #46-#47 |
| 49 | #45 not #48 |
| 50 | #13 and #49 |

**Online Resource 2.** Risk of bias assessment for the included RCTs (n=13)

| Study | Random allocation | Concealed allocation | Participant blinding | Provider blinding | Assessor blinding | Drop-outs | Intention-to-treat analysis | Selective reporting | Similar groups (baseline) | Co-interventions | Compliance | Timing of outcomes | Other biases | Overall ROB Rating |
| --- | --- | --- | --- | --- | --- | --- | --- | --- | --- | --- | --- | --- | --- | --- |
|  | Selection bias | | Performance bias | | Detection bias | Attrition bias | | Reporting bias | Selection bias | Other biases | | | |
| Chhabra 2018 (43412) | 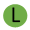 | 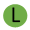 | 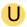 | 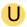 | 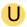 | 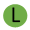 | 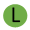 | 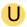 | 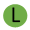 | 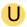 | 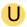 | 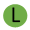 | 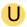 | Unclear |
| Costa 2009 (9861) | 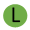 | 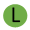 | 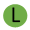 | 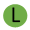 | 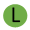 | 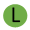 | 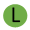 | 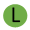 | 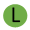 | 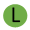 | 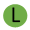 | 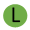 | 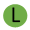 | Low |
| Fukuda 2021 (53055) | 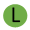 | 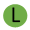 | 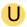 | 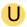 | 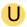 | 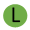 | 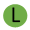 | 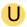 | 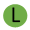 | 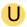 | 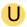 | 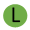 | 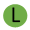 | Unclear |
| Jinnouchi 2020 (47336) | 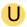 | 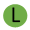 | 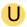 | 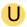 | 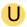 | 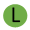 | 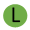 | 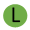 | 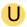 | 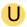 | 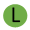 | 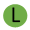 | 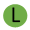 | Unclear |
| Miyamoto 2013 (9791) | 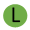 | 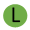 | 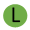 | 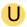 | 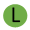 | 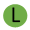 | 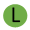 | 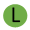 | 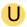 | 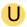 | 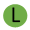 | 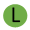 | 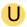 | Unclear |
| Nardin 2022 (53154) | 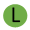 | 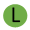 | 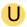 | 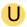 | 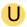 | 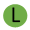 | 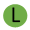 | 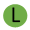 | 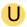 | 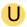 | 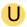 | 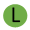 | 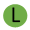 | Unclear |
| Rahbar 2018 (43745) | 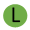 | 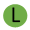 | 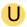 | 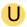 | 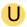 | 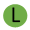 | 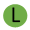 | 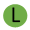 | 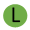 | 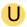 | 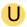 | 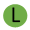 | 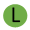 | Unclear |
| Rotter 2022 (53220) | 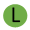 | 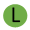 | 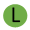 | 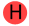 | 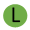 | 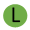 | 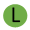 | 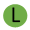 | 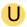 |  |  |  |  | Unclear |
| Shamsi 2022 (53159) |  |  |  |  |  |  |  |  |  |  |  |  |  | Unclear |
| Smeets 2008 (16226) |  |  |  |  |  |  |  |  |  |  |  |  |  | Low |
| Takinaci 2019 (44427) |  |  |  |  |  |  |  |  |  |  |  |  |  | Unclear |
| Tilbrook 2011 (17167) |  |  |  |  |  |  |  |  |  |  |  |  |  | Unclear |
| Weiner 2008 (6068) |  |  |  |  |  |  |  |  |  |  |  |  |  | Unclear |

Risk of bias assessment conducted using ROB1 criteria.

First author last name and year (reference ID number).

H=high risk of bias, L=low risk of bias, ROB=risk of bias, U=Unclear

Risk of bias graph for the included RCTs: review authors’ judgements about each risk of bias item presented as percentages across all included RCTs

Risk of bias assessment for the eligible RCTs rated as high risk of bias and excluded from synthesis (n= 111)

| Study | Random allocation | Concealed allocation | Participant blinding | Provider blinding | Assessor blinding | Drop-outs | Intention-to-treat analysis | Selective reporting | Similar groups (baseline) | Co-interventions | Compliance | Timing of outcomes | Other biases | Overall ROB Rating |
| --- | --- | --- | --- | --- | --- | --- | --- | --- | --- | --- | --- | --- | --- | --- |
|  | Selection bias | | Performance bias | | Detection bias | Attrition bias | | Reporting bias | Selection bias | Other biases | | | |  |
| Abadi 2019 (46204)[1] |  |  |  |  |  |  |  |  |  |  |  |  |  | High |
| Abdel-Azeim 2021 (53158)[2] |  |  |  |  |  |  |  |  |  |  |  |  |  | High |
| Afzal 2022 (53165)[3] |  |  |  |  |  |  |  |  |  |  |  |  |  | High |
| Ahmadizadeh 2019 (46220)[4] |  |  |  |  |  |  |  |  |  |  |  |  |  | High |
| Ain 2019 (46853)[5] |  |  |  |  |  |  |  |  |  |  |  |  |  | High |
| Alayat 2014 (11899)[6] |  |  |  |  |  |  |  |  |  |  |  |  |  | High |
| Alikhajeh 2020 (46957)[7] |  |  |  |  |  |  |  |  |  |  |  |  |  | High |
| Alvani 2021 (1776)[8] |  |  |  |  |  |  |  |  |  |  |  |  |  | High |
| Ansari 2021 (46984)[9] |  |  |  |  |  |  |  |  |  |  |  |  |  | High |
| Arampatzis 2017 (40745)[10] |  |  |  |  |  |  |  |  |  |  |  |  |  | High |
| Barni 2018 (43368)[11] |  |  |  |  |  |  |  |  |  |  |  |  |  | High |
| Bruehl 2020 (47043)[12] |  |  |  |  |  |  |  |  |  |  |  |  |  | High |
| Chen 2014 (8990)[13] |  |  |  |  |  |  |  |  |  |  |  |  |  | High |
| Cortell-Tormo 2018 (41536)[14] |  |  |  |  |  |  |  |  |  |  |  |  |  | High |
| Cruz-Diaz 2016 (40738)[15] |  |  |  |  |  |  |  |  |  |  |  |  |  | High |
| Cruz-Diaz 2017 (41532)[16] |  |  |  |  |  |  |  |  |  |  |  |  |  | High |
| Cuesta-Vargas 2012 (17147)[17] |  |  |  |  |  |  |  |  |  |  |  |  |  | High |
| da Luz 2019 (46326)[18] |  |  |  |  |  |  |  |  |  |  |  |  |  | High |
| da Silva 2014 (46060)[19] |  |  |  |  |  |  |  |  |  |  |  |  |  | High |
| Dalichau 2003 (1621)[20] |  |  |  |  |  |  |  |  |  |  |  |  |  | High |
| Dalichau 2005 (1865)[21] |  |  |  |  |  |  |  |  |  |  |  |  |  | High |
| Dineshkumar 2015 (46063)[22] |  |  |  |  |  |  |  |  |  |  |  |  |  | High |
| Djavid 2007 (9826)[23] |  |  |  |  |  |  |  |  |  |  |  |  |  | High |
| Esmaili 2016 (1871)[24] |  |  |  |  |  |  |  |  |  |  |  |  |  | High |
| Fouda 2021 (1625)[25] |  |  |  |  |  |  |  |  |  |  |  |  |  | High |
| Frost 1995 (17107)[26] |  |  |  |  |  |  |  |  |  |  |  |  |  | High |
| Gao 2018 (45037)[27] |  |  |  |  |  |  |  |  |  |  |  |  |  | High |
| Ge 2022 (53137)[28] |  |  |  |  |  |  |  |  |  |  |  |  |  | High |
| Gladwell 2006 (13530)[29] |  |  |  |  |  |  |  |  |  |  |  |  |  | High |
| Groessl 2017 (41398)[30] |  |  |  |  |  |  |  |  |  |  |  |  |  | High |
| Gupta 2019 (44157)[31] |  |  |  |  |  |  |  |  |  |  |  |  |  | High |
| Gur 2003 (17109)[32] |  |  |  |  |  |  |  |  |  |  |  |  |  | High |
| Hall 2011 (13730)[33] |  |  |  |  |  |  |  |  |  |  |  |  |  | High |
| Harris 2017 (40692)[34] |  |  |  |  |  |  |  |  |  |  |  |  |  | High |
| Harts 2008 (13808)[35] |  |  |  |  |  |  |  |  |  |  |  |  |  | High |
| Hasanpour-Dehkordi 2017 (41378)[36] |  |  |  |  |  |  |  |  |  |  |  |  |  | High |
| Hatefi 2021 (1879)[37] |  |  |  |  |  |  |  |  |  |  |  |  |  | High |
| Heidari 2018 (43527)[38] |  |  |  |  |  |  |  |  |  |  |  |  |  | High |
| Hemmati 2011 (45008)[39] |  |  |  |  |  |  |  |  |  |  |  |  |  | High |
| Hildebrandt 2000 (1046)[40] |  |  |  |  |  |  |  |  |  |  |  |  |  | High |
| Idowu 2020 (1650)[41] |  |  |  |  |  |  |  |  |  |  |  |  |  | High |
| Joseph 2018 (43560)[42] |  |  |  |  |  |  |  |  |  |  |  |  |  | High |
| Kanwal 2021 (1937)[43] |  |  |  |  |  |  |  |  |  |  |  |  |  | High |
| Karimi 2009 (1845)[44] |  |  |  |  |  |  |  |  |  |  |  |  |  | High |
| Karimzadeh 2016 (41261)[45] |  |  |  |  |  |  |  |  |  |  |  |  |  | High |
| Keane 2017 (40013)[46] |  |  |  |  |  |  |  |  |  |  |  |  |  | High |
| Kell 2009 (14331)[47] |  |  |  |  |  |  |  |  |  |  |  |  |  | High |
| Kell 2011 (45010)[48] |  |  |  |  |  |  |  |  |  |  |  |  |  | High |
| Kim 2015 (40015)[49] |  |  |  |  |  |  |  |  |  |  |  |  |  | High |
| Kim 2020 (47380)[50] |  |  |  |  |  |  |  |  |  |  |  |  |  | High |
| Kim 2020 (47385)[51] |  |  |  |  |  |  |  |  |  |  |  |  |  | High |
| Kofotolis 2006 (4956)[52] |  |  |  |  |  |  |  |  |  |  |  |  |  | High |
| Kofotolis 2016 (41230)[53] |  |  |  |  |  |  |  |  |  |  |  |  |  | High |
| Lang 2021 (1942)[54] |  |  |  |  |  |  |  |  |  |  |  |  |  | High |
| Lee 2016 (40594)[55] |  |  |  |  |  |  |  |  |  |  |  |  |  | High |
| Li 2008 (5086)[56] |  |  |  |  |  |  |  |  |  |  |  |  |  | High |
| Li 2015 (45015)[57] |  |  |  |  |  |  |  |  |  |  |  |  |  | High |
| Li 2021 (1892)[58] |  |  |  |  |  |  |  |  |  |  |  |  |  | High |
| Little 2008 (14752)[59] |  |  |  |  |  |  |  |  |  |  |  |  |  | High |
| Liu 2018 (45038)[60] |  |  |  |  |  |  |  |  |  |  |  |  |  | High |
| Liu 2019 (44259)[61] |  |  |  |  |  |  |  |  |  |  |  |  |  | High |
| Lopes 2017 (41195)[62] |  |  |  |  |  |  |  |  |  |  |  |  |  | High |
| Madadi-Shad 2020 (47447)[63] |  |  |  |  |  |  |  |  |  |  |  |  |  | High |
| Magnussen 2005 (14878)[64] |  |  |  |  |  |  |  |  |  |  |  |  |  | High |
| Maul 2005 (5249)[65] |  |  |  |  |  |  |  |  |  |  |  |  |  | High |
| Mazloum 2018 (41150)[66] |  |  |  |  |  |  |  |  |  |  |  |  |  | High |
| McIlveen 1998 (52017)[67] |  |  |  |  |  |  |  |  |  |  |  |  |  | High |
| Mendes 2022 (53045)[68] |  |  |  |  |  |  |  |  |  |  |  |  |  | High |
| Moussouli 2014 (17179)[69] |  |  |  |  |  |  |  |  |  |  |  |  |  | High |
| Natour 2015 (15309)[70] |  |  |  |  |  |  |  |  |  |  |  |  |  | High |
| Noormohammadpour 2018 (43701)[71] |  |  |  |  |  |  |  |  |  |  |  |  |  | High |
| Okafor 2012 (15431)[72] |  |  |  |  |  |  |  |  |  |  |  |  |  | High |
| Park 2020 (47631)[73] |  |  |  |  |  |  |  |  |  |  |  |  |  | High |
| Patti 2016 (41038)[74] |  |  |  |  |  |  |  |  |  |  |  |  |  | High |
| Paungmali 2017 (41037)[75] |  |  |  |  |  |  |  |  |  |  |  |  |  | High |
| Phattharasupharerk 2019 (41031)[76] |  |  |  |  |  |  |  |  |  |  |  |  |  | High |
| Prado 2019 (44352)[77] |  |  |  |  |  |  |  |  |  |  |  |  |  | High |
| Puppin 2011 (17173)[78] |  |  |  |  |  |  |  |  |  |  |  |  |  | High |
| Quinn 2011 (17180)[79] |  |  |  |  |  |  |  |  |  |  |  |  |  | High |
| Raoul 2019 (46752)[80] |  |  |  |  |  |  |  |  |  |  |  |  |  | High |
| Rathi 2013 (52020)[81] |  |  |  |  |  |  |  |  |  |  |  |  |  | High |
| Raza 2020 (1601)[82] |  |  |  |  |  |  |  |  |  |  |  |  |  | High |
| Roshini 2019 (44100)[83] |  |  |  |  |  |  |  |  |  |  |  |  |  | High |
| Rydeard 2006 (9694)[84] |  |  |  |  |  |  |  |  |  |  |  |  |  | High |
| Salavati 2016 (40999)[85] |  |  |  |  |  |  |  |  |  |  |  |  |  | High |
| Sato 2021 (1738)[86] |  |  |  |  |  |  |  |  |  |  |  |  |  | High |
| Schinhan 2016 (40616)[87] |  |  |  |  |  |  |  |  |  |  |  |  |  | High |
| Sedaghati 2017 (40972)[88] |  |  |  |  |  |  |  |  |  |  |  |  |  | High |
| Segal-Snir 2016 (40150)[89] |  |  |  |  |  |  |  |  |  |  |  |  |  | High |
| Shariat 2019 (46789)[90] |  |  |  |  |  |  |  |  |  |  |  |  |  | High |
| Shu 2021 (1603)[91] |  |  |  |  |  |  |  |  |  |  |  |  |  | High |
| Sjogren 2006 (16193)[92] |  |  |  |  |  |  |  |  |  |  |  |  |  | High |
| Sokhanguei 2017 (40938)[93] |  |  |  |  |  |  |  |  |  |  |  |  |  | High |
| Sousa 2009 (5809)[94] |  |  |  |  |  |  |  |  |  |  |  |  |  | High |
| Srivastav 2018 (43827)[95] |  |  |  |  |  |  |  |  |  |  |  |  |  | High |
| Steele 2013 (16327)[96] |  |  |  |  |  |  |  |  |  |  |  |  |  | High |
| Stephan 2011 (17175)[97] |  |  |  |  |  |  |  |  |  |  |  |  |  | High |
| Suh 2019 (44414)[98] |  |  |  |  |  |  |  |  |  |  |  |  |  | High |
| Vicente-Campos 2021 (1957)[99] |  |  |  |  |  |  |  |  |  |  |  |  |  | High |
| Vincent 2014 (45025)[100] |  |  |  |  |  |  |  |  |  |  |  |  |  | High |
| Vollenbroek-Hutten 2004 (6025)[101] |  |  |  |  |  |  |  |  |  |  |  |  |  | High |
| Walsh 2020 (47828)[102] |  |  |  |  |  |  |  |  |  |  |  |  |  | High |
| Wattamwar 2012 (45028)[103] |  |  |  |  |  |  |  |  |  |  |  |  |  | High |
| Weifen 2013 (6756)[104] |  |  |  |  |  |  |  |  |  |  |  |  |  | High |
| Williams 2005 (6105)[105] |  |  |  |  |  |  |  |  |  |  |  |  |  | High |
| Williams 2009 (6104)[106] |  |  |  |  |  |  |  |  |  |  |  |  |  | High |
| Xu 2021 (1814)[107] |  |  |  |  |  |  |  |  |  |  |  |  |  | High |
| Yalfani 2022 (53132)[108] |  |  |  |  |  |  |  |  |  |  |  |  |  | High |
| Yelland 2004 (17131)[109] |  |  |  |  |  |  |  |  |  |  |  |  |  | High |
| Zadro 2019 (44491)[110] |  |  |  |  |  |  |  |  |  |  |  |  |  | High |
| Zakari 2019 (46901)[111] |  |  |  |  |  |  |  |  |  |  |  |  |  | High |

Risk of bias assessment conducted using ROB1 criteria.

First author last name and year (reference ID number).

H=high risk of bias, L=low risk of bias, ROB=risk of bias, U=Unclear

Risk of bias graph for the RCTs with an overall high risk of bias: review authors’ judgements about each risk of bias item presented as percentages across all included RCTs

**Online Resource 3.** Description of all included randomized controlled trials (RCTs) (n=13)

| **Chhabra 2018** (**43412**) | | |
| --- | --- | --- |
| Methods | | **Study design:** RCT  **Country:** India  **Exercise groups:** 1  **Comparison groups:** 1 |
| Participants | | **Number of participants:** 93 (E1: 45, C1: 48)  **Mean age, years (SD):** E1: 41.4 (14.2), C1: 41.0 (14.2)  **Age stratification (60+ years):** No  **Gender (female)**^†^**:** NR  **Chronic LBP type:** nonspecific  **Mean chronic LBP duration, months (SD):** E1: 22.8 (22), C1: 28 (25.5)  **Leg pain:** unclassified presence of leg pain  **Population source:** healthcare |
| Interventions | | **Intervention:** smartphone app (Snapcare) including home exercise (E1)  **Rationale:** aimed at motivating, promoting, and guiding the participants to increase their level of physical activity and exercise adherence  **Materials:** smartphone Snapcare app  **Procedures:** received Snapcare, along with a regular written prescription from the doctor. Patients received daily activity goals (including back and aerobic exercises), which were developed based on their health status, activities of daily living, and daily activity progress. Participants were also advised to continue with their medicines as usual. Snapcare addressed the following: 1) increase in physical activity, 2) improvement in function, 3) increase in engagement and compliance  **Exercise type(s):** aerobic, core strengthening  **Dominant exercise type:** aerobic, core strengthening  **Exercise specificity:** both whole body and back-specific  **Program design individualization:** individualized  **Format:** mixed online and in-person  **Home exercise component (yes/no):** yes  **Duration:** 12 weeks  **Location**: outpatient department at hospital [single centre]  **Provider:** physician  **Mode of delivery:** independent (home exercise) with therapist follow-up  **Tailoring:** daily activity goals and exercises were developed based on patient health status  **Modifications:** NR  **Adherence:** NR |
| Comparisons | | **Comparisons assessed:** smartphone app (Snapcare) including exercise (E1) vs. conventional therapy (C1)  **Intervention:** conventional therapy (C1)  **Procedure:** received a written prescription from the physician, containing a list of prescribed medicines and dosages, and stating the recommended level of physical activity (including home exercises)  **Materials:** NR  **Format:** in-person  **Duration:** 12 weeks  **Location:** outpatient department at hospital [single centre]  **Provider:** physician  **Mode of delivery:** independent  **Tailoring:** NR  **Modifications:** NR |
| Outcomes | | Pain (NPRS 0-10)  Function (Modified ODI 0-100)  **Follow-up (post intervention):** immediate term (closest to 2 weeks) |
| Risk of bias | | Unclear (refer to Appendix 2 for details) |
| **Costa 2009** (9861) | | |
| Methods | **Study design:** RCT  **Country:** Australia  **Exercise groups:** 1  **Comparison groups:** 1 | |
| Participants | **Number of participants:** 154 (E1: 77, C1: 77)  **Mean age, years (SD):** E1: 54.6 (13.0), C1: 52.8 (12.7)  **Age stratification (60+ years):** No  **Gender (female)**^†^**:** E1: 58%, C1: 62%  **Chronic LBP type:** nonspecific  **Mean chronic LBP duration, weeks (SD):** E1: 334.8 (392.3), C1: 328.2 (395.1)  **Leg pain:** unclassified presence of leg pain  **Population Source:** healthcare | |
| Interventions | **Intervention:** motor control exercise program (E1)  **Rationale:** stability and control of the spine are altered in people with low back pain; motor control exercises may help to restore stability and control  **Materials:** ultrasound machine (for biofeedback)  **Procedures:** participants underwent a 2-stage program; stage 1: exercises to train coordinated activity of the trunk muscles including independent activation of deeper muscles (i.e. transversus abdominis, multifidus, pelvic floor) and reduce overactivity in superficial muscles, use of ultrasound biofeedback to enhance learning; exercises progressed until participant able to maintain isolated contractions of target muscles for 10 repetitions of 10 seconds each while maintaining normal respiration; stage 2: implement precision of the desired coordination and train skills in static tasks and incorporate them into dynamic tasks and functional positions  **Exercise type(s):** motor control exercise  **Dominant exercise type:** motor control exercise  **Exercise specificity:** back-specific  **Program design individualization:** individualized  **Format:** in-person  **Home exercise component:** yes  **Duration:** 30 min, 12 sessions, 8 weeks  **Location:** outpatient physical therapy department of a university teaching hospital [single centre]  **Provider:** physical therapist  **Mode of delivery:** individual (1:1) with therapist  **Tailoring:** exercise selection was individualized based on the participant’s presentation  **Modifications:** None  **Adherence:** NR | |
| Comparisons | **Comparisons assessed:** motor control exercise program (E1) vs. placebo (C1)  **Intervention:** placebo (C1)  **Procedure:** participants received 20 minutes of detuned shortwave diathermy and 5 minutes of detune ultrasound  **Materials:** shortwave diathermy and ultrasound device  **Format:** in-person  **Duration:** 30 min, 12 sessions, 8 weeks  **Location:** outpatient physical therapy clinic of a university teaching hospital [single centre]  **Provider:** physical therapist  **Mode of delivery:** individual (1:1) with therapist  **Tailoring:** None  **Modifications:** None | |
| Outcomes | Pain (NPRS 0-10)  Function (RMDQ 0-24; Patient-Specific Functional Scale 0-10)  Adverse events (all participants asked at immediate follow-up only)  **Follow-up (post-intervention):** immediate term (closest to 2 weeks), intermediate term (closest to 6 months), long term (closest to 12 months) | |
| Risk of bias | Low (refer to Appendix 2 for details) | |
| **Fukuda 2021** (53055) | | |
| Methods | | **Study design:** RCT  **Setting:** Brazil  **Exercise groups:** 1  **Comparison groups:** 1 |
| Participants | | **Number of participants:** 70 (E1: 35, C1: 35)  **Mean age, years (SD):** E1: 40.2 (12.4), C1: 35.2 (12.5)  **Age stratification (60+ years):** No  **Gender (female)**^†^**:** 53% (not individually reported)  **Chronic LBP type:** nonspecific  **Mean chronic LBP duration, months (SD):** E1: 8.1 (8.9), C1: 6.9 (8.1)  **Leg pain:** none  **Population source:** healthcare, general population |
| Interventions | | **Intervention:** hip strengthening exercises + manual therapy and lumbar stabilization (E1)  **Rationale:** NR  **Materials:** resistance bands, ankle weights  **Procedures:** four resistance exercises to strengthen the hip (clam shells, squats and lateral stepping using elastic resistance around the thighs/knees, lateral straight leg raise using ankle weights); load standardized to 70% of 1 Repetition Maximum; 3 sets of 10 repetitions per exercise  **Exercise type(s):** core strengthening, general strength training  **Dominant exercise type:** general strength training  **Exercise specificity:** whole body + back-specific exercises  **Program design individualization:** standard  **Format (program delivery site):** in-person  **Home exercise component (yes/no):** no  **Duration:** 45 min, 10 sessions, 5 weeks  **Location:** Centro Universitário São Camilo [single centre]  **Provider:** physical therapist  **Mode of delivery:** independent (1:1) with therapist  **Tailoring:** none  **Modifications:** NR  **Adherence:** NR |
| Comparisons | | **Comparisons assessed:** hip strengthening exercises + manual therapy and lumbar stabilization (E1) vs. manual therapy and lumbar stabilization exercises (C1)  **Intervention:** manual therapy and lumbar stabilization exercises (C1)  **Procedure:** joint mobilization using Maitland method, myofascial release, segmental stabilization focused on training the recruitment of the deep stabilizer muscles (transversus abdominis, multifidus, pelvic floor muscles, diaphragm)  **Materials:** NR  **Format:** in-person  **Duration:** 30 min, 10 sessions, 5 weeks  **Location:** Centro Universitário São Camilo [single centre]  **Provider:** physical therapist  **Mode of delivery:** individual  **Tailoring:** posterior-anterior central mobilization was performed at selected segments  between L1 to L5 that were considered stiff upon manual examination  **Modifications:** NR |
| Outcomes | | Pain (VAS 0-10)  Function (RMDQ 0-24)  **Follow-up (post-intervention):** immediate term (closest to 2 weeks), moderate term (closest to 6 months), long-term (closest to 6 months) |
| Risk of bias | | Unclear (refer to Appendix 2 for details) |
| **Jinnouchi 2020** (**47336**) | | |
| Methods | | **Study design:** RCT (superiority study)  **Country:** Japan  **Exercise groups:** 1  **Comparison groups:** 1 |
| Participants | | **Number of participants:** 52 (E1: 26, C1: 26)  **Mean age, years (range):** E1: 65 (62-70), C1: 66 (64-71)  **Age stratification (60+ years):** yes  **Gender (female)**^†^**:** E1: 65.4%, C1: 61.5%  **Chronic LBP type:** nonspecific  **Chronic LBP duration, years:** E1: 1-5y: 53.8%, 5-15y: 26.9%, 15+y: 19.2%; C1: 3m-1y: 3.9%, 1-5y: 42.3%, 5-15y: 19.2%, 15y+ 34.6%  **Leg pain:** unclassified presence of leg pain  **Population source:** general population |
| Interventions | | **Intervention:** brief-see (E1)  **Rationale:** foster independent exercise skills thorough self-exercise education using materials based on the ACE concept: type I (Alignment), optimizing postural alignment; type II (Core muscles), strengthening deep muscles; and type III (Endogenous activation), activating endogenous substances in the body  **Materials:** textbook, DVD  **Procedures:** 60-100 minutes of consultation total, tailor-made self-exercise program, and individualized direct short teaching. Provided a textbook and DVD for all participants. These materials included 13 therapeutic self-exercises: standing trunk extension, standing trunk lateral flexion, prone press up into lumbar extension, seated hamstring stretches, kneeling hip flexor stretches, a seated postural exercise (scapular retraction with external rotation), quadruped opposite arm and leg raises, a single-leg bridging exercise, an abdominal drawing-in exercise, walking with good posture, aquatic exercise, cycling, and bicycle ergometer  **Exercise type(s):** aerobic, core strengthening, stretching, or flexibility/mobilizing exercises  **Dominant exercise types:** mixed exercise (3 or more types)  **Exercise specificity:** both whole body and back-specific  **Program design individualization:** individualized  **Format:** in-person  **Home exercise component (yes/no):** yes  **Duration:** 30min, number of sessions NR, 4 weeks  **Location**: NR  **Provider:** physical therapist, doctor  **Mode of delivery:** independent (home exercise) with therapist follow-up  **Tailoring:** tailor-made self-exercise program, and individualized advice regarding modifications, encouragement  **Modifications:** NR  **Adherence:** 100% attended in-person sessions; frequency of self-exercise 4 days or more per week: 73.1% at 4 weeks |
| Comparisons | | **Comparisons assessed:** brief-see (E1) vs. material-based education only (C1)  **Intervention:** material-based education only (C1)  **Procedure:** participants received the same educational materials as the brief-see group (without individiualized patient-provider interactions)  **Materials:** textbook, DVD  **Format:** home-based  **Duration:** 4 weeks  **Location:** n/a  **Provider:** n/a  **Mode of delivery:** independent  **Tailoring:** none  **Modifications:** NR |
| Outcomes | | Pain (NPRS 0-10)  Function (RMDQ 0-24)  Health-related QofL (EQ-5D 0-1)  Self-efficacy (PSEQ)  **Follow-up (post intervention):** immediate term (closest to 2 weeks), short term (closest to 3 months), moderate term (closest to 6 months) |
| Risk of bias | | Unclear (refer to Appendix 2 for details) |
| **Miyamoto 2013** (9791) | | |
| Methods | **Study design:** RCT  **Country:** Brazil  **Exercise groups:** 1  **Comparison groups:** 1 | |
| Participants | **Number of participants:** 86 (E1: 43, C1: 43)  **Mean age, years (SD):** E1: 40.7 (11.8), C1: 38.3 (11.4)  **Age stratification (60+ years):** No  **Gender (female)**^†^**:** E1: 83.7%, C1: 79.1%  **Chronic LBP type:** nonspecific  **Mean chronic LBP duration, months (SD):** E1: 73.3 (79.6), E2: 56.7 (53.5)  **Leg pain:** unclassified presence of leg pain  **Population source:** general population | |
| Interventions | **Intervention:** Pilates + educational booklet (E1)  **Rationale:** Pilates aims to improve static and dynamic stability, as well as posture and movements in general, thereby improving pain and function in patients with low back pain  **Materials:** NR  **Procedures:** participants received the same educational booklet as in C1; in addition, they received an individual, supervised treatment using the modified Pilates method; all exercises aimed at improving breathing, core stability, motor control, posture, flexibility, and mobility with the spine in neutral position; 5-10 repetitions per exercise based on patient ability  **Exercise Type(s):** Pilates  **Dominant exercise type:** Pilates  **Exercise Specificity:** back-specific  **Program design individualization:** standard  **Format:** in-person  **Home exercise component:** NR  **Duration:** 60 min, 12 sessions, 6 weeks  **Location:** outpatient physical therapy clinic [single centre]  **Provider:** physical therapist  **Mode of delivery:** Individual (1:1) with therapist  **Tailoring:** number of repetitions for each exercise individualized for each participant; exercises were tailored individually and progressed in difficulty in 3 levels (basic, intermediate, and advanced)  **Modifications:** NR  **Adherence:** 90.3% attendance at sessions; mean (SD) sessions attended: 10.8 (3.0) | |
| Comparisons | **Comparisons assessed:** Pilates + educational booklet (E1) vs. educational booklet (C1)  **Intervention:** educational booklet (C1)  **Procedure:** participants received written information about anatomy of the spine and pelvis and low back pain and recommendations regarding posture and movements involved in activities of daily living; twice weekly telephone calls for clarifications regarding the booklet  **Materials:** none  **Format:** in-person  **Dose (minutes per session):** NR  **Number of sessions:** 1  **Duration:** 6 weeks  **Location:** outpatient physical therapy clinic [single centre]  **Provider:** physical therapist  **Mode of delivery:** independent with therapist follow-up by telephone  **Tailoring:** NR  **Modifications:** NR | |
| Outcomes | Pain (NPRS 0-10)  Function (RMDQ 0-24; Patient-Specific Functional Scale 0-10)  Fear avoidance (TSK 17-68)  Adverse events (methods not reported)  **Follow-up (post-intervention):** immediate term (closest to 2 weeks), intermediate term (closest to 6 months) | |
| Risk of bias | Unclear (refer to Appendix 2 for details) | |
| **Nardin 2022** (53154) | | |
| Methods | | **Study design:** RCT  **Setting:** Brazil  **Exercise groups:** 2 (only E1 exercise group relevant to this review)  **Comparison groups:** 1 |
| Participants | | **Number of participants:** 60 (E1: 20, E2: 20, C1: 20)  **Mean age, years (SD):** E1: 42.2 (9.1), E2: 42.8 (8.4), C1: 43.1 (10.7)  **Age stratification (60+ years):** No  **Gender (female)**^†^**:** E1: 60%, E2: 75%, C1: 56.9%  **Chronic LBP type:** nonspecific  **Mean chronic LBP duration, years (SD):** NR  **Leg pain:** unclassified presence of leg pain  **Population source:** NR |
| Interventions | | **Intervention:** training (deep water running) + photobiomodulation (E1)  **Rationale:** NR  **Materials:** pool, floating vest  **Procedures:** simulating running with the use of a floating vest, which serves to keep the body in an upright position and helps prevent contact between the feet and the bottom of the pool. Training sessions were performed twice a week, on non-consecutive days, for 4 weeks and included 5 min of initial warm-up in all sessions and continuous training (30 min of moderate intensity), and interval (30 min of training that alternates 30 s of high intensity running and 30 s of walking), performed alternately for all participants  **Exercise type(s):** aerobic  **Dominant exercise type:** aerobic  **Exercise specificity:** whole body  **Program design individualization:** standard  **Format:** in-person  **Home exercise component (yes/no):** No  **Duration:** 30 min, 8 sessions, 4 weeks  **Location:** NR  **Provider:** NR  **Mode of delivery:** independent (1:1) with therapist  **Tailoring:** intensity and prescription of the training based submaximal effort test carried out prior to beginning of intervention  **Modifications:** NR  **Adherence:** NR (participants required to attend a minimum frequency of 90% of the intervention sessions) |
| Comparisons | | **Comparisons assessed:** training (deep water running) + photobiomodulation (E1) vs. photobiomodulation (C1)  **Intervention:** photobiomodulation (C1)  **Procedure:** photobiomodulation was performed through direct contact of the equipment with the irradiated site for 30 s, with an angle of 90° in relation to the skin surface. The participant received the intervention while in ventral decubitus on a stretcher, with a headset (without identifying/listening if the device was on or off) characterizing the condition of placebo (E2). All participants received the application at four points in the lumbar region (L1-L3 and L3-L5, bilateral)  **Materials:** multi-diode LED with cluster probe (THOR® DD2 control unit; THOR, London, UK)  **Format:** in-person  **Duration:** 8 sessions, 4 weeks  **Location:** NR  **Provider:** NR  **Mode of delivery:** individual  **Tailoring:** none  **Modifications:** NR |
| Outcomes | | Pain (VAS 0-10)  Function (ODI 0-100)  **Follow-up (post-intervention):** immediate term (closest to 2 weeks) |
| Risk of bias | | Unclear (refer to Appendix 2 for details) |
| **Rahbar 2018** (**43745**) | | |
| Methods | | **Study design:** RCT  **Country:** Iran  **Exercise groups:** 1  **Comparison groups:** 1 |
| Participants | | **Number of participants:** 80 (E1:40, C1:40)  **Mean age, years (SD):** E1: 46.25 (7.97), C1: 46.22 (7.83)  **Age stratification (60+ years):** No  **Gender (female)**^†^**:** E1: 32.5%, C1: 27.5%  **Chronic LBP type:** nonspecific  **Mean chronic LBP duration, months (SD):** E1: 7.05 (1.74), C1: 7.22 (1.79)  **Leg pain:** unclassified presence of leg pain  **Population source:** healthcare |
| Interventions | | **Intervention:** mechanical horseback riding (hippotherapy simulator) + standardized conventional physical therapy (E1)  **Rationale:** it provides postural and gestural exercise for subjects: these are carried out throughout rehabilitation sessions  **Materials:** AZ horseback riding machine  **Procedures:** subjects remained in sitting position for 15 minutes on the simulator during these sessions, with extension of the trunk and stabilization of the pelvis. Feet were placed on the footplates as the simulator produced a rhythmic and repetitive movement similar to a walking horse. The simulator can produce several modes of rhythmic and repetitive motions. Treatment sessions were carried out in preparatory mode assuming they would result in better transmission of motions to the user. Standardized conventional physical therapy included physical modalities (heat, ultrasound, TENS) followed by therapeutic exercises (lumbar and core stabilizing and strengthening and lower back stretching), tailored to low back pain and dysfunction  **Exercise type(s):** core strengthening, stretching, or flexibility/mobilizing exercises  **Dominant exercise type:** core strengthening  **Exercise specificity:** back-specific  **Program design individualization:** standard  **Format:** in-person  **Home exercise component (yes/no):** no  **Duration:** 15 min, 15 sessions  **Location**: outpatient physical therapy centre [single centre]  **Provider:** physiotherapist  **Mode of delivery:** independent (1:1) with therapist  **Tailoring:** NR  **Modifications:** NR  **Adherence:** 100 % |
| Comparisons | | **Comparisons assessed:** mechanical horseback riding (hippotherapy simulator) + standardized conventional physical therapy (E1) vs. standardized conventional physical therapy (C1)  **Intervention:** standardized conventional physical therapy (C1)  **Procedure:** standardized conventional physical therapy included physical modalities (heat, ultrasound, TENS) followed by therapeutic exercises (lumbar and core stabilizing and strengthening and lower back stretching), tailored to low back pain and dysfunction  **Materials:** hot pack, ultrasound, TENS unit  **Format:** in-person  **Duration:** 15 sessions  **Location:** outpatient physical therapy centre [single centre]  **Provider:** physiotherapist  **Mode of delivery:** independent  **Tailoring:** NR  **Modifications:** NR |
| Outcomes | | Pain (VAS 0-100)  Function (RMDQ 0-24)  Adverse events (methods not reported)  **Follow-up (post intervention):** immediate terms (closest to 2 weeks) |
| Risk of bias | | Unclear (refer to Appendix 2 for details) |
| **Rotter 2022** (53220) | | |
| Methods | | **Study design:** RCT  **Setting:** Germany  **Exercise groups:** 1  **Comparison groups:** 1 |
| Participants | | **Number of participants:** 55 (E1: 29, C1: 26)  **Mean age, years (SD):** E1: 52.5 (8.7), C1: 54.8 (7.5)  **Age stratification (60+ years):** No  **Gender (female)**^†^**:** E1: 82.8%, C1: 84.6%  **Chronic LBP type:** nonspecific  **Mean chronic LBP duration, years (SD):** E1: 9.6 (10.1), C1: 7.6 (10.1)  **Leg pain:** unclassified presence of leg pain  **Population source:** healthcare, general population |
| Interventions | | **Intervention:** mindful walking program (E1)  **Rationale:** mindfulness-based interventions have been reported to be effective in alleviating various bio-psychosocially influenced conditions, including pain  **Materials:** none  **Procedures:** group-based sessions (15 per group); each session built on previous sessions starting with general concept of mindfulness awareness and adding instructions on the technique of mindful walking (conscious rolling of the feet, swinging of the arms, conscious torso posture); sessions incorporated 10 minutes of warm-up stretching, 15-25 minutes of group walking instruction, 10 minutes of individual mindful walking, 5 minutes of stretching and 5 minutes of feedback; participants were instructed to self-exercise between group sessions  **Exercise type(s):** aerobic, stretching, or flexibility/mobilizing exercises  **Dominant exercise type:** aerobic  **Exercise specificity:** whole body  **Program design individualization:** standard  **Format:** in-person  **Home exercise component (yes/no):** yes  **Duration:** 60 min, 8 sessions, 8 weeks  **Location:** outpatient clinic [single centre]  **Provider:** physiotherapist, physician, sports therapist  **Mode of delivery:** group  **Tailoring:** participants were instructed to self-exercise between group sessions. Patients were allowed and instructed to use rescue medication on demand (paracetamol; maximum dosage, four times 500 mg/day)  **Modifications:** NR  **Adherence:** NR |
| Comparisons | | **Comparisons assessed:** mindful walking program (E1) vs. no treatment (C1)  **Intervention:** no treatment (C1)  **Procedure:** n/a  **Materials:** n/a  **Format:** n/a  **Duration:** n/a  **Location:** n/a  **Provider:** n/a  **Mode of delivery:** n/a  **Tailoring:** patients were allowed and instructed to use rescue medication on demand (paracetamol; maximum dosage, four times 500 mg/day)  **Modifications:** n/a |
| Outcomes | | Pain (VAS 0-100)  Function (Hannover Functional Ability Questionnaire 0-100)  Health-related QofL (SF-36 0-100)  Adverse events (measured across the whole study period)  **Follow-up (post-intervention):** immediate term (closest to 2 weeks), short term (closest to 3 months) |
| Risk of bias | | Unclear (refer to Appendix 2 for details) |
| **Shamsi 2022** (53159) | | |
| Methods | | **Study design:** RCT  **Setting:** Iran  **Exercise groups:** 2  **Comparison groups:** 1 |
| Participants | | **Number of participants:** 45 (E1: 15, E2: 15, C1: 15)  **Mean age, years (SD):** E1: 37.67 (8.96), E2: 37.07 (13.39), C1: 39.12 (11.61)  **Age stratification (60+ years):** No  **Gender (female)**^†^**:** E1: 33.3%, E2: 26.7%, C1: 33.3%  **Chronic LBP type:** nonspecific  **Mean chronic LBP duration, years (SD):** NR  **Leg pain:** none  **Population source:** healthcare |
| Interventions | | **Intervention:** hamstring static stretching + physiotherapy (E1)  **Rationale:** stretching of passive ligaments appropriately makes the paraspinal muscles relax due to a stretch inhibition reflex. This reflex may be the cause of flexion-relaxation ratio phenomenon and therefore instead of active muscles, the spinal stability is the product of passive structures  **Materials:** spring, sling, supports  **Procedures:** the hamstring was stretched by passively flexing the hip with knee fully extended while lying in supine position applying traction to ankle by a spring. Three sets of 2 min of stretch with 2 min rest in between were applied. Physiotherapy treatment included 15 min of heat therapy (hot pack), 15 min application of transcutaneous electrical stimulation (TENS) to low back area and the commonly used general exercises for back pain including paraspinal and abdominal muscle strengthening and stretching exercises  **Exercise type(s):** core strengthening, stretching, or flexibility/mobilizing exercises  **Dominant exercise type:** stretching, or flexibility/mobilizing exercises  **Exercise specificity:** hamstring flexibility  **Program design individualization:** standard  **Format:** in-person  **Home exercise component (yes/no):** NR  **Duration:** 12 sessions, 4 weeks  **Location:** physiotherapy clinic [single centre]  **Provider:** physiotherapist  **Mode of delivery:** independent (1:1) with therapist  **Tailoring:** NR  **Modifications:** NR  **Adherence:** NR (three consecutive or five intermittent absences in attending sessions, they were excluded)  **Intervention:** hamstring strengthening in lengthened position + physiotherapy (E2)  **Rationale:** stretching of passive ligaments appropriately makes the paraspinal muscles relax due to a stretch inhibition reflex. This reflex may be the cause of flexion-relaxation ratio phenomenon and therefore instead of active muscles, the spinal stability is the product of passive structures  **Materials:** spring, sling, supports  **Procedures:** the hamstring was contracted concentrically in its lengthened position. To attain this position, patients were seated on a chair with the thigh resting on a support keeping hip joint in 120 degrees of flexion and knee in full extension. A spring was attached to their ankle flexing hip joint and the patients contracted hamstring muscle against the spring by extending the hip. The other hip and knee joint remained flexed in 90 degrees. Physiotherapy treatment included 15 min of heat therapy (hot pack), 15 min application of transcutaneous electrical stimulation (TENS) to low back area and the commonly used general exercises for back pain including paraspinal and abdominal muscle strengthening and stretching exercises  **Exercise type(s):** core strengthening, general strength training, stretching, or flexibility/mobilizing exercises  **Dominant exercise type:** general strength training  **Exercise specificity:** hamstring strength  **Program design individualization:** standard  **Format:** in-person  **Home exercise component (yes/no):** NR  **Duration:** 12 sessions, 4 weeks  **Location:** physiotherapy clinic [single centre]  **Provider:** physiotherapist  **Mode of delivery:** individual  **Tailoring:** NR  **Modifications:** NR  **Adherence:** NR (three consecutive or five intermittent absences in attending sessions, they were excluded) |
| Comparisons | | **Comparisons assessed:**   1. hamstring static stretching + physiotherapy (E1) vs. physiotherapy (C1) 2. hamstring strengthening in lengthened position + physiotherapy (E2) vs. physiotherapy (C1)   **Intervention:** physiotherapy (C1)  **Procedure:** physiotherapy treatment included 15 min of heat therapy (hot pack), 15 min application of transcutaneous electrical stimulation (TENS) to low back area and the commonly used general exercises for back pain including paraspinal and abdominal muscle strengthening and stretching exercises  **Materials:** hot pack, TENS unit  **Format:** in-person  **Duration:** 12 sessions, 4 weeks  **Location:** physiotherapy clinic  **Provider:** physiotherapist  **Mode of delivery:** individual (1:1) with therapist  **Tailoring:** NR  **Modifications:** NR |
| Outcomes | | Pain (VAS 0-100)  Function (ODI 0-100)  **Follow-up (post-intervention):** immediate term (closest to 2 weeks) |
| Risk of bias | | Unclear (refer to Appendix 2 for details) |
| **Smeets 2008** (16226) | | |
| Methods | | **Study design:** RCT (cluster randomized trial)  **Setting:** Netherlands  **Exercise groups:** 1  **Comparison groups:** 1 |
| Participants | | **Number of participants:** 223 (E1: 61, C1: 58, C2: 53, C3: 51) *only E1 and C1 relevant for review  **Mean age, years (SD):** E1: 40.7 (10.1), C1: 42.5 (9.7)  **Age stratification (60+ years):** No  **Gender (female)**^†^**:** E1: 37.7%, C1: 58.6%  **Chronic LBP type:** nonspecific  **Mean chronic LBP duration, months (SD):** E1: 56.1 (67.5), C1: 68.3 (74.2)  **Leg pain:** mixed with and without leg pain (non-radicular)  **Population source:** healthcare |
| Interventions | | **Intervention:** active physical treatment + graded activity with problem solving training (E1)  **Rationale:** increased aerobic capacity and muscle reconditioning, especially of the deep lumbar extensor muscles (multifidus muscle), are needed for better functioning  **Materials:** bicycle  **Procedures:** active physical treatment consisted of 30 min of aerobic training on a bicycle (65–80% heart rate maximum) and 75 min of strength and endurance training of their lower back and upper leg muscles (three series of 15–18 repetitions in a dynamic–static manner with a training intensity of 70% of the 1-Repetition Maximum), three times a week over 10 weeks. Graded activity and problem-solving training provided in identical manner to C1 and started in the 3rd week of training  **Exercise type(s):** aerobic, core strengthening  **Dominant exercise type:** aerobic, core strengthening (mixed)  **Exercise specificity:** both whole body and back-specific  **Program design individualization:** standard  **Format:** in-person  **Home exercise component (yes/no):** no  **Duration:** 105 minutes/session, 30 sessions, 10 weeks  **Location:** NR  **Provider:** physiotherapist  **Mode of delivery:** group  **Tailoring:** training intensity tailored to each participant of 70% of their 1-repetition maximum  **Modifications:** NR  **Adherence:** 72% |
| Comparisons | | **Comparisons assessed:**   1. active physical treatment + graded activity with problem solving training (E1) vs. graded activity with problem solving training combination treatment (C1)   **Intervention:** graded activity with problem solving training (C1)  **Procedures:** started with active physical treatment and problem-solving training, both offered in the same frequency and duration as in active physical treatment and graded activity with problem solving training combination treatment, respectively. Graded activity started in the 3rd week with the selection of three patient specific activities. By the end of the 4th week, the gradual increase of these three activities was started. The active physical treatment sessions were given three times a week, problem solving treatment once a week, and graded activity initially three times a week, and gradually decreasing to once a week. The patients did not have to come to the treatment centre more often than three times a week, meaning that once a week, a patient received all interventions, all at one day  **Materials:** NR  **Format:** in-person  **Duration:** graded activity: 30 minutes/session, maximum 20 sessions, 10 weeks; problem solving training: 90 minutes/session, 10 sessions, 10 weeks  **Location:** NR  **Provider:** physiotherapist or occupational therapist and clinical psychologist or social worker  **Mode of delivery:** group  **Tailoring:** selection of three activities relevant to each participants’ personal situation  **Modifications:** NR |
| Outcomes | | Pain (VAS 0-100)  Functional limitations (RMDQ 0-24)  Depression (BDI 0-63)  Adverse events (recorded when reported by participants)  **Follow-up:** immediate term (closest to 2 weeks), moderate term (closest to 6 months), long term (closest to 12 months) |
| Risk of bias | | Low (refer to Appendix 2 for details) |
| **Takinaci 2019** (44427) | | |
| Methods | **Study design:** RCT  **Country:** Turkey  **Exercise groups:** 1  **Comparison groups:** 1 | |
| Participants | **Number of participants:** 60 (E1: 30, C1: 30)  **Mean age, years (SD):** E1: 64.5 (11.9), C1: 60.33 (9.6)  **Age stratification (60+ years):** No  **Gender (female)**^†^**:** E1: 69.2%, C1: 75%  **Chronic LBP type:** nonspecific  **Mean chronic LBP duration, years (SD):** NR  **Leg pain:** unclassified presence of leg pain  **Population** **source:** healthcare | |
| Interventions | **Intervention:** exercise + balneotherapy (E1)  **Rationale:** flexion-based exercises reduce compressive forces and stretch lumbar posterior ligamentous and myofascial tissues; stretching the hip flexors and strengthening the gluteal and abdominal muscles protects the spine from supporting overloads  **Materials:** NR  **Procedures:** participants received same balneotherapy intervention as C1 and participated in once daily supervised group exercise consisting of lumbar flexion, pelvic mobilization, strengthening of the gluteal, abdominal and knee extensor muscles, and stretching of the hip and knee flexor muscles (15 repetitions per exercise)  **Exercise type(s):** general strength training, stretching, or flexibility/mobilizing exercises  **Dominant Exercise Type:** mixed exercise (3 or more types)  **Exercise Specificity:** whole body  **Program design individualization:** standard  **Format:** in person  **Home exercise component (yes/no):** NR  **Duration:** 12 sessions, 2 weeks  **Location:** spa hotel [single centre]  **Provider:** physiotherapist  **Mode of delivery:** group  **Tailoring:** NR  **Modifications:** NR  **Adherence:** NR | |
| Comparisons | **Comparisons assessed:** exercise + balneotherapy (E1) vs. balneotherapy (C1)  **Intervention:** balneotherapy (C1)  **Procedure:** participants underwent two immersions per day (one in a bath filled with thermo-mineral water at 36 to 37 °C and one at 38 °C); each session followed by 20 minutes rest  **Materials:** immersion baths  **Format:** in-person  **Duration:** 20 min, 24 sessions (twice daily), 2 weeks  **Location:** spa hotel [single centre]  **Provider:** physiotherapist  **Mode of delivery:** NR  **Tailoring:** NR  **Modifications:** NR | |
| Outcomes | Pain (VAS 0-100)  Function (WI 0-9)  Health-related QofL (Health Assessment Questionnaire 0-3)  **Follow-up (post-intervention):** immediate term (closest to 2 weeks), short-term (closest to 3 months), intermediate term (closest to 6 months) | |
| Risk of bias | Unclear (refer to Appendix 2 for details) | |
| **Tilbrook 2011** (17167) | | |
| Methods | | **Study design:** RCT  **Setting:** United Kingdom  **Exercise groups:** 1  **Comparison groups:** 1 |
| Participants | | **Number of participants:** 313 (E1: 156, C1: 157)  **Mean age, years (SD):** E1: 46.4 (11.3), C1: 46.3 (11.5)  **Age stratification (60+ years):** No  **Gender (female)**^†^**:** E1: 68%, C1: 73%  **Chronic LBP type:** nonspecific  **Mean chronic LBP duration, months (SD):** E1: 130.3 (117), C1: 113.5 (115.3)  **Leg pain:** unclassified presence of leg pain  **Population source:** healthcare |
| Interventions | | **Intervention:** Iyengar yoga + back book + usual care (E1)  **Rationale:** the benefits of yoga may be greater than those of exercise alone because yoga offers a combination of physical exercise with mental focus, and patients are taught good posture, self-awareness, and self-care along with relaxation  **Materials:** mat, manual, relaxation CD, home practice sheets  **Procedures:** the yoga program introduced participants to the foundational elements of yoga adapted appropriately for low back pain, including asana, pranayama, relaxation techniques, mental focus, and philosophy  **Exercise type(s):** yoga  **Dominant exercise type:** yoga  **Exercise specificity:** whole body  **Program design individualization:** standard  **Format:** in-person  **Home exercise component:** yes  **Duration:** 75 minutes/session, 12 sessions, 12 weeks  **Location:** non-medical centres [multicentre]; community-based  **Provider:** exercise specialist (yoga teacher) experienced and trained specifically for the trial  **Mode of delivery:** group  **Tailoring:** none  **Modifications:** NR  **Adherence:** 60% (attended at least 3 of the first 6 classes and at least any other 3 classes (adhered); of the remaining participants, 40 (26%) attended at least 1 class but did not meet the above criteria) |
| Comparisons | | **Comparisons assessed:** Iyengar yoga (E1) vs. usual care + back book (C1)  **Intervention:** usual care + back book (C1)  **Procedure:** received usual care and back book education  **Materials:** back book  **Format:** n/a  **Duration:** 12 weeks  **Location:** n/a  **Exercise specificity:** n/a  **Home exercise component:** n/a  **Provider:** NR  **Mode of delivery:** independent  **Tailoring:** NR  **Modifications:** NR |
| Outcomes | | Pain (Aberdeen Back Pain Scale 0-100)  Function (RMDQ 0-24)  Health-related QofL (SF-12 0-100)  Self-efficacy (PSEQ 0-60)  Adverse events (recorded when reported by participants)  **Follow-up:** immediate term (closest to 2 weeks), moderate term (closest to 6 months), long term (closest to 12 months) |
| Risk of bias | | Unclear (refer to Appendix 2 for details) |
| **Weiner 2008** (6068) | | |
| Methods | **Study design:** RCT  **Country:** United States  **Exercise groups:** 2  **Comparison groups:** 2 | |
| Participants | **Number of participants:** 200 (E1: 50, E2: 50, C1: 50, C2: 50)  **Mean age, years (SD):** E1: 73.9 (5.2), E2: 73.3 (6.0), C1: 74.3 (6.4); C2: 74.1 (5.6)  **Age stratification (60+ years):** yes  **Gender (female)**^†^**:** E1: 56%, E2: 60%, C1: 54%, C2: 58%  **Chronic LBP type:** NR (assumed to be nonspecific primary)  **Mean chronic LBP duration, years:** E1: 9.0, E2: 5.0, C1: 10.0, C2: 9.0  **Leg pain:** mixed with and without leg pain (unclassified radicular vs. non-radicular); E1: 10.0%, E2: 10.0%, C1: 12.0%, C2: 4.0%  **Population source:** healthcare | |
| Interventions | **Intervention:** general conditioning and aerobic exercise + PENS (E1)  **Rationale:** patients with chronic low back pain become deconditioned due to inactivity; general conditioning and aerobic exercise can aid in reconditioning, thereby helping to reduce pain and improve function  **Materials:** treadmill or stationary bicycle  **Procedures:** participants performed on-site exercises consisting of: 1) general conditioning (strength and flexibility exercises, each performed for 2 minutes with 1-minute rest) and 2) aerobic exercise (maximum 30 minutes of treadmill or stationary bicycle); in addition, a home exercise program consisted of: 1) flexibility exercises (12 stretches targeting lower extremity and low back musculature; 3 repetitions per exercise, 3 times per day); and 2) graded walking program (maximum 30 minutes per day, 3 times per week); PENS delivered in an identical manner to C1  **Exercise type(s):** aerobic, general strength training, stretching or flexibility/mobilizing exercises  **Dominant exercise type:** mixed exercise (3 or more types)  **Exercise specificity:** whole body  **Program design individualization:** standard  **Format:** in-person  **Home exercise component (yes/no):** yes (stretches 3x daily, graded walking program 3 days per week for 6 weeks)  **Duration:** 12 sessions, 6 weeks  **Location:** outpatient pain management program [single centre]  **Provider:** physical therapist  **Mode of delivery:** individual (1:1) with therapist  **Tailoring:** intensity and volume for general conditioning and aerobic exercise tailored to the participant based on baseline evaluation; type of aerobic exercise (treadmill vs. bicycle) based on participant preference  **Modifications:** NR  **Adherence:** NR  **Intervention:** general conditioning and aerobic exercise + sham PENS (E2)  **Rationale:** patients with chronic low back pain become deconditioned due to inactivity; general conditioning and aerobic exercise can aid in reconditioning, thereby helping to reduce pain and improve function  **Materials:** treadmill or stationary bicycle  **Procedures:** exercise program delivered in an identical manner to E1; sham PENS delivered in an identical manner to C2  **Exercise type(s):** aerobic, general strength training, stretching, or flexibility/mobilizing exercises  **Dominant exercise type:** mixed exercise (3 or more types)  **Exercise specificity:** whole body  **Program design individualization:** standard  **Format:** in-person  **Home exercise component (yes/no):** yes (stretches 3x daily, graded walking program 3 days per week for 6 weeks)  **Duration:** 60 min, 12 sessions, 6 weeks  **Location:** outpatient pain management program [single centre]  **Provider:** physical therapist  **Mode of delivery:** individual (1:1) with therapist  **Tailoring:** intensity and volume for general conditioning and aerobic exercise tailored to the participant based on baseline evaluation; type of aerobic exercise (treadmill vs. bicycle) based on participant preference  **Modifications:** NR  **Adherence:** NR | |
| Comparisons | **Comparisons assessed:**   1. general conditioning and aerobic exercise + PENS (E1) vs. PENS (C1) 2. general conditioning and aerobic exercise + Sham PENS (E2) vs. Sham (C2)   **Intervention:** PENS (C1)  **Procedure:** 32-gauge 40 mm needles placed at depth of 15 mm; ten needles per session, placed bilaterally at dermatomal, myotomal, scleratomal, and sympathetic levels corresponding to T-12, L3, L5, and S2, and the motor point for the piriformis muscle; electrical stimulation was applied for 30 min, using a specific pattern (alternating positive and negative leads) using Pantheon Research PENS electrostimulator; frequency used is determined by the response to the previous treatment session; amplitude set to create a perceived stimulus of moderate intensity and was adjusted throughout the session to maintain constant stimulus perceptibility; in addition, two needles were placed bilaterally at the T-12 dermatome with electrical stimulation applied in an identical manner to C2.  **Materials:** 32-guage 40 mm needles; PENS electrostimulator  **Format:** in-person  **Duration:** 30 min, 12 sessions, 6 weeks  **Location:** outpatient pain management program  **Provider:** physical therapist  **Mode of delivery:** individual (1:1) with therapist  **Tailoring:** frequency tailored based on response to treatment; amplitude set to patient tolerance to achieve a moderate intensity stimulus  **Modifications:** NR  **Intervention:** sham PENS (C2)  **Procedure:** ten needles placed in identical manner to C1 without electrical stimulation, two needles were placed bilaterally at the T-12 dermatome with electrical stimulation applied using Pantheon Research PENS electrostimulator at frequency of 100 Hz for 5 minutes then turned off for the remainder of the session.  **Materials:** 32-guage 40 mm needles; PENS electrostimulator  **Format:** in-person  **Duration:** 30 min, 12 sessions, 6 weeks  **Location:** outpatient pain management program  **Provider:** physical therapist  **Mode of delivery:** individual (1:1) with therapist  **Tailoring:** none  **Modifications:** NR | |
| Outcomes | Pain (MPQ 0-45)  Function (RMDQ 0-24)  Health-related QofL (SF-36 0-100)  Depression (Geriatric Depression Scale 0-30)  Self-efficacy (Chronic Pain Self-Efficacy Scale 10-100)  Catastrophizing (Coping Strategies Questionnaire – Catastrophizing 0-6)  Fear avoidance (FABQ-PA 0-24)  Usual pace gait speed (25 meters; m/s)  Chair raise time (5 consecutive raises; seconds)  Stair climb time (seconds)  Adverse events (methods not reported)  **Follow-up (post-intervention):** immediate term (closest to 2 weeks), intermediate term (closest to 6 months) | |
| Risk of bias | Unclear (refer to Appendix 2 for details) | |

First author last name and year (reference ID)

^†^We used the terms ‘female or male’ to describe gender because these were the terms used by study authors; however, we recognize that gender is a social construct and sex is a biological construct.2013

**C1:** Comparison treatment group 1, **E1:** Exercise treatment group 1, **BDI:** Beck Depression Inventory, **FABQ-PA:** Fear Avoidance Beliefs Questionnaire Physical Activities subscale, **LBP:** low back pain, **NPRS:** numeric pain rating scale, **n/a:** not applicable; **NR:** not reported; **ODI:** Oswestry Disability Index, **PENS:** Percutaneous electrical nerve stimulation, **PSEQ:** Pain Self-Efficacy Questionnaire, **QofL:** Quality of life, **RCT:** randomized controlled trial, **RMDQ:** Roland-Morris Disability Questionnaire, **SD:** standard deviation, **SF-12:** 12-item Short Form Health Survey, **SF-36:** 36-item Short Form Health Survey, **TENS:** Transcutaneous Electrical Nerve Stimulation, **TSK:** Tampa Scale of Kinesiophobia, **VAS:** Visual Analogue Scale, **WI:** Waddell Disability Index

**Online Resource 4.** List of excluded studies with reasons for exclusion (n=341)

| Reason | Study |
| --- | --- |
| Ineligible publication type  n = 22 | 1. Akodu et al. (Ref ID 53140). Effects of core stabilization exercise and cognitive behavioural therapy in the management of patients with non-specific chronic low back pain. 2021. 2. Antunes et al. (Ref ID 1777). Comparison between the intervention with back school and postural reeducation in the posture of elderly people with low back pain. 2021. 3. Bellido-Fernandez et al. (Ref ID 1645). Corrigendum to "Effectiveness of Massage Therapy and Abdominal Hypopressive Gymnastics in Nonspecific Chronic Low Back Pain: a Randomized Controlled Pilot Study". 2018. 4. Bendix et al. (Ref ID 1781). Intensive multidisciplinary treatment of back pain--2 controlled prospective studies. 1994. 5. Bussing et al. (Ref ID 1828). Randomized clinical trial to treat patients with chronic back pain: a comparison of the efficacy of yoga, eurythmy therapy and standard physiotherapy. 2017. 6. Chan et al. (Ref ID 1789). 12 month results of a randomised controlled trial comparing subgroup specific physiotherapy against advice for people with low back disorders. 2013. 7. Dalichau et al. (Ref ID 1496). Significance of aquatic functional training in the therapy of chronic low back pain. 2015. 8. Ergezen et al. (CRSID – 19355010) (Ref ID 60001). Effect of stretching exercises in individuals with piriformis syndrome. 9. Ferri-Caruana et al. (Ref ID 53062). Effect of a Pilates exercise program on the flexion-relaxation rate in women with chronic low back pain. 2022. 10. Groessl et al. (Ref ID 1876). Yoga for chronic low back and neck pain in military personnel. 2020. 11. Hurley et al. (Ref ID 1658). The supervised walking in comparison to fitness training for back pain trial: 3 and 6 month clinical outcomes. 2011 12. Lukens et al. (Ref ID 1847). Training of the transverse abdominal muscle using a sling therapy system in comparison to an established technique for CLBP. 2014. 13. McDonough et al. (Ref ID 1498). A pedometer-based walking programme for people with chronic low back pain: Experience from the back 2 activity trial. 2011. 14. Ogunniran et al. (Ref ID 53147). Effects of kinesiology taping and core stabilization exercise on pain, disability, psychological status and sleep disturbance in patients with non-specific chronic low back pain. 2022. 15. Ozcan et al. (Ref ID 1794). The effectiveness of a participatory ergonomic intervention for preventing work-related musculoskeletal disorders in nurses. 2013. 16. Patel et al. (Ref ID 1799). The effects of footwear generated biomechanical manipulation of gait on pain and function in a chronic nonspecific low back pain population. 2019. 17. Sheeran et al. (Ref ID 1147). The effect of classification-based cognitive functional therapy on spinal kinematics and function in subgroups of chronic low back pain. 2016. 18. Toy et al. (Ref ID 1750). Comparison of the effectiveness of physical therapy, exercise and kinesio-taping in patients with chronic low back pain: preliminary results. 2013. 19. Vincent et al. (Ref ID 1899). Low back strength gain contributes to walking improvement in obese older adults with chronic low back pain. 2013. 20. Wittig et al. (Ref ID 1759). Efficacy of physiotherapy and ultra-sound in patients with chronic low back pain. 2011. 21. Wu et al. (Ref ID 52025). The clinical research of chronic non-specific low back pain which based on the theory of muscle imbalance. 2016.* 22. Zaworski et al. (Ref ID 1922). The effectiveness of manual therapy and proprioceptive neuromuscular facilitation (PNF) compared with traditional kinesiotherapy in the treatment of non-specific low back pain. 2018 |
| Ineligible study design  n = 31 | 1. Added et al. Strengthening of the gluteus maximus in subjects with sacroiliac dysfunction. 2018.* 2. Alshami et al. (Ref ID 53092). Effect of Neural Mobilization Exercises in Patients With Low Back-Related Leg Pain With Peripheral Nerve Sensitization: a Prospective, Controlled Trial. 2021. 3. Ben-Ami et al. (Ref ID 1644). Outcomes in Distressed Patients with Chronic Low Back Pain: subgroup Analysis of a Clinical Trial. 2018. 4. Canaway et al. (Ref ID 43406). Is an enhanced behaviour change intervention cost-effective compared with physiotherapy for patients with chronic low back pain? Results from a multicentre trial in Israel. 2018. 5. Chen et al. (Ref ID 46108). Core stability training improves back muscle endurance and increases peak values of isokinetic strength of back muscles. 2018. 6. Chen et al. (Ref ID 52001). Effects of horse riding simulator on pain, oswestry disability index and balance in adults with nonspecific chronic low back pain. 2016.* 7. Engkvist et al. (Ref ID 1252). Evaluation of an intervention comprising a No Lifting Policy in Australian hospitals. 2006. 8. Ghasemi et al. (Ref ID 52006). Comparative study of muscle energy technique, craniosacral therapy, and sensorimotor training effects on postural control in patients with nonspecific chronic low back pain. 2020.* 9. Gohil et al. (Ref ID 43502). The efficacy of a lumbar strengthening program in lumbar spine derangement syndrome 1. 2018. 10. Hemmati et al. (Ref ID 1839). Effects of consecutive supervised core stability training on pain and disability in women with nonspecific chronic low back pain. 2011. 11. Hergenroeder et al. (Ref ID 53061). Effect of a 6-month sedentary behavior reduction intervention on well-being and workplace health in desk workers with low back pain. 2022. 12. Hochheim et al. (Ref ID 53050). Cost-effectiveness analysis of a chronic back pain multidisciplinary biopsychosocial rehabilitation (MBR) compared to standard care for privately insured in Germany. 2021. 13. Hooker et al. (Ref ID 53223). Motor skill training versus strength and flexibility exercise in people with chronic low back pain: preplanned analysis of effects on kinematics during a functional activity. 2022. 14. Huge et al. (Ref ID 1881). Impact of a functional restoration program on pain and health-related quality of life in patients with chronic low back pain. 2006. 15. Jeong et al. (Ref ID 52010). The effects of sling bridging exercise to pain scale and trunk muscle activity in low back pain patients. 2013.* 16. Kaur et al. (Ref ID 52011). Effectiveness of trunk balance exercises and wii fit TM balance exercises in managing disability and pain in patients with chronic low back pain. 2019.* 17. Leonard et al. (Ref ID 1891). Changes in Transversus Abdominis Muscle Thickness after Lumbo-Pelvic Core Stabilization Training among Chronic Low Back Pain Individuals. 2015. 18. Marshall et al. (Ref ID 1584). Changes in Pain Self-Efficacy, Coping Skills and Fear Avoidance Beliefs in a Randomized Controlled Trial of Yoga, Physical Therapy, and Education for Chronic Low Back Pain. 2021. 19. Nielsen et al. (Ref ID 1526). Group acupuncture therapy with yoga therapy for chronic pain in underserved population: feasibility pilot study. 2020. 20. Oratsch et al. (Ref ID 46706). Treatment for chronic back pain? : Active multimodal, interdisciplinary pain therapy vs. physiotherapy-physical therapy for chronic back pain. 2019. 21. Pacrez-de-la-Cruz et al. (Ref ID 40723). Effectiveness of a Program of Romana's Pilates for Non-Specific Low Back Pain. A Pilot Study. 2017. 22. Pahilaj et al. (Ref ID 1731). To Study the Combined Effect of Lumbar and Stabilization Exercises and Ift in Subjects with Chronic Low Back Pain- An Experimental Study. 2020 23. Schroder et al. (Ref ID 53205). Improved adherence to clinical guidelines for low back pain after implementation of the BetterBack model of care: a stepped cluster randomized controlled trial within a hybrid type 2 trial. 2022. 24. Sitges et al. (Ref ID 1807). Acute Effects of a Brief Physical Exercise Intervention on Somatosensory Perception, Lumbar Strength, and Flexibility in Patients with Nonspecific Chronic Low-Back Pain. 2021. 25. Surkit et al. (Ref ID 40098). Effects of individualised directional preference management versus advice for reducible discogenic pain: A pre-planned secondary analysis of a randomised controlled trial. 2016. 26. Tanovic et al. (Ref ID 815). Intermittent traction therapy in the treatment of chronic low back pain. 2021. 27. Turner et al. (Ref ID 1897). Comparison of group progressive-relaxation training and cognitive-behavioral group therapy for chronic low back pain. 1982. 28. Vlaeyen et al. (Ref ID 401). The treatment of fear of movement/(re)injury in chronic low back pain: further evidence on the effectiveness of exposure in vivo. 2002. 29. Xu et al. (Ref ID 53278). Whole body physical training in the treatment of chronic low back pain. 2021 30. Yoo et al. (Ref ID 52027). The effect of horse simulator riding on visual analogue scale, body composition and trunk strength in the patients with chronic low back pain. 2014* 31. (Ref ID 1818). Effects of Mckenzie method of mechanical diagnosis and therapy on lumbar rom & pain in patients with non-specific low back pain. 2021. |
| Ineligible population  n = 82 | 1. Abasolo et al. (Ref ID 948). Musculoskeletal work disability for clinicians: time course and effectiveness of a specialized intervention program by diagnosis. 2007. 2. Alzahrani et al. (Ref ID 1823). Wearables-based walking program in addition to usual physiotherapy care for the management of patients with low back pain at medium or high risk of chronicity: a pilot randomized controlled trial. 2021. 3. Anan et al. (Ref ID 1825). Effects of an Artificial Intelligence-Assisted Health Program on Workers With Neck/Shoulder Pain/Stiffness and Low Back Pain: randomized Controlled Trial. 2021. 4. Barbosa Silva et al. (Ref ID 46053). Comparação do método Pilates com e sem a estabilização segmentar na dor lombar crónica. 2013. 5. Carvalho et al. Effectiveness of additional deep-water running for disability, lumbar pain intensity, and functional capacity in patients with chronic low back pain: a randomised controlled trial with 3-month follow-up. 2020.* 6. Cassidy et al. (Ref ID 1787). A blinded randomized clinical trial of manual therapy and physiotherapy for chronic back and neck complaints: physical outcome measures. 1992. 7. Cavalcanti et al. (Ref ID 47062). Global Postural Re-education in non-specific neck and low back pain treatment: A pilot study. 2020 8. Cherkin et al. (Ref ID 52002). A comparison of physical therapy, chiropractic manipulation, and provision of an educational booklet for the treatment of patients with low back pain. 1998.* 9. Cimarras-Otal et al. (Ref ID 47081). Adapted exercises versus general exercise recommendations on chronic low back pain in industrial workers: A randomized control pilot study. 2020. 10. Damush et al. (Ref ID 1528). The long-term effects of a self-management program for inner-city primary care patients with acute low back pain. 2003. 11. de Oliveira et al. (Ref ID 46047). Comparing the hydrokinesiotherapy effects with land exercises in non specific low back pain patients: randomized clinical Trial. 2011 12. Descarreaux et al. (Ref ID 52003). Evaluation of a specific home exercise program for low back pain. 2002.* 13. Ebrall et al. (Ref ID 1646). A blinded randomized clinical trial of manual therapy and physiotherapy for chronic back and neck complaints physical outcome measures. 1992. 14. Fazel et al. (Ref ID 53080). The Effect of Exercise Programs on Pain Management and Motor Control in Patients with Nonspecific Chronic Low Back Pain: a Randomized Matched Subjects Trial. 2021. 15. Ferreira et al. (Ref ID 1836). Exercise Is Medicine, But Perhaps Not for Preventing Low Back Pain: a Randomized Trial of Exercise and Education to Prevent Low Back Pain Recurrence. 2021. 16. Filipczyk et al. (Ref ID 53211). Influence of stabilization techniques used in the treatment of low back pain on the level of kinesiophobia. 2021. 17. Franca et al. (Ref ID 44363). Motor Control Training Compared With Transcutaneous Electrical Nerve Stimulation in Patients With Disc Herniation With Associated Radiculopathy: A Randomized Controlled Trial. 2019. 18. Greenfield et al. (Ref ID 265). Nurse-protocol management of low back pain. Outcomes, patient satisfaction and efficiency of primary care. 1975. 19. Hansen et al. (Ref ID 1626). Intensive, dynamic back-muscle exercises, conventional physiotherapy, or placebo-control treatment of low-back pain: a randomized, observer-blind trial. 1993. 20. Harper et al. (Ref ID 44164). Fascial manipulation vs. standard physical therapy practice for low back pain diagnoses: A pragmatic study. 2019. 21. Hill et al. (Ref ID 52008). Comparison of stratified primary care management for low back pain with current best practice (STarT Back): a randomised controlled trial. 2011.* 22. Hui et al. (Ref ID 1054). A Rehabilitation Programme Focussing on Pelvic Floor Muscle Training for Persistent Lumbopelvic Pain after Childbirth: a randomized controlled trial. 2021 23. Ikram et al. (Ref ID 53097). Effect of postero-anterior lumber spine mobilization versus mckenzie press-ups in non-specific low back pain. 2021. 24. Jahantiqh et al. (Ref ID 43552). Effects of Reiki Versus Physiotherapy on Relieving Lower Back Pain and Improving Activities Daily Living of Patients With Intervertebral Disc Hernia. 2018. 25. Javadov et al. (Ref ID 53174). The Efficiency of Manual Therapy and Sacroiliac and Lumbar Exercises in Patients with Sacroiliac Joint Dysfunction Syndrome. 2021. 26. Koppenaal et al. (Ref ID 53000). The 3-Month Effectiveness of a Stratified Blended Physiotherapy Intervention in Patients With Nonspecific Low Back Pain: cluster Randomized Controlled Trial. 2022. 27. Kotteeswaran et al. (Ref ID 1887). Effect of Proprioceptive Neuromuscular Facilitation stretching and dynamic soft tissue mobilization on hamstring flexibility in subjects with low back ache - single blinded randomised controlled study. 2014. 28. Layanna et al. (Ref ID 43013). The effects of the exercises of segmental stabilization in low back pain. 2015. 29. Li et al. (Ref ID 43201). Rehabilitation effect of exercise with soft tissue manipulation in patients with lumbar muscle strain. 2017. 30. Lin et al. (Ref ID 52015). Tai Chi Yun hand to improve the proprioception of patient with chronic low back pain for 30 cases. 2015.* 31. Lisi et al. (Ref ID 387). A Pulsed Electromagnetic Field Therapy Device for Non-Specific Low Back Pain: a Pilot Randomized Controlled Trial. 2019. 32. Lyons et al. (Ref ID 1389). Core and whole body vibration exercise influences muscle sensitivity and posture during a military foot march. 2021. 33. Malfliet et al. (Ref ID 1329). Effect of Pain Neuroscience Education Combined With Cognition-Targeted Motor Control Training on Chronic Spinal Pain: a Randomized Clinical Trial. 2018. 34. Manikandan et al. (Ref ID 53126). Effectiveness of Pilates exercises on non-specific low back pain to determine pain and disability. 2021. 35. Minobes-Molina et al. (Ref ID 1905). Effectiveness of specific stabilization exercise compared with traditional trunk exercise in women with non-specific low back pain: a pilot randomized controlled trial. 2020. 36. Moseley et al. (Ref ID 1585). Joining forces - Combining cognition-targeted motor control training with group or individual pain physiology education: a successful treatment for chronic low back pain. 2003. 37. Mumtaz et al. (Ref ID 53072). Effect of core stability exercises with conventional physiotherapy in reducing pain among patients with non-specific low back pain: RCT. 2021. 38. Natraj et al. (Ref ID 46153). Efficacy of slump stretching in combinations with conventional therapy in chronic non-radicular low back pain - A randomized control trial. 2018. 39. Nayyab et al. (Ref ID 1560). The effects of an exercise programme for core muscle strengthening in patients with low back pain after Caesarian-section: a single blind randomized controlled trial. 2021. 40. Nechvatal et al. (Ref ID 1720). Comparison of the effect of the McKenzie method and spiral stabilization in patients with low back pain: a prospective, randomized clinical trial. 2021. 41. Ning et al. (Ref ID 45039). Bookmark and Share Email this article Print this article Clinical study on five mimic -animal boxing combined with core muscular strength exercise for the treatment of nonspecific low back pain. 2015. 42. Oh et al. (Ref ID 47604). Comparison of Effects of Abdominal Draw-In Lumbar Stabilization Exercises with and without Respiratory Resistance on Women with Low Back Pain: A Randomized Controlled Trial. 2020. 43. Oka et al. (Ref ID 43711). The effect of the "One Stretch" exercise on the improvement of low back pain in Japanese nurses: a large-scale, randomized, controlled trial. 2018. 44. Ornek et al. (Ref ID 654). The effectiveness of local steroid injection and exercise for the treatment of thoracolumbar junction syndrome (maigne syndrome). 2013. 45. Park et al. (Ref ID 1600). Comparative Analysis of Pain, Muscle Strength, Disability, and Quality of Life in Middle-Aged and Older Adults After Web Video Lower Back Exercise. 2021. 46. Patel et al. (Ref ID 1144). Primum non nocere: shared informed decision making in low back pain--a pilot cluster randomised trial. 2014. 47. Ramos et al. (Ref ID 43751). Comparison Between Transcutaneous Electrical Nerve Stimulation and Stabilization Exercises in Fatigue and Transversus Abdominis Activation in Patients With Lumbar Disk Herniation: a Randomized Study. 2018. 48. Reddingtion et al. (Ref ID 173). Does early intervention improve outcomes in the physiotherapy management of lumbar radicular syndrome? Results of the POLAR pilot randomised controlled trial. 2018. 49. Rodriguez-Romero et al. (Ref ID 44373). A Therapeutic Exercise Program Improves Pain and Physical Dimension of Health-Related Quality of Life in Young Adults: A Randomized Controlled Trial. 2019. 50. Rosario et al. (Ref ID 1803). Relief from Back Pain Through Postural Adjustment: a Controlled Clinical Trial of the Immediate Effects of Muscular Chains Therapy (MCT). 2014. 51. Rungthip et al. (Ref ID 43770). The effect of core stabilization exercise on lumbar joint position sense in patients with subacute non-specific low back pain: a randomized controlled trial. 2018. 52. Saiklang et al. (Ref ID 47683). An Evaluation of an Innovative Exercise to Relieve Chronic Low Back Pain in Sedentary Workers. 2020. 53. Saleem et al. (Ref ID 1582). Effect of exercise on inter-recti distance and associated low back pain among post-partum females: a randomized controlled trial. 2021. 54. Sandal et al. (Ref ID 1736). Effectiveness of App-Delivered, Tailored Self-management Support for Adults With Lower Back Pain-Related Disability: a selfBACK Randomized Clinical Trial. 2021. 55. Sanika et al. (Ref ID 693). Comparison of Glutues Maximus Activation to Flexion Bias Exercises Along with MET Technique in Subjects with Anterior Rotated Sacroiliac Joint Dysfunction-a Randomised Controlled Trial. 2021. 56. Schenk et al. (Ref ID 52022). A randomized trial comparing interventions in patients with lumbar posterior derangement. 2003.* 57. Schulz et al. (Ref ID 44389). Spinal manipulative therapy and exercise for older adults with chronic low back pain: a randomized clinical trial. 2019. 58. Sharma et al. (Ref ID 1643). Efficacy of Manual Therapy versus Conventional Physical Therapy in Chronic Low Back Pain Due to Lumbar Spondylosis. A Pilot Study. 2015. 59. Shebib et al. (Ref ID 44397). Randomized controlled trial of a 12-week digital care program in improving low back pain. 2019. 60. Sihawong et al. (Ref ID 1902). Efficacy of risk factor education on pain intensity and disability in office workers with nonspecific neck or low back pain: a pilot cluster randomized clinical trial. 2020. 61. Sipko et al. (Ref ID 1485). Acute effects of proprioceptive neuromuscular facilitation exercises on the postural strategy in patients with chronic low back pain. 2021. 62. Song et al. (Ref ID 45009). A Study on Effect of Taijiquan on Lumbar Disc Protrusion [J]. 2008. 63. Sonmezer et al. (Ref ID 1530). The effects of clinical Pilates exercises on functional disability, pain, quality of life and lumbopelvic stabilization in pregnant women with low back pain: a randomized controlled study. 2020. 64. Sousa Filho et al. (Ref ID 53230). Neurodynamic exercises provide no additional benefit to extension-oriented exercises in people with chronic low back-related leg pain and a directional preference: a randomized clinical trial. 2022. 65. Srivastava et al. (Ref ID 47737). Short-term effect of muscle energy technique and mechanical diagnosis and therapy in sacroiliac joint dysfunction: A pilot randomized clinical trial. 2020. 66. Szulc et al. (Ref ID 1554). Impact of Mckenzie method therapy enriched by muscular energy techniques on subjective and objective parameters related to spine function in patients with chronic low back pain. 2015. 67. Telles et al. (Ref ID 1930). A Randomized Controlled Trial to Assess Pain and Magnetic Resonance Imaging-Based (MRI-Based) Structural Spine Changes in Low Back Pain Patients after Yoga Practice. 2016. 68. Talbot et al. (Ref ID 1810). Non-pharmacological Home Therapies for Subacute Low Back Pain in Active Duty Military Personnel: a Randomized Controlled Trial. 2021. 69. Tavafian et al. (Ref ID 278). Treatment of low back pain: first extended follow up of an original trial (NCT00600197) comparing a multidisciplinary group-based rehabilitation program with oral drug treatment alone up to 24 months. 2017. 70. Torlak et al. (Ref ID 53083). The effect of intermittent diet and/or physical therapy in patients with chronic low back pain: a single-blinded randomized controlled trial. 2022. 71. Turner et al. (Ref ID 43868). Randomized trial of chronic pain self-management program in the community or clinic for low-income primary care patients. 2018. 72. Vicente et al. (Ref ID 43626). Pain perception and low back pain functional disability after a 10-week core and mobility training program: A pilot study. 2018. 73. Wahyuddin et al. (Ref ID 47824). Immediate effects of muscle energy technique and stabilization exercise in patients with chronic low back pain with suspected facet joint origin: A pilot study. 2020. 74. Yuan et al. (Ref ID 46081). Observation on the forward curative effect of back muscle functional exercise in the treatment of lumbar vertebra small joint synovial incarcerated. 2016. 75. Zaworski et al. (Ref ID 1816). The effect of motor control training according to the Kinetic Control concept on the back pain of female football players. 2021. 76. Zhang et al. (Ref ID 44493). Curative effects on muscle function and proprioception in patients with chronic lumbar disk herniation using isokinetic trunk muscle strength training. 2019. 77. (Ref ID 1548). The Effect of Lumbar Stability Exercise Program on Sedentary Life Female, Lumbosacral Region Angle, Muscular Strength, Physical Fitness and Pain Scale. 2017. 78. (Ref ID 1568). An Exploratory Study of Low Back Pain among Yoga Practisioners And Non Yoga Practisioners In Relation To Anger And Heart Rate. 2020. 79. (Ref ID 1606). Effects of Manual Therapy on Pain and Function of Patients with Chronic Low Back Pain. 2017. 80. (Ref ID 1608). Effect of Visual Feedback Squat Motion on Core Muscles Thickness of Young People with Lower Back Pain. 2019. 81. (Ref ID 60002). Observations on the efficacy of electroacupuncture plus turgunmed training system in treating lumbar intervertebral disc herniation. 2016. 82. (Ref ID 60003). The effectiveness of balneotherapy and thermal aquatic exercise in postoperative persistent lumbar pain syndrome. 2021. |
| Ineligible intervention  n = 1 | 1. Bade et al. (Ref ID 41626). Effects of manual therapy and exercise targeting the hips in patients with low-back pain – A randomized controlled trial. 2016. |
| Ineligible comparison  n = 182 | 1. Afzal et al. (Ref ID 53165). Effects of virtual reality exercises and routine physical therapy on pain intensity and functional disability in patients with chronic low back pain. 2022. 2. Abdel-Aziem et al. (Ref ID 53158). The Effects of Stabilization Exercises Combined With Pelvic Floor Exercise in Women With Nonspecific Low Back Pain: a Randomized Clinical Study. 2021. 3. Aboufazeli et al. (Ref ID 1903). Recovery of the lumbar multifidus muscle size in chronic low back pain patients by strengthening hip abductors: a randomized clinical trial. 2021. 4. Ahadi et al. (Ref ID 1443). A Randomized Clinical Trial on the Effect of Biofeedback on Pain and Quality of Life of Patients With Chronic Coccydynia. 2020. 5. Ahmadi et al. (Ref ID 46944). Comparison of the effects of the Feldenkrais method versus core stability exercise in the management of chronic low back pain: a randomised control trial. 2020. 6. Ahmadnezhad et al. (Ref ID 53206). Increasing Activity and Co-contraction of Local Muscles in the Core Region and Lumbopelvic Motor Control through Immediate Respiratory Muscle Training: a Double-Blind Randomized Controlled Trial. 2022. 7. Ahmed et al. (Ref ID 1612). Effects of dynamic stabilization exercises and muscle energy technique on selected biopsychosocial outcomes for patients with chronic non-specific low back pain: a double-blind randomized controlled trial. 2021. 8. Ak et al. (Ref ID 46068). Comparative Efficacy of Core Stabilization Exercise and Pilates Exercise on Patients with Nonspecific Chronic Low Back Pain. 2016. 9. Akodu et al. (Ref ID 46950). Effects of core stabilization and McKenzie back extension exercises on pain, disability and insomnia in patients with non-specific chronic low back pain. 2020. 10. Alfuth et al. (Ref ID 40001). [Chronic low back pain: Comparison of mobilization and core stability exercises]. [German]. 2016. 11. Alhowimel et al. (Ref ID 484). Feasibility study and process evaluation of MRI plus physiotherapy vs. physiotherapy alone in non-specific chronic low back pain among patients in Saudi Arabia. 2020. 12. Almhdawi et al. (Ref ID 46964). Efficacy of an innovative smartphone application for office workers with chronic non-specific low back pain: a pilot randomized controlled trial. 2020. 13. Altinbilek et al. (Ref ID 46974). A comparison of application frequency of physical therapy modalities in patients with chronic mechanical low back pain. 2020. 14. Amit et al. (Ref ID 45000). Effect of trunk muscles stabilization exercises and general exercises on pain in recurrent nonspecific low back ache. 2013. 15. Amorim et al. (Ref ID 43978). Integrating Mobile-health, health coaching, and physical activity to reduce the burden of chronic low back pain trial (IMPACT): a pilot randomised controlled trial. 2019. 16. Areeudomwong et al. (Ref ID 43357). Proprioceptive neuromuscular facilitation training improves pain-related and balance outcomes in working-age patients with chronic low back pain: a randomized controlled trial. 2018. 17. Areeudomwong et al. (Ref ID 46241). Comparison of Core Stabilisation Exercise and Proprioceptive Neuromuscular Facilitation Training on Pain-related and Neuromuscular Response Outcomes for Chronic Low Back Pain: A Randomised Controlled Trial. 2019. 18. Atilgan et al. (Ref ID 294). The effects of breathing exercises in mothers of children with special health care needs: a randomized controlled trial. 2021. 19. Batibay et a. (Ref ID 1935). Effect of Pilates mat exercise and home exercise programs on pain, functional level, and core muscle thickness in women with chronic low back pain. 2020. 20. Baskan et al. (Ref ID 1958). Effectiveness of a clinical Pilates program in women with chronic low back pain: a randomized controlled trial. 2021. 21. Batool et al. (Ref ID 46259). Effectiveness of core stability with and without neck extension on pain intensity and functional disability in patients with chronic low back pain. 2019. 22. Belavy et al. (Ref ID 53181). Exercise may impact on lumbar vertebrae marrow adipose tissue: randomised controlled trial. 2022. 23. Bello (Ref ID 43378). Effects of lumbar stabilisation and treadmill exercise on function in patients with chronic mechanical low back pain. 2018. 24. Bertrand et al. (Ref ID 53257). Self-Treatment of Chronic Low Back Pain Based on a Rapid and Objective Sacroiliac Asymmetry Test: a Pilot Study. 2021. 25. Blanchard et al. (Ref ID 53267). Technical Feasibility of Supervision of Stretching Exercises by a Humanoid Robot Coach for Chronic Low Back Pain: the R-COOL Randomized Trial. 2022. 26. Brodsky et al. (Ref ID 44011). Randomized Pilot Trial for a Community-Based Group Stretching Exercise Program for Chronic Low Back Pain. 2019. 27. Bruehl et al. (Ref ID 1936). Does aerobic exercise training alter responses to opioid analgesics in individuals with chronic low back pain? a randomized controlled trial. 2020. 28. Buran Cirak et al. (Ref ID 1661). Effect of Sustained Natural Apophyseal Glides on Stiffness of Lumbar Stabilizer Muscles in Patients With Nonspecific Low Back Pain: randomized Controlled Trial. 2021. 29. Buttagat et al. (Ref ID 44015). Effectiveness of traditional Thai self-massage combined with stretching exercises for the treatment of patients with chronic non-specific low back pain: A single-blinded randomized controlled trial. 2019. 30. Calatayud et al. (Ref ID 47050). Effectiveness of a Group-Based Progressive Strength Training in Primary Care to Improve the Recurrence of Low Back Pain Exacerbations and Function: A Randomised Trial. 2020. 31. Castro et al. (Ref ID 53020). Cognitive functional therapy compared with core exercise and manual therapy in patients with chronic low back pain: randomised controlled trial. 2022. 32. Cerini et al. (Ref ID 53001). 12 weeks high intensity interval training versus moderate intensity continuous training in chronic low back pain subjects: a randomised single-blinded feasibility study. 2022. 33. Collazo et al. (Ref ID 1617). Exercise and auricular acupuncture for chronic low-back pain. 2012. 34. Cox et al. (Ref ID 45005). A randomised controlled trial of yoga for the treatment of chronic low back pain: results of a pilot study. 2010. 35. Dadarkhah et al. (Ref ID 1863). Remote Versus in-Person Exercise Instruction for Chronic Nonspecific Low Back Pain Lasting 12 Weeks or Longer: a Randomized Clinical Trial. 2020. 36. Das et al. (Ref ID 44089). Effectiveness of multipurpose health-worker-led exercise therapy on pain reduction among patients with chronic nonspecific low backache in primary health-care setting: A randomized control trial. 2019. 37. Daulat et al. (Ref ID 46059). A phase II pilot study comparing a home total body strengthening programme plus manual therapy with a standard physiotherapy exercise regimen plus manual therapy in the management of chronic low back pain. 2014. 38. Daulat et al. (Ref ID 46070). A pragmatic randomized controlled trial to compare a novel group physiotherapy programme with a standard group exercise programme for managing chronic low back pain in primary care. 2016. 39. De Giorgio et al. (Ref ID 43432). Effectiveness of yoga combined with back school program on anxiety, kinesiophobia and pain in people with non-specific chronic low back pain: a prospective randomized trial. 2018. 40. del Pozo-Cruz et al. (Ref ID 45007). Effects of whole body vibration therapy on main outcome measures for chronic non-specific low back pain: a single-blind randomized controlled trial. 2011. 41. Demirel et al. (Ref ID 44102), Stabilization exercise versus yoga exercise in non-specific low back pain: Pain, disability, quality of life, performance: a randomized controlled trial. 2019. 42. de Oliveira et al. (Ref ID 47137). Osteopathic manipulation treatment versus therapeutic exercises in patients with chronic nonspecific low back pain: A randomized, controlled and double-blind study. 2020. 43. de Oliveira et al. (Ref ID 53005). Adding Physical Activity Coaching and an Activity Monitor Was No More Effective Than Adding an Attention Control Intervention to Group Exercise for Patients With Chronic Nonspecific Low Back Pain (PAyBACK Trial): a Randomized Trial. 2022. 44. de Oliveira et al. (Ref ID 53270). Treatment of non-specific chronic low back pain: resistance training with or without using weights? 2021. 45. Ehsani et al. (Ref ID 46351). The Effects of Stabilization Exercise on the Thickness of Lateral Abdominal Muscles During Standing Tasks in Women With Chronic Low Back Pain: A Randomized Triple-Blinded Clinical Trial Study. 2019. 46. Fairbank et al. (Ref ID1929). Randomised controlled trial to compare surgical stabilisation of the lumbar spine with an intensive rehabilitation programme for patients with chronic low back pain: the MRC spine stabilisation trial. 2005. 47. Fang et al. (Ref ID 45036). The effect of Wuqinxi Exercise on mechanics characteristic of abdominal and back muscles and pain in patients with chronic nonspecific low back pain. 2015. 48. Ferreira et al. (Ref ID 43146). Maitland in chronic lumbar pain of young adults improves pain and functionality. 2017. 49. Ferrell et al. (Ref ID 52004). A randomized trial of walking versus physical methods for chronic pain management. 1997.* 50. Finta et al. (Ref ID 1924). The effect of diaphragm training on lumbar stabilizer muscles: a new concept for improving segmental stability in the case of low back pain. 2018. 51. Fouda et al. (Ref ID 1625). Effects of proprioceptive neuromuscular facilitation techniques in treating chronic nonspecific low back pain patients. 2021. 52. Freitas et al. (Ref ID 1873). Comparison between isokinetic dynamometer and therapeutic ball exercises in chronic low-back pain of mechanical origin. 2008. 53. Galan-Martin et al. (Ref ID 47203). Pain neuroscience education and physical therapeutic exercise for patients with chronic spinal pain in Spanish physiotherapy primary care: a pragmatic randomized controlled trial. 2020. 54. Garcia et al. (Ref ID 1934). An 8-Week Self-Administered At-Home Behavioral Skills-Based Virtual Reality Program for Chronic Low Back Pain: double-Blind, Randomized, Placebo-Controlled Trial Conducted During COVID-19. 2021. 55. Gardner et al. (Ref ID 44139). Combined education and patient-led goal setting intervention reduced chronic low back pain disability and intensity at 12 months: a randomised controlled trial. 2019. 56. Ge et al. (Ref ID 53137). Effects of core stability training on older women with low back pain: a randomized controlled trial. 2022. 57. Ghasemi et al. (Ref ID 47210). Comparison of the effects of craniosacral therapy, muscle energy technique, and sensorimotor training on non-specific chronic low back pain. 2020. 58. Gholami Borujeni et al. (Ref ID 46384). Reduction of postural sway in athletes with chronic low back pain through eight weeks of inspiratory muscle training: A randomized controlled trial. 2019. 59. Gibbs et al. (Ref ID 53054). Does a powerlifting inspired exercise programme better compliment pain education compared to bodyweight exercise for people with chronic low back pain? A multicentre, single-blind, randomised controlled trial. 2022. 60. Gorji et al. (Ref ID 53234). Pain Neuroscience Education and Motor Control Exercises versus Core Stability Exercises on Pain, Disability, and Balance in Women with Chronic Low Back Pain. 2022. 61. Gul et al. (Ref ID 1525). Physiotherapy combined with therapeutic neuroscience education versus physiotherapy alone for patients with chronic low back pain: a pilot, randomized-controlled trial. 2021. 62. Hosseinifar et al. (Ref ID 1923). Comparison of balance and stabilizing trainings on balance indices in patients suffering from nonspecific chronic low back pain. 2018. 63. Ibrahim et al. (Ref ID 43548). Motor control exercise and patient education program for low resource rural community dwelling adults with chronic low back pain: a pilot randomized clinical trial. 2018. 64. In et al. (Ref ID 1956). Effects of the Multidimensional Treatment on Pain, Disability, and Sitting Posture in Patients with Low Back Pain: a Randomized Controlled Trial. 2021. 65. Jalalvandi et al. (Ref ID 53135). Effects of back exercises versus transcutaneous electric nerve stimulation on relief of pain and disability in operating room nurses with chronic non-specific LBP: a randomized clinical trial. 2022. 66. Joseph et al. (Ref ID 43561). Comparison of Effects Between Core Stability Training and Sports Massage Therapy Among EliteWeightlifters with Chronic Non-Specific Low Back Pain: A Randomized Cross-Over Study. 2018. 67. Kamali et al. (Ref ID 44204). Comparison of manipulation and stabilization exercises in patients with sacroiliac joint dysfunction patients: A randomized clinical trial. 2019. 68. Kanas et al. Home-based exercise therapy for treating non-specific chronic low back pain. 2018.* 69. Kang et al. (Ref ID 43569). Effect of 6-week lumbar stabilization exercise performed on stable versus unstable surfaces in automobile assembly workers with mechanical chronic low back pain. 2018. 70. Kankaanpa et al. (Ref ID 1629). The efficacy of active rehabilitation in chronic low back pain. Effect on pain intensity, self-experienced disbility, and lumbar fatigability. 1999. 71. Kanwal et al. (Ref ID 1937). Effects of core muscle stability on low back pain and quality of life in post- menopausal women: a comparative study. 2021. 72. Khandhar et al. (Ref ID 47374). Comparative effect of trunk balance exercise over conventional back care exercise in patients with chronic mechanical low back pain. 2020. 73. Kim et al. (Ref ID 44220). Effectiveness of Simulated Horseback Riding for Chronic Low Back Pain Patients: a Randomized Controlled Trial. 2019. 74. Kim et al. (Ref ID 47385). Effects of 3D Moving Platform Exercise on Physiological Parameters and Pain in Patients with Chronic Low Back Pain. 2020. 75. Kim et al. (Ref ID 47387). Classification-Specific Treatment Improves Pain, Disability, Fear-Avoidance Beliefs, and Erector Spinae Muscle Activity During Walking in Patients With Low Back Pain Exhibiting Lumbar Extension-Rotation Pattern: A Randomized Controlled Trial. 2020. 76. Kim et al. (Ref ID 53077). Effect of direct vibration on the activity of deep trunk muscles of patients with non-specific chronic low back pain. 2022. 77. Kim et al. (Ref ID 43578). Effect of Whole Body Horizontal Vibration Exercise in Chronic Low Back Pain Patients: Vertical Versus Horizontal Vibration Exercise. 2018. 78. Kole-Snijders et a. (Ref ID 1630). Chronic low-back pain: what does cognitive coping skills training add to operant behavioral treatment? Results of a randomized clinical trial. 1999. 79. Krishna et al. (Ref ID 47403). Yoga-Based Relaxation Technique Facilitates Sustained Attention in Patients with Low Back Pain: A Pilot Study. 2020. 80. Kuvaz-Izi et al. (Ref ID 43600). Effectiveness of yoga and educational intervention on disability, anxiety, depression, and pain in people with CLBP: A randomized controlled trial. 2018. 81. Kwon et al. (Ref ID 47409). The effects of lumbar stabilization exercise on transversus abdominis muscle activation capacity and function in low back pain patients. 2020. 82. Lambeek et al. (Ref ID 52013). Effect of integrated care for sick listed patients with chronic low back pain: economic evaluation alongside a randomised controlled trial. 2010.* 83. Lara-Palomo et al. (Ref ID 53038). Comparison of the effectiveness of an e-health program versus a home rehabilitation program in patients with chronic low back pain: a double blind randomized controlled trial. 2022. 84. Leeuw et al. (Ref ID 45014). Exposure in vivo versus operant graded activity in chronic low back pain patients: results of a randomized controlled trial. 2008. 85. Lena et al. (Ref ID 53218). The Mezieres Method as a Novel Treatment for Elite Spanish Second-Division Soccer League Players With Low Back Pain: a Randomized Controlled Trial. 2022. 86. Li et al. (Ref ID 1892). The Effect of Virtual Reality Training on Anticipatory Postural Adjustments in Patients with Chronic Nonspecific Low Back Pain: a Preliminary Study. 2021. 87. Liu et al. (Ref ID 1636). Acupuncture and moxibustion cooperated with sports therapy in treating lumbago in 65 cases. 1988. 88. Lomond et al. (Ref ID 52016). Altered postural responses persist following physical therapy of general versus specific trunk exercises in people with low back pain. 2014.* 89. Luz et al. (Ref ID 46326). Neuromuscular electrical stimulation associated with core stability exercises in nonspecific postural low back pain: a randomized clinical trial. 2019. 90. Mataran-Penarrocha et al. (Ref ID 47459). Comparison of efficacy of a supervised versus non-supervised physical therapy exercise program on the pain, functionality and quality of life of patients with non-specific chronic low-back pain: a randomized controlled trial. 2020. 91. Matheve et al. (Ref ID 47460). Virtual reality distraction induces hypoalgesia in patients with chronic low back pain: a randomized controlled trial. 2020. 92. Matos et al. (Ref ID 47461). Analysis of pain symptoms, flexibility and hydroxyproline concentration in individuals with low back pain submitted to Global Postural Re-education and stretching. 2020. 93. Mazloum et al. (Ref ID 40022). Comparing the effects of Pilates training and McKenzie exercises on core muscles cross-sectional area and strength in patients with chronic non-specific low back pain: A clinical trial. [Persian]. 2016. 94. Mbada et al. (Ref ID 17078). Influence of Mckenzie protocol and two modes of endurance exercises on health-related quality of life of patients with long-term mechanical low-back pain. 2014. 95. Mbada et al. (Ref ID 44281). Comparative Efficacy of Clinic-Based and Telerehabilitation Application of Mckenzie Therapy in Chronic Low-Back Pain. 2019. 96. Mendes Tozim et al. (Ref ID 1557). Efficacy of the Pilates versus general exercises versus educational workshops on neuromuscular parameters: a randomized controlled trial. 2021. 97. Mendes et al. (Ref ID 53045). Core stabilisation exercises reduce chronic low back pain in Air Force fighter pilots: a randomised controlled trial. 2022. 98. Michalsen et al. (Ref ID 1904). Yoga, Eurythmy Therapy and Standard Physiotherapy (YES-Trial) for Patients With Chronic Non-specific Low Back Pain: a Three-Armed Randomized Controlled Trial. 2021. 99. Micke et al. (Ref ID 1414).Similar Pain Intensity Reductions and Trunk Strength Improvements Following Whole-Body Electromyostimulation vs. Whole-Body Vibration vs. Conventional Back-Strengthening Training in Chronic Non-specific Low Back Pain Patients: a Three-Armed Randomized Controlled Trial. 2021. 100. Mitova et al. (Ref ID 300). Kinesiotape methodology for chronic pain syndrome in the lumbosacral region. 2021. 101. Miyachi et al. (Ref ID 53143). Effects of dynamic lumbar motor control training on lumbar proprioception: a randomized controlled trial. 2022. 102. Miyamoto et al. (Ref ID 1675). Education With Therapeutic Alliance Did Not Improve Symptoms in Patients With Chronic Low Back Pain and Low Risk of Poor Prognosis Compared to Education Without Therapeutic Alliance: a Randomized Controlled Trial. 2021. 103. Moncelon et al. (Ref ID 46067). Méthode McKenzie et lombalgiques chroniques avec Préférence Directionnelle. 2015. 104. Nambi et al. (Ref ID 1652). Isokinetic training - its radiographic and inflammatory effects on chronic low back pain: a randomized controlled trial. 2020. 105. Nambi et al. (Ref ID 1933). Radiological (Magnetic Resonance Image and Ultrasound) and biochemical effects of virtual reality training on balance training in football players with chronic low back pain: a randomized controlled study. 2020. 106. Nambi et al. (Ref ID 47499). Isokinetic back training is more effective than core stabilization training on pain intensity and sports performances in football players with chronic low back pain: A randomized controlled trial. 2020. 107. Nambi et al. (Ref ID 47500). Virtual reality or Isokinetic training; its effect on pain, kinesiophobia and serum stress hormones in chronic low back pain: A randomized controlled trial. 2020. 108. Nambi et al. (Ref ID 47501). Comparative Effects of Isokinetic Training and Virtual Reality Training on Sports Performances in University Football Players with Chronic Low Back Pain-Randomized Controlled Study. 2020. 109. Nambi et al. (Ref ID 53016). Clinical and physical efficiency of virtual reality games in soccer players with low back pain. 2021. 110. Narouei et al. (Ref ID 47502). Effects of core stabilization exercises on thickness and activity of trunk and hip muscles in subjects with nonspecific chronic low back pain. 2020. 111. Neyaz et al. (Ref ID 44318). Effectiveness of Hatha Yoga Versus Conventional Therapeutic Exercises for Chronic Nonspecific Low-Back Pain. 2019. 112. Nishimura et al. (Ref ID 53084). Effect of Interventions for Improving Lumbar Motor Control on Low Back Pain in Sedentary Office Workers: a Randomized Controlled Trials. 2021. 113. Ojoawo et al. (Ref ID 451). Therapeutic efficacy of Lofnac Gel via phonophoresis in the management of chronic nonspecific low back pain: a randomised controlled trial. 2015. 114. O'Keeffe et al. (Ref ID 47600). Cognitive functional therapy compared with a group-based exercise and education intervention for chronic low back pain: a multicentre randomised controlled trial (RCT). 2020. 115. Otadi et al. (Ref ID 1653). Effects of combining diaphragm training with electrical stimulation on pain, function, and balance in athletes with chronic low back pain: a randomized clinical trial. 2021. 116. Owen et al. (Ref ID 47617). Exercise for the intervertebral disc: a 6-month randomised controlled trial in chronic low back pain. 2020. 117. Ozcan et al. (Ref ID 1725). The Effectiveness of Matrix Rhythm Therapy in Patients with Chronic Low Back Pain. 2021. 118. Ozden et al. (Ref ID 1795). The effect of video exercise-based telerehabilitation on clinical outcomes, expectation, satisfaction, and motivation in patients with chronic low back pain. 2021. 119. Park et al. (Ref ID 47632). Effects of lumbar segmental stabilization exercise and respiratory exercise on the vital capacity in patients with chronic back pain. 2020. 120. Patil et al. (Ref ID 43727). A randomized trial comparing effect of yoga and exercises on quality of life in among nursing population with chronic low back pain. 2018. 121. Peng et al. (Ref ID 53172). Efficacy of Therapeutic Aquatic Exercise vs Physical Therapy Modalities for Patients With Chronic Low Back Pain: a Randomized Clinical Trial. 2022. 122. Polaski et al. (Ref ID 1655). Integrated Meditation and Exercise Therapy: a Randomized Controlled Pilot of a Combined Nonpharmacological Intervention Focused on Reducing Disability and Pain in Patients with Chronic Low Back Pain. 2021. 123. Puntumetakul et al. (Ref ID 1951). The effects of core stabilization exercise with the abdominal drawing-in maneuver technique versus general strengthening exercise on lumbar segmental motion in patients with clinical lumbar instability: a randomized controlled trial with 12-month follow-up. 2021. 124. Salik Sengul et al. (Ref ID 1583). Effects of stabilization exercises on disability, pain and core stability in patients with non-specific low back pain: a randomized controlled trial, 2021. 125. Suh et al. 2019. (Ref ID 44414). The effect of lumbar stabilization and walking exercises on chronic low back pain: A randomized controlled trial. 2019. 126. Rabiei et al. (Ref ID 47662). Comparing Pain Neuroscience Education Followed by Motor Control Exercises With Group-Based Exercises for Chronic Low Back Pain: A Randomized Controlled Trial. 2020. 127. Rae et al. (Ref ID 1939). Yoga vs Stretching in Veterans With Chronic Lower Back Pain and the Role of Mindfulness: a Pilot Randomized Controlled Trial. 2020. 128. Rajpal et al. (Ref ID 45017). A study on efficacy of Pilates & Pilates & Mckenzie exercises in postural low back pain—a rehabilitative protocol. 2008. 129. Raza et al. (Ref ID 1601). Effectiveness of spinal stabilization exercises with and without stretching of latissimus dorsi muscle in chronic mechanical low back pain. 2020. 130. Rezai et al. (Ref ID 1801). Comparing the Effects of Different Types of Aquatic Walking on Endurance and Electrical Activities of Spine Extensor Muscles in Men with Nonspecific Chronic Back Pain. 2020. 131. Saiklang et al. (Ref ID 1804). The immediate effect of the abdominal drawing-in maneuver technique on stature change in seated sedentary workers with chronic low back pain. 2021. 132. Salas et al. (Ref ID 43773). The relative efficacy of two exercise methods for older adults with chronic low back pain: a preliminary randomized control study. 2018. 133. Samir et al. (Ref ID 45035). Mulligan versus Maitland Mobilizations In Patients with Chronic Low Back Dysfunction. 2016. 134. Santos et al. (Ref ID 53164). Effects of two training programs on health variables in adults with chronic low back pain: a randomized clinical trial. 2022. 135. Sarker et al. (Ref ID 44383). Effect of spinal manipulation on pain sensitivity, postural sway, and health-related quality of life among patients with non-specific chronic low back pain: A randomised control trial. 2019. 136. Sawant et al. (Ref ID 44386). Effect of Hydrotherapy Based Exercises for Chronic Nonspecific Low Back Pain. 2019. 137. Schmidt et al. (Ref ID 47698). The effect of an integrated multidisciplinary rehabilitation programme alternating inpatient interventions with home-based activities for patients with chronic low back pain: a randomized controlled trial. 2020. 138. Sheeran et al. (Ref ID 45021). Classification-guided versus generalized postural intervention in subgroups of nonspecific chronic low back pain: a pragmatic randomized controlled study. 2013. 139. Schemer et al. (Ref ID 497). Exposure and cognitive-behavioural therapy for chronic back pain: An RCT on treatment processes. 2019. 140. Sherman et al. (Ref ID 47716). T'ai Chi for Chronic Low Back Pain in Older Adults: A Feasibility Trial. 2020. 141. Schmidt et al. (Ref ID 1388). Assessment of a taping method combined with manual therapy as a treatment of non-specific chronic low back pain - a randomized controlled trial. 2021. 142. Semrau et al. (Ref ID 1948). Effects of behavioural exercise therapy on the effectiveness of multidisciplinary rehabilitation for chronic non-specific low back pain: a randomised controlled trial. 2021. 143. Seo et al. (Ref ID 44390). The effects of Gyrotonic expansion system exercise and trunk stability exercise on muscle activity and lumbar stability for the subjects with chronic low back pain. 2019. 144. Singh et al. (Ref ID 46802). Effect of Rhythmic Stabilization Exercise v/s Conventional Physiotherapy on Pain and Disability with Patients of Chronic Mechanical Low Back Pain. 2019. 145. Sipaviciene et al. (Ref ID 47723). Effect of different exercise programs on non-specific chronic low back pain and disability in people who perform sedentary work. 2020. 146. Soares et al. (Ref ID 52024). Effects of school-based exercise program of posture and global postural reeducation on the range of motion and pain levels in patients with chronic low back pain. 2016.* 147. ŠPringrovÁ et al. (Ref ID 47736). Comparison of the impact of two physical therapy methods on pain and disability in patients with non-specific lower back pain: a controlled clinical pilot study. 2020. 148. Stamm et al. (Ref ID 53276). Virtual reality exergame for supplementing multimodal pain therapy in older adults with chronic back pain: a randomized controlled pilot study. 2022. 149. Srivastav et al. (Ref ID 43827). Comparison between Effectiveness of Lumbar Stabilization Exercises and Conventional Physical Therapy in the Management of Mechanical Low Back Pain. 2018. 150. Taheri et a. (Ref ID 298). Extracorporeal shock wave therapy combined with oral medication and exercise for chronic low back pain: a randomized controlled trial. 2021. 151. Teixeira et al. (Ref ID 43061). Evaluation of functional disability and pain in patients with chronic low back pain submitted to physiotherapy. 2016. 152. Thomas et al. (Ref ID 43068). Feasibility and safety of a virtual reality dodgeball intervention for chronic low back pain: a randomized clinical trial [with consumer summary]. 2016. 153. Tsatsakos et al (Ref ID 52005). Effect of Physical Activity on Functional Status in Elderly with Chronic Low Back Pain. 2014.* 154. van Baal et al. (Ref ID 47805). Effects of a movement control and tactile acuity training in patients with nonspecific chronic low back pain and control impairment - a randomised controlled pilot study. 2020. 155. Van Dillen et al. (Ref ID 1813). Effect of Motor Skill Training in Functional Activities vs Strength and Flexibility Exercise on Function in People with Chronic Low Back Pain: a Randomized Clinical Trial. 2020. 156. Verbrugghe et al. (Ref ID 44453). Exercise Intensity Matters in Chronic Nonspecific Low Back Pain Rehabilitation. 2019. 157. Verra et al. (Ref ID 43886). Effectiveness of subgroup-specific pain rehabilitation: a randomized controlled trial in patients with chronic back pain. 2018. 158. Von Korff et al. (Ref ID 938). A trial of an activating intervention for chronic back pain in primary care and physical therapy settings. 2005. 159. Wang et al. (Ref ID 44464). Effects of whole-body vibration exercise for non-specific chronic low back pain: an assessor-blind, randomized controlled trial. 2019. 160. Wang et a. (Ref I D 47834). Effect of Cangguitanxue acupuncture combined with suspension exercise therapy on chronic low back pain. 2020. 161. Waseem et al. (Ref ID 44466). Treatment of disability associated with chronic non-specific low back pain using core stabilization exercises in Pakistani population. 2019. 162. Yalfani et al. (Ref ID 47865). Effects of eight-week water versus mat Pilates on female patients with chronic nonspecific low back pain: Double-blind randomized clinical trial. 2020. 163. Yalfani et al. (Ref ID 53132). Effects of an 8-Week Virtual Reality Training Program on Pain, Fall Risk, and Quality of Life in Elderly Women with Chronic Low Back Pain: double-Blind Randomized Clinical Trial. 2022. 164. Yang C-Y et al. (Ref ID 1580). Pilates-based core exercise improves health-related quality of life in people living with chronic low back pain: a pilot study. 2021. 165. Yang et al. (Ref ID 45029). The effects of whole body vibration on static balance, spinal curvature, pain, and disability of patients with low back pain. 2015. 166. Yao et al. (Ref ID 47868). Effects of Wuqinxi in the Patients with Chronic Low Back Pain: A Randomized Controlled Trial. 2020. 167. Ye et al. (Ref ID 53048). Core Training under Suspension Exercise Therapy on Treatment of Low Back Pain. 2021. 168. Young et al. (Ref ID 52028). Effect of proprioceptive neuromuscular facilitation integration pattern and swiss ball training on pain and balance in elderly patients with chronic back pain. 2015.* 169. Yucesoy et al. (Ref ID 489). Effects of balneological outpatient treatment on clinical parameters and serum cytokine levels in patients with chronic low back pain: a single-blind randomized controlled trial. 2021. 170. Zakari et al. (Ref ID 46901). Comparison of the effects of positional release therapy and lumbar stabilization exercises in the management of chronic mechanical low back pain: a randomized controlled trial. 2019. 171. Zarazycka et al. (Ref ID 43945). Comparative evaluation of rehabilitation systems for patients with chronic lumbar overload syndrome. 2018. 172. Zheng et al. (Ref ID 53057). Does m-health-based exercise (guidance plus education) improve efficacy in patients with chronic low-back pain? A preliminary report on the intervention's significance. 2022. 173. Zhongliang et al. (Ref ID 46188). Spinal mechanical impulsive manipulation versus conventional physiotherapy for chronic low back pain: Differences in range of lumbar motion and trunk muscle strength. 2018. 174. Zurek et al. (Ref ID 53273). Vibrating Exercise Equipment in Middle-Age and Older Women with Chronic Low Back Pain and Effects on Bioelectrical Activity, Range of Motion and Pain Intensity: a Randomized, Single-Blinded Sham Intervention Study. 2022. 175. (Ref ID 1609). The Effects of Stretching and Strengthening Exercise on the Pain, Pelvic Tilt, Functional Disability Index, and Balance Ability of Patients with Chronic Lower Back Pain. 2019. 176. (Ref ID 1659). Effect of Exercise Stage-Matched Intervention for Elderly Women with Chronic Back Pain in the Contemplation and Preparation Stage. 2019. 177. (Ref ID 1764). Change of Pain, Lumbar Sagittal Alignment and Multifidus after Sling Exercise Therapy for Patients with Chronic Low Back Pain. 2018. 178. (Ref ID 1765). Effects of Sling and Resistance Rotation Exercises on Pelvic Rotation and Pain in Patients with Chronic Low Back Pain. 2018. 179. (Ref ID 1771). Short-Term Psychological and Hormonal Effects of Virtual Reality Training on Chronic Low Back Pain in Soccer Players. 2021. 180. (Ref ID 1817). Efficacy of Lumbar Segmental Stabilization Exercises and Breathing Exercises on Segmental Stabilization in Lumbar Instability Patients. 2017. 181. (Ref ID 46083). The effect of stabilization exercises on lumbar lordosis in patients with low back pain. 2017. 182. (Ref ID 60004). Lumbopelvic Stabilization Exercises and McKenzie Method in Low Back Pain Due to Disc Protrusion: a Blind Randomized Clinical Trial. |
| Ineligible outcome  n = 1 | 1. Olkoski. (Ref ID 1724). Comparing the effects of aquatic exercises with or without high intensity on the functional status, muscular endurance, and performance of patients with chronic low back pain. 2021. |
| Full-text not retrievable  n = 12 | 1. Airaksinen et al. (Ref ID 1613). Back school with and without physical training at voluntary group meetings. A randomized controlled trial in patients with nonspecific low back pain. 1997. 2. Baekgaard et al. (Ref ID 1848). Cardiovascular conditioning (aerobics) versus resistance training as a treatment for chronic low back pain (clbp). 1996. 3. Mucha et al. (Ref ID 1586). Back school for nursing staff. 1996. 4. Palekar et al. (Ref ID 52018). A comparative study between core stabilization and superficial strengthening exercises for the treatment of low back pain in two wheeler riders. 2015.* 5. Pan et al. (Ref ID 1798). Evaluation of exercise therapy, magnetotherapy and light therapy in ameliorating the bone mineral density and low back pain in elderly patients with osteoporosis. 2004. 6. Qiu et al. (Ref ID 1735). Biomechanical changes in the muscle and lumbar vertebrae tissue in patients with low back pain caused by sport training. 2007. 7. Si et al. (Ref ID 699). Clinical observation on Mchenzie mechanics principle plus ultrashort wave in treatment of lumbar and leg pain. 2005. 8. Soni et al. (Ref ID 1604). Efficacy of backschool program versus Swiss ball exercise on pain and core endurance in individuals with non-specific low back pain: a comparative study. 2021. 9. Wang et al. (Ref ID 1757). Active exercise and massage for nonspecific low back pain: a clinical randomized controlled trial. 2005 10. Wu et al. (Ref ID 52031). 52 cases of chronic low back pain in the community treated by medium frequency electrotherapy combined with Baduanjin. 2012.* 11. (Ref ID 60000). Curative effect of nonoperative therapy for the lumbar disc herniation. 12. (Ref ID 60005). Application of satisfaction with treatment in the evaluation of exercise therapy improving lumbar pain elderly patients with lumbar disc herniation. |
| Previously assessed in Hayden’s Cochrane review (Duplicates)  n = 10 | 1. Bendix et al. (Ref ID 1782). Functional restoration vrs.active physical out-patient training in chronic low-back pain - a randomized 1-year follow-up study. 1996. 2. Cambron et al. (Ref ID 1786). One-Year Follow-Up of a Randomised Clinical Trial Comparing Flexion Distraction with an Exercise Program for Chronic Low-Back Pain. 2006. 3. Cimarras-Otal et al. (Ref ID 1940). Adapted exercises versus general exercise recommendations on chronic low back pain in industrial workers: a randomized control pilot study. 2020. 4. Gudavalli et al. (Ref ID 1877). A randomized clinical trial comparing flexion-distraction with active exercise for chronic low back pain: a feasibility study. 2004. 5. Hildebrandt et al. (Ref ID 1898). Cesar therapy is temporarily more effective than a standard treatment from the general practitioner in patients with chronic aspecific lower back pain; randomized, controlled and blinded study with 1 year follow-up. 2000. 6. Rydeard et al. (Ref ID 45018). Pilates-based therapeutic exercise: effect on subjects with nonspecific chronic low back pain and functional disability: a randomized controlled trial. 2006. 7. Sengul et al. (Ref ID 1740). Comparing the effects of stabilization and conventional exercises on pain, disability and core stability in patients with chronic low back pain: a randomized controlled trial. 2020. 8. Shamsi et al. (Ref ID 44396). Comparing the effects of static stretching and strengthening in lengthened position on EMG activity of hamstring muscle in patients with chronic non-specific LBP having shortened muscle: a randomised controlled clinical trial. 2019. 9. Smeets et al. (Ref ID 1808). Chronic lower back pain: physical training, gradual activity with problem-solving training or combination - No difference in effectiveness. 2009. 10. (Ref ID 46189). 2018. |

*Identified from supplemental search (n = 22)

**Online Resource 5.** GRADE Evidence Profile tables

GRADE Evidence Profile table 1: ***What are the benefits and harms of aerobic exercise in the management of community-dwelling adults (including older adults aged 60 years and over) with chronic primary low back pain (with or without leg pain) compared with no treatment/no additional treatment?***

| **Certainty assessment** | | | | | | | **№ of patients** | | **Effect** | | **Certainty** | **Importance** |
| --- | --- | --- | --- | --- | --- | --- | --- | --- | --- | --- | --- | --- |
| **№ of studies** | **Study design** | **Risk of bias** | **Inconsistency** | **Indirectness** | **Imprecision** | **Other considerations** | **Aerobic exercise** | **No intervention** | **Relative (95% CI)** | **Absolute (95% CI)** |
| **All Adults** | | | | | | | | | | | | |
| **Pain (high-income country, unclassified presence of leg pain) (follow-up: closest to 2 weeks; assessed with: VAS; benefit indicated by lower values; Scale from: 0 to 10)** | | | | | | | | | | | | |
| 2^1,2,a,b^ | randomised trials | serious^c^ | not serious^d^ | not serious^e^ | very serious^f^ | none | 44 | 44 | - | MD **1.33 lower** (2.27 lower to 0.4 lower) | ⨁◯◯◯ Very low | CRITICAL |
| **Pain (high-income country, unclassified presence of leg pain) (follow-up: closest to 3 months; assessed with: VAS; benefit indicated by lower values; Scale from: 0 to 10)** | | | | | | | | | | | | |
| 1^2,b^ | randomised trials | serious^g^ | not serious^h^ | serious^i^ | very serious^f^ | none | 24 | 23 | - | MD **1.26 lower** (2.51 lower to 0.01 lower) | ⨁◯◯◯ Very low | CRITICAL |
| **Function (high-income country, unclassified presence of leg pain) (follow-up: closest to 2 weeks; assessed with: ODI, Hannover; benefit indicated by lower values; Scale from: 0 to 100)** | | | | | | | | | | | | |
| 2^1,2,a,b^ | randomised trials | serious^c^ | not serious^d^ | not serious^e^ | very serious^f^ | none | 44 | 44 | - | MD **1.3 lower** (3.89 lower to 1.29 higher) | ⨁◯◯◯ Very low | CRITICAL |
| **Function (high-income country, unclassified presence of leg pain) (follow-up: closest to 3 months; assessed with: Hannover; benefit indicated by lower values; Scale from: 0 to 100)** | | | | | | | | | | | | |
| 1^2,b^ | randomised trials | serious^g^ | not serious^h^ | serious^i^ | very serious^f^ | none | 24 | 23 | - | MD **0.9 higher** (5.66 lower to 7.46 higher) | ⨁◯◯◯ Very low | CRITICAL |
| **Health-related quality of life (high-income country, unclassified presence of leg pain) (follow-up: closest to 2 weeks; assessed with: SF-36 (PCS); benefit indicated by lower values; Scale from: 0 to 100)** | | | | | | | | | | | | |
| 1^2,b^ | randomised trials | serious^g^ | not serious^h^ | serious^i^ | very serious^f^ | none | 24 | 24 | - | MD **3.5 higher** (0.05 lower to 7.05 higher) | ⨁◯◯◯ Very low | CRITICAL |
| **Health-related quality of life (high-income country, unclassified presence of leg pain) (follow-up: closest to 2 weeks; assessed with: SF-36 (MCS); benefit indicated by lower values; Scale from: 0 to 100)** | | | | | | | | | | | | |
| 1^2,b^ | randomised trials | serious^g^ | not serious^h^ | serious^i^ | very serious^f^ | none | 24 | 24 | - | MD **1.2 lower** (5.22 lower to 2.82 higher) | ⨁◯◯◯ Very low | CRITICAL |
| **Health-related quality of life (high-income country, unclassified presence of leg pain) (follow-up: closest to 3 months; assessed with: SF-36 (PCS); benefit indicated by lower values; Scale from: 0 to 100)** | | | | | | | | | | | | |
| 1^2,b^ | randomised trials | serious^g^ | not serious^h^ | serious^i^ | very serious^f^ | none | 24 | 23 | - | MD **3.7 higher** (0.05 higher to 7.35 higher) | ⨁◯◯◯ Very low | CRITICAL |
| **Health-related quality of life (high-income country, unclassified presence of leg pain) (follow-up: closest to 3 months; assessed with: SF-36 (MCS); benefit indicated by lower values; Scale from: 0 to 100)** | | | | | | | | | | | | |
| 1^2,b^ | randomised trials | serious^g^ | not serious^h^ | serious^i^ | very serious^f^ | none | 24 | 23 | - | MD **2.2 higher** (3.15 lower to 7.55 higher) | ⨁◯◯◯ Very low | CRITICAL |
| **Adverse events/harms** | | | | | | | | | | | | |
| 1^2,b^ | randomised trials | serious^g^ | not serious^h^ | serious^i^ | very serious^f^ | none | Study authors reported that no adverse events occurred (55 participants total). | | | | ⨁◯◯◯ Very low | CRITICAL |

**CI:** confidence interval; **MCS:** Mental Component Summary; **MD:** mean difference; **ODI:** Oswestry Disability Index; **PCS:** Physical Component Summary; **SF-36:** 36-item Short-Form survey; **VAS:** visual analogue scale

The following was used to guide the ratings.

**Risk of bias:** *Not serious:* trial(s) are rated as overall low risk of bias. *Serious:* some of the weight (>50%) comes from overall unclear risk of bias trial(s).

**Inconsistency:** *Not serious:* high extent of similarity of point estimates and overlap of confidence intervals; statistical heterogeneity (I^2^) is between 0% and 40%, which might not be important. *Serious:* some extent of similarity of point estimates and overlap of confidence intervals; statistical heterogeneity (I^2^) is between 30% and 60%, which could not be explained due to small subgroups and may represent moderate heterogeneity. *Very serious:* little or no similarity of point estimates and overlap of confidence intervals; statistical heterogeneity (I^2^) is between 50% and 90% or 75% and 100%, which could not be explained due to small subgroups and may represent substantial or considerable heterogeneity, respectively.

**Indirectness:** *Not serious:* trial(s) were conducted in different countries or settings. *Serious:* trial(s) were conducted from a single country/setting. *Very serious:* evidence is not directly related to PICO question.

**Imprecision:** *Not serious:* Optimal Information Size (OIS) was reached (i.e., sample sizes with at least 200 participants per group may provide prognostic balance); and the entire confidence interval lies on one side of the threshold that may be considered clinically important (≥10% scale range or SMD ≥0.2 for continuous variables, ≥10% for binary variables), such that the clinical course of action would not differ if the upper versus the lower boundary of the confidence interval represented the truth. *Serious:* OIS would not have been reached (sample sizes with less than 200 participants per group); if the OIS was reached, the clinical course of action might differ if the upper versus the lower boundary of the confidence interval represented the truth. *Very serious:* similar to ‘serious’ but to a greater extent (e.g., very small sample sizes and confidence intervals crossing appreciable benefit and harm).

**Other considerations:** *Not serious:* Publication bias is undetected. *Serious/very serious:* Publication bias is strongly suspected.

#### Explanations

a. Nardin 2022: individually supervised exercise; 8 30-minute sessions.

b. Rotter 2022: group supervised exercise; 8 60-min sessions.

c. Risk of bias: We downgraded once due to potential risk of bias (performance and detection domains).

d. Inconsistency: We did not downgrade. The point estimates are in the same direction with overlapping confidence intervals. Statistical heterogeneity is between 0% and 40%, which might not be important (i.e., I2 = 0%).

e. Indirectness: We did not downgrade because the trials were conducted in different countries (high or upper-middle income).

f. Imprecision: We downgraded twice. The sample size is small (OIS would not have been reached).

g. Risk of bias: We downgraded once due to potential risk of bias (performance domain).

h. Inconsistency: We did not downgrade; however, there are no other trials with which to compare findings.

i. Indirectness: We downgraded once. The trial was conducted in one country (high or upper-middle income).

#### References

1.DMK, Stocco Nardin MR, Aguiar AF, Machado FA, de Oliveira RG, Andraus RAC. Effects of photobiomodulation and deep water running in patients with chronic non-specific low back pain: a randomized controlled trial. . 2022.

2.Rotter G, Ortiz M, Binting S, et al. Mindful Walking in Patients with Chronic Low Back Pain: A Randomized Controlled Trial. 2022.

GRADE Evidence Profile table 2: ***What are the benefits and harms of core strengthening exercise in the management of community-dwelling adults (including older adults aged 60 years and over) with chronic primary low back pain (with or without leg pain) compared with no treatment/no additional treatment?***

| **Certainty assessment** | | | | | | | **№ of patients** | | **Effect** | | **Certainty** | **Importance** |
| --- | --- | --- | --- | --- | --- | --- | --- | --- | --- | --- | --- | --- |
| **№ of studies** | **Study design** | **Risk of bias** | **Inconsistency** | **Indirectness** | **Imprecision** | **Other considerations** | **Core strengthening** | **No intervention** | **Relative (95% CI)** | **Absolute (95% CI)** |
| **ALL ADULTS** | | | | | | | | | | | | |
| **Pain (unclassified presence of leg pain, low-income country) (follow-up: closest to 2 weeks; assessed with: VAS; benefit indicated by lower values; Scale from: 0 to 10)** | | | | | | | | | | | | |
| 1^1,a^ | randomised trials | serious^b^ | not serious^c^ | serious^d^ | very serious^e^ | none | 40 | 40 | - | MD **0.56 lower** (0.94 lower to 0.19 lower) | ⨁◯◯◯ Very low | CRITICAL |
| **Function (unclassified presence of leg pain, low-income country) (follow-up: closest to 2 weeks; assessed with: RMDQ; benefit indicated by lower values; Scale from: 0 to 24)** | | | | | | | | | | | | |
| 1^1,a^ | randomised trials | serious^b^ | not serious^c^ | serious^d^ | very serious^e^ | none | 40 | 40 | - | MD **1.7 lower** (2.42 lower to 0.98 lower) | ⨁◯◯◯ Very low | CRITICAL |
| **Adverse events/harms** | | | | | | | | | | | | |
| 1^1,a^ | randomised trials | serious^b^ | not serious^c^ | serious^d^ | very serious^e^ | none | Authors reported that no adverse events occurred (80 participants total). | | | | ⨁◯◯◯ Very low | CRITICAL |

**CI:** confidence interval; **MD:** mean difference; **RMDQ:** Roland-Morris Disability Questionnaire; **VAS:** visual analogue scale

The following was used to guide the ratings.

**Risk of bias:** *Not serious:* trial(s) are rated as overall low risk of bias. *Serious:* some of the weight (>50%) comes from overall unclear risk of bias trial(s).

**Inconsistency:** *Not serious:* high extent of similarity of point estimates and overlap of confidence intervals; statistical heterogeneity (I^2^) is between 0% and 40%, which might not be important. *Serious:* some extent of similarity of point estimates and overlap of confidence intervals; statistical heterogeneity (I^2^) is between 30% and 60%, which could not be explained due to small subgroups and may represent moderate heterogeneity. *Very serious:* little or no similarity of point estimates and overlap of confidence intervals; statistical heterogeneity (I^2^) is between 50% and 90% or 75% and 100%, which could not be explained due to small subgroups and may represent substantial or considerable heterogeneity, respectively.

**Indirectness:** *Not serious:* trial(s) were conducted in different countries or settings. *Serious:* trial(s) were conducted from a single country/setting. *Very serious:* evidence is not directly related to PICO question.

**Imprecision:** *Not serious:* Optimal Information Size (OIS) was reached (i.e., sample sizes with at least 200 participants per group may provide prognostic balance); and the entire confidence interval lies on one side of the threshold that may be considered clinically important (≥10% scale range or SMD ≥0.2 for continuous variables, ≥10% for binary variables), such that the clinical course of action would not differ if the upper versus the lower boundary of the confidence interval represented the truth. *Serious:* OIS would not have been reached (sample sizes with less than 200 participants per group); if the OIS was reached, the clinical course of action might differ if the upper versus the lower boundary of the confidence interval represented the truth. *Very serious:* similar to ‘serious’ but to a greater extent (e.g., very small sample sizes and confidence intervals crossing appreciable benefit and harm).

**Other considerations:** *Not serious:* Publication bias is undetected. *Serious/very serious:* Publication bias is strongly suspected.

#### Explanations

a. Rahbar 2018: participants had individual supervised exercise; 15 15-minute sessions.

b. Risk of bias: We downgraded once due to potential risk of bias (performance and detection domains).

c. Inconsistency: We did not downgrade; however, there are no other trials with which to compare findings.

d. Indirectness: We downgraded once. The trial was conducted in one country (low or lower-middle income).

e. Imprecision: We downgraded twice. The sample size was small (OIS would not have been achieved).

#### References

1.Rahbar M, Salekzamani Y, Jahanjou F, Eslamian F, Niroumand A, Dolatkhah N. Effect of hippotherapy simulator on pain, disability, and range of motion of the spinal column in subjects with mechanical low back pain: A randomized single-blind clinical trial. 2018.

GRADE Evidence Profile table 3: ***What are the benefits and harms of muscle strength training in the management of community-dwelling adults (including older adults aged 60 years and over) with chronic primary low back pain (with or without leg pain) compared with no treatment/no additional treatment?***

| **Certainty assessment** | | | | | | | **№ of patients** | | **Effect** | | **Certainty** | **Importance** |
| --- | --- | --- | --- | --- | --- | --- | --- | --- | --- | --- | --- | --- |
| **№ of studies** | **Study design** | **Risk of bias** | **Inconsistency** | **Indirectness** | **Imprecision** | **Other considerations** | **Muscle strength training** | **No intervention** | **Relative (95% CI)** | **Absolute (95% CI)** |
| **ALL ADULTS** | | | | | | | | | | | | |
| **Pain (no leg pain) (follow-up: closest to 2 weeks; assessed with: VAS; benefit indicated by lower values; Scale from: 0 to 10)** | | | | | | | | | | | | |
| 2^1,2,a,b^ | randomised trials | serious^c^ | not serious^d^ | not serious^e^ | very serious^f^ | none | 50 | 50 | - | MD **0.39 lower** (1.16 lower to 0.38 higher) | ⨁◯◯◯ Very low | CRITICAL |
| **Pain in adults in high to upper-middle income countries (follow-up: closest to 2 weeks; assessed with: VAS; benefit indicated by lower values; Scale from: 0 to 10)** | | | | | | | | | | | | |
| 1^1,a^ | randomised trials | serious^c^ | not serious^g^ | serious^h^ | very serious^f^ | none | 35 | 35 | - | MD **0.6 lower** (1.59 lower to 0.39 higher) | ⨁◯◯◯ Very low | CRITICAL |
| **Pain in adults in low to lower-middle income countries (follow-up: closest to 2 weeks; assessed with: VAS; benefit indicated by lower values; Scale from: 0 to 10)** | | | | | | | | | | | | |
| 1^2,b^ | randomised trials | serious^c^ | not serious^g^ | serious^i^ | very serious^f^ | none | 15 | 15 | - | MD **0.07 lower** (1.29 lower to 1.15 higher) | ⨁◯◯◯ Very low | CRITICAL |
| **Pain (no leg pain, high-income country) (follow-up: closest to 6 months; assessed with: VAS; benefit indicated by lower values; Scale from: 0 to 10)** | | | | | | | | | | | | |
| 1^1,a^ | randomised trials | serious^c^ | not serious^g^ | serious^h^ | very serious^f^ | none | 35 | 35 | - | MD **0.4 lower** (1.67 lower to 0.87 higher) | ⨁◯◯◯ Very low | CRITICAL |
| **Pain (no leg pain, high-income country) (follow-up: closest to 12 months; assessed with: VAS; benefit indicated by lower values; Scale from: 0 to 10)** | | | | | | | | | | | | |
| 1^1,a^ | randomised trials | serious^c^ | not serious^g^ | serious^h^ | very serious^f^ | none | 35 | 35 | - | MD **0.1 lower** (1.32 lower to 1.12 higher) | ⨁◯◯◯ Very low | CRITICAL |
| **Function (no leg pain) (follow-up: closest to 2 weeks; assessed with: RMDQ, ODI; benefit indicated by lower values)** | | | | | | | | | | | | |
| 2^1,2,a,b^ | randomised trials | serious^c^ | not serious^d^ | not serious^e^ | very serious^f^ | none | 50 | 50 | - | SMD **0.05 higher** (0.34 lower to 0.45 higher) | ⨁◯◯◯ Very low | CRITICAL |
| **Function in adults in high to upper-middle income countries (follow-up: closest to 2 weeks; assessed with: RMDQ; benefit indicated by lower values)** | | | | | | | | | | | | |
| 1^1,a^ | randomised trials | serious^c^ | not serious^g^ | serious^h^ | very serious^f^ | none | 35 | 35 | - | SMD **0.05 SD higher** (0.42 lower to 0.52 higher) | ⨁◯◯◯ Very low | CRITICAL |
| **Function in adults in low to lower-middle income countries (follow-up: closest to 2 weeks; assessed with: ODI; benefit indicated by lower values)** | | | | | | | | | | | | |
| 1^2,b^ | randomised trials | serious^c^ | not serious^g^ | serious^i^ | very serious^f^ | none | 15 | 15 | - | SMD **0.06 SD higher** (0.65 lower to 0.78 higher) | ⨁◯◯◯ Very low | CRITICAL |
| **Function (no leg pain, high-income country) (follow-up: closest to 6 months; assessed with: RMDQ; benefit indicated by lower values)** | | | | | | | | | | | | |
| 1^1,a^ | randomised trials | serious^c^ | not serious^g^ | serious^h^ | very serious^f^ | none | 35 | 35 | - | MD **0.6 lower** (3.2 lower to 2 higher) | ⨁◯◯◯ Very low | CRITICAL |
| **Function (no leg pain, high income country) (follow-up: closest to 12 months; assessed with: RMDQ; benefit indicated by lower values)** | | | | | | | | | | | | |
| 1^1,a^ | randomised trials | serious^c^ | not serious^g^ | serious^h^ | very serious^f^ | none | 35 | 35 | - | MD **0.2 lower** (2.73 lower to 2.33 higher) | ⨁◯◯◯ Very low | CRITICAL |

**CI:** confidence interval; **MD:** mean difference; **ODI:** Oswestry Disability Index; **SMD:** standardized mean difference; **RMDQ:** Roland-Morris Disability Questionnaire; **VAS:** visual analogue scale

The following was used to guide the ratings.

**Risk of bias:** *Not serious:* trial(s) are rated as overall low risk of bias. *Serious:* some of the weight (>50%) comes from overall unclear risk of bias trial(s).

**Inconsistency:** *Not serious:* high extent of similarity of point estimates and overlap of confidence intervals; statistical heterogeneity (I^2^) is between 0% and 40%, which might not be important. *Serious:* some extent of similarity of point estimates and overlap of confidence intervals; statistical heterogeneity (I^2^) is between 30% and 60%, which could not be explained due to small subgroups and may represent moderate heterogeneity. *Very serious:* little or no similarity of point estimates and overlap of confidence intervals; statistical heterogeneity (I^2^) is between 50% and 90% or 75% and 100%, which could not be explained due to small subgroups and may represent substantial or considerable heterogeneity, respectively.

**Indirectness:** *Not serious:* trial(s) were conducted in different countries or settings. *Serious:* trial(s) were conducted from a single country/setting. *Very serious:* evidence is not directly related to PICO question.

**Imprecision:** *Not serious:* Optimal Information Size (OIS) was reached (i.e., sample sizes with at least 200 participants per group may provide prognostic balance); and the entire confidence interval lies on one side of the threshold that may be considered clinically important (≥10% scale range or SMD ≥0.2 for continuous variables, ≥10% for binary variables), such that the clinical course of action would not differ if the upper versus the lower boundary of the confidence interval represented the truth. *Serious:* OIS would not have been reached (sample sizes with less than 200 participants per group); if the OIS was reached, the clinical course of action might differ if the upper versus the lower boundary of the confidence interval represented the truth. *Very serious:* similar to ‘serious’ but to a greater extent (e.g., very small sample sizes and confidence intervals crossing appreciable benefit and harm).

**Other considerations:** *Not serious:* Publication bias is undetected. *Serious/very serious:* Publication bias is strongly suspected.

#### Explanations

a. Fukuda 2021: participants had individual supervised exercise; 10 45-minute sessions.

b. Shamsi 2022: participants had individual supervised exercise; 12 sessions (session duration not reported).

c. Risk of bias: We downgraded once due to potential risk of bias (performance and detection domains).

d. Inconsistency: We did not downgrade. The point estimates are in the same direction with overlapping confidence intervals. Statistical heterogeneity is between 0% and 40%, which might not be important (i.e., I2 = 0%).

e. Indirectness: We did not downgrade. The trials were conducted in different countries (high- and low-income).

f. Imprecision: We downgraded twice. Sample size was small (OIS would not have been reached).

g. Inconsistency: We did not downgrade; however, there are no other trials with which to compare findings.

h. Indirectness: We downgraded once. The trial was conducted in one country (high or upper-middle income).

i. Indirectness: We downgraded once. The trial was conducted in one country (low or lower-middle income).

#### References

1.Fukuda TY, Aquino LM, Pereira P, et al. Does adding hip strengthening exercises to manual therapy and segmental stabilization improve outcomes in patients with nonspecific low back pain? A randomized controlled trial. 2021.

2.Shamsi M, Ahmadi A, Mirzaei M, Jaberzadeh S. Effects of static stretching and strengthening exercises on flexion relaxation ratio in patients with LBP: A randomized clinical trial. 2022.

GRADE Evidence Profile table 4: ***What are the benefits and harms of mixed exercise in the management of community-dwelling adults (including older adults aged 60 years and over) with chronic primary low back pain (with or without leg pain) compared with no treatment/no additional treatment?***

| **Certainty assessment** | | | | | | | **№ of patients** | | **Effect** | | **Certainty** | **Importance** |
| --- | --- | --- | --- | --- | --- | --- | --- | --- | --- | --- | --- | --- |
| **№ of studies** | **Study design** | **Risk of bias** | **Inconsistency** | **Indirectness** | **Imprecision** | **Other considerations** | **Mixed exercise** | **No intervention** | **Relative (95% CI)** | **Absolute (95% CI)** |
| **ALL ADULTS** | | | | | | | | | | | | |
| **Pain (adults in high to upper-middle income countries) (follow-up: closest to 2 weeks; assessed with: VAS, MPQ; benefit indicated by lower values)** | | | | | | | | | | | | |
| 2^1,2,a,b,c^ | randomised trials | serious^d^ | not serious^e^ | not serious^f^ | very serious^g^ | none | 126 | 124 | - | SMD **0.01 lower** (0.32 lower to 0.31 higher) | ⨁◯◯◯ Very low | CRITICAL |
| **Pain in adults with unclassified presence of leg pain (follow-up: closest to 2 weeks; assessed with: VAS; benefit indicated by lower values)** | | | | | | | | | | | | |
| 1^1,a^ | randomised trials | serious^d^ | not serious^h^ | serious^i^ | very serious^j^ | none | 26 | 24 | - | SMD **0.3 higher** (0.26 lower to 0.86 higher) | ⨁◯◯◯ Very low | CRITICAL |
| **Pain in adults either with or without leg pain (follow-up: closest to 2 weeks; assessed with: MPQ; benefit indicated by lower values)** | | | | | | | | | | | | |
| 1^2,b,c^ | randomised trials | serious^d^ | not serious^h^ | serious^i^ | very serious^g^ | none | 100 | 100 | - | SMD **0.1 lower** (0.44 lower to 0.23 higher) | ⨁◯◯◯ Very low | CRITICAL |
|  | | | | | | | | | | | | |
| **Pain (unclassified presence of leg pain, high to upper-middle income country) (follow-up: closest to 3 months; assessed with: VAS; benefit indicated by lower values; Scale from: 0 to 10)** | | | | | | | | | | | | |
| 1^1,a^ | randomised trials | serious^d^ | not serious^h^ | serious^i^ | very serious^j^ | none | 26 | 24 | - | MD **0.1 lower** (1.34 lower to 1.14 higher) | ⨁◯◯◯ Very low | CRITICAL |
| **Pain (adults in high to upper-middle income countries) (follow-up: closest to 6 months; assessed with: VAS, MPQ; benefit indicated by lower values)** | | | | | | | | | | | | |
| 2^1,2,a,b,k^ | randomised trials | serious^d^ | not serious^l^ | not serious^f^ | very serious^g^ | none | 126 | 122 | - | SMD **0.03 higher** (0.23 lower to 0.29 higher) | ⨁◯◯◯ Very low | CRITICAL |
| **Pain in adults with unclassified presence of leg pain (follow-up: closest to 6 months; assessed with: VAS; benefit indicated by lower values)** | | | | | | | | | | | | |
| 1^1,a^ | randomised trials | serious^d^ | not serious^h^ | serious^i^ | very serious^j^ | none | 26 | 22 | - | SMD **0.13 higher** (0.44 lower to 0.7 higher) | ⨁◯◯◯ Very low | CRITICAL |
| **Pain in adults either with or without leg pain (follow-up: closest to 6 months; assessed with: MPQ; benefit indicated by lower values)** | | | | | | | | | | | | |
| 1^2,b,k^ | randomised trials | serious^d^ | not serious^h^ | serious^i^ | very serious^g^ | none | 100 | 100 | - | SMD **0.01 higher** (0.39 lower to 0.4 higher) | ⨁◯◯◯ Very low | CRITICAL |
| **Pain (either with or without non-radicular leg pain, high to upper-middle income country) (follow-up: closest to 12 months; assessed with: VAS, 0 to 100; benefit indicated by lower values)** | | | | | | | | | | | | |
| 1^3,m^ | randomised trials | not serious^n^ | not serious^h^ | serious^i^ | very serious^j^ | none | Between-group (graded activity with problem solving training alone vs. combination treatment) MD = 8.88, 95% CI -0.36 to 18.13 (119 participants total). | | | | ⨁◯◯◯ Very low | CRITICAL |
| **Function (adults in high to upper-middle income countries) (follow-up: closest to 2 weeks; assessed with: RMDQ, Waddell Disability Index; benefit indicated by lower values)** | | | | | | | | | | | | |
| 2^1,2,a,b,o^ | randomised trials | serious^d^ | not serious^p^ | not serious^f^ | serious^q^ | none | 124 | 126 | - | SMD **0.15 lower** (0.48 lower to 0.18 higher) | ⨁⨁◯◯ Low | CRITICAL |
| **Function in adults with unclassified presence of leg pain (follow-up: closest to 2 weeks; assessed with: Waddell Disability Index; benefit indicated by lower values)** | | | | | | | | | | | | |
| 1^1,a^ | randomised trials | serious^d^ | not serious^h^ | serious^i^ | very serious^j^ | none | 24 | 26 | - | SMD **0.58 lower** (1.15 lower to 0.02 lower) | ⨁◯◯◯ Very low | CRITICAL |
| **Function in adults either with or without leg pain (follow-up: closest to 2 weeks; assessed with: RMDQ; benefit indicated by lower values)** | | | | | | | | | | | | |
| 1^2,b,o^ | randomised trials | serious^d^ | not serious^h^ | serious^i^ | very serious^g^ | none | 100 | 100 | - | SMD **0.01 lower** (0.29 lower to 0.27 higher) | ⨁◯◯◯ Very low | CRITICAL |
| **Function (unclassified presence of leg pain, high to upper-middle income country) (follow-up: closest to 3 months; assessed with: Waddell Disability Index; benefit indicated by lower values; Scale from: 0 to 9)** | | | | | | | | | | | | |
| 1^1,a^ | randomised trials | serious^d^ | not serious^h^ | serious^i^ | very serious^j^ | none | 24 | 26 | - | MD **1.25 lower** (2.79 lower to 0.29 higher) | ⨁◯◯◯ Very low | CRITICAL |
| **Function (adults in high to upper-middle income countries) (follow-up: closest to 6 months; assessed with: RMDQ, Waddell Disability Index; benefit indicated by lower values)** | | | | | | | | | | | | |
| 2^1,2,a,b,r^ | randomised trials | serious^d^ | not serious^p^ | not serious^f^ | very serious^g^ | none | 122 | 126 | - | SMD **0.09 lower** (0.42 lower to 0.24 higher) | ⨁◯◯◯ Very low | CRITICAL |
| **Function in adults with unclassified presence of leg pain (follow-up: closest to 6 months; assessed with: Waddell Disability Index; benefit indicated by lower values)** | | | | | | | | | | | | |
| 1^1,a^ | randomised trials | serious^d^ | not serious^h^ | serious^i^ | very serious^j^ | none | 22 | 26 | - | SMD **0.51 lower** (1.09 lower to 0.07 higher) | ⨁◯◯◯ Very low | CRITICAL |
| **Function in adults either with or without leg pain (follow-up: closest to 6 months; assessed with: RMDQ; benefit indicated by lower values)** | | | | | | | | | | | | |
| 1^2,b,r^ | randomised trials | serious^d^ | not serious^h^ | serious^i^ | very serious^g^ | none | 100 | 100 | - | SMD **0.03 higher** (0.24 lower to 0.31 higher) | ⨁◯◯◯ Very low | CRITICAL |
| **Function (either with or without non-radicular leg pain, high to upper-middle income country) (follow-up: closest to 12 months; assessed with: RMDQ, 0 to 24; benefit indicated by lower values)** | | | | | | | | | | | | |
| 1^3,m^ | randomised trials | not serious^n^ | not serious^h^ | serious^i^ | very serious^j^ | none | Between-group (graded activity with problem solving training alone vs. combination treatment) MD = 1.62, 95% CI -0.06 to 3.31 (119 participants total). | | | | ⨁◯◯◯ Very low | CRITICAL |
| **Health-related quality of life (unclassified presence of leg pain, high to upper-middle income country) (follow-up: closest to 2 weeks; assessed with: Health Assessment Questionnaire; benefit indicated by higher values; Scale from: 0 to 3)** | | | | | | | | | | | | |
| 1^1,a^ | randomised trials | serious^d^ | not serious^h^ | serious^i^ | very serious^j^ | none | 26 | 24 | - | MD **0.24 higher** (0.06 lower to 0.54 higher) | ⨁◯◯◯ Very low | CRITICAL |
| **Health-related quality of life (either with or without leg pain, high to upper-middle income country) (follow-up: closest to 2 weeks; assessed with: SF-36 (PCS); benefit indicated by higher values; Scale from: 0 to 100)** | | | | | | | | | | | | |
| 1^2,b^ | randomised trials | serious^d^ | not serious^h^ | serious^i^ | serious^q^ | none | 100 | 100 | - | MD **6.56 lower** (13.03 lower to 0.1 lower) | ⨁◯◯◯ Very low | CRITICAL |
| **Health-related quality of life (either with or without leg pain, high to upper-middle income country) (follow-up: closest to 2 weeks; assessed with: SF-36 (MCS); benefit indicated by higher values; Scale from: 0 to 100)** | | | | | | | | | | | | |
| 1^2,b^ | randomised trials | serious^d^ | not serious^h^ | serious^i^ | serious^q^ | none | 100 | 100 | - | MD **1.05 lower** (4.38 lower to 2.28 higher) | ⨁◯◯◯ Very low | CRITICAL |
| **Health-related quality of life (unclassified presence of leg pain, high to upper-middle income country) (follow-up: closest to 3 months; assessed with: Health Assessment Questionnaire; benefit indicated by higher values; Scale from: 0 to 3)** | | | | | | | | | | | | |
| 1^1,a^ | randomised trials | serious^d^ | not serious^h^ | serious^i^ | very serious^j^ | none | 26 | 24 | - | MD **0.17 higher** (0.07 lower to 0.41 higher) | ⨁◯◯◯ Very low | CRITICAL |
| **Health-related quality of life (unclassified presence of leg pain, high to upper-middle income country) (follow-up: closest to 6 months; assessed with: Health Assessment Questionnaire; benefit indicated by higher values; Scale from: 0 to 3)** | | | | | | | | | | | | |
| 1^1,a^ | randomised trials | serious^d^ | not serious^h^ | serious^i^ | very serious^j^ | none | 26 | 22 | - | MD **0.19 higher** (0.09 lower to 0.47 higher) | ⨁◯◯◯ Very low | CRITICAL |
| **Health-related quality of life (either with or without leg pain, high to upper-middle income country) (follow-up: closest to 6 months; assessed with: SF-36 (PCS); benefit indicated by higher values; Scale from: 0 to 100)** | | | | | | | | | | | | |
| 1^2,b^ | randomised trials | serious^d^ | not serious^h^ | serious^i^ | serious^q^ | none | 100 | 100 | - | MD **2.31 lower** (9.33 lower to 4.7 higher) | ⨁◯◯◯ Very low | CRITICAL |
| **Health-related quality of life (either with or without leg pain, high to upper-middle income country) (follow-up: closest to 6 months; assessed with: SF-36 (MCS); benefit indicated by higher values; Scale from: 0 to 100)** | | | | | | | | | | | | |
| 1^2,b^ | randomised trials | serious^d^ | not serious^h^ | serious^i^ | serious^q^ | none | 100 | 100 | - | MD **0.83 lower** (8.67 lower to 7 higher) | ⨁◯◯◯ Very low | CRITICAL |
| **Depression (either with or without leg pain, high to upper-middle income country) (follow-up: closest to 2 weeks; assessed with: Geriatric Depression Scale; benefit indicated by lower values; Scale from: 0 to 30)** | | | | | | | | | | | | |
| 1^2,b,s^ | randomised trials | serious^d^ | not serious^h^ | serious^i^ | serious^q^ | none | 100 | 100 | - | MD **0.11 lower** (1.87 lower to 1.66 higher) | ⨁◯◯◯ Very low | CRITICAL |
| **Depression (either with or without leg pain, high to upper-middle income country) (follow-up: closest to 6 months; assessed with: Geriatric Depression Scale; benefit indicated by lower values; Scale from: 0 to 30)** | | | | | | | | | | | | |
| 1^2,b,t^ | randomised trials | serious^d^ | not serious^h^ | serious^i^ | serious^q^ | none | 100 | 100 | - | MD **0.14 higher** (1.92 lower to 2.2 higher) | ⨁◯◯◯ Very low | CRITICAL |
| **Depression (either with or without non-radicular leg pain, high to upper-middle income country) (follow-up: closest to 12 months; assessed with: BDI, 0-63; benefit indicated by lower values)** | | | | | | | | | | | | |
| 1^3,m^ | randomised trials | not serious^n^ | not serious^h^ | serious^i^ | very serious^j^ | none | Between-group (graded activity with problem solving training alone vs. combination treatment) MD = -0.09, 95% CI -2.11 to 1.93 (119 participants total). | | | | ⨁◯◯◯ Very low | CRITICAL |
| **Self-efficacy (either with or without leg pain, high to upper-middle income country) (follow-up: closest to 2 weeks; assessed with: Chronic Pain Self-Efficacy Scale, 10-100; benefit indicated by higher values)** | | | | | | | | | | | | |
| 1^2,u^ | randomised trials | serious^d^ | not serious^h^ | serious^i^ | serious^q^ | none | Between-group change score: 2.1 (SE 3.1), p=0.50 (no significant difference between groups) (200 participants total). | | | | ⨁◯◯◯ Very low | CRITICAL |
| **Self-efficacy (either with or without leg pain, high to upper-middle income country) (follow-up: closest to 6 months; assessed with: Chronic Pain Self-Efficacy Scale, 10-100; benefit indicated by higher values)** | | | | | | | | | | | | |
| 1^2,u^ | randomised trials | serious^d^ | not serious^h^ | serious^i^ | serious^q^ | none | Between-group change score: -0.8 (SE 3.2), p=0.80 (no significant difference between groups) (200 participants total). | | | | ⨁◯◯◯ Very low | CRITICAL |
| **Catastrophizing (either with or without leg pain, high to upper-middle income country) (follow-up: closest to 2 weeks; assessed with: Coping Strategies Questionnaire - Catastrophizing, 0-6; benefit indicated by lower values)** | | | | | | | | | | | | |
| 1^2,u^ | randomised trials | serious^d^ | not serious^h^ | serious^i^ | serious^q^ | none | Between-group change score: -0.1 (SE 0.2), p=0.35 (no significant difference between groups) (200 participants total). | | | | ⨁◯◯◯ Very low | CRITICAL |
| **Catastrophizing (either with or without leg pain, high to upper-middle income country) (follow-up: closest to 6 months; assessed with: Coping Strategies Questionnaire - Catastrophizing, 0-6; benefit indicated by lower values)** | | | | | | | | | | | | |
| 1^2,u^ | randomised trials | serious^d^ | not serious^h^ | serious^i^ | serious^q^ | none | Between-group change score: -0.2 (SE 0.2), p=0.14 (no significant difference between groups) (200 participants total). | | | | ⨁◯◯◯ Very low | CRITICAL |
| **Fear avoidance (either with or without leg pain, high to upper-middle income country) (follow-up: closest to 2 weeks; assessed with: FABQ-PA, 0-24; benefit indicated by lower values)** | | | | | | | | | | | | |
| 1^2,u^ | randomised trials | serious^d^ | not serious^h^ | serious^i^ | serious^q^ | none | Clinically unimportant but statistically significant difference in between-group change scores (comparison 1: -0.6 SE 0.3; p=0.0426; ES=0.37; comparison 2: -0.9 SE 0.3; p=0.0039; ES=0.54) favouring mixed exercise (200 participants total). | | | | ⨁◯◯◯ Very low | CRITICAL |
| **Fear avoidance (either with or without leg pain, high to upper-middle income country) (follow-up: closest to 6 months; assessed with: FABQ-PA, 0-24; benefit indicated by lower values)** | | | | | | | | | | | | |
| 1^2,u^ | randomised trials | serious^d^ | not serious^h^ | serious^i^ | serious^q^ | none | Between-group change score: -0.3 (SE 0.3), p=0.38 (no significant difference between groups) (200 participants total). | | | | ⨁◯◯◯ Very low | CRITICAL |
|  | | | | | | | | | | | | |
| **Adverse events/harms (either with or without leg pain, high to upper-middle income country)** | | | | | | | | | | | | |
| 2^2,3,b,v,w^ | randomised trials | serious^d^ | not serious^x^ | not serious^f^ | very serious^y^ | none | 5/155 (3.2%) | 0/155 (0.0%) | **OR 4.24** (0.69 to 25.95) | **0 fewer per 1,000** (from 0 fewer to 0 fewer) | ⨁◯◯◯ Very low | CRITICAL |
| **OLDER ADULTS (aged 60 years or more)** | | | | | | | | | | | | |
| **Pain (either with or without leg pain, high to upper-middle income country) (follow-up: closest to 2 weeks; assessed with: MPQ; benefit indicated by lower values)** | | | | | | | | | | | | |
| 1^2,b^ | randomised trials | serious^d^ | not serious^h^ | serious^i^ | very serious^g^ | none | 100 | 100 | - | SMD **0.1 lower** (0.44 lower to 0.23 higher) | ⨁◯◯◯ Very low | CRITICAL |
| **Pain (either with or without leg pain, high to upper-middle income country) (follow-up: closest to 6 months; assessed with: MPQ; benefit indicated by lower values)** | | | | | | | | | | | | |
| 1^2,b^ | randomised trials | serious^d^ | not serious^h^ | serious^i^ | very serious^g^ | none | 100 | 100 | - | SMD **0.01 higher** (0.39 lower to 0.4 higher) | ⨁◯◯◯ Very low | CRITICAL |
| **Function (either with or without leg pain, high to upper-middle income country) (follow-up: closest to 2 weeks; assessed with: RMDQ; benefit indicated by lower values)** | | | | | | | | | | | | |
| 1^2,b^ | randomised trials | serious^d^ | not serious^h^ | serious^i^ | very serious^g^ | none | 100 | 100 | - | SMD **0.01 lower** (0.29 lower to 0.27 higher) | ⨁◯◯◯ Very low | CRITICAL |
| **Function (either with or without leg pain, high to upper-middle income country) (follow-up: closest to 6 months; assessed with: RMDQ; benefit indicated by lower values)** | | | | | | | | | | | | |
| 1^2,b^ | randomised trials | serious^d^ | not serious^h^ | serious^i^ | very serious^g^ | none | 100 | 100 | - | SMD **0.03 higher** (0.24 lower to 0.31 higher) | ⨁◯◯◯ Very low | CRITICAL |
| **Health-related quality of life (either with or without leg pain, high to upper-middle income country) (follow-up: closest to 2 weeks; assessed with: SF-36 (PCS); benefit indicated by higher values; Scale from: 0 to 100)** | | | | | | | | | | | | |
| 1^2,b^ | randomised trials | serious^d^ | not serious^h^ | serious^i^ | serious^q^ | none | 100 | 100 | - | MD **6.56 lower** (13.03 lower to 0.1 lower) | ⨁◯◯◯ Very low | CRITICAL |
| **Health-related quality of life (either with or without leg pain, high to upper-middle income country) (follow-up: closest to 2 weeks; assessed with: SF-36 (MCS); benefit indicated by higher values; Scale from: 0 to 100)** | | | | | | | | | | | | |
| 1^2,b^ | randomised trials | serious^d^ | not serious^h^ | serious^i^ | serious^q^ | none | 100 | 100 | - | MD **1.05 lower** (4.38 lower to 2.28 higher) | ⨁◯◯◯ Very low | CRITICAL |
| **Health-related quality of life (either with or without leg pain, high to upper-middle income country) (follow-up: closest to 6 months; assessed with: SF-36 (PCS); benefit indicated by higher values; Scale from: 0 to 100)** | | | | | | | | | | | | |
| 1^2,b^ | randomised trials | serious^d^ | not serious^h^ | serious^i^ | serious^q^ | none | 100 | 100 | - | MD **2.31 lower** (9.33 lower to 4.7 higher) | ⨁◯◯◯ Very low | CRITICAL |
| **Health-related quality of life (either with or without leg pain, high to upper-middle income country) (follow-up: closest to 6 months; assessed with: SF-36 (MCS); benefit indicated by higher values; Scale from: 0 to 100)** | | | | | | | | | | | | |
| 1^2,b^ | randomised trials | serious^d^ | not serious^h^ | serious^i^ | serious^q^ | none | 100 | 100 | - | MD **0.83 lower** (8.67 lower to 7 higher) | ⨁◯◯◯ Very low | CRITICAL |
| **Depression (either with or without leg pain, high to upper-middle income country) (follow-up: closest to 2 weeks; assessed with: Geriatric Depression Scale; benefit indicated by lower values; Scale from: 0 to 30)** | | | | | | | | | | | | |
| 1^2,b^ | randomised trials | serious^d^ | not serious^h^ | serious^i^ | serious^q^ | none | 100 | 100 | - | MD **0.11 lower** (1.87 lower to 1.66 higher) | ⨁◯◯◯ Very low | CRITICAL |
| **Depression (either with or without leg pain, high to upper-middle income country) (follow-up: closest to 6 months; assessed with: Geriatric Depression Scale; benefit indicated by lower values; Scale from: 0 to 30)** | | | | | | | | | | | | |
| 1^2,b^ | randomised trials | serious^d^ | not serious^h^ | serious^i^ | serious^q^ | none | 100 | 100 | - | MD **0.14 higher** (1.92 lower to 2.2 higher) | ⨁◯◯◯ Very low | CRITICAL |
| **Self-efficacy (either with or without leg pain, high to upper-middle income country) (follow-up: closest to 2 weeks; assessed with: Chronic Pain Self-Efficacy Scale, 10-100; benefit indicated by higher values)** | | | | | | | | | | | | |
| 1^2,u^ | randomised trials | serious^d^ | not serious^h^ | serious^i^ | serious^q^ | none | Between-group change score: 2.1 (SE 3.1), p=0.50 (no significant difference between groups) (200 participants total). | | | | ⨁◯◯◯ Very low | CRITICAL |
| **Self-efficacy (either with or without leg pain, high to upper-middle income country) (follow-up: closest to 6 months; assessed with: Chronic Pain Self-Efficacy Scale, 10-100; benefit indicated by higher values)** | | | | | | | | | | | | |
| 1^2,u^ | randomised trials | serious^d^ | not serious^h^ | serious^i^ | serious^q^ | none | Between-group change score: -0.8 (SE 3.2), p=0.80 (no significant difference between groups) (200 participants total). | | | | ⨁◯◯◯ Very low | CRITICAL |
| **Catastrophizing (either with or without leg pain, high to upper-middle income country) (follow-up: closest to 2 weeks; assessed with: Coping Strategies Questionnaire - Catastrophizing, 0-6; benefit indicated by lower values)** | | | | | | | | | | | | |
| 1^2,u^ | randomised trials | serious^d^ | not serious^h^ | serious^i^ | serious^q^ | none | Between-group change score: -0.1 (SE 0.2), p=0.35 (no significant difference between groups) (200 participants total). | | | | ⨁◯◯◯ Very low | CRITICAL |
| **Catastrophizing (either with or without leg pain, high to upper-middle income country) (follow-up: closest to 6 months; assessed with: Coping Strategies Questionnaire - Catastrophizing, 0-6; benefit indicated by lower values)** | | | | | | | | | | | | |
| 1^2,u^ | randomised trials | serious^d^ | not serious^h^ | serious^i^ | serious^q^ | none | Between-group change score: -0.2 (SE 0.2), p=0.14 (no significant difference between groups) (200 participants total). | | | | ⨁◯◯◯ Very low | CRITICAL |
| **Fear avoidance (either with or without leg pain, high to upper-middle income country) (follow-up: closest to 2 weeks; assessed with: FABQ-PA, 0-24; benefit indicated by lower values)** | | | | | | | | | | | | |
| 1^2,u^ | randomised trials | serious^d^ | not serious^h^ | serious^i^ | serious^q^ | none | Clinically unimportant but statistically significant difference in between-group change scores (comparison 1: -0.6 SE 0.3; p=0.0426; ES=0.37; comparison 2: -0.9 SE 0.3; p=0.0039; ES=0.54) favouring mixed exercise (200 participants total). | | | | ⨁◯◯◯ Very low | CRITICAL |
| **Fear avoidance (either with or without leg pain, high to upper-middle income country) (follow-up: closest to 6 months; assessed with: FABQ-PA, 0-24; benefit indicated by lower values)** | | | | | | | | | | | | |
| 1^2,u^ | randomised trials | serious^d^ | not serious^h^ | serious^i^ | serious^q^ | none | Between-group change score: -0.3 (SE 0.3), p=0.38 (no significant difference between groups) (200 participants total). | | | | ⨁◯◯◯ Very low | CRITICAL |
| **Performance-based physical functioning (either with or without leg pain, high to upper-middle income country) (follow-up: closest to 2 weeks)** | | | | | | | | | | | | |
| 1^2,u^ | randomised trials | serious^d^ | not serious^h^ | serious^i^ | serious^q^ | none | Between-group change scores (200 participants total):  Usual pace gait speed (25 meters) (meters/second): no significant difference between groups (0.02 (SE 0.01), p=0.29). Chair raise time (5 consecutive raises) (seconds): small but statistically significant difference favouring mixed exercise for comparison 2 (-0.8 (SE 0.3), p=0.008, ES=0.29).  Stair climb time (seconds): no significant difference between groups (-0.0 (SE 1.2), p=0.99). | | | | ⨁◯◯◯ Very low | CRITICAL |
| **Performance-based physical functioning (either with or without leg pain, high to upper-middle income country) (follow-up: closest to 6 months)** | | | | | | | | | | | | |
| 1^2,u^ | randomised trials | serious^d^ | not serious^h^ | serious^i^ | serious^q^ | none | Between-group change scores (200 participants total): Usual pace gait speed (25 meters) (meters/second): no significant difference between groups (0.00 (SE 0.02), p=0.92). Chair raise time (5 consecutive raises) (seconds): no significant difference between groups (0.1 (SE 0.3), p=0.88). Stair climb time (seconds): no significant difference between groups (-0.6 (SE 1.2), p=0.61). | | | | ⨁◯◯◯ Very low | CRITICAL |
| **Adverse events/harms (either with or without leg pain, high to upper-middle income country)** | | | | | | | | | | | | |
| 1^2,b,z^ | randomised trials | serious^d^ | not serious^h^ | serious^i^ | very serious^y^ | none | 2/100 (2.0%) | 0/100 (0.0%) | **OR 3.06** (0.31 to 29.93) | **0 fewer per 1,000** (from 0 fewer to 0 fewer) | ⨁◯◯◯ Very low | CRITICAL |

**CI:** confidence interval; **FABQ-PA:** Fear Avoidance Behaviour Questionnaire – Physical Activity; **MCS:** Mental Component Summary; **MD:** mean difference; **MPQ:** McGill Pain Questionnaire; **n/a:** not applicable; **OR:** odds ratio; **PCS:** Physical Component Summary; **SMD:** standardized mean difference; **RMDQ:** Roland-Morris Disability Questionnaire; **VAS:** visual analogue scale

The following was used to guide the ratings.

**Risk of bias:** *Not serious:* trial(s) are rated as overall low risk of bias. *Serious:* some of the weight (>50%) comes from overall unclear risk of bias trial(s).

**Inconsistency:** *Not serious:* high extent of similarity of point estimates and overlap of confidence intervals; statistical heterogeneity (I^2^) is between 0% and 40%, which might not be important. *Serious:* some extent of similarity of point estimates and overlap of confidence intervals; statistical heterogeneity (I^2^) is between 30% and 60%, which could not be explained due to small subgroups and may represent moderate heterogeneity. *Very serious:* little or no similarity of point estimates and overlap of confidence intervals; statistical heterogeneity (I^2^) is between 50% and 90% or 75% and 100%, which could not be explained due to small subgroups and may represent substantial or considerable heterogeneity, respectively.

**Indirectness:** *Not serious:* trial(s) were conducted in different countries or settings. *Serious:* trial(s) were conducted from a single country/setting. *Very serious:* evidence is not directly related to PICO question.

**Imprecision:** *Not serious:* Optimal Information Size (OIS) was reached (i.e., sample sizes with at least 200 participants per group may provide prognostic balance); and the entire confidence interval lies on one side of the threshold that may be considered clinically important (≥10% scale range or SMD ≥0.2 for continuous variables, ≥10% for binary variables), such that the clinical course of action would not differ if the upper versus the lower boundary of the confidence interval represented the truth. *Serious:* OIS would not have been reached (sample sizes with less than 200 participants per group); if the OIS was reached, the clinical course of action might differ if the upper versus the lower boundary of the confidence interval represented the truth. *Very serious:* similar to ‘serious’ but to a greater extent (e.g., very small sample sizes and confidence intervals crossing appreciable benefit and harm).

**Other considerations:** *Not serious:* Publication bias is undetected. *Serious/very serious:* Publication bias is strongly suspected.

#### Explanations

a. Takinaci 2019: participants had general strength training, stretching, or flexibility/mobilizing exercises; group supervised; 12 sessions (session duration was not reported).

b. Weiner 2008: had 2 comparisons (both included in meta-analysis). Participants had strength, flexibility, aerobic exercises; individual supervision; 12 sessions (session duration was not reported).

c. Smeets 2008 was not included in the meta-analysis (provided within-group mean changes; no follow-up scores). 119 participants total, rated as overall low risk of bias. Participants performed combination treatment (active physical treatment [aerobic and core strengthening exercises] + graded activity with problem solving training) vs. graded activity with problem solving training alone. Intervention was group supervised; 30 105-minute sessions. Between-group MD (VAS 0-100) graded activity with problem solving training alone vs. combination treatment = 5.35, 95% CI -3.73 to 14.42.

d. Risk of bias: We downgraded once due to potential risk of bias (performance and detection domains).

e. Inconsistency: We did not downgrade. The confidence intervals overlapped, and statistical heterogeneity is between 0% and 40%, which might not be important (i.e., I2 = 34%).

f. Indirectness: We did not downgrade because trials were conducted in different countries (high or upper-middle income).

g. Imprecision: We downgraded twice. The point estimate did not reach the pre-specified threshold for what may be considered clinically important (SMD ≥ 0.2). The lower boundary of the 95% CI crosses the threshold for what may be considered appreciable benefit (-0.2), and the upper boundary crosses the threshold for what may be considered appreciable harm (+0.2).

h. Inconsistency: We did not downgrade; however, there are no other trials with which to compare findings

i. Indirectness: We downgraded once. The trial was conducted in one country (high or upper-middle income).

j. Imprecision: We downgraded twice. The sample size was small (OIS would not have been reached).

k. Smeets 2008 was not included in the meta-analysis (provided within-group mean changes; no follow-up scores). 119 participants total, rated as overall low risk of bias. Participants performed combination treatment (active physical treatment [aerobic and core strengthening exercises] + graded activity with problem solving training) vs. graded activity with problem solving training alone. Intervention was group supervised; 30 105-minute sessions. Between-group MD (VAS 0-100) graded activity with problem solving training alone vs. combination treatment = 6.25, 95% CI -2.94 to 15.44.

l. Inconsistency: We did not downgrade. The confidence intervals overlapped, and statistical heterogeneity is between 0% and 40%, which might not be important (i.e., I2 = 6%).

m. Smeets 2008 was not included in the meta-analysis (provided within-group mean changes; no follow-up scores). Participants performed combination treatment (active physical treatment [aerobic and core strengthening exercises] + graded activity with problem solving training) vs. graded activity with problem solving training alone. Intervention was group supervised; 30 105-minute sessions.

n. Risk of bias: We did not downgrade because the trial was rated as overall low risk of bias.

o. Smeets 2008 was not included in the meta-analysis (provided within-group mean changes; no follow-up scores). 119 participants total, rates as overall low risk of bias. Participants performed combination treatment (active physical treatment [aerobic and core strengthening exercises] + graded activity with problem solving training) vs. graded activity with problem solving training alone. Intervention was group supervised; 30 105-minute sessions. Between-group MD (RMDQ 0-24) graded activity with problem solving training alone vs. combination treatment = 0.58, 95% CI -1.08 to 2.24.

p. Inconsistency: We did not downgrade. The confidence intervals overlapped, and statistical heterogeneity is between 0% and 40%, which might not be important (i.e., I2 = 40%).

q. Imprecision: We downgraded once. The sample size was small (OIS would not have been reached).

r. Smeets 2008 was not included in the meta-analysis (provided within-group mean changes; no follow-up scores). 119 participants total, rated as overall low risk of bias. Participants performed combination treatment (active physical treatment [aerobic and core strengthening exercises] + graded activity with problem solving training) vs. graded activity with problem solving training alone. Intervention was group supervised; 30 105-minute sessions. Between-group MD (RMDQ 0-24) graded activity with problem solving training alone vs. combination treatment = 1.11, 95% CI -0.56 to 2.79.

s. Smeets 2008 was not included in the meta-analysis (provided within-group mean changes; no follow-up scores). 119 participants total; rated as overall low risk of bias. Participants performed combination treatment (active physical treatment [aerobic and core strengthening exercises] + graded activity with problem solving training) vs. graded activity with problem solving training alone. Intervention was group supervised; 30 105-minute sessions. Between-group MD (BDI 0-63) graded activity with problem solving training alone vs. combination treatment = 1.62, 95% CI -0.36 to 3.61.

t. Smeets 2008 was not included in the meta-analysis (provided within-group mean changes; no follow-up scores). 119 participants total, rated as overall low risk of bias. Participants performed combination treatment (active physical treatment [aerobic and core strengthening exercises] + graded activity with problem solving training) vs. graded activity with problem solving training alone. Intervention was group supervised; 30 105-minute sessions. Between-group MD (BDI 0-63) graded activity with problem solving training alone vs. combination treatment = 0.26, 95% CI -1.74 to 2.27.

u. Weiner 2008: participants had strength, flexibility, aerobic exercises; individual supervision; 12 sessions (session duration was not reported). Only within and between-group change scores were reported.

v. Smeets 2008: participants performed combination treatment (active physical treatment [aerobic and core strengthening exercises] + graded activity with problem solving training) vs. graded activity with problem solving training alone. Intervention was group supervised; 30 105-minute sessions.

w. Adverse events: were mainly minor and included back and knee pain.

x. Inconsistency: We did not downgrade. The point estimates are similar with overlapping confidence intervals. Statistical heterogeneity is between 0% and 40%, which might not be important (i.e., I2 = 0%).

y. Imprecision: We downgraded twice due to small sample size and number of events.

z. Adverse events: included back pain. Authors reported no significant intervention-associated adverse events.

#### References

1. Z, Takinaci. Clinical efficacy of spa therapy (balneotherapy) for chronic low back pain: A randomized single-blind trial. 2019.

2.Weiner DK, Perera S, Rudy TE, Glick RM, Shenoy S, Delitto A. Efficacy of percutaneous electrical nerve stimulation and therapeutic exercise for older adults with chronic low back pain: a randomized controlled trial. 2008.

3.Smeets R, Vlaeyen JWS, Hidding A, Kester ADM, van der Heijden G, Knottnerus AJ. Chronic low back pain: physical training, graded activity with problem solving training, or both? The one-year post-treatment results of a randomized controlled trial. 2008.

GRADE Evidence Profile table 5: ***What are the benefits and harms of mixed exercise in the management of community-dwelling adults (including older adults aged 60 years and over) with chronic primary low back pain (with or without leg pain) compared with usual care?***

| **Certainty assessment** | | | | | | | **№ of patients** | | **Effect** | | **Certainty** | **Importance** |
| --- | --- | --- | --- | --- | --- | --- | --- | --- | --- | --- | --- | --- |
| **№ of studies** | **Study design** | **Risk of bias** | **Inconsistency** | **Indirectness** | **Imprecision** | **Other considerations** | **Mixed exercise** | **Usual care** | **Relative (95% CI)** | **Absolute (95% CI)** |
| **ALL ADULTS** | | | | | | | | | | | | |
| **Pain (unclassified presence of leg pain) (follow-up: closest to 2 weeks; assessed with: NRS; benefit indicated by lower values; Scale from: 0 to 10)** | | | | | | | | | | | | |
| 2^1,2,a,b^ | randomised trials | serious^c^ | not serious^d^ | not serious^e^ | very serious^f^ | none | 71 | 71 | - | MD **0.12 lower** (0.91 lower to 0.68 higher) | ⨁◯◯◯ Very low | CRITICAL |
| **Pain in adults in high to upper-middle income countries (follow-up: closest to 2 weeks; assessed with: NRS; benefit indicated by lower values; Scale from: 0 to 10)** | | | | | | | | | | | | |
| 1^2,b^ | randomised trials | serious^c^ | not serious^g^ | serious^h^ | very serious^f^ | none | 26 | 23 | - | MD **0.8 lower** (2.42 lower to 0.82 higher) | ⨁◯◯◯ Very low | CRITICAL |
| **Pain in adults in low to lower-middle income countries (follow-up: closest to 2 weeks; assessed with: NRS; benefit indicated by lower values; Scale from: 0 to 10)** | | | | | | | | | | | | |
| 1^1,a^ | randomised trials | serious^c^ | not serious^g^ | serious^i^ | very serious^f^ | none | 45 | 48 | - | MD **0.1 higher** (0.81 lower to 1.01 higher) | ⨁◯◯◯ Very low | CRITICAL |
| **Pain (unclassified presence of leg pain, high to upper-middle income country) (follow-up: closest to 3 months; assessed with: NRS; benefit indicated by lower values; Scale from: 0 to 10)** | | | | | | | | | | | | |
| 1^2,b^ | randomised trials | serious^c^ | not serious^g^ | serious^h^ | very serious^f^ | none | 26 | 22 | - | MD **0.3 lower** (1.66 lower to 1.06 higher) | ⨁◯◯◯ Very low | CRITICAL |
| **Pain (unclassified presence of leg pain, high to upper-middle income country) (follow-up: closest to 6 months; assessed with: NRS; benefit indicated by lower values; Scale from: 0 to 10)** | | | | | | | | | | | | |
| 1^2,b^ | randomised trials | serious^c^ | not serious^g^ | serious^h^ | very serious^f^ | none | 25 | 23 | - | MD **0**  (1.26 lower to 1.26 higher) | ⨁◯◯◯ Very low | CRITICAL |
| **Function (unclassified presence of leg pain) (follow-up: closest to 2 weeks; assessed with: RMDQ, Modified ODI; benefit indicated by lower values)** | | | | | | | | | | | | |
| 2^1,2,a,b^ | randomised trials | serious^c^ | not serious^d^ | not serious^e^ | very serious^f^ | none | 71 | 71 | - | SMD **0.62 lower** (0.96 lower to 0.28 lower) | ⨁◯◯◯ Very low | CRITICAL |
| **Function in adults in high to upper-middle income countries (follow-up: closest to 2 weeks; assessed with: Modified ODI; benefit indicated by lower values)** | | | | | | | | | | | | |
| 1^2,b^ | randomised trials | serious^c^ | not serious^g^ | serious^h^ | very serious^f^ | none | 26 | 23 | - | SMD **0.86 SD lower** (1.45 lower to 0.27 lower) | ⨁◯◯◯ Very low | CRITICAL |
|  | | | | | | | | | | | | |
| **Function in adults in low to lower-middle income countries (follow-up: closest to 2 weeks; assessed with: RMDQ; benefit indicated by lower values)** | | | | | | | | | | | | |
| 1^1,a^ | randomised trials | serious^c^ | not serious^g^ | serious^i^ | very serious^f^ | none | 45 | 48 | - | SMD **0.51 SD lower** (0.92 lower to 0.09 lower) | ⨁◯◯◯ Very low | CRITICAL |
| **Function (unclassified presence of leg pain, high to upper-middle income country) (follow-up: closest to 3 months; assessed with: RMDQ; benefit indicated by lower values; Scale from: 0 to 24)** | | | | | | | | | | | | |
| 1^2,b^ | randomised trials | serious^c^ | not serious^g^ | serious^h^ | very serious^f^ | none | 26 | 22 | - | MD **2.3 lower** (4.92 lower to 0.32 higher) | ⨁◯◯◯ Very low | CRITICAL |
| **Function (unclassified presence of leg pain, high to upper-middle income country) (follow-up: closest to 6 months; assessed with: RMDQ; benefit indicated by lower values; Scale from: 0 to 24)** | | | | | | | | | | | | |
| 1^2,b^ | randomised trials | serious^c^ | not serious^g^ | serious^h^ | very serious^f^ | none | 25 | 23 | - | MD **2.5 lower** (5.19 lower to 0.19 higher) | ⨁◯◯◯ Very low | CRITICAL |
| **Health-related quality of life (unclassified presence of leg pain, high to upper-middle income country) (follow-up: closest to 2 weeks; assessed with: EQ-5D; benefit indicated by higher values; Scale from: 0 to 1)** | | | | | | | | | | | | |
| 1^2,b^ | randomised trials | serious^c^ | not serious^g^ | serious^h^ | very serious^f^ | none | 26 | 23 | - | MD **0.05 higher** (0.01 lower to 0.11 higher) | ⨁◯◯◯ Very low | CRITICAL |
| **Health-related quality of life (unclassified presence of leg pain, high to upper-middle income country) (follow-up: closest to 3 months; assessed with: EQ-5D; benefit indicated by higher values; Scale from: 0 to 1)** | | | | | | | | | | | | |
| 1^2,b^ | randomised trials | serious^c^ | not serious^g^ | serious^h^ | very serious^f^ | none | 26 | 22 | - | MD **0.04 higher** (0 to 0.08 higher) | ⨁◯◯◯ Very low | CRITICAL |
| **Health-related quality of life (unclassified presence of leg pain, high to upper-middle income country) (follow-up: closest to 6 months; assessed with: EQ-5D; benefit indicated by higher values; Scale from: 0 to 1)** | | | | | | | | | | | | |
| 1^2,b^ | randomised trials | serious^c^ | not serious^g^ | serious^h^ | very serious^f^ | none | 25 | 23 | - | MD **0.05 higher** (0 to 0.1 higher) | ⨁◯◯◯ Very low | CRITICAL |
| **Self-efficacy (unclassified presence of leg pain, high to upper-middle income country) (follow-up: closest to 2 weeks; assessed with: PSEQ; benefit indicated by higher values; Scale from: 0 to 60)** | | | | | | | | | | | | |
| 1^2,b^ | randomised trials | serious^c^ | not serious^g^ | serious^h^ | very serious^f^ | none | 26 | 23 | - | MD **3 higher** (2.39 lower to 8.39 higher) | ⨁◯◯◯ Very low | CRITICAL |
| **Self-efficacy (unclassified presence of leg pain, high to upper-middle income country) (follow-up: closest to 3 months; assessed with: PSEQ; benefit indicated by higher values; Scale from: 0 to 60)** | | | | | | | | | | | | |
| 1^2,b^ | randomised trials | serious^c^ | not serious^g^ | serious^h^ | very serious^f^ | none | 26 | 22 | - | MD **3 higher** (1.63 lower to 7.63 higher) | ⨁◯◯◯ Very low | CRITICAL |
| **Self-efficacy (unclassified presence of leg pain, high to upper-middle income country) (follow-up: closest to 6 months; assessed with: PSEQ; benefit indicated by higher values; Scale from: 0 to 60)** | | | | | | | | | | | | |
| 1^2,b^ | randomised trials | serious^c^ | not serious^g^ | serious^h^ | very serious^f^ | none | 25 | 23 | - | MD **4 higher** (3.81 lower to 11.81 higher) | ⨁◯◯◯ Very low | CRITICAL |
| **OLDER ADULTS (60 years old or more)** | | | | | | | | | | | | |
| **Pain (unclassified presence of leg pain, high to upper-middle income country) (follow-up: closest to 2 weeks; assessed with: NRS; benefit indicated by lower values; Scale from: 0 to 10)** | | | | | | | | | | | | |
| 1^2,b^ | randomised trials | serious^c^ | not serious^g^ | serious^h^ | very serious^f^ | none | 26 | 23 | - | MD **0.8 lower** (2.42 lower to 0.82 higher) | ⨁◯◯◯ Very low | CRITICAL |
| **Pain (unclassified presence of leg pain, high to upper-middle income country) (follow-up: closest to 3 months; assessed with: NRS; benefit indicated by lower values; Scale from: 0 to 10)** | | | | | | | | | | | | |
| 1^2,b^ | randomised trials | serious^c^ | not serious^g^ | serious^h^ | very serious^f^ | none | 26 | 22 | - | MD **0.3 lower** (1.66 lower to 1.06 higher) | ⨁◯◯◯ Very low | CRITICAL |
| **Pain (unclassified presence of leg pain, high to upper-middle income country) (follow-up: closest to 6 months; assessed with: NRS; benefit indicated by lower values; Scale from: 0 to 10)** | | | | | | | | | | | | |
| 1^2,b^ | randomised trials | serious^c^ | not serious^g^ | serious^h^ | very serious^f^ | none | 25 | 23 | - | MD **0**  (1.26 lower to 1.26 higher) | ⨁◯◯◯ Very low | CRITICAL |
| **Function (unclassified presence of leg pain, high to upper-middle income country) (follow-up: closest to 2 weeks; assessed with: RMDQ; benefit indicated by lower values)** | | | | | | | | | | | | |
| 1^2,b^ | randomised trials | serious^c^ | not serious^g^ | serious^h^ | very serious^f^ | none | 26 | 23 | - | SMD **0.86 SD lower** (1.45 lower to 0.27 lower) | ⨁◯◯◯ Very low | CRITICAL |
| **Function (unclassified presence of leg pain, high to upper-middle income country) (follow-up: closest to 3 months; assessed with: RMDQ; benefit indicated by lower values; Scale from: 0 to 24)** | | | | | | | | | | | | |
| 1^2,b^ | randomised trials | serious^c^ | not serious^g^ | serious^h^ | very serious^f^ | none | 26 | 22 | - | MD **2.3 lower** (4.92 lower to 0.32 higher) | ⨁◯◯◯ Very low | CRITICAL |
| **Function (unclassified presence of leg pain, high to upper-middle income country) (follow-up: closest to 6 months; assessed with: RMDQ; benefit indicated by lower values; Scale from: 0 to 24)** | | | | | | | | | | | | |
| 1^2,b^ | randomised trials | serious^c^ | not serious^g^ | serious^h^ | very serious^f^ | none | 25 | 23 | - | MD **2.5 lower** (5.19 lower to 0.19 higher) | ⨁◯◯◯ Very low | CRITICAL |
| **Health-related quality of life (unclassified presence of leg pain, high to upper-middle income country) (follow-up: closest to 2 weeks; assessed with: EQ-5D; benefit indicated by higher values; Scale from: 0 to 1)** | | | | | | | | | | | | |
| 1^2,b^ | randomised trials | serious^c^ | not serious^g^ | serious^h^ | very serious^f^ | none | 26 | 23 | - | MD **0.05 higher** (0.01 lower to 0.11 higher) | ⨁◯◯◯ Very low | CRITICAL |
| **Health-related quality of life (unclassified presence of leg pain, high to upper-middle income country) (follow-up: closest to 3 months; assessed with: EQ-5D; benefit indicated by higher values; Scale from: 0 to 1)** | | | | | | | | | | | | |
| 1^2,b^ | randomised trials | serious^c^ | not serious^g^ | serious^h^ | very serious^f^ | none | 26 | 22 | - | MD **0.04 higher** (0 to 0.08 higher) | ⨁◯◯◯ Very low | CRITICAL |
| **Health-related quality of life (unclassified presence of leg pain, high to upper-middle income country) (follow-up: closest to 6 months; assessed with: EQ-5D; benefit indicated by higher values; Scale from: 0 to 1)** | | | | | | | | | | | | |
| 1^2,b^ | randomised trials | serious^c^ | not serious^g^ | serious^h^ | very serious^f^ | none | 25 | 23 | - | MD **0.05 higher** (0 to 0.1 higher) | ⨁◯◯◯ Very low | CRITICAL |
| **Self-efficacy (unclassified presence of leg pain, high to upper-middle income country) (follow-up: closest to 2 weeks; assessed with: PSEQ; benefit indicated by higher values; Scale from: 0 to 60)** | | | | | | | | | | | | |
| 1^2,b^ | randomised trials | serious^c^ | not serious^g^ | serious^h^ | very serious^f^ | none | 26 | 23 | - | MD **3 higher** (2.39 lower to 8.39 higher) | ⨁◯◯◯ Very low | CRITICAL |
| **Self-efficacy (unclassified presence of leg pain, high to upper-middle income country) (follow-up: closest to 3 months; assessed with: PSEQ; benefit indicated by higher values; Scale from: 0 to 60)** | | | | | | | | | | | | |
| 1^2,b^ | randomised trials | serious^c^ | not serious^g^ | serious^h^ | very serious^f^ | none | 26 | 22 | - | MD **3 higher** (1.63 lower to 7.63 higher) | ⨁◯◯◯ Very low | CRITICAL |
| **Self-efficacy (unclassified presence of leg pain, high to upper-middle income country) (follow-up: closest to 6 months; assessed with: PSEQ; benefit indicated by higher values; Scale from: 0 to 60)** | | | | | | | | | | | | |
| 1^2,b^ | randomised trials | serious^c^ | not serious^g^ | serious^h^ | very serious^f^ | none | 25 | 23 | - | MD **4 higher** (3.81 lower to 11.81 higher) | ⨁◯◯◯ Very low | CRITICAL |

**CI:** confidence interval; **EQ-5D:** EuroQoL; **MD:** mean difference; **NRS:** numeric rating scale; **ODI:** Oswestry Disability Index; **PSEQ:** Pain Self-Efficacy Questionnaire; **RMDQ:** Roland-Morris Disability Questionnaire; **SMD:** standardized mean difference

The following was used to guide the ratings.

**Risk of bias:** *Not serious:* trial(s) are rated as overall low risk of bias. *Serious:* some of the weight (>50%) comes from overall unclear risk of bias trial(s).

**Inconsistency:** *Not serious:* high extent of similarity of point estimates and overlap of confidence intervals; statistical heterogeneity (I^2^) is between 0% and 40%, which might not be important. *Serious:* some extent of similarity of point estimates and overlap of confidence intervals; statistical heterogeneity (I^2^) is between 30% and 60%, which could not be explained due to small subgroups and may represent moderate heterogeneity. *Very serious:* little or no similarity of point estimates and overlap of confidence intervals; statistical heterogeneity (I^2^) is between 50% and 90% or 75% and 100%, which could not be explained due to small subgroups and may represent substantial or considerable heterogeneity, respectively.

**Indirectness:** *Not serious:* trial(s) were conducted in different countries or settings. *Serious:* trial(s) were conducted from a single country/setting. *Very serious:* evidence is not directly related to PICO question.

**Imprecision:** *Not serious:* Optimal Information Size (OIS) was reached (i.e., sample sizes with at least 200 participants per group may provide prognostic balance); and the entire confidence interval lies on one side of the threshold that may be considered clinically important (≥10% scale range or SMD ≥0.2 for continuous variables, ≥10% for binary variables), such that the clinical course of action would not differ if the upper versus the lower boundary of the confidence interval represented the truth. *Serious:* OIS would not have been reached (sample sizes with less than 200 participants per group); if the OIS was reached, the clinical course of action might differ if the upper versus the lower boundary of the confidence interval represented the truth. *Very serious:* similar to ‘serious’ but to a greater extent (e.g., very small sample sizes and confidence intervals crossing appreciable benefit and harm).

**Other considerations:** *Not serious:* Publication bias is undetected. *Serious/very serious:* Publication bias is strongly suspected.

#### Explanations

a. Chhabra 2018: participants received home exercise with follow-up

b. Jinnouchi 2020: participants received home exercise with follow-up

c. Risk of bias: We downgraded once due to potential risk of bias (performance and detection domains).

d. Inconsistency: We did not downgrade. The confidence intervals overlap; statistical heterogeneity is between 0% and 40%, which might not be important (i.e., I2 = 0%).

e. Indirectness: We did not downgrade because the trials were conducted in different countries (high and low to lower-middle income).

f. Imprecision: We downgraded twice. The sample size was small (OIS would not have been reached).

g. Inconsistency: We did not downgrade; however, there are no other trials with which to compare findings.

h. Indirectness: We downgraded once. The trial was conducted in one country (high or upper-middle income).

i. Indirectness: We downgraded once. The trial was conducted in one country (low or lower-middle income).

#### References

1.Chhabra HS, Sharma S, Verma S. Smartphone app in self-management of chronic low back pain: a randomized controlled trial. 2018.

2.Jinnouchi H, Matsudaira K, Kitamura A, et al. Effects of brief self-exercise education on the management of chronic low back pain: A community-based, randomized, parallel-group pragmatic trial. 2020.

Grade Evidence Profile table 6: ***What are the benefits and harms of Pilates exercise in the management of community-dwelling adults (including older adults aged 60 years and over) with chronic primary low back pain (with or without leg pain) compared with no treatment/no additional treatment?***

| **Certainty assessment** | | | | | | | **№ of patients** | | **Effect** | | **Certainty** | **Importance** |
| --- | --- | --- | --- | --- | --- | --- | --- | --- | --- | --- | --- | --- |
| **№ of studies** | **Study design** | **Risk of bias** | **Inconsistency** | **Indirectness** | **Imprecision** | **Other considerations** | **Pilates** | **No intervention** | **Relative (95% CI)** | **Absolute (95% CI)** |
| **ALL ADULTS** | | | | | | | | | | | | |
| **Pain (unclassified presence of leg pain, high-income country) (follow-up: closest to 2 weeks; assessed with: NRS; benefit indicated by lower values; Scale from: 0 to 10)** | | | | | | | | | | | | |
| 1^1,a^ | randomised trials | serious^b^ | not serious^c^ | serious^d^ | very serious^e^ | none | 43 | 43 | - | MD **2.1 lower** (3.07 lower to 1.13 lower) | ⨁◯◯◯ Very low | CRITICAL |
| **Pain (unclassified presence of leg pain, high-income country) (follow-up: closest to 6 months; assessed with: NRS; benefit indicated by lower values; Scale from: 0 to 10)** | | | | | | | | | | | | |
| 1^1,a^ | randomised trials | serious^b^ | not serious^c^ | serious^d^ | very serious^e^ | none | 43 | 43 | - | MD **0.8 lower** (1.75 lower to 0.15 higher) | ⨁◯◯◯ Very low | CRITICAL |
| **Function (unclassified presence of leg pain, high-income country) (follow-up: closest to 2 weeks; assessed with: RMDQ; benefit indicated by lower values; Scale from: 0 to 24)** | | | | | | | | | | | | |
| 1^1,a,f^ | randomised trials | serious^b^ | not serious^c^ | serious^d^ | very serious^e^ | none | 43 | 43 | - | MD **3.5 lower** (5.48 lower to 1.52 lower) | ⨁◯◯◯ Very low | CRITICAL |
| **Function (unclassified presence of leg pain, high-income country) (follow-up: closest to 6 months; assessed with: RMDQ; benefit indicated by lower values; Scale from: 0 to 24)** | | | | | | | | | | | | |
| 1^1,a,g^ | randomised trials | serious^b^ | not serious^c^ | serious^d^ | very serious^e^ | none | 43 | 43 | - | MD **2.2 lower** (4.35 lower to 0.05 lower) | ⨁◯◯◯ Very low | CRITICAL |
| **Fear Avoidance (unclassified presence of leg pain, high-income country) (follow-up: closest to 2 weeks; assessed with: TSK; benefit indicated by lower values; Scale from: 17 to 68)** | | | | | | | | | | | | |
| 1^1,a^ | randomised trials | serious^b^ | not serious^c^ | serious^d^ | very serious^e^ | none | 43 | 43 | - | MD **1.8 lower** (5.12 lower to 1.52 higher) | ⨁◯◯◯ Very low | CRITICAL |
| **Fear Avoidance (unclassified presence of leg pain, high-income country) (follow-up: closest to 6 months; assessed with: TSK; benefit indicated by lower values; Scale from: 17 to 68)** | | | | | | | | | | | | |
| 1^1,a^ | randomised trials | serious^b^ | not serious^c^ | serious^d^ | very serious^e^ | none | 43 | 43 | - | MD **0.8 lower** (3.86 lower to 2.26 higher) | ⨁◯◯◯ Very low | CRITICAL |
| **Adverse events/harms (unclassified presence of leg pain, high-income country)** | | | | | | | | | | | | |
| 1^1,a^ | randomised trials | serious^b^ | not serious^c^ | serious^d^ | very serious^e^ | none | Study authors reported that no adverse events occurred (86 participants total). | | | | ⨁◯◯◯ Very low | CRITICAL |

**CI:** confidence interval; **MD:** mean difference; **NRS:** numeric rating scale; **RMDQ:** Roland-Morris Disability Questionnaire; **TSK:** Tampa Scale for Kinesiophobia

The following was used to guide the ratings.

**Risk of bias:** *Not serious:* trial(s) are rated as overall low risk of bias. *Serious:* some of the weight (>50%) comes from overall unclear risk of bias trial(s).

**Inconsistency:** *Not serious:* high extent of similarity of point estimates and overlap of confidence intervals; statistical heterogeneity (I^2^) is between 0% and 40%, which might not be important. *Serious:* some extent of similarity of point estimates and overlap of confidence intervals; statistical heterogeneity (I^2^) is between 30% and 60%, which could not be explained due to small subgroups and may represent moderate heterogeneity. *Very serious:* little or no similarity of point estimates and overlap of confidence intervals; statistical heterogeneity (I^2^) is between 50% and 90% or 75% and 100%, which could not be explained due to small subgroups and may represent substantial or considerable heterogeneity, respectively.

**Indirectness:** *Not serious:* trial(s) were conducted in different countries or settings. *Serious:* trial(s) were conducted from a single country/setting. *Very serious:* evidence is not directly related to PICO question.

**Imprecision:** *Not serious:* Optimal Information Size (OIS) was reached (i.e., sample sizes with at least 200 participants per group may provide prognostic balance); and the entire confidence interval lies on one side of the threshold that may be considered clinically important (≥10% scale range or SMD ≥0.2 for continuous variables, ≥10% for binary variables), such that the clinical course of action would not differ if the upper versus the lower boundary of the confidence interval represented the truth. *Serious:* OIS would not have been reached (sample sizes with less than 200 participants per group); if the OIS was reached, the clinical course of action might differ if the upper versus the lower boundary of the confidence interval represented the truth. *Very serious:* similar to ‘serious’ but to a greater extent (e.g., very small sample sizes and confidence intervals crossing appreciable benefit and harm).

**Other considerations:** *Not serious:* Publication bias is undetected. *Serious/very serious:* Publication bias is strongly suspected.

#### Explanations

a. Miyamoto 2013: participants received individual supervised exercise; 12 60-minute sessions.

b. Risk of bias: We downgraded once due to potential risk of bias (performance domain).

c. Inconsistency: We did not downgrade; however, there are no other trials with which to compare findings.

d. Indirectness: We downgraded once. The trial was conducted in one trial (high-income).

e. Imprecision: We downgraded twice. The sample size was small (OIS would not have been reached).

f. Miyamoto 2013: function was also measured using the Patient-Specific Functional Scale (PSFS, 0-10); MD = 1.10, 95% CI 0.23 to 1.97.

g. Miyamoto 2013: function was also measured using the Patient-Specific Functional Scale (PSFS, 0-10); MD = 0.80, 95% CI -0.00 to 1.60.

#### References

1.Miyamoto GC, Costa LO, Galvanin T, Cabral CM. Efficacy of the addition of modified Pilates exercises to a minimal intervention in patients with chronic low back pain: a randomized controlled trial. 2013.

GRADE Evidence Profile table 7: ***What are the benefits and harms of stretching, flexibility or mobilizing exercises in the management of community-dwelling adults (including older adults aged 60 years and over) with chronic primary low back pain (with or without leg pain) compared with no treatment/no additional treatment?***

| **Certainty assessment** | | | | | | | **№ of patients** | | **Effect** | | **Certainty** | **Importance** |
| --- | --- | --- | --- | --- | --- | --- | --- | --- | --- | --- | --- | --- |
| **№ of studies** | **Study design** | **Risk of bias** | **Inconsistency** | **Indirectness** | **Imprecision** | **Other considerations** | **Stretching, flexibility or mobilizing exercises** | **No intervention** | **Relative (95% CI)** | **Absolute (95% CI)** |
| **ALL ADULTS** | | | | | | | | | | | | |
| **Pain (no leg pain, low income country) (follow-up: closest to 2 weeks; assessed with: VAS; benefit indicated by lower values; Scale from: 0 to 10)** | | | | | | | | | | | | |
| 1^1,a^ | randomised trials | serious^b^ | not serious^c^ | serious^d^ | very serious^e^ | none | 15 | 15 | - | MD **0.18 lower** (1.61 lower to 1.25 higher) | ⨁◯◯◯ Very low | CRITICAL |
| **Function (no leg pain, low income country) (follow-up: closest to 2 weeks; assessed with: ODI; benefit indicated by lower values; Scale from: 0 to 100)** | | | | | | | | | | | | |
| 1^1,a^ | randomised trials | serious^b^ | not serious^c^ | serious^d^ | very serious^e^ | none | 15 | 15 | - | MD **3.97 lower** (13.14 lower to 5.19 higher) | ⨁◯◯◯ Very low | CRITICAL |
| **Adverse events/harms** | | | | | | | | | | | | |
| 0 |  |  |  |  |  |  |  |  |  |  |  | CRITICAL |

**CI:** confidence interval; **MD:** mean difference; **ODI:** Oswestry Disability Index; **VAS:** visual analogue scale

The following was used to guide the ratings.

**Risk of bias:** *Not serious:* trial(s) are rated as overall low risk of bias. *Serious:* some of the weight (>50%) comes from overall unclear risk of bias trial(s).

**Inconsistency:** *Not serious:* high extent of similarity of point estimates and overlap of confidence intervals; statistical heterogeneity (I^2^) is between 0% and 40%, which might not be important. *Serious:* some extent of similarity of point estimates and overlap of confidence intervals; statistical heterogeneity (I^2^) is between 30% and 60%, which could not be explained due to small subgroups and may represent moderate heterogeneity. *Very serious:* little or no similarity of point estimates and overlap of confidence intervals; statistical heterogeneity (I^2^) is between 50% and 90% or 75% and 100%, which could not be explained due to small subgroups and may represent substantial or considerable heterogeneity, respectively.

**Indirectness:** *Not serious:* trial(s) were conducted in different countries or settings. *Serious:* trial(s) were conducted from a single country/setting. *Very serious:* evidence is not directly related to PICO question.

**Imprecision:** *Not serious:* Optimal Information Size (OIS) was reached (i.e., sample sizes with at least 200 participants per group may provide prognostic balance); and the entire confidence interval lies on one side of the threshold that may be considered clinically important (≥10% scale range or SMD ≥0.2 for continuous variables, ≥10% for binary variables), such that the clinical course of action would not differ if the upper versus the lower boundary of the confidence interval represented the truth. *Serious:* OIS would not have been reached (sample sizes with less than 200 participants per group); if the OIS was reached, the clinical course of action might differ if the upper versus the lower boundary of the confidence interval represented the truth. *Very serious:* similar to ‘serious’ but to a greater extent (e.g., very small sample sizes and confidence intervals crossing appreciable benefit and harm).

**Other considerations:** *Not serious:* Publication bias is undetected. *Serious/very serious:* Publication bias is strongly suspected.

#### Explanations

a. Shamsi 2022: participants had individual supervised exercise; 12 sessions (session duration not reported).

b. Risk of bias: We downgraded once due to potential risk of bias (performance and detection domains).

c. Inconsistency: We did not downgrade; however, there are no other trials with which to compare findings.

d. Indirectness: We downgraded once. The trial was conducted in one country (low or lower-middle income).

e. Imprecision: We downgraded twice. The sample size is small (OIS would not have been met).

#### References

1.Shamsi M, Ahmadi A, Mirzaei M, Jaberzadeh S. Effects of static stretching and strengthening exercises on flexion relaxation ratio in patients with LBP: A randomized clinical trial. 2022.

GRADE Evidence Profile table 8: ***What are the benefits and harms of yoga exercise in the management of community-dwelling adults (including older adults aged 60 years and over) with chronic primary low back pain (with or without leg pain) compared with usual care?***

| **Certainty assessment** | | | | | | | **№ of patients** | | **Effect** | | **Certainty** | **Importance** |
| --- | --- | --- | --- | --- | --- | --- | --- | --- | --- | --- | --- | --- |
| **№ of studies** | **Study design** | **Risk of bias** | **Inconsistency** | **Indirectness** | **Imprecision** | **Other considerations** | **Yoga** | **Usual care** | **Relative (95% CI)** | **Absolute (95% CI)** |
| **ALL ADULTS** | | | | | | | | | | | | |
| **Pain (unclassified presence of leg pain, high-income country) (follow-up: closest to 2 weeks; assessed with: Aberdeen Back Pain Scale, 0-100; benefit indicated by lower values)** | | | | | | | | | | | | |
| 1^1,a,b^ | randomised trials | serious^c^ | not serious^d^ | serious^e^ | serious^f^ | none | Yoga vs. usual care: difference in mean change -2.42, 95% CI -4.97 to 0.12 (313 participants total). | | | | ⨁◯◯◯ Very low | CRITICAL |
| **Pain (unclassified presence of leg pain, high-income country) (follow-up: closest to 6 months; assessed with: Aberdeen Back Pain Scale, 0-100; benefit indicated by lower values)** | | | | | | | | | | | | |
| 1^1,a,b^ | randomised trials | serious^c^ | not serious^d^ | serious^e^ | serious^f^ | none | Yoga vs. usual care: difference in mean change -1.74, 95% CI -4.32 to 0.84 (313 participants total). | | | | ⨁◯◯◯ Very low | CRITICAL |
| **Pain (unclassified presence of leg pain, high-income country) (follow-up: closest to 12 months; assessed with: Aberdeen Pain Scale, 0-100; benefit indicated by lower values)** | | | | | | | | | | | | |
| 1^1,a,b^ | randomised trials | serious^c^ | not serious^d^ | serious^e^ | serious^f^ | none | Yoga vs. usual care: difference in mean change -0.73, 95% CI -3.30 to 1.84 (313 participants total). | | | | ⨁◯◯◯ Very low | CRITICAL |
| **Function (unclassified presence of leg pain, high-income country) (follow-up: closest to 2 weeks; assessed with: RMDQ, 0-24; benefit indicated by lower values)** | | | | | | | | | | | | |
| 1^1,a,b^ | randomised trials | serious^c^ | not serious^d^ | serious^e^ | serious^f^ | none | Yoga vs. usual care: difference in mean change -2.17, 95% CI -3.31 to -1.03 (313 participants total). | | | | ⨁◯◯◯ Very low | CRITICAL |
| **Function (unclassified presence of leg pain, high-income country) (follow-up: closest to 6 months; assessed with: RMDQ, 0-24; benefit indicated by lower values)** | | | | | | | | | | | | |
| 1^1,a,b^ | randomised trials | serious^c^ | not serious^d^ | serious^e^ | serious^f^ | none | Yoga vs. usual care: difference in mean change -1.48, 95% CI -2.62 to -0.33 (313 participants total). | | | | ⨁◯◯◯ Very low | CRITICAL |
| **Function (unclassified presence of leg pain, high-income country) (follow-up: closest to 12 months; assessed with: RMDQ, 0-24; benefit indicated by lower values)** | | | | | | | | | | | | |
| 1^1,a,b^ | randomised trials | serious^c^ | not serious^d^ | serious^e^ | serious^f^ | none | Yoga vs. usual care: difference in mean change -1.57, 95% CI -2.71 to -0.42 (313 participants total). | | | | ⨁◯◯◯ Very low | CRITICAL |
| **Health-related quality of life (unclassified presence of leg pain, high-income country) (follow-up: closest to 2 weeks; assessed with: SF-12, 0-100; benefit indicated by higher values)** | | | | | | | | | | | | |
| 1^1,a,b^ | randomised trials | serious^c^ | not serious^d^ | serious^e^ | serious^f^ | none | Yoga vs. usual care: difference in mean change 1.36, 95% CI -0.70 to 3.41 (PCS); 2.02, 95% CI -0.31 to 4.35 (MCS) (313 participants total). | | | | ⨁◯◯◯ Very low | CRITICAL |
| **Health-related quality of life (unclassified presence of leg pain, high-income country) (follow-up: closest to 6 months; assessed with: SF-12, 0-100; benefit indicated by higher values)** | | | | | | | | | | | | |
| 1^1,a,b^ | randomised trials | serious^c^ | not serious^d^ | serious^e^ | serious^f^ | none | Yoga vs. usual care: difference in mean change 1.24, 95% CI -0.83 to 3.33 (PCS); 2.02, 95% CI -0.34 to 4.37 (MCS) (313 participants total). | | | | ⨁◯◯◯ Very low | CRITICAL |
| **Health-related quality of life (unclassified presence of leg pain, high-income country) (follow-up: closest to 12 months; assessed with: SF-12, 0-100; benefit indicated by higher values)** | | | | | | | | | | | | |
| 1^1,a,b^ | randomised trials | serious^c^ | not serious^d^ | serious^e^ | serious^f^ | none | Yoga vs. usual care: difference in mean change 0.80, 95% CI -1.28 to 2.87 (PCS); 0.42, 95% CI -1.92 to 2.77 (MCS) (313 participants total). | | | | ⨁◯◯◯ Very low | CRITICAL |
| **Self-efficacy (unclassified presence of leg pain, high income-country) (follow-up: closest to 2 weeks; assessed with: PSEQ, 0-60; benefit indicated by higher values)** | | | | | | | | | | | | |
| 1^1,a,b^ | randomised trials | serious^c^ | not serious^d^ | serious^e^ | serious^f^ | none | Yoga vs. usual care: difference in mean change 2.96, 95% CI 0.35 to 5.58 (313 participants total). | | | | ⨁◯◯◯ Very low | CRITICAL |
| **Self-efficacy (unclassified presence of leg pain, high-income country) (follow-up: closest to 6 months; assessed with: PSEQ, 0-60; benefit indicated by higher values)** | | | | | | | | | | | | |
| 1^1,a,b^ | randomised trials | serious^c^ | not serious^d^ | serious^e^ | serious^f^ | none | Yoga vs. usual care: difference in mean change 3.33, 95% CI 0.68 to 5.97 (313 participants total). | | | | ⨁◯◯◯ Very low | CRITICAL |
| **Self-efficacy (unclassified presence of leg pain, high-income country) (follow-up: closest to 12 months; assessed with: PSEQ, 0-60; benefit indicated by higher values)** | | | | | | | | | | | | |
| 1^1,a,b^ | randomised trials | serious^c^ | not serious^d^ | serious^e^ | serious^f^ | none | Yoga vs. usual care: difference in mean change 1.75, 95% CI -0.87 to 4.38 (313 participants total). | | | | ⨁◯◯◯ Very low | CRITICAL |
| **Minor adverse events/harms (unclassified presence of leg pain, high-income country)** | | | | | | | | | | | | |
| 1^1,a,g^ | randomised trials | serious^c^ | not serious^d^ | serious^e^ | very serious^h^ | none | 11/135 (8.1%) | 0/139 (0.0%) | **OR 25.77** (1.50 to 441.85) | **0 fewer per 1,000** (from 0 fewer to 0 fewer) | ⨁◯◯◯ Very low | CRITICAL |
| **Serious adverse events/harms (unclassified presence of leg pain, high-income country)** | | | | | | | | | | | | |
| 1^1,a,i^ | randomised trials | serious^c^ | not serious^d^ | serious^e^ | very serious^h^ | none | 1/135 (0.7%) | 2/139 (1.4%) | **OR 0.51** (0.05 to 5.70) | **7 fewer per 1,000** (from 14 fewer to 62 more) | ⨁◯◯◯ Very low | CRITICAL |

**CI:** confidence interval; **OR:** odds ratio: **PSEQ:** Pain Self-Efficacy Questionnaire; **SF-12:** 12-item Short Form survey; **RMDQ:** Roland-Morris Disability Questionnaire

The following was used to guide the ratings.

**Risk of bias:** *Not serious:* trial(s) are rated as overall low risk of bias. *Serious:* some of the weight (>50%) comes from overall unclear risk of bias trial(s).

**Inconsistency:** *Not serious:* high extent of similarity of point estimates and overlap of confidence intervals; statistical heterogeneity (I^2^) is between 0% and 40%, which might not be important. *Serious:* some extent of similarity of point estimates and overlap of confidence intervals; statistical heterogeneity (I^2^) is between 30% and 60%, which could not be explained due to small subgroups and may represent moderate heterogeneity. *Very serious:* little or no similarity of point estimates and overlap of confidence intervals; statistical heterogeneity (I^2^) is between 50% and 90% or 75% and 100%, which could not be explained due to small subgroups and may represent substantial or considerable heterogeneity, respectively.

**Indirectness:** *Not serious:* trial(s) were conducted in different countries or settings. *Serious:* trial(s) were conducted from a single country/setting. *Very serious:* evidence is not directly related to PICO question.

**Imprecision:** *Not serious:* Optimal Information Size (OIS) was reached (i.e., sample sizes with at least 200 participants per group may provide prognostic balance); and the entire confidence interval lies on one side of the threshold that may be considered clinically important (≥10% scale range or SMD ≥0.2 for continuous variables, ≥10% for binary variables), such that the clinical course of action would not differ if the upper versus the lower boundary of the confidence interval represented the truth. *Serious:* OIS would not have been reached (sample sizes with less than 200 participants per group); if the OIS was reached, the clinical course of action might differ if the upper versus the lower boundary of the confidence interval represented the truth. *Very serious:* similar to ‘serious’ but to a greater extent (e.g., very small sample sizes and confidence intervals crossing appreciable benefit and harm).

**Other considerations:** *Not serious:* Publication bias is undetected. *Serious/very serious:* Publication bias is strongly suspected.

#### Explanations

a. Tilbrook 2011: Participants had group supervised exercise; 12 75-minute sessions.

b. Tilbrook 2011: only reported within-group changes; follow-up scores were not provided.

c. Risk of bias: We downgraded once due to potential risk of bias (performance and detection domains).

d. Inconsistency: We did not downgrade; however, there are no other trials with which to compare findings.

e. Indirectness: We downgraded once because the trial was conducted in one country (high-income).

f. Imprecision: We downgraded once. The sample size was small (OIS would not have been achieved).

g. Minor adverse events: included increased pain.

h. Imprecision: We downgraded twice due to small sample size and number of events.

i. Major adverse event: 1 participant experienced severe pain (possibly associated with yoga). In usual care group, 1 participant died; 1 had severe accident/injury.

#### References

1.Tilbrook HE, Cox H, Hewitt CE, et al. Yoga for chronic low back pain: a randomized trial. 2011.

GRADE Evidence Profile table 9: ***What are the benefits and harms of motor control exercise in the management of community-dwelling adults (including older adults aged 60 years and over) with chronic primary low back pain (with or without leg pain) compared with sham?***

| **Certainty assessment** | | | | | | | **№ of patients** | | **Effect** | | **Certainty** | **Importance** |
| --- | --- | --- | --- | --- | --- | --- | --- | --- | --- | --- | --- | --- |
| **№ of studies** | **Study design** | **Risk of bias** | **Inconsistency** | **Indirectness** | **Imprecision** | **Other considerations** | **Motor control exercise** | **Sham** | **Relative (95% CI)** | **Absolute (95% CI)** |
| **ALL ADULTS** | | | | | | | | | | | | |
| **Pain (unclassified presence of leg pain, high-income country) (follow-up: closest to 2 weeks; assessed with: NRS; benefit indicated by lower values; Scale from: 0 to 10)** | | | | | | | | | | | | |
| 1^1,a^ | randomised trials | not serious^b^ | not serious^c^ | serious^d^ | very serious^e^ | none | 77 | 77 | - | MD **1 lower** (1.85 lower to 0.15 lower) | ⨁◯◯◯ Very low |  |
| **Pain (unclassified presence of leg pain, high-income country) (follow-up: closest to 6 months; assessed with: NRS; benefit indicated by lower values; Scale from: 0 to 10)** | | | | | | | | | | | | |
| 1^1,a^ | randomised trials | not serious^b^ | not serious^c^ | serious^d^ | very serious^e^ | none | 77 | 77 | - | MD **0.6 lower** (1.46 lower to 0.26 higher) | ⨁◯◯◯ Very low |  |
| **Pain (unclassified presence of leg pain, high-income country) (follow-up: closest to 12 months; assessed with: NRS; benefit indicated by lower values; Scale from: 0 to 10)** | | | | | | | | | | | | |
| 1^1,a^ | randomised trials | not serious^b^ | not serious^c^ | serious^d^ | very serious^e^ | none | 77 | 77 | - | MD **1.3 lower** (2.13 lower to 0.47 lower) | ⨁◯◯◯ Very low |  |
| **Function (unclassified presence of leg pain, high-income country) (follow-up: closest to 2 weeks; assessed with: RMDQ; benefit indicated by lower values; Scale from: 0 to 24)** | | | | | | | | | | | | |
| 1^1,a,f^ | randomised trials | not serious^b^ | not serious^c^ | serious^d^ | very serious^e^ | none | 77 | 77 | - | MD **2.3 lower** (4.26 lower to 0.34 lower) | ⨁◯◯◯ Very low |  |
| **Function (unclassified presence of leg pain, high-income country) (follow-up: closest to 6 months; assessed with: RMDQ; benefit indicated by lower values; Scale from: 0 to 24)** | | | | | | | | | | | | |
| 1^1,a,g^ | randomised trials | not serious^b^ | not serious^c^ | serious^d^ | very serious^e^ | none | 77 | 77 | - | MD **1.9 lower** (4.06 lower to 0.26 higher) | ⨁◯◯◯ Very low |  |
| **Function (unclassified presence of leg pain, high-income country) (follow-up: closest to 12 months; assessed with: RMDQ; benefit indicated by lower values; Scale from: 0 to 24)** | | | | | | | | | | | | |
| 1^1,a,h^ | randomised trials | not serious^b^ | not serious^c^ | serious^d^ | very serious^e^ | none | 77 | 77 | - | MD **0.9 lower** (3.15 lower to 1.35 higher) | ⨁◯◯◯ Very low |  |
| **Adverse events/harms (unclassified presence of leg pain, high-income country)** | | | | | | | | | | | | |
| 1^1,a,i^ | randomised trials | not serious^b^ | not serious^c^ | serious^d^ | very serious^j^ | none | 3/77 (3.9%) | 2/77 (2.6%) | **OR 1.52** (0.25 to 9.36) | **13 more per 1,000** (from 19 fewer to 174 more) | ⨁◯◯◯ Very low |  |

**CI:** confidence interval; **MD:** mean difference; **NRS:** numeric rating scale; **OR:** odds ratio; **RMDQ:** Roland-Morris Disability Questionnaire

The following was used to guide the ratings.

**Risk of bias:** *Not serious:* trial(s) are rated as overall low risk of bias. *Serious:* some of the weight (>50%) comes from overall unclear risk of bias trial(s).

**Inconsistency:** *Not serious:* high extent of similarity of point estimates and overlap of confidence intervals; statistical heterogeneity (I^2^) is between 0% and 40%, which might not be important. *Serious:* some extent of similarity of point estimates and overlap of confidence intervals; statistical heterogeneity (I^2^) is between 30% and 60%, which could not be explained due to small subgroups and may represent moderate heterogeneity. *Very serious:* little or no similarity of point estimates and overlap of confidence intervals; statistical heterogeneity (I^2^) is between 50% and 90% or 75% and 100%, which could not be explained due to small subgroups and may represent substantial or considerable heterogeneity, respectively.

**Indirectness:** *Not serious:* trial(s) were conducted in different countries or settings. *Serious:* trial(s) were conducted from a single country/setting. *Very serious:* evidence is not directly related to PICO question.

**Imprecision:** *Not serious:* Optimal Information Size (OIS) was reached (i.e., sample sizes with at least 200 participants per group may provide prognostic balance); and the entire confidence interval lies on one side of the threshold that may be considered clinically important (≥10% scale range or SMD ≥0.2 for continuous variables, ≥10% for binary variables), such that the clinical course of action would not differ if the upper versus the lower boundary of the confidence interval represented the truth. *Serious:* OIS would not have been reached (sample sizes with less than 200 participants per group); if the OIS was reached, the clinical course of action might differ if the upper versus the lower boundary of the confidence interval represented the truth. *Very serious:* similar to ‘serious’ but to a greater extent (e.g., very small sample sizes and confidence intervals crossing appreciable benefit and harm).

**Other considerations:** *Not serious:* Publication bias is undetected. *Serious/very serious:* Publication bias is strongly suspected.

#### Explanations

a. Costa 2009: participants had individual supervised exercise; 12 30-minute sessions.

b. Risk of bias: We did not downgrade. The study was rated as overall low risk of bias.

c. Inconsistency: We did not downgrade; however, there are no other trials with which to compare findings.

d. Indirectness: We downgraded once because the trial was conducted in one country (high or upper-middle income).

e. Imprecision: We downgraded twice due to small sample size (OIS would not have been achieved).

f. Costa 2009: function was also measured using the Patient-Specific Functional Scale (PSFS, 0-10); MD = 1.10, 95% CI 0.36 to 1.84.

g. Costa 2009: function was also measured using the Patient-Specific Functional Scale (PSFS, 0-10); MD = 1.00, 95% CI 0.16 to 1.84.

h. Costa 2009: function was also measured using the Patient-Specific Functional Scale (PSFS, 0-10); MD = 1.50, 95% CI 0.68 to 2.32.

i. Adverse events: temporary exacerbations of pain.

j. Imprecision: We downgraded twice due to small sample size and number of events.

#### References

1.Costa LO, Maher CG, Latimer J, et al. Motor control exercise for chronic low back pain: a randomized placebo-controlled trial. 2009.

GRADE Evidence Profile table 10: ***What are the benefits and harms of exercise in the management of community-dwelling adults (including older adults aged 60 years and over) with chronic primary low back pain (with or without leg pain) compared with no treatment/no additional treatment?***

| **Certainty assessment** | | | | | | | **№ of patients** | | **Effect** | | **Certainty** | **Importance** |
| --- | --- | --- | --- | --- | --- | --- | --- | --- | --- | --- | --- | --- |
| **№ of studies** | **Study design** | **Risk of bias** | **Inconsistency** | **Indirectness** | **Imprecision** | **Other considerations** | **Exercise** | **No treatment** | **Relative (95% CI)** | **Absolute (95% CI)** |
| **ALL ADULTS**  **Pain (follow-up: closest to 2 weeks; assessed with: NRS, VAS, MPQ; benefit indicated by lower values)** | | | | | | | | | | | | |
| 8^1,2,3,4,5,6,7,8,a,b,c^ | randomised trials | serious^d^ | not serious^e^ | not serious^f^ | not serious^g^ | none | 318 | 301 | - | SMD **0.33 lower** (0.58 lower to 0.08 lower) | ⨁⨁⨁◯ Moderate | CRITICAL |
| **Pain in adults without leg pain (follow-up: closest to 2 weeks; assessed with: VAS; benefit indicated by lower values)** | | | | | | | | | | | | |
| 2^1,6,a^ | randomised trials | serious^d^ | not serious^h^ | not serious^f^ | very serious^i^ | none | 65 | 50 | - | SMD **0.2 lower** (0.58 lower to 0.17 higher) | ⨁◯◯◯ Very low | CRITICAL |
| **Pain in adults with unclassified presence of leg pain (follow-up: closest to 2 weeks; assessed with: NRS, VAS; benefit indicated by lower values)** | | | | | | | | | | | | |
| 5^2,3,4,5,7^ | randomised trials | serious^d^ | not serious^j^ | not serious^f^ | serious^k^ | none | 153 | 151 | - | SMD **0.51 lower** (0.92 lower to 0.1 lower) | ⨁⨁◯◯ Low | CRITICAL |
| **Pain in adults either with or without leg pain (unclassified radicular vs. non-radicular) (follow-up: closest to 2 weeks; assessed with: MPQ; benefit indicated by lower values )** | | | | | | | | | | | | |
| 1^8,b,c^ | randomised trials | serious^d^ | not serious^l^ | serious^m^ | very serious^n^ | none | 100 | 100 | - | SMD **0.1 lower** (0.44 lower to 0.23 higher) | ⨁◯◯◯ Very low | CRITICAL |
| **Pain in adults in high or upper-middle income country (follow-up: closest to 2 weeks; assessed with: NRS, VAS, MPQ; benefit indicated by lower values)** | | | | | | | | | | | | |
| 6^1,2,3,5,7,8,b,c^ | randomised trials | serious^d^ | serious^o^ | not serious^p^ | not serious^g^ | none | 248 | 246 | - | SMD **0.32 lower** (0.63 lower to 0.01 lower) | ⨁⨁◯◯ Low | CRITICAL |
| **Pain in adults in low or lower-middle income country (follow-up: closest to 2 weeks; assessed with: VAS; benefit indicated by lower values)** | | | | | | | | | | | | |
| 2^4,6,a^ | randomised trials | serious^d^ | not serious^q^ | serious^r^ | very serious^i^ | none | 70 | 55 | - | SMD **0.42 lower** (0.82 lower to 0.02 lower) | ⨁◯◯◯ Very low | CRITICAL |
| **Pain - aerobic exercise (follow-up: closest to 2 weeks; assessed with: VAS; benefit indicated by lower values)** | | | | | | | | | | | | |
| 2^3,5^ | randomised trials | serious^d^ | not serious^h^ | not serious^p^ | very serious^i^ | none | 44 | 44 | - | SMD **0.59 lower** (1.02 lower to 0.16 lower) | ⨁◯◯◯ Very low | CRITICAL |
| **Pain - core strengthening (follow-up: closest to 2 weeks; assessed with: VAS; benefit indicated by lower values)** | | | | | | | | | | | | |
| 1^4^ | randomised trials | serious^d^ | not serious^l^ | serious^r^ | very serious^i^ | none | 40 | 40 | - | SMD **0.65 lower** (1.1 lower to 0.2 lower) | ⨁◯◯◯ Very low | CRITICAL |
| **Pain - mixed exercise (follow-up: closest to 2 weeks; assessed with: VAS, MPQ; benefit indicated by lower values)** | | | | | | | | | | | | |
| 2^7,8,b,c^ | randomised trials | serious^d^ | not serious^s^ | not serious^p^ | very serious^n^ | none | 126 | 124 | - | SMD **0.01 lower** (0.32 lower to 0.31 higher) | ⨁◯◯◯ Very low | CRITICAL |
| **Pain - muscle strength training (follow-up: closest to 2 weeks; assessed with: VAS; benefit indicated by lower values)** | | | | | | | | | | | | |
| 2^1,6^ | randomised trials | serious^d^ | not serious^h^ | not serious^f^ | very serious^i^ | none | 50 | 43 | - | SMD **0.23 lower** (0.64 lower to 0.19 higher) | ⨁◯◯◯ Very low | CRITICAL |
| **Pain - Pilates (follow-up: closest to 2 weeks; assessed with: NRS; benefit indicated by lower values)** | | | | | | | | | | | | |
| 1^2^ | randomised trials | serious^d^ | not serious^l^ | serious^m^ | very serious^i^ | none | 43 | 43 | - | SMD **0.9 lower** (1.35 lower to 0.46 lower) | ⨁◯◯◯ Very low | CRITICAL |
| **Pain - stretching or flexibility/mobilizing exercise (follow-up: closest to 2 weeks; assessed with: VAS; benefit indicated by lower values)** | | | | | | | | | | | | |
| 1^6^ | randomised trials | serious^d^ | not serious^l^ | serious^r^ | very serious^i^ | none | 15 | 7 | - | SMD **0.09 lower** (0.98 lower to 0.81 higher) | ⨁◯◯◯ Very low | CRITICAL |
| **Pain (unclassified presence of leg pain, high or upper-middle income country) (follow-up: closest to 3 months; assessed with: VAS; benefit indicated by lower values; Scale from: 0 to 10)** | | | | | | | | | | | | |
| 2^5,7^ | randomised trials | serious^d^ | not serious^t^ | not serious^p^ | very serious^i^ | none | 50 | 47 | - | MD **0.68 lower** (1.82 lower to 0.46 higher) | ⨁◯◯◯ Very low | CRITICAL |
| **Pain - aerobic exercise (follow-up: closest to 3 months; assessed with: VAS; benefit indicated by lower values; Scale from: 0 to 10)** | | | | | | | | | | | | |
| 1^5^ | randomised trials | serious^d^ | not serious^l^ | serious^m^ | very serious^i^ | none | 24 | 23 | - | MD **1.26 lower** (2.51 lower to 0.01 lower) | ⨁◯◯◯ Very low | CRITICAL |
| **Pain - mixed exercise (follow-up: closest to 3 months; assessed with: VAS; benefit indicated by lower values; Scale from: 0 to 10)** | | | | | | | | | | | | |
| 1^7^ | randomised trials | serious^d^ | not serious^l^ | serious^m^ | very serious^i^ | none | 26 | 24 | - | MD **0.1 lower** (1.34 lower to 1.14 higher) | ⨁◯◯◯ Very low | CRITICAL |
| **Pain (high or upper-middle income country) (follow-up: closest to 6 months; assessed with: VAS, NRS, MPQ; benefit indicated by lower values)** | | | | | | | | | | | | |
| 4^1,2,7,8,b,u^ | randomised trials | serious^d^ | not serious^q^ | not serious^p^ | serious^v^ | none | 204 | 200 | - | SMD **0.08 lower** (0.29 lower to 0.13 higher) | ⨁⨁◯◯ Low | CRITICAL |
| **Pain in adults without leg pain (follow-up: closest to 6 months; assessed with: VAS; benefit indicated by lower values)** | | | | | | | | | | | | |
| 1^1^ | randomised trials | serious^d^ | not serious^l^ | serious^m^ | very serious^i^ | none | 35 | 35 | - | SMD **0.15 lower** (0.62 lower to 0.32 higher) | ⨁◯◯◯ Very low | CRITICAL |
| **Pain in adults with unclassified presence of leg pain (follow-up: closest to 6 months; assessed with: NRS, VAS; benefit indicated by lower values)** | | | | | | | | | | | | |
| 2^2,7^ | randomised trials | serious^d^ | serious^w^ | not serious^p^ | very serious^i^ | none | 69 | 65 | - | SMD **0.15 lower** (0.62 lower to 0.32 higher) | ⨁◯◯◯ Very low | CRITICAL |
| **Pain in adults either with or without leg pain (unclassified radicular vs. non-radicular) (follow-up: closest to 6 months; assessed with: MPQ; benefit indicated by lower values)** | | | | | | | | | | | | |
| 1^8,b,u^ | randomised trials | serious^d^ | not serious^l^ | serious^m^ | very serious^n^ | none | 100 | 100 | - | SMD **0.01 higher** (0.39 lower to 0.4 higher) | ⨁◯◯◯ Very low | CRITICAL |
| **Pain - mixed exercise (follow-up: closest to 6 months; assessed with: VAS, MPQ; benefit indicated by lower values)** | | | | | | | | | | | | |
| 2^7,8,b,u^ | randomised trials | serious^d^ | not serious^x^ | not serious^p^ | very serious^n^ | none | 126 | 122 | - | SMD **0.03 higher** (0.23 lower to 0.29 higher) | ⨁◯◯◯ Very low | CRITICAL |
| **Pain - muscle strength training (follow-up: closest to 6 months; assessed with: VAS; benefit indicated by lower values)** | | | | | | | | | | | | |
| 1^1^ | randomised trials | serious^d^ | not serious^l^ | serious^m^ | very serious^i^ | none | 35 | 35 | - | SMD **0.15 lower** (0.62 lower to 0.32 higher) | ⨁◯◯◯ Very low | CRITICAL |
| **Pain - Pilates (follow-up: closest to 6 months; assessed with: NRS; benefit indicated by lower values)** | | | | | | | | | | | | |
| 1^2^ | randomised trials | serious^d^ | not serious^l^ | serious^m^ | very serious^i^ | none | 43 | 43 | - | SMD **0.35 lower** (0.78 lower to 0.07 higher) | ⨁◯◯◯ Very low | CRITICAL |
| **Pain (high-income country, either with or without non-radicular leg pain, mixed exercise) (follow-up: closest to 12 months; assessed with: VAS, 0-100; benefit indicated by lower values)** | | | | | | | | | | | | |
| 1^9,y^ | randomised trials | not serious^z^ | not serious^l^ | serious^m^ | very serious^i^ | none | Between-group MD graded activity with problem solving training alone vs. combination treatment = 8.88, 95% CI -0.36 to 18.13 (119 participants total). | | | | ⨁◯◯◯ Very low | CRITICAL |
| **Function (follow-up: closest to 2 weeks; assessed with: RMDQ, ODI, Hannover, WI; benefit indicated by lower values)** | | | | | | | | | | | | |
| 8^1,2,3,4,5,6,7,8,a,aa,b^ | randomised trials | serious^d^ | not serious^ab^ | not serious^f^ | not serious^g^ | none | 316 | 303 | - | SMD **0.31 lower** (0.57 lower to 0.05 lower) | ⨁⨁⨁◯ Moderate | CRITICAL |
|  | | | | | | | | | | | | |
| **Function in adults without leg pain (follow-up: closest to 2 weeks; assessed with: RMDQ, ODI; benefit indicated by lower values)** | | | | | | | | | | | | |
| 2^1,6,a^ | randomised trials | serious^d^ | not serious^h^ | not serious^f^ | very serious^i^ | none | 65 | 50 | - | SMD **0.01 lower** (0.38 lower to 0.37 higher) | ⨁◯◯◯ Very low | CRITICAL |
| **Function in adults with unclassified presence of leg pain (follow-up: closest to 2 weeks; assessed with: RMDQ, ODI, Hannover, WI; benefit indicated by lower values)** | | | | | | | | | | | | |
| 5^2,3,4,5,7^ | randomised trials | serious^d^ | not serious^t^ | not serious^f^ | serious^k^ | none | 151 | 153 | - | SMD **0.6 lower** (0.9 lower to 0.29 lower) | ⨁⨁◯◯ Low | CRITICAL |
| **Function in adults either with or without leg pain (unclassified radicular vs. non-radicular) (follow-up: closest to 2 weeks; assessed with: RMDQ; benefit indicated by lower values)** | | | | | | | | | | | | |
| 1^8,aa,b^ | randomised trials | serious^d^ | not serious^l^ | serious^m^ | very serious^n^ | none | 100 | 100 | - | SMD **0.01 lower** (0.29 lower to 0.27 higher) | ⨁◯◯◯ Very low | CRITICAL |
| **Function in adults in high or upper-middle income country (follow-up: closest to 2 weeks; assessed with: RMDQ, ODI, Hannover, WI; benefit indicated by lower values)** | | | | | | | | | | | | |
| 6^1,2,3,5,7,8,aa,b^ | randomised trials | serious^d^ | not serious^ac^ | not serious^p^ | serious^ad^ | none | 246 | 248 | - | SMD **0.23 lower** (0.47 lower to 0.01 higher) | ⨁⨁◯◯ Low | CRITICAL |
| **Function in adults in low or lower-middle income country (follow-up: closest to 2 weeks; assessed with: RMDQ, ODI; benefit indicated by lower values)** | | | | | | | | | | | | |
| 2^4,6,a^ | randomised trials | serious^d^ | serious^o^ | serious^r^ | very serious^i^ | none | 70 | 55 | - | SMD **0.5 lower** (1.2 lower to 0.21 higher) | ⨁◯◯◯ Very low | CRITICAL |
| **Function - aerobic exercise (follow-up: closest to 2 weeks; assessed with: ODI, Hannover; benefit indicated by lower values)** | | | | | | | | | | | | |
| 2^3,5^ | randomised trials | serious^d^ | not serious^h^ | not serious^p^ | very serious^i^ | none | 44 | 44 | - | SMD **0.22 lower** (0.64 lower to 0.2 higher) | ⨁◯◯◯ Very low | CRITICAL |
| **Function - core strengthening (follow-up: closest to 2 weeks; assessed with: RMDQ; benefit indicated by lower values)** | | | | | | | | | | | | |
| 1^4^ | randomised trials | serious^d^ | not serious^l^ | serious^r^ | very serious^i^ | none | 40 | 40 | - | SMD **1.02 lower** (1.49 lower to 0.55 lower) | ⨁◯◯◯ Very low | CRITICAL |
| **Function - mixed exercise (follow-up: closest to 2 weeks; assessed with: RMDQ, WI; benefit indicated by lower values)** | | | | | | | | | | | | |
| 2^7,8,aa,b^ | randomised trials | serious^d^ | serious^ae^ | not serious^p^ | serious^k^ | none | 124 | 126 | - | SMD **0.15 lower** (0.48 lower to 0.18 higher) | ⨁◯◯◯ Very low | CRITICAL |
| **Function - muscle strength training (follow-up: closest to 2 weeks; assessed with: RMDQ, ODI; benefit indicated by lower values)** | | | | | | | | | | | | |
| 2^1,6^ | randomised trials | serious^d^ | not serious^h^ | not serious^f^ | very serious^i^ | none | 50 | 43 | - | SMD **0.05 higher** (0.36 lower to 0.46 higher) | ⨁◯◯◯ Very low | CRITICAL |
| **Function - Pilates (follow-up: closest to 2 weeks; assessed with: RMDQ; benefit indicated by lower values)** | | | | | | | | | | | | |
| 1^2^ | randomised trials | serious^d^ | not serious^l^ | serious^m^ | very serious^i^ | none | 43 | 43 | - | SMD **0.74 lower** (1.18 lower to 0.3 lower) | ⨁◯◯◯ Very low | CRITICAL |
| **Function - stretching or flexibility/mobilizing exercise (follow-up: closest to 2 weeks; assessed with: ODI; benefit indicated by lower values)** | | | | | | | | | | | | |
| 1^6^ | randomised trials | serious^d^ | not serious^l^ | serious^r^ | very serious^i^ | none | 15 | 7 | - | SMD **0.29 lower** (1.2 lower to 0.61 higher) | ⨁◯◯◯ Very low | CRITICAL |
| **Function (high or upper-middle income country, unclassified presence of leg pain) (follow-up: closest to 3 months; assessed with: Hannover, WI; benefit indicated by lower values)** | | | | | | | | | | | | |
| 2^5,7^ | randomised trials | serious^d^ | not serious^h^ | not serious^p^ | very serious^i^ | none | 47 | 50 | - | SMD **0.26 lower** (0.67 lower to 0.14 higher) | ⨁◯◯◯ Very low | CRITICAL |
| **Function - aerobic exercise (follow-up: closest to 3 months; assessed with: Hannover; benefit indicated by lower values)** | | | | | | | | | | | | |
| 1^5^ | randomised trials | serious^d^ | not serious^l^ | serious^m^ | very serious^i^ | none | 23 | 24 | - | SMD **0.08 lower** (0.65 lower to 0.49 higher) | ⨁◯◯◯ Very low | CRITICAL |
| **Function - mixed exercise (follow-up: closest to 3 months; assessed with: WI; benefit indicated by lower values)** | | | | | | | | | | | | |
| 1^7^ | randomised trials | serious^d^ | not serious^l^ | serious^m^ | very serious^i^ | none | 24 | 26 | - | SMD **0.44 lower** (1.01 lower to 0.12 higher) | ⨁◯◯◯ Very low | CRITICAL |
| **Function (high or upper-middle income country) (follow-up: closest to 6 months; assessed with: RMDQ, WI; benefit indicated by lower values)** | | | | | | | | | | | | |
| 4^1,2,7,8,af,b^ | randomised trials | serious^d^ | not serious^ag^ | not serious^p^ | serious^v^ | none | 200 | 204 | - | SMD **0.16 lower** (0.39 lower to 0.07 higher) | ⨁⨁◯◯ Low | CRITICAL |
| **Function in adults without leg pain (follow-up: closest to 6 months; assessed with: RMDQ; benefit indicated by lower values)** | | | | | | | | | | | | |
| 1^1^ | randomised trials | serious^d^ | not serious^l^ | serious^m^ | very serious^i^ | none | 35 | 35 | - | SMD **0.11 lower** (0.58 lower to 0.36 higher) | ⨁◯◯◯ Very low | CRITICAL |
| **Function in adults with unclassified presence of leg pain (follow-up: closest to 6 months; assessed with: RMDQ, WI; benefit indicated by lower values)** | | | | | | | | | | | | |
| 2^2,7^ | randomised trials | serious^d^ | not serious^h^ | not serious^p^ | very serious^i^ | none | 65 | 69 | - | SMD **0.46 lower** (0.8 lower to 0.11 lower) | ⨁◯◯◯ Very low | CRITICAL |
| **Function in adults either with or without leg pain (unclassified radicular vs. non-radicular) (follow-up: closest to 6 months; assessed with: RMDQ; benefit indicated by lower values)** | | | | | | | | | | | | |
| 1^8,af,b^ | randomised trials | serious^d^ | not serious^l^ | serious^m^ | very serious^n^ | none | 100 | 100 | - | SMD **0.03 higher** (0.24 lower to 0.31 higher) | ⨁◯◯◯ Very low | CRITICAL |
| **Function - mixed exercise (follow-up: closest to 6 months; assessed with: RMDQ, WI; benefit indicated by lower values)** | | | | | | | | | | | | |
| 2^7,8,af,b^ | randomised trials | serious^d^ | not serious^t^ | not serious^p^ | very serious^n^ | none | 122 | 126 | - | SMD **0.09 lower** (0.42 lower to 0.24 higher) | ⨁◯◯◯ Very low | CRITICAL |
| **Function - muscle strength training (follow-up: closest to 6 months; assessed with: RMDQ; benefit indicated by lower values)** | | | | | | | | | | | | |
| 1^1^ | randomised trials | serious^d^ | not serious^l^ | serious^m^ | very serious^i^ | none | 35 | 35 | - | SMD **0.11 lower** (0.58 lower to 0.36 higher) | ⨁◯◯◯ Very low | CRITICAL |
| **Function - Pilates (follow-up: closest to 6 months; assessed with: RMDQ; benefit indicated by lower values)** | | | | | | | | | | | | |
| 1^2^ | randomised trials | serious^d^ | not serious^l^ | serious^m^ | very serious^i^ | none | 43 | 43 | - | SMD **0.43 lower** (0.86 lower to 0 ) | ⨁◯◯◯ Very low | CRITICAL |
| **Function (high-income country, either with or without non-radicular leg pain, mixed exercise) (follow-up: closest to 12 months; assessed with: RMDQ, 0 to 24; benefit indicated by lower values)** | | | | | | | | | | | | |
| 1^9,c^ | randomised trials | not serious^z^ | not serious^l^ | serious^m^ | very serious^i^ | none | Between-group MD (RMDQ 0-24) graded activity with problem solving training alone vs. combination treatment = 1.62, 95% CI -0.06 to 3.31 (119 partipants total). | | | | ⨁◯◯◯ Very low | CRITICAL |
| **Health-related quality of life (high or upper-middle income country) (follow-up: closest to 2 weeks; assessed with: SF-36 (PCS); benefit indicated by higher values; Scale from: 0 to 100)** | | | | | | | | | | | | |
| 2^5,8,b^ | randomised trials | serious^d^ | serious^ah^ | not serious^p^ | serious^k^ | none | 124 | 124 | - | MD **2.31 lower** (10.36 lower to 5.75 higher) | ⨁◯◯◯ Very low | CRITICAL |
| **Health-related quality of life in adults with unclassified presence of leg pain (follow-up: closest to 2 weeks; assessed with: SF-36 (PCS); benefit indicated by higher values; Scale from: 0 to 100)** | | | | | | | | | | | | |
| 1^5^ | randomised trials | serious^d^ | not serious^l^ | serious^m^ | very serious^i^ | none | 24 | 24 | - | MD **3.5 higher** (0.05 lower to 7.05 higher) | ⨁◯◯◯ Very low | CRITICAL |
| **Health-related quality of life in adults either with or without leg pain (unclassified radicular vs. non-radicular) (follow-up: closest to 2 weeks; assessed with: SF-36 (PCS); benefit indicated by higher values; Scale from: 0 to 100)** | | | | | | | | | | | | |
| 1^8,b^ | randomised trials | serious^d^ | not serious^l^ | serious^m^ | serious^k^ | none | 100 | 100 | - | MD **6.56 lower** (13.03 lower to 0.1 lower) | ⨁◯◯◯ Very low | CRITICAL |
| **Health-related quality of life - aerobic exercise (follow-up: closest to 2 weeks; assessed with: SF-36 (PCS); benefit indicated by higher values; Scale from: 0 to 100)** | | | | | | | | | | | | |
| 1^5^ | randomised trials | serious^d^ | not serious^l^ | serious^m^ | very serious^i^ | none | 24 | 24 | - | MD **3.5 higher** (0.05 lower to 7.05 higher) | ⨁◯◯◯ Very low | CRITICAL |
| **Health-related quality of life - mixed exercise (follow-up: closest to 2 weeks; assessed with: SF-36 (PCS); benefit indicated by higher values; Scale from: 0 to 100)** | | | | | | | | | | | | |
| 1^8,b^ | randomised trials | serious^d^ | not serious^l^ | serious^m^ | serious^k^ | none | 100 | 100 | - | MD **6.56 lower** (13.03 lower to 0.1 lower) | ⨁◯◯◯ Very low | CRITICAL |
| **Health-related quality of life (high or upper-middle income country) (follow-up: closest to 2 weeks; assessed with: SF-36 (MCS); benefit indicated by higher values; Scale from: 0 to 100)** | | | | | | | | | | | | |
| 2^5,8,b^ | randomised trials | serious^d^ | not serious^h^ | not serious^p^ | serious^k^ | none | 124 | 124 | - | MD **1.11 lower** (3.67 lower to 1.45 higher) | ⨁⨁◯◯ Low | CRITICAL |
| **Health-related quality of life in adults with unclassified presence of leg pain (follow-up: closest to 2 weeks; assessed with: SF-36 (MCS); benefit indicated by higher values; Scale from: 0 to 100)** | | | | | | | | | | | | |
| 1^5^ | randomised trials | serious^d^ | not serious^l^ | serious^m^ | very serious^i^ | none | 24 | 24 | - | MD **1.2 lower** (5.22 lower to 2.82 higher) | ⨁◯◯◯ Very low | CRITICAL |
| **Health-related quality of life in adults either with or without leg pain (unclassified radicular vs. non-radicular) (follow-up: closest to 2 weeks; assessed with: SF-36 (MCS); benefit indicated by higher values; Scale from: 0 to 100)** | | | | | | | | | | | | |
| 1^8,b^ | randomised trials | serious^d^ | not serious^l^ | serious^m^ | serious^k^ | none | 100 | 100 | - | MD **1.05 lower** (4.38 lower to 2.28 higher) | ⨁◯◯◯ Very low | CRITICAL |
| **Health-related quality of life - aerobic exercise (follow-up: closest to 2 weeks; assessed with: SF-36 (MCS); benefit indicated by higher values; Scale from: 0 to 100)** | | | | | | | | | | | | |
| 1^5^ | randomised trials | serious^d^ | not serious^l^ | serious^m^ | very serious^i^ | none | 24 | 24 | - | MD **1.2 lower** (5.22 lower to 2.82 higher) | ⨁◯◯◯ Very low | CRITICAL |
| **Health-related quality of life - mixed exercise (follow-up: closest to 2 weeks; assessed with: SF-36 (MCS); benefit indicated by higher values; Scale from: 0 to 100)** | | | | | | | | | | | | |
| 1^8,b^ | randomised trials | serious^d^ | not serious^l^ | serious^m^ | serious^k^ | none | 100 | 100 | - | MD **1.05 lower** (4.38 lower to 2.28 higher) | ⨁◯◯◯ Very low | CRITICAL |
| **Adverse events (high or upper-middle income country, either with or without leg pain, mixed exercise)** | | | | | | | | | | | | |
| 2^8,9,ai,b^ | randomised trials | serious^d^ | not serious^h^ | not serious^p^ | very serious^aj^ | none | 5/155 (3.2%) | 0/155 (0.0%) | **OR 4.24** (0.69 to 25.95) | **0 fewer per 1,000** (from 0 fewer to 0 fewer) | ⨁◯◯◯ Very low | CRITICAL |

**CI:** confidence interval; **MD:** mean difference; **OR:** odds ratio; **SMD:** standardized mean difference

The following was used to guide the ratings.

**Risk of bias:** *Not serious:* trial(s) are rated as overall low risk of bias. *Serious:* some of the weight (>50%) comes from overall unclear risk of bias trial(s).

**Inconsistency:** *Not serious:* high extent of similarity of point estimates and overlap of confidence intervals; statistical heterogeneity (I^2^) is between 0% and 40%, which might not be important. *Serious:* some extent of similarity of point estimates and overlap of confidence intervals; statistical heterogeneity (I^2^) is between 30% and 60%, which could not be explained due to small subgroups and may represent moderate heterogeneity. *Very serious:* little or no similarity of point estimates and overlap of confidence intervals; statistical heterogeneity (I^2^) is between 50% and 90% or 75% and 100%, which could not be explained due to small subgroups and may represent substantial or considerable heterogeneity, respectively.

**Indirectness:** *Not serious:* trial(s) were conducted in different countries or settings. *Serious:* trial(s) were conducted from a single country/setting. *Very serious:* evidence is not directly related to PICO question.

**Imprecision:** *Not serious:* Optimal Information Size (OIS) was reached (i.e., sample sizes with at least 200 participants per group may provide prognostic balance); and the entire confidence interval lies on one side of the threshold that may be considered clinically important (≥10% scale range or SMD ≥0.2 for continuous variables, ≥10% for binary variables), such that the clinical course of action would not differ if the upper versus the lower boundary of the confidence interval represented the truth. *Serious:* OIS would not have been reached (sample sizes with less than 200 participants per group); if the OIS was reached, the clinical course of action might differ if the upper versus the lower boundary of the confidence interval represented the truth. *Very serious:* similar to ‘serious’ but to a greater extent (e.g., very small sample sizes and confidence intervals crossing appreciable benefit and harm).

**Other considerations:** *Not serious:* Publication bias is undetected. *Serious/very serious:* Publication bias is strongly suspected.

#### Explanations

a. Shamsi 2022: had 2 comparisons (both included in meta-analysis).

b. Weiner 2008: had 2 comparisons (both included in meta-analysis).

c. Smeets 2008 was not included in the meta-analysis (provided within-group mean changes; no follow-up scores). 119 participants rated as overall low risk of bias. Participants performed combination treatment (active physical treatment [aerobic and core strengthening exercises] + graded activity with problem solving training) vs. graded activity with problem solving training alone. Between-group MD (VAS 0-100) graded activity with problem solving training alone vs. combination treatment = 5.35, 95% CI -3.73 to 14.42.

d. Risk of bias: We downgraded once. Most or all trial(s) rated as overall unclear risk of bias.

e. Inconsistency: We did not downgrade. Most of the point estimates are in the same direction and confidence intervals overlap. Statistical heterogeneity is between 30% and 60% (i.e., I2 = 55%). This could not be explained due to small subgroups and may represent moderate heterogeneity.

f. Indirectness: We did not downgrade. The trials were conducted in different countries (high- and low-income).

g. Imprecision: We did not downgrade. The point estimate reached the pre-specified threshold for what may be considered clinically important (MD ≥ 1 or SMD ≥ 0.2 ). The confidence interval does not cross the null.

h. Inconsistency: We did not downgrade. Most or all the point estimates are in the same direction and confidence intervals overlap. Statistical heterogeneity is between 0% and 40%, which might not be important (i.e., I2 = 0%).

i. Imprecision: We downgraded twice. The sample size was small (OIS would not have been achieved).

j. Inconsistency: We downgraded once. Most of the point estimates are in the same direction and confidence intervals overlap. Statistical heterogeneity is between 50% and 90% (i.e., I2 = 67%). This could not be explained due to small subgroups and may represent substantial heterogeneity.

k. Imprecision: We downgraded once. The sample size was small (OIS would not have been achieved).

l. Inconsistency: We did not downgrade; however, there are no other trials with which to compare findings.

m. Indirectness: We downgraded once. The trial was conducted in one country (high or upper-middle income).

n. Imprecision: We downgraded twice. The point estimate did not reach the pre-specified threshold for what may be considered clinically important (MD ≥ 1 or SMD ≥ 0.2). The boundaries of the confidence interval cross the thresholds for what may be considered appreciable benefit and harm (MD ≥ 1 or SMD ≥ 0.2).

o. Inconsistency: We downgraded once. Most of the point estimates are in the same direction and confidence intervals overlap. Statistical heterogeneity is between 50% and 90% (i.e., I2 = 65%). This could not be explained due to small subgroups and may represent substantial heterogeneity.

p. Indirectness: We did not downgrade. The trials were conducted in different countries (high or upper-middle income).

q. Inconsistency: We did not downgrade. Most or all the point estimates are in the same direction and confidence intervals overlap. Statistical heterogeneity is between 0% and 40%, which might not be important (i.e., I2 = 11%).

r. Indirectness: We downgraded once; trial(s) conducted in one country (low or lower-middle income).

s. Inconsistency: We did not downgrade. Most of the point estimates are in the same direction and confidence intervals overlap. Statistical heterogeneity is between 0% and 40%, which might not be important (i.e., I2 = 34%).

t. Inconsistency: We did not downgrade. Most or all the point estimates are in the same direction and confidence intervals overlap. Statistical heterogeneity is between 0% and 40%, which might not be important (i.e., I2 = 40%).

u. Smeets 2008 was not included in the meta-analysis (provided within-group mean changes; no follow-up scores). 119 participants rated as overall low risk of bias. Participants performed combination treatment (active physical treatment [aerobic and core strengthening exercises] + graded activity with problem solving training) vs. graded activity with problem solving training alone. Between-group MD (VAS 0-100) graded activity with problem solving training alone vs. combination treatment = 6.25, 95% CI -2.94 to 15.44.

v. Imprecision: We downgraded once. The point estimate did not reach the pre-specified threshold for what may be considered clinically important (MD ≥ 1 or SMD ≥ 0.2). One of the boundaries of the confidence interval crosses this threshold.

w. Inconsistency: We downgraded once. The point estimates are in different directions with some overlap of confidence intervals. Statistical heterogeneity is between 30% and 60% (i.e., I2 = 44%). This could not be explained due to small subgroups and may represent moderate heterogeneity.

x. Inconsistency: We did not downgrade. Most or all of the point estimates are in the same direction and confidence intervals overlap. Statistical heterogeneity is between 0% and 40%, which might not be important (i.e., I2 = 6%).

y. Smeets 2008 provided within-group mean changes, no follow-up scores.

z. Risk of bias: We did not downgrade because the study was rated as overall low risk of bias.

aa. Smeets 2008 was not included in the meta-analysis (provided within-group mean changes; no follow-up scores). 119 participants rated as overall low risk of bias. Participants performed combination treatment (active physical treatment [aerobic and core strengthening exercises] + graded activity with problem solving training) vs. graded activity with problem solving training alone. Between-group MD (RMDQ 0-24) graded activity with problem solving training alone vs. combination treatment = 0.58, 95% CI -1.08 to 2.24.

ab. Inconsistency: We did not downgrade. Most of the point estimates are in the same direction with some overlap of confidence intervals. Statistical heterogeneity is between 30% and 60% (i.e., I2 = 58%). This could not be explained due to small subgroups and may represent moderate heterogeneity.

ac. Inconsistency: We did not downgrade. Most of the point estimates are in the same direction and confidence intervals overlap. Statistical heterogeneity is between 30% and 60% (i.e., I2 = 44%). This could not be explained due to small subgroups and may represent moderate heterogeneity.

ad. Imprecision: We downgraded once. The point estimate reached the pre-specified threshold for what may be considered clinically important (MD ≥ 1 or SMD ≥ 0.2). One of the boundaries of the confidence interval crosses this threshold.

ae. Inconsistency: We downgraded once. Most of the point estimates are in the same direction and confidence intervals overlap. Statistical heterogeneity is between 30% and 60% (i.e., I2 = 40%). This could not be explained due to small subgroups and may represent moderate heterogeneity.

af. Smeets 2008 was not included in the meta-analysis (provided within-group mean changes; no follow-up scores). 119 participants rated as overall low risk of bias. Participants performed combination treatment (active physical treatment [aerobic and core strengthening exercises] + graded activity with problem solving training) vs. graded activity with problem solving training alone. Between-group MD (RMDQ 0-24) graded activity with problem solving training alone vs. combination treatment = 1.11, 95% CI -0.56 to 2.79.

ag. Inconsistency: We did not downgrade. Most of the point estimates are in the same direction and confidence intervals overlap. Statistical heterogeneity is between 0% and 40%, which might not be important (i.e., I2 = 26%).

ah. Inconsistency: We downgraded once. Most of the point estimates are in the same direction and confidence intervals overlap. Statistical heterogeneity is between 50% and 90% (i.e., I2 = 74%). This could not be explained due to small subgroups and may represent substantial heterogeneity.

ai. Adverse events: were mainly minor and included back and knee pain.

aj. Imprecision: We downgraded twice due to small sample size and number of events.

#### References

1.Fukuda TY, Aquino LM, Pereira P, et al. Does adding hip strengthening exercises to manual therapy and segmental stabilization improve outcomes in patients with nonspecific low back pain? A randomized controlled trial. 2021.

2.Miyamoto GC, Costa LO, Galvanin T, Cabral CM. Efficacy of the addition of modified Pilates exercises to a minimal intervention in patients with chronic low back pain: a randomized controlled trial. 2013.

3.Nardin DMK, Stocco MR ,Aguiar AF, Machado FA, de Oliveira RG, Andraus RAC. Effects of photobiomodulation and deep water running in patients with chronic non-specific low back pain: a randomized controlled trial. 2022.

4.Rahbar M, Salekzamani Y,J ahanjou F, Eslamian F, Niroumand A, Dolatkhah N. Effect of hippotherapy simulator on pain, disability, and range of motion of the spinal column in subjects with mechanical low back pain: A randomized single-blind clinical trial. 2018.

5.Rotter G, Ortiz M, Binting S, et al. Mindful Walking in Patients with Chronic Low Back Pain: A Randomized Controlled Trial. 2022.

6.Shamsi M, Ahmadi A, Mirzaei M, Jaberzadeh S. Effects of static stretching and strengthening exercises on flexion relaxation ratio in patients with LBP: A randomized clinical trial. 2022.

7.Z, Takinaci. Clinical efficacy of spa therapy (balneotherapy) for chronic low back pain: A randomized single-blind trial. 2019.

8.Weiner DK, Perera S, Rudy TE, Glick RM, Shenoy S, Delitto A. Efficacy of percutaneous electrical nerve stimulation and therapeutic exercise for older adults with chronic low back pain: a randomized controlled trial. 2008.

9.Smeets R, Vlaeyen JWS, Hidding A, Kester ADM, van der Heijden G, Knottnerus AJ. Chronic low back pain: physical training, graded activity with problem solving training, or both? The one-year post-treatment results of a randomized controlled trial. 2008.

**Online Resource 6.** GRADE Summary of Findings tables

Summary of Findings table 1: ***What are the benefits and harms of aerobic exercise in the management of community-dwelling adults (including older adults aged 60 years and over) with chronic primary low back pain (with or without leg pain) compared with no treatment/no additional treatment?***

| Outcomes | **Anticipated absolute effects^*^** (95% CI) | | Relative effect (95% CI) | № of participants (studies) | Certainty of the evidence (GRADE) | Comments |
| --- | --- | --- | --- | --- | --- | --- |
|  | **Risk with no intervention** | **Risk with aerobic exercise** |  |  |  |  |
| **All Adults** | | | | | | |
| **Pain**  0 to 10; 0 = no pain follow-up: closest to 2 weeks | The mean pain was **3.5 to 4.54** | MD **1.33 lower** (2.27 lower to 0.4 lower) | - | 88 (2 RCTs)^1,2,a,b^ | ⨁◯◯◯ Very low^c,d,e,f^ | Aerobic exercise reduces pain more than no treatment in the immediate term. |
| **Pain**  0 to 10; 0 = no pain follow-up: closest to 3 months | The mean pain was **4.74** | MD **1.26 lower** (2.51 lower to 0.01 lower) | - | 47 (1 RCT)^2,b^ | ⨁◯◯◯ Very low^f,g,h,i^ | Aerobic exercise reduces pain more than no treatment in the short term. |
| **Function** 0 to 100; 0 = no disability follow-up: closest to 2 weeks | The mean function was **7.6 to 75.1** | MD **1.3 lower** (3.89 lower to 1.29 higher) | - | 88 (2 RCTs)^1,2,a,b^ | ⨁◯◯◯ Very low^c,d,e,f^ | Aerobic exercise does not improve function compared to no treatment in the immediate term. |
| **Function**  0 to 100; 0 = no disability follow-up: closest to 3 months | The mean function was **74** | MD **0.9 higher** (5.66 lower to 7.46 higher) | - | 47 (1 RCT)^2,b^ | ⨁◯◯◯ Very low^f,g,h,i^ | Aerobic exercise does not improve function compared to no treatment in the short term. |
| **Health-related quality of life**  (physical component) 0 to 100; 0 = poor QofL follow-up: closest to 2 weeks | The mean health-related quality of life was **39.9** | MD **3.5 higher** (0.05 lower to 7.05 higher) | - | 48 (1 RCT)^2,b^ | ⨁◯◯◯ Very low^f,g,h,i^ | Aerobic exercise does not improve health-related quality of life (physical component) compared to no treatment in the immediate term. |
| **Health-related quality of life**  mental component) 0 to 100; 0 = poor QofL follow-up: closest to 2 weeks | The mean health-related quality of life was **46.9** | MD **1.2 lower** (5.22 lower to 2.82 higher) | - | 48 (1 RCT)^2,b^ | ⨁◯◯◯ Very low^f,g,h,i^ | Aerobic exercise does not improve health-related quality of life (mental component) compared to no treatment in the immediate term. |
| **Health-related quality of life**  physical component) 0 to 100; 0 = poor QofL follow-up: closest to 3 months | The mean health-related quality of life was **39.9** | MD **3.7 higher** (0.05 higher to 7.35 higher) | - | 47 (1 RCT)^2,b^ | ⨁◯◯◯ Very low^f,g,h,i^ | Aerobic exercise does not improve health-related quality of life (physical component) compared to no treatment in the short term. |
| **Health-related quality of life**  mental component) 0 to 100; 0 = poor QofL follow-up: closest to 3 months | The mean health-related quality of life was **44.5** | MD **2.2 higher** (3.15 lower to 7.55 higher) | - | 47 (1 RCT)^2,b^ | ⨁◯◯◯ Very low^f,g,h,i^ | Aerobic exercise does not improve health-related quality of life (mental component) compared to no treatment in the short term. |
| Psychological functioning (depression, fear avoidance, catastrophizing, anxiety, self-efficacy) | The mean psychological functioning (depression, fear avoidance, catastrophizing, anxiety, self-efficacy) was **0** | **0**  (0 to 0 ) | - | (0 studies) | - |  |
| Social participation | The mean social participation was **0** | **0**  (0 to 0 ) | - | (0 studies) | - |  |
| Adverse events/harms | Study authors reported that no adverse events occurred (55 participants total). | |  | (1 RCT)^2,b^ | ⨁◯◯◯ Very low^f,g,h,i^ | Aerobic exercise does not contribute to adverse events/harms more than no treatment. |
| ***The risk in the intervention group** (and its 95% confidence interval) is based on the assumed risk in the comparison group and the **relative effect** of the intervention (and its 95% CI).  **CI:** confidence interval; **MD:** mean difference | | | | | | |
| **GRADE Working Group grades of evidence** **High certainty:** we are very confident that the true effect lies close to that of the estimate of the effect. **Moderate certainty:** we are moderately confident in the effect estimate: the true effect is likely to be close to the estimate of the effect, but there is a possibility that it is substantially different. **Low certainty:** our confidence in the effect estimate is limited: the true effect may be substantially different from the estimate of the effect. **Very low certainty:** we have very little confidence in the effect estimate: the true effect is likely to be substantially different from the estimate of effect. | | | | | | |

#### Explanations

a. Nardin 2022: individually supervised exercise; 8 30-minute sessions.

b. Rotter 2022: group supervised exercise; 8 60-min sessions.

c. Risk of bias: We downgraded once due to potential risk of bias (performance and detection domains).

d. Inconsistency: We did not downgrade. The point estimates are in the same direction with overlapping confidence intervals. Statistical heterogeneity is between 0% and 40%, which might not be important (i.e., I2 = 0%).

e. Indirectness: We did not downgrade because the trials were conducted in different countries (high or upper-middle income).

f. Imprecision: We downgraded twice. The sample size is small (OIS would not have been reached).

g. Risk of bias: We downgraded once due to potential risk of bias (performance domain).

h. Inconsistency: We did not downgrade; however, there are no other trials with which to compare findings.

i. Indirectness: We downgraded once. The trial was conducted in one country (high or upper-middle income).

#### References

1.DMK, Stocco Nardin MR, Aguiar AF, Machado FA, de Oliveira RG, Andraus RAC. Effects of photobiomodulation and deep water running in patients with chronic non-specific low back pain: a randomized controlled trial. . 2022.

2.Rotter G, Ortiz M, Binting S, et al. Mindful Walking in Patients with Chronic Low Back Pain: A Randomized Controlled Trial. 2022.

Summary of Findings table 2: ***What are the benefits and harms of core strengthening exercise in the management of community-dwelling adults (including older adults aged 60 years and over) with chronic primary low back pain (with or without leg pain) compared with no treatment/no additional treatment?***

| Outcomes | **Anticipated absolute effects^*^** (95% CI) | | Relative effect (95% CI) | № of participants (studies) | Certainty of the evidence (GRADE) | Comments |
| --- | --- | --- | --- | --- | --- | --- |
|  | **Risk with no intervention** | **Risk with core strengthening** |  |  |  |  |
| **ALL ADULTS** | | | | | | |
| **Pain**  0 to 10; 0 = no pain follow-up: closest to 2 weeks | The mean pain was **2.184** | MD **0.56 lower** (0.94 lower to 0.19 lower) | - | 80 (1 RCT)^1,a^ | ⨁◯◯◯ Very low^b,c,d,e^ | Core strengthening exercise reduces pain more than no treatment in the immediate term (may not reach clinically important threshold). |
| **Function** 0 to 24; 0 = no disability follow-up: closest to 2 weeks | The mean function was **7.3** | MD **1.7 lower** (2.42 lower to 0.98 lower) | - | 80 (1 RCT)^1,a^ | ⨁◯◯◯ Very low^b,c,d,e^ | Core strengthening exercise improves function more than no treatment in the immediate term (may not reach clinically important threshold). |
| **Adverse events/harms** | Authors reported that no adverse events occurred (80 participants total). | |  | (1 RCT)^1,a^ | ⨁◯◯◯ Very low^b,c,d,e^ | Core strengthening exercise does not contribute to adverse events/harms more than no treatment. |
| ***The risk in the intervention group** (and its 95% confidence interval) is based on the assumed risk in the comparison group and the **relative effect** of the intervention (and its 95% CI).  **CI:** confidence interval; **MD:** mean difference | | | | | | |
| **GRADE Working Group grades of evidence** **High certainty:** we are very confident that the true effect lies close to that of the estimate of the effect. **Moderate certainty:** we are moderately confident in the effect estimate: the true effect is likely to be close to the estimate of the effect, but there is a possibility that it is substantially different. **Low certainty:** our confidence in the effect estimate is limited: the true effect may be substantially different from the estimate of the effect. **Very low certainty:** we have very little confidence in the effect estimate: the true effect is likely to be substantially different from the estimate of effect. | | | | | | |

#### Explanations

a. Rahbar 2018: participants had individual supervised exercise; 15 15-minute sessions.

b. Risk of bias: We downgraded once due to potential risk of bias (performance and detection domains).

c. Inconsistency: We did not downgrade; however, there are no other trials with which to compare findings.

d. Indirectness: We downgraded once. The trial was conducted in one country (low or lower-middle income).

e. Imprecision: We downgraded twice. The sample size was small (OIS would not have been achieved).

#### References

1.Rahbar M, Salekzamani Y, Jahanjou F, Eslamian F, Niroumand A, Dolatkhah N. Effect of hippotherapy simulator on pain, disability, and range of motion of the spinal column in subjects with mechanical low back pain: A randomized single-blind clinical trial. 2018.

Summary of Findings table 3: ***What are the benefits and harms of muscle strength training in the management of community-dwelling adults (including older adults aged 60 years and over) with chronic primary low back pain (with or without leg pain) compared with no treatment/no additional treatment?***

| Outcomes | **Anticipated absolute effects^*^** (95% CI) | | Relative effect (95% CI) | № of participants (studies) | Certainty of the evidence (GRADE) | Comments |
| --- | --- | --- | --- | --- | --- | --- |
|  | **Risk with no intervention** | **Risk with muscle strength training** |  |  |  |  |
| **ALL ADULTS** | | | | | | |
| **Pain**  0 to 10; 0 = no pain follow-up: closest to 2 weeks | The mean pain was **2.607 to 2.9** | MD **0.39 lower** (1.16 lower to 0.38 higher) | - | 100 (2 RCTs)^1,2,a,b^ | ⨁◯◯◯ Very low^c,d,e,f^ | Muscle strength training does not reduce pain more than no treatment in the immediate term. |
| **Pain**  0 to 10; 0 = no pain follow-up: closest to 6 months | The mean pain was **3.9** | MD **0.4 lower** (1.67 lower to 0.87 higher) | - | 70 (1 RCT)^1,a^ | ⨁◯◯◯ Very low^c,f,g,h^ | Muscle strength training does not reduce pain more than no treatment in the intermediate term. |
| **Pain**  0 to 10; 0 = no pain follow-up: closest to 12 months | The mean pain (no leg pain, high-income country) was **0** | MD **0.1 lower** (1.32 lower to 1.12 higher) | - | 70 (1 RCT)^1,a^ | ⨁◯◯◯ Very low^c,f,g,h^ | Muscle strength training does not reduce pain more than no treatment in the long term. |
| **Function**  follow-up: closest to 2 weeks | The mean function was **4.3 (RMDQ, 0-24) to 19.433 (ODI, 0-100)** | SMD **0.05 higher** (0.34 lower to 0.45 higher) | - | 100 (2 RCTs)^1,2,a,b^ | ⨁◯◯◯ Very low^c,d,e,f^ | Muscle strength training does not improve function more than no treatment in the immediate term. |
| **Function**  0 to 24; 0 = no disability follow-up: closest to 6 months | The mean function was **5.7** | MD **0.6 lower** (3.2 lower to 2 higher) | - | 70 (1 RCT)^1,a^ | ⨁◯◯◯ Very low^c,f,g,h^ | Muscle strength training does not improve function more than no treatment in the intermediate term. |
| **Function**  0 to 24; 0 = no disability follow-up: closest to 12 months | The mean function was **4.7** | MD **0.2 lower** (2.73 lower to 2.33 higher) | - | 70 (1 RCT)^1,a^ | ⨁◯◯◯ Very low^c,f,g,h^ | Muscle strength training does not improve function more than no treatment in the long term. |
| ***The risk in the intervention group** (and its 95% confidence interval) is based on the assumed risk in the comparison group and the **relative effect** of the intervention (and its 95% CI).  **CI:** confidence interval; **MD:** mean difference; **SMD:** standardised mean difference | | | | | | |
| **GRADE Working Group grades of evidence** **High certainty:** we are very confident that the true effect lies close to that of the estimate of the effect. **Moderate certainty:** we are moderately confident in the effect estimate: the true effect is likely to be close to the estimate of the effect, but there is a possibility that it is substantially different. **Low certainty:** our confidence in the effect estimate is limited: the true effect may be substantially different from the estimate of the effect. **Very low certainty:** we have very little confidence in the effect estimate: the true effect is likely to be substantially different from the estimate of effect. | | | | | | |

#### Explanations

a. Fukuda 2021: participants had individual supervised exercise; 10 45-minute sessions.

b. Shamsi 2022: participants had individual supervised exercise; 12 sessions (session duration not reported).

c. Risk of bias: We downgraded once due to potential risk of bias (performance and detection domains).

d. Inconsistency: We did not downgrade. The point estimates are in the same direction with overlapping confidence intervals. Statistical heterogeneity is between 0% and 40%, which might not be important (i.e., I2 = 0%).

e. Indirectness: We did not downgrade. The trials were conducted in different countries (high- and low-income).

f. Imprecision: We downgraded twice. Sample size was small (OIS would not have been reached).

g. Inconsistency: We did not downgrade; however, there are no other trials with which to compare findings.

h. Indirectness: We downgraded once. The trial was conducted in one country (high or upper-middle income).

i. Indirectness: We downgraded once. The trial was conducted in one country (low or lower-middle income).

#### References

1.Fukuda TY, Aquino LM, Pereira P, et al. Does adding hip strengthening exercises to manual therapy and segmental stabilization improve outcomes in patients with nonspecific low back pain? A randomized controlled trial. 2021.

2.Shamsi M, Ahmadi A, Mirzaei M, Jaberzadeh S. Effects of static stretching and strengthening exercises on flexion relaxation ratio in patients with LBP: A randomized clinical trial. 2022.

Summary of Findings table 4: ***What are the benefits and harms of mixed exercise in the management of community-dwelling adults (including older adults aged 60 years and over) with chronic primary low back pain (with or without leg pain) compared with no treatment/no additional treatment?***

| Outcomes | **Anticipated absolute effects^*^** (95% CI) | | Relative effect (95% CI) | № of participants (studies) | Certainty of the evidence (GRADE) | Comments |
| --- | --- | --- | --- | --- | --- | --- |
|  | **Risk with no intervention** | **Risk with mixed exercise** |  |  |  |  |
| **ALL ADULTS** | | | | | | |
| **Pain** follow-up: closest to 2 weeks | The mean pain was **8.4** (MPQ, 0-45) **to 36.83** (VAS, 0-100) | SMD **0.01 lower** (0.32 lower to 0.31 higher) | - | 250 (2 RCTs)^1,2,a,b,c^ | ⨁◯◯◯ Very low^d,e,f,g^ | Mixed exercise does not reduce pain more than no treatment in the immediate term. |
| **Pain**  0 to 10; 0 = no pain follow-up: closest to 3 months | The mean pain was **4.092** | MD **0.1 lower** (1.34 lower to 1.14 higher) | - | 50 (1 RCT)^1,a^ | ⨁◯◯◯ Very low^d,h,i,j^ | Mixed exercise does not reduce pain more than no treatment in the short term. |
| **Pain** follow-up: closest to 6 months | The mean pain was **7.4** (MPQ, 0-45) **to 41.32** (VAS, 0-100) | SMD **0.03 higher** (0.23 lower to 0.29 higher) | - | 248 (2 RCTs)^1,2,a,b,k^ | ⨁◯◯◯ Very low^d,f,g,l^ | Mixed exercise does not reduce pain more than no treatment in the immediate term. |
| **Pain** 0-100 follow-up: closest to 12 months | Between-group (graded activity with problem solving training alone vs. combination treatment) MD = 8.88, 95% CI -0.36 to 18.13 (119 participants total). | |  | (1 RCT)^3,m^ | ⨁◯◯◯ Very low^h,i,j,n^ | Mixed exercise does not reduce pain more than no treatment in the long term. |
| **Function**  follow-up: closest to 2 weeks | The mean function was **6.15** (WI, 0-9) **to 7.9** (RMDQ, 0-24) | SMD **0.15 lower** (0.48 lower to 0.18 higher) | - | 250 (2 RCTs)^1,2,a,b,o^ | ⨁⨁◯◯ Low^d,f,p,q^ | Mixed exercise does not improve function more than no treatment in the immediate term. |
| **Function** 0 to 9; 0 = no disability follow-up: closest to 3 months | The mean function was **5.58** | MD **1.25 lower** (2.79 lower to 0.29 higher) | - | 50 (1 RCT)^1,a^ | ⨁◯◯◯ Very low^d,h,i,j^ | Mixed exercise does not improve function more than no treatment in the short term. |
| **Function**  follow-up: closest to 6 months | The mean function was **6.46** (WI, 0-9) **to 8.4** (RMDQ, 0-24) | SMD **0.09 lower** (0.42 lower to 0.24 higher) | - | 248 (2 RCTs)^1,2,a,b,r^ | ⨁◯◯◯ Very low^d,f,g,p^ | Mixed exercise does not improve function more than no treatment in the intermediate term. |
| **Function** 0 to 24; 0 = no disability follow-up: closest to 12 months | Between-group (graded activity with problem solving training alone vs. combination treatment) MD = 1.62, 95% CI -0.06 to 3.31 (119 participants total). | |  | (1 RCT)^3,m^ | ⨁◯◯◯ Very low^h,i,j,n^ | Mixed exercise does not improve function more than no treatment in the long term. |
| **Health-related quality of life** 0 to 3; 0 = poor QofL follow-up: closest to 2 weeks | The mean health-related quality of life was **0.58** | MD **0.24 higher** (0.06 lower to 0.54 higher) | - | 50 (1 RCT)^1,a^ | ⨁◯◯◯ Very low^d,h,i,j^ | Mixed exercise does not improve health-related quality of life more than no treatment in the immediate term. |
| **Health-related quality of life**  (physical component) 0 to 100; 0 = poor QofL follow-up: closest to 2 weeks | The mean health-related quality of life was **59.3 to 62.2** | MD **6.56 lower** (13.03 lower to 0.1 lower) | - | 200 (1 RCT)^2,b^ | ⨁◯◯◯ Very low^d,h,i,q^ | Mixed exercise does not improve health-related quality of life (physical component) more than no treatment in the immediate term. |
| **Health-related quality of life**  mental component) 0 to 100; 0 = poor QofL follow-up: closest to 2 weeks | The mean health-related quality of life was **90.3 to 90.8** | MD **1.05 lower** (4.38 lower to 2.28 higher) | - | 200 (1 RCT)^2,b^ | ⨁◯◯◯ Very low^d,h,i,q^ | Mixed exercise does not improve health-related quality of life (mental component) more than no treatment in the immediate term. |
| **Health-related quality of life** 0 to 3; 0 = poor QofL follow-up: closest to 3 months | The mean health-related quality of life was **0.6** | MD **0.17 higher** (0.07 lower to 0.41 higher) | - | 50 (1 RCT)^1,a^ | ⨁◯◯◯ Very low^d,h,i,j^ | Mixed exercise does not improve health-related quality of life more than no treatment in the short term. |
| **Health-related quality of life** 0 to 3; 0 = poor QofL follow-up: closest to 6 months | The mean health-related quality of life was **0.67** | MD **0.19 higher** (0.09 lower to 0.47 higher) | - | 48 (1 RCT)^1,a^ | ⨁◯◯◯ Very low^d,h,i,j^ | Mixed exercise does not improve health-related quality of life more than no treatment in the intermediate term. |
| **Health-related quality of life**  physical component) 0 to 100; 0 = poor QofL follow-up: closest to 6 months | The mean health-related quality of life was **54.5 to 61.4** | MD **2.31 lower** (9.33 lower to 4.7 higher) | - | 200 (1 RCT)^2,b^ | ⨁◯◯◯ Very low^d,h,i,q^ | Mixed exercise does not improve health-related quality of life (physical component) more than no treatment in the intermediate term. |
| **Health-related quality of life**  mental component) 0 to 100; 0 = poor QofL follow-up: closest to 6 months | The mean health-related quality of life was **87 to 92.1** | MD **0.83 lower** (8.67 lower to 7 higher) | - | 200 (1 RCT)^2,b^ | ⨁◯◯◯ Very low^d,h,i,q^ | Mixed exercise does not improve health-related quality of life (mental component) more than no treatment in the intermediate term. |
| **Depression** 0 to 30; 0 = no depression follow-up: closest to 2 weeks | The mean depression was **4 to 4.9** | MD **0.11 lower** (1.87 lower to 1.66 higher) | - | 200 (1 RCT)^2,b,s^ | ⨁◯◯◯ Very low^d,h,i,q^ | Mixed exercise does not reduce depression more than no treatment in the intermediate term. |
| **Depression** 0 to 30; 0 = no depression follow-up: closest to 6 months | The mean depression was **4 to 4.9** | MD **0.14 higher** (1.92 lower to 2.2 higher) | - | 200 (1 RCT)^2,b,t^ | ⨁◯◯◯ Very low^d,h,i,q^ | Mixed exercise does not reduce depression more than no treatment in the intermediate term. |
| **Depression** 0 to 63; 0 = no depression follow-up: closest to 12 months | Between-group (graded activity with problem solving training alone vs. combination treatment) MD = -0.09, 95% CI -2.11 to 1.93 (119 participants total). | |  | (1 RCT)^3,m^ | ⨁◯◯◯ Very low^h,i,j,n^ | Mixed exercise does not reduce depression more than no treatment in the long term. |
| **Self-efficacy** 10 to 100; 0 = no self-efficacy; follow-up: closest to 2 weeks | Between-group change score: 2.1 (SE 3.1), p=0.50 (no significant difference between groups) (200 participants total). | |  | (1 RCT)^2,u^ | ⨁◯◯◯ Very low^d,h,i,q^ | Mixed exercise does not improve self-efficacy more than no treatment in the immediate term. |
| **Self-efficacy** 10 to 100; 0 = no self-efficacy; follow-up: closest to 6 months | Between-group change score: -0.8 (SE 3.2), p=0.80 (no significant difference between groups) (200 participants total). | |  | (1 RCT)^2,u^ | ⨁◯◯◯ Very low^d,h,i,q^ | Mixed exercise does not improve self-efficacy more than no treatment in the intermediate term. |
| **Catastrophizing** 0 to 6; 0 = no catastrophizing; follow-up: closest to 2 weeks | Between-group change score: -0.1 (SE 0.2), p=0.35 (no significant difference between groups) (200 participants total). | |  | (1 RCT)^2,u^ | ⨁◯◯◯ Very low^d,h,i,q^ | Mixed exercise does not reduce catastrophizing more than no treatment in the immediate term. |
| **Catastrophizing** 0 to 6; 0 = no catastrophizing; follow-up: closest to 6 months | Between-group change score: -0.2 (SE 0.2), p=0.14 (no significant difference between groups) (200 participants total). | |  | (1 RCT)^2,u^ | ⨁◯◯◯ Very low^d,h,i,q^ | Mixed exercise does not reduce catastrophizing more than no treatment in the intermediate term. |
| **Fear avoidance** 0 to 24; 0 = no fear avoidance behaviours; follow-up: closest to 2 weeks | Clinically unimportant but statistically significant difference in between-group change scores (comparison 1: -0.6 SE 0.3; p=0.0426; ES=0.37; comparison 2: -0.9 SE 0.3; p=0.0039; ES=0.54) favouring mixed exercise (200 participants total). | |  | (1 RCT)^2,u^ | ⨁◯◯◯ Very low^d,h,i,q^ | Mixed exercise does not reduce fear avoidance more than no treatment in the immediate term. |
| **Fear avoidance** 0 to 24; 0 = no fear avoidance behaviours; follow-up: closest to 6 months | Between-group change score: -0.3 (SE 0.3), p=0.38 (no significant difference between groups) (200 participants total). | |  | (1 RCT)^2,u^ | ⨁◯◯◯ Very low^d,h,i,q^ | Mixed exercise does not reduce fear avoidance more than no treatment in the intermediate term. |
| **Adverse events/harms** | 0 per 1,000 | **20 - 55 per 1,000** (0.12 to 76.95 – 0.37 to 146.73) | **OR 4.24** (0.69 to 25.95) | 310 (2 RCTs)^2,3,b,v,w^ | ⨁◯◯◯ Very low^d,f,x,y^ | Mixed exercise does not contribute to adverse events/harms more than no treatment. |
| **OLDER ADULTS** | | | | | | |
| **Pain** follow-up: closest to 2 weeks | The mean pain was **8.4** (MPQ, 0-45) **to 10.5** (MPQ, 0-45) | SMD **0.1 lower** (0.44 lower to 0.23 higher) | - | 200 (1 RCT)^2,b^ | ⨁◯◯◯ Very low^d,g,h,i^ | Mixed exercise does not reduce pain more than no treatment in the immediate term. |
| **Pain** follow-up: closest to 6 months | The mean pain was **7.4** (MPQ, 0-45) **to 10** (MPQ, 0-45) | SMD **0.01 higher** (0.39 lower to 0.4 higher) | - | 200 (1 RCT)^2,b^ | ⨁◯◯◯ Very low^d,g,h,i^ | Mixed exercise does not reduce pain more than no treatment in the intermediate term. |
| **Function**  follow-up: closest to 2 weeks | The mean function was **7.8** (RMDQ, 0-24) **to 7.9** (RMDQ, 0-24) | SMD **0.01 lower** (0.29 lower to 0.27 higher) | - | 200 (1 RCT)^2,b^ | ⨁◯◯◯ Very low^d,g,h,i^ | Mixed exercise does not improve function more than no treatment in the immediate term. |
| **Function**  follow-up: closest to 6 months | The mean function was **7.5** (RMDQ, 0-24) **to 8.4** (RMDQ, 0-24) | SMD **0.03 higher** (0.24 lower to 0.31 higher) | - | 200 (1 RCT)^2,b^ | ⨁◯◯◯ Very low^d,g,h,i^ | Mixed exercise does not improve function more than no treatment in the intermediate term. |
| **Health-related quality of life**  (physical component) 0 to 100; 0 = poor QofL follow-up: closest to 2 weeks | The mean health-related quality of life was **59.3 to 62.2** | MD **6.56 lower** (13.03 lower to 0.1 lower) | - | 200 (1 RCT)^2,b^ | ⨁◯◯◯ Very low^d,h,i,q^ | Mixed exercise does not improve health-related quality of life (physical component) more than no treatment in the immediate term. |
| **Health-related quality of life**  mental component) 0 to 100; 0 = poor QofL follow-up: closest to 2 weeks | The mean health-related quality of life was **90.3 to 90.8** | MD **1.05 lower** (4.38 lower to 2.28 higher) | - | 200 (1 RCT)^2,b^ | ⨁◯◯◯ Very low^d,h,i,q^ | Mixed exercise does not improve health-related quality of life (mental component) more than no treatment in the immediate term. |
| **Health-related quality of life**  physical component) 0 to 100; 0 = poor QofL follow-up: closest to 6 months | The mean health-related quality of life was **54.5 to 61.4** | MD **2.31 lower** (9.33 lower to 4.7 higher) | - | 200 (1 RCT)^2,b^ | ⨁◯◯◯ Very low^d,h,i,q^ | Mixed exercise does not improve health-related quality of life (physical component) more than no treatment in the intermediate term. |
| **Health-related quality of life**  mental component) 0 to 100; 0 = poor QofL follow-up: closest to 6 months | The mean health-related quality of life was **87 to 92.1** | MD **0.83 lower** (8.67 lower to 7 higher) | - | 200 (1 RCT)^2,b^ | ⨁◯◯◯ Very low^d,h,i,q^ | Mixed exercise does not improve health-related quality of life (mental component) more than no treatment in the intermediate term. |
| **Depression** 0 to 30; 0 = no depression follow-up: closest to 2 weeks | The mean depression was **4.2 to 4.7** | MD **0.11 lower** (1.87 lower to 1.66 higher) | - | 200 (1 RCT)^2,b^ | ⨁◯◯◯ Very low^d,h,i,q^ | Mixed exercise does not reduce depression more than no treatment in the immediate term. |
| **Depression** 0 to 30; 0 = no depression follow-up: closest to 6 months | The mean depression was **4 to 4.9** | MD **0.14 higher** (1.92 lower to 2.2 higher) | - | 200 (1 RCT)^2,b^ | ⨁◯◯◯ Very low^d,h,i,q^ | Mixed exercise does not reduce depression more than no treatment in the intermediate term. |
| **Self-efficacy** 10 to 100; 0 = no self-efficacy; follow-up: closest to 2 weeks | Between-group change score: 2.1 (SE 3.1), p=0.50 (no significant difference between groups) (200 participants total). | |  | (1 RCT)^2,u^ | ⨁◯◯◯ Very low^d,h,i,q^ | Mixed exercise does not improve self-efficacy more than no treatment in the immediate term. |
| **Self-efficacy** 10 to 100; 0 = no self-efficacy; follow-up: closest to 6 months | Between-group change score: -0.8 (SE 3.2), p=0.80 (no significant difference between groups) (200 participants total). | |  | (1 RCT)^2,u^ | ⨁◯◯◯ Very low^d,h,i,q^ | Mixed exercise does not improve self-efficacy more than no treatment in the intermediate term. |
| **Catastrophizing** 0 to 6; 0 = no catastrophizing; follow-up: closest to 2 weeks | Between-group change score: -0.1 (SE 0.2), p=0.35 (no significant difference between groups) (200 participants total). | |  | (1 RCT)^2,u^ | ⨁◯◯◯ Very low^d,h,i,q^ | Mixed exercise does not reduce catastrophizing more than no treatment in the immediate term. |
| **Catastrophizing** 0 to 6; 0 = no catastrophizing; follow-up: closest to 6 months | Between-group change score: -0.2 (SE 0.2), p=0.14 (no significant difference between groups) (200 participants total). | |  | (1 RCT)^2,u^ | ⨁◯◯◯ Very low^d,h,i,q^ | Mixed exercise does not reduce catastrophizing more than no treatment in the intermediate term. |
| **Fear avoidance** 0 to 24; 0 = no fear avoidance behaviours; follow-up: closest to 2 weeks | Clinically unimportant but statistically significant difference in between-group change scores (comparison 1: -0.6 SE 0.3; p=0.0426; ES=0.37; comparison 2: -0.9 SE 0.3; p=0.0039; ES=0.54) favouring mixed exercise (200 participants total). | |  | (1 RCT)^2,u^ | ⨁◯◯◯ Very low^d,h,i,q^ | Mixed exercise does not reduce fear avoidance more than no treatment in the immediate term. |
| **Fear avoidance** 0 to 24; 0 = no fear avoidance behaviours; follow-up: closest to 6 months | Between-group change score: -0.3 (SE 0.3), p=0.38 (no significant difference between groups) (200 participants total). | |  | (1 RCT)^2,u^ | ⨁◯◯◯ Very low^d,h,i,q^ | Mixed exercise does not reduce fear avoidance more than no treatment in the intermediate term. |
| **Performance-based physical functioning** follow-up: closest to 2 weeks | Between-group change scores (200 participants total):  Usual pace gait speed (25 meters) (meters/second): no significant difference between groups (0.02 (SE 0.01), p=0.29). Chair raise time (5 consecutive raises) (seconds): small but statistically significant difference favouring mixed exercise for comparison 2 (-0.8 (SE 0.3), p=0.008, ES=0.29).  Stair climb time (seconds): no significant difference between groups (-0.0 (SE 1.2), p=0.99). | |  | (1 RCT)^2,u^ | ⨁◯◯◯ Very low^d,h,i,q^ | Mixed exercise does not improve performance-based physical functioning more than no treatment in the immediate term. |
| **Performance-based physical functioning** follow-up: closest to 6 months | Between-group change scores (200 participants total): Usual pace gait speed (25 meters) (meters/second): no significant difference between groups (0.00 (SE 0.02), p=0.92). Chair raise time (5 consecutive raises) (seconds): no significant difference between groups (0.1 (SE 0.3), p=0.88). Stair climb time (seconds): no significant difference between groups (-0.6 (SE 1.2), p=0.61). | |  | (1 RCT)^2,u^ | ⨁◯◯◯ Very low^d,h,i,q^ | Mixed exercise does not improve performance-based physical functioning more than no treatment in the intermediate term. |
| Adverse events/harms (either with or without leg pain, high to upper-middle income country) | 0 per 1,000 | **20 per 1,000** (0.12 to 76.95) | **OR 3.06** (0.31 to 29.93) | 200 (1 RCT)^2,b,z^ | ⨁◯◯◯ Very low^d,h,i,y^ | Mixed exercise does not contribute to adverse events/harms more than no treatment. |
| ***The risk in the intervention group** (and its 95% confidence interval) is based on the assumed risk in the comparison group and the **relative effect** of the intervention (and its 95% CI).  **CI:** confidence interval; **MD:** mean difference; **OR:** odds ratio; **SMD:** standardised mean difference | | | | | | |
| **GRADE Working Group grades of evidence** **High certainty:** we are very confident that the true effect lies close to that of the estimate of the effect. **Moderate certainty:** we are moderately confident in the effect estimate: the true effect is likely to be close to the estimate of the effect, but there is a possibility that it is substantially different. **Low certainty:** our confidence in the effect estimate is limited: the true effect may be substantially different from the estimate of the effect. **Very low certainty:** we have very little confidence in the effect estimate: the true effect is likely to be substantially different from the estimate of effect. | | | | | | |

#### Explanations

a. Takinaci 2019: participants had general strength training, stretching, or flexibility/mobilizing exercises; group supervised; 12 sessions (session duration was not reported).

b. Weiner 2008: had 2 comparisons (both included in meta-analysis). Participants had strength, flexibility, aerobic exercises; individual supervision; 12 sessions (session duration was not reported).

c. Smeets 2008 was not included in the meta-analysis (provided within-group mean changes; no follow-up scores). 119 participants total, rated as overall low risk of bias. Participants performed combination treatment (active physical treatment [aerobic and core strengthening exercises] + graded activity with problem solving training) vs. graded activity with problem solving training alone. Intervention was group supervised; 30 105-minute sessions. Between-group MD (VAS 0-100) graded activity with problem solving training alone vs. combination treatment = 5.35, 95% CI -3.73 to 14.42.

d. Risk of bias: We downgraded once due to potential risk of bias (performance and detection domains).

e. Inconsistency: We did not downgrade. The confidence intervals overlapped, and statistical heterogeneity is between 0% and 40%, which might not be important (i.e., I2 = 34%).

f. Indirectness: We did not downgrade because trials were conducted in different countries (high or upper-middle income).

g. Imprecision: We downgraded twice. The point estimate did not reach the pre-specified threshold for what may be considered clinically important (SMD ≥ 0.2). The lower boundary of the 95% CI crosses the threshold for what may be considered appreciable benefit (-0.2), and the upper boundary crosses the threshold for what may be considered appreciable harm (+0.2).

h. Inconsistency: We did not downgrade; however, there are no other trials with which to compare findings.

i. Indirectness: We downgraded once. The trial was conducted in one country (high or upper-middle income).

j. Imprecision: We downgraded twice. The sample size was small (OIS would not have been reached).

k. Smeets 2008 was not included in the meta-analysis (provided within-group mean changes; no follow-up scores). 119 participants total, rated as overall low risk of bias. Participants performed combination treatment (active physical treatment [aerobic and core strengthening exercises] + graded activity with problem solving training) vs. graded activity with problem solving training alone. Intervention was group supervised; 30 105-minute sessions. Between-group MD (VAS 0-100) graded activity with problem solving training alone vs. combination treatment = 6.25, 95% CI -2.94 to 15.44.

l. Inconsistency: We did not downgrade. The confidence intervals overlapped, and statistical heterogeneity is between 0% and 40%, which might not be important (i.e., I2 = 6%).

m. Smeets 2008 was not included in the meta-analysis (provided within-group mean changes; no follow-up scores). Participants performed combination treatment (active physical treatment [aerobic and core strengthening exercises] + graded activity with problem solving training) vs. graded activity with problem solving training alone. Intervention was group supervised; 30 105-minute sessions.

n. Risk of bias: We did not downgrade because the trial was rated as overall low risk of bias.

o. Smeets 2008 was not included in the meta-analysis (provided within-group mean changes; no follow-up scores). 119 participants total, rates as overall low risk of bias. Participants performed combination treatment (active physical treatment [aerobic and core strengthening exercises] + graded activity with problem solving training) vs. graded activity with problem solving training alone. Intervention was group supervised; 30 105-minute sessions. Between-group MD (RMDQ 0-24) graded activity with problem solving training alone vs. combination treatment = 0.58, 95% CI -1.08 to 2.24.

p. Inconsistency: We did not downgrade. The confidence intervals overlapped, and statistical heterogeneity is between 0% and 40%, which might not be important (i.e., I2 = 40%).

q. Imprecision: We downgraded once. The sample size was small (OIS would not have been reached).

r. Smeets 2008 was not included in the meta-analysis (provided within-group mean changes; no follow-up scores). 119 participants total, rated as overall low risk of bias. Participants performed combination treatment (active physical treatment [aerobic and core strengthening exercises] + graded activity with problem solving training) vs. graded activity with problem solving training alone. Intervention was group supervised; 30 105-minute sessions. Between-group MD (RMDQ 0-24) graded activity with problem solving training alone vs. combination treatment = 1.11, 95% CI -0.56 to 2.79.

s. Smeets 2008 was not included in the meta-analysis (provided within-group mean changes; no follow-up scores). 119 participants total; rated as overall low risk of bias. Participants performed combination treatment (active physical treatment [aerobic and core strengthening exercises] + graded activity with problem solving training) vs. graded activity with problem solving training alone. Intervention was group supervised; 30 105-minute sessions. Between-group MD (BDI 0-63) graded activity with problem solving training alone vs. combination treatment = 1.62, 95% CI -0.36 to 3.61.

t. Smeets 2008 was not included in the meta-analysis (provided within-group mean changes; no follow-up scores). 119 participants total, rated as overall low risk of bias. Participants performed combination treatment (active physical treatment [aerobic and core strengthening exercises] + graded activity with problem solving training) vs. graded activity with problem solving training alone. Intervention was group supervised; 30 105-minute sessions. Between-group MD (BDI 0-63) graded activity with problem solving training alone vs. combination treatment = 0.26, 95% CI -1.74 to 2.27.

u. Weiner 2008: participants had strength, flexibility, aerobic exercises; individual supervision; 12 sessions (session duration was not reported). Only within and between-group change scores were reported.

v. Smeets 2008: participants performed combination treatment (active physical treatment [aerobic and core strengthening exercises] + graded activity with problem solving training) vs. graded activity with problem solving training alone. Intervention was group supervised; 30 105-minute sessions.

w. Adverse events: were mainly minor and included back and knee pain.

x. Inconsistency: We did not downgrade. The point estimates are similar with overlapping confidence intervals. Statistical heterogeneity is between 0% and 40%, which might not be important (i.e., I2 = 0%).

y. Imprecision: We downgraded twice due to small sample size and number of events.

z. Adverse events: included back pain. Authors reported no significant intervention-associated adverse events.

#### References

1.Z, Takinaci. Clinical efficacy of spa therapy (balneotherapy) for chronic low back pain: A randomized single-blind trial. 2019.

2.Weiner DK, Perera S, Rudy TE, Glick RM, Shenoy S, Delitto A. Efficacy of percutaneous electrical nerve stimulation and therapeutic exercise for older adults with chronic low back pain: a randomized controlled trial. 2008.

3.Smeets R, Vlaeyen JWS, Hidding A, Kester ADM, van der Heijden G, Knottnerus AJ. Chronic low back pain: physical training, graded activity with problem solving training, or both? The one-year post-treatment results of a randomized controlled trial. 2008.

Summary of Findings table 5: ***What are the benefits and harms of mixed exercise in the management of community-dwelling adults (including older adults aged 60 years and over) with chronic primary low back pain (with or without leg pain) compared with usual care?***

| Outcomes | **Anticipated absolute effects^*^** (95% CI) | | Relative effect (95% CI) | № of participants (studies) | Certainty of the evidence (GRADE) | Comments |
| --- | --- | --- | --- | --- | --- | --- |
|  | **Risk with usual care** | **Risk with mixed exercise** |  |  |  |  |
| **ALL ADULTS** | | | | | | |
| **Pain**  0 to 10; 0 = no pain follow-up: closest to 2 weeks | The mean pain was **3.2 to 4** | MD **0.12 lower** (0.91 lower to 0.68 higher) | - | 142 (2 RCTs)^1,2,a,b^ | ⨁◯◯◯ Very low^c,d,e,f^ | Mixed exercise does not reduce pain more than usual care in the immediate term. |
| **Pain**  0 to 10; 0 = no pain follow-up: closest to 3 months | The mean pain was **3.5** | MD **0.3 lower** (1.66 lower to 1.06 higher) | - | 48 (1 RCT)^2,b^ | ⨁◯◯◯ Very low^c,f,g,h^ | Mixed exercise does not reduce pain more than usual care in the short term. |
| **Pain**  0 to 10; 0 = no pain follow-up: closest to 6 months | The mean pain was **4** | MD **0**  (1.26 lower to 1.26 higher) | - | 48 (1 RCT)^2,b^ | ⨁◯◯◯ Very low^c,f,g,h^ | Mixed exercise does not reduce pain more than usual care in the intermediate term. |
| **Function**  follow-up: closest to 2 weeks | The mean function was 5.4 (RMDQ, 0-24) to 29.9 (ODI, 0-100) | SMD **0.62 lower** (0.96 lower to 0.28 lower) | - | 142 (2 RCTs)^1,2,a,b^ | ⨁◯◯◯ Very low^c,d,e,f^ | Mixed exercise improves function more than usual care in the immediate term. |
| **Function** 0 to 24; 0 = no disability follow-up: closest to 3 months | The mean function was **4.5** | MD **2.3 lower** (4.92 lower to 0.32 higher) | - | 48 (1 RCT)^2,b^ | ⨁◯◯◯ Very low^c,f,g,h^ | Mixed exercise does not improve function more than usual care in the short term. |
| **Function** 0 to 24; 0 = no disability follow-up: closest to 6 months | The mean function was **5.1** | MD **2.5 lower** (5.19 lower to 0.19 higher) | - | 48 (1 RCT)^2,b^ | ⨁◯◯◯ Very low^c,f,g,h^ | Mixed exercise does not improve function more than usual care in the intermediate term. |
| **Health-related quality of life**  0 to 1; 0 = poor QofL follow-up: closest to 2 weeks | The mean health-related quality of life was **0.82** | MD **0.05 higher** (0.01 lower to 0.11 higher) | - | 49 (1 RCT)^2,b^ | ⨁◯◯◯ Very low^c,f,g,h^ | Mixed exercise does not improve health-related quality of life more than usual care in the immediate term. |
| **Health-related quality of life**  0 to 1; 0 = poor QofL follow-up: closest to 3 months | The mean health-related quality of life was **0.84** | MD **0.04 higher** (0 to 0.08 higher) | - | 48 (1 RCT)^2,b^ | ⨁◯◯◯ Very low^c,f,g,h^ | Mixed exercise does not improve health-related quality of life more than usual care in the short term. |
| **Health-related quality of life**  0 to 1; 0 = poor QofL follow-up: closest to 6 months | The mean health-related quality of life was **0.81** | MD **0.05 higher** (0 to 0.1 higher) | - | 48 (1 RCT)^2,b^ | ⨁◯◯◯ Very low^c,f,g,h^ | Mixed exercise does not improve health-related quality of life more than usual care in the intermediate term. |
| **Self-efficacy**  0 to 60; 0 = no self-efficacy follow-up: closest to 2 weeks | The mean self-efficacy was **45** | MD **3 higher** (2.39 lower to 8.39 higher) | - | 49 (1 RCT)^2,b^ | ⨁◯◯◯ Very low^c,f,g,h^ | Mixed exercise does not improve self-efficacy more than usual care in the immediate term. |
| **Self-efficacy**  0 to 60; 0 = no self-efficacy follow-up: closest to 3 months | The mean self-efficacy was **47** | MD **3 higher** (1.63 lower to 7.63 higher) | - | 48 (1 RCT)^2,b^ | ⨁◯◯◯ Very low^c,f,g,h^ | Mixed exercise does not improve self-efficacy more than usual care in the short term. |
| **Self-efficacy**  0 to 60; 0 = no self-efficacy follow-up: closest to 6 months | The mean self-efficacy was **43** | MD **4 higher** (3.81 lower to 11.81 higher) | - | 48 (1 RCT)^2,b^ | ⨁◯◯◯ Very low^c,f,g,h^ | Mixed exercise does not improve self-efficacy more than usual care in the intermediate term. |
| **OLDER ADULTS (aged 60 years or more)** | | | | | | |
| **Pain**  0 to 10; 0 = no pain follow-up: closest to 2 weeks | The mean pain was **4** | MD **0.8 lower** (2.42 lower to 0.82 higher) | - | 49 (1 RCT)^2,b^ | ⨁◯◯◯ Very low^c,f,g,h^ | Mixed exercise does not reduce pain more than usual care in the immediate term. |
| **Pain**  0 to 10; 0 = no pain follow-up: closest to 3 months | The mean pain was **3.5** | MD **0.3 lower** (1.66 lower to 1.06 higher) | - | 48 (1 RCT)^2,b^ | ⨁◯◯◯ Very low^c,f,g,h^ | Mixed exercise does not reduce pain more than usual care in the short term. |
| **Pain**  0 to 10; 0 = no pain follow-up: closest to 6 months | The mean pain was **4** | MD **0**  (1.26 lower to 1.26 higher) | - | 48 (1 RCT)^2,b^ | ⨁◯◯◯ Very low^c,f,g,h^ | Mixed exercise does not reduce pain more than usual care in the intermediate term. |
| **Function**  follow-up: closest to 2 weeks | The mean function was **5.4** (RMDQ, 0-24) | SMD **0.86 SD lower** (1.45 lower to 0.27 lower) | - | 49 (1 RCT)^2,b^ | ⨁◯◯◯ Very low^c,f,g,h^ | Mixed exercise improves function more than usual care in the immediate term. |
| **Function** 0 to 24; 0 = no disability follow-up: closest to 3 months | The mean function was **4.5** | MD **2.3 lower** (4.92 lower to 0.32 higher) | - | 48 (1 RCT)^2,b^ | ⨁◯◯◯ Very low^c,f,g,h^ | Mixed exercise does not improve function more than usual care in the short term. |
| **Function** 0 to 24; 0 = no disability follow-up: closest to 6 months | The mean function was **5.1** | MD **2.5 lower** (5.19 lower to 0.19 higher) | - | 48 (1 RCT)^2,b^ | ⨁◯◯◯ Very low^c,f,g,h^ | Mixed exercise does not improve function more than usual care in the intermediate term. |
| **Health-related quality of life**  0 to 1; 0 = poor QofL follow-up: closest to 2 weeks | The mean health-related quality of life was **0.82** | MD **0.05 higher** (0.01 lower to 0.11 higher) | - | 49 (1 RCT)^2,b^ | ⨁◯◯◯ Very low^c,f,g,h^ | Mixed exercise does not improve health-related quality of life more than usual care in the immediate term. |
| **Health-related quality of life**  0 to 1; 0 = poor QofL follow-up: closest to 3 months | The mean health-related quality of life was **0.84** | MD **0.04 higher** (0 to 0.08 higher) | - | 48 (1 RCT)^2,b^ | ⨁◯◯◯ Very low^c,f,g,h^ | Mixed exercise does not improve health-related quality of life more than usual care in the short term. |
| **Health-related quality of life**  0 to 1; 0 = poor QofL follow-up: closest to 6 months | The mean health-related quality of life was **0.81** | MD **0.05 higher** (0 to 0.1 higher) | - | 48 (1 RCT)^2,b^ | ⨁◯◯◯ Very low^c,f,g,h^ | Mixed exercise does not improve health-related quality of life more than usual care in the intermediate term. |
| **Self-efficacy**  0 to 60; 0 = no self-efficacy follow-up: closest to 2 weeks | The mean self-efficacy was **45** | MD **3 higher** (2.39 lower to 8.39 higher) | - | 49 (1 RCT)^2,b^ | ⨁◯◯◯ Very low^c,f,g,h^ | Mixed exercise does not improve self-efficacy more than usual care in the immediate term. |
| **Self-efficacy**  0 to 60; 0 = no self-efficacy follow-up: closest to 3 months | The mean self-efficacy was **47** | MD **3 higher** (1.63 lower to 7.63 higher) | - | 48 (1 RCT)^2,b^ | ⨁◯◯◯ Very low^c,f,g,h^ | Mixed exercise does not improve self-efficacy more than usual care in the short term. |
| **Self-efficacy**  0 to 60; 0 = no self-efficacy follow-up: closest to 6 months | The mean self-efficacy was **43** | MD **4 higher** (3.81 lower to 11.81 higher) | - | 48 (1 RCT)^2,b^ | ⨁◯◯◯ Very low^c,f,g,h^ | Mixed exercise does not improve self-efficacy more than usual care in the intermediate term. |
| ***The risk in the intervention group** (and its 95% confidence interval) is based on the assumed risk in the comparison group and the **relative effect** of the intervention (and its 95% CI).  **CI:** confidence interval; **MD:** mean difference; **SMD:** standardised mean difference | | | | | | |
| **GRADE Working Group grades of evidence** **High certainty:** we are very confident that the true effect lies close to that of the estimate of the effect. **Moderate certainty:** we are moderately confident in the effect estimate: the true effect is likely to be close to the estimate of the effect, but there is a possibility that it is substantially different. **Low certainty:** our confidence in the effect estimate is limited: the true effect may be substantially different from the estimate of the effect. **Very low certainty:** we have very little confidence in the effect estimate: the true effect is likely to be substantially different from the estimate of effect. | | | | | | |

#### Explanations

a. Chhabra 2018: participants received home exercise with follow-up

b. Jinnouchi 2020: participants received home exercise with follow-up

c. Risk of bias: We downgraded once due to potential risk of bias (performance and detection domains).

d. Inconsistency: We did not downgrade. The confidence intervals overlap; statistical heterogeneity is between 0% and 40%, which might not be important (i.e., I2 = 0%).

e. Indirectness: We did not downgrade because the trials were conducted in different countries (high and low to lower-middle income).

f. Imprecision: We downgraded twice. The sample size was small (OIS would not have been reached).

g. Inconsistency: We did not downgrade; however, there are no other trials with which to compare findings.

h. Indirectness: We downgraded once. The trial was conducted in one country (high or upper-middle income).

i. Indirectness: We downgraded once. The trial was conducted in one country (low or lower-middle income).

#### References

1.Chhabra HS, Sharma S, Verma S. Smartphone app in self-management of chronic low back pain: a randomized controlled trial. 2018.

2.Jinnouchi H, Matsudaira K, Kitamura A, et al. Effects of brief self-exercise education on the management of chronic low back pain: A community-based, randomized, parallel-group pragmatic trial. 2020.

Summary of Findings table 6: ***What are the benefits and harms of Pilates exercise in the management of community-dwelling adults (including older adults aged 60 years and over) with chronic primary low back pain (with or without leg pain) compared with no treatment/no additional treatment?***

| Outcomes | **Anticipated absolute effects^*^** (95% CI) | | Relative effect (95% CI) | № of participants (studies) | Certainty of the evidence (GRADE) | Comments |
| --- | --- | --- | --- | --- | --- | --- |
|  | **Risk with no intervention** | **Risk with Pilates** |  |  |  |  |
| **ALL ADULTS** | | | | | | |
| **Pain**  0 to 10; 0 = no pain follow-up: closest to 2 weeks | The mean pain (unclassified presence of leg pain, high income country) was **5.2** | MD **2.1 lower** (3.07 lower to 1.13 lower) | - | 86 (1 RCT)^1,a^ | ⨁◯◯◯ Very low^b,c,d,e^ | Pilates reduces pain more than no treatment in the immediate term. |
| **Pain**  0 to 10; 0 = no pain follow-up: closest to 6 months | The mean pain (unclassified presence of leg pain, high income country) was **5.3** | MD **0.8 lower** (1.75 lower to 0.15 higher) | - | 86 (1 RCT)^1,a^ | ⨁◯◯◯ Very low^b,c,d,e^ | Pilates does not reduce pain more than no treatment in the intermediate term. |
| **Function** 0 to 24; 0 = no disability follow-up: closest to 2 weeks | The mean function (unclassified presence of leg pain, high income country) was **7.1** | MD **3.5 lower** (5.48 lower to 1.52 lower) | - | 86 (1 RCT)^1,a,f^ | ⨁◯◯◯ Very low^b,c,d,e^ | Pilates improves function more than no treatment in the immediate term. |
| **Function** 0 to 24; 0 = no disability follow-up: closest to 6 months | The mean function (unclassified presence of leg pain, high income country) was **6.7** | MD **2.2 lower** (4.35 lower to 0.05 lower) | - | 86 (1 RCT)^1,a,g^ | ⨁◯◯◯ Very low^b,c,d,e^ | Pilates improves function more than no treatment in the intermediate term (may not reach threshold for what may be considered clinically important). |
| **Fear Avoidance** 17 to 68; 0 = no fear avoidance follow-up: closest to 2 weeks | The mean fear Avoidance (unclassified presence of leg pain, high income country) was **38.1** | MD **1.8 lower** (5.12 lower to 1.52 higher) | - | 86 (1 RCT)^1,a^ | ⨁◯◯◯ Very low^b,c,d,e^ | Pilates does not reduce fear avoidance more than no treatment in the immediate term. |
| **Fear Avoidance** 17 to 68; 0 = no fear avoidance follow-up: closest to 6 months | The mean fear Avoidance (unclassified presence of leg pain, high income country) was **38.9** | MD **0.8 lower** (3.86 lower to 2.26 higher) | - | 86 (1 RCT)^1,a^ | ⨁◯◯◯ Very low^b,c,d,e^ | Pilates does not reduce fear avoidance more than no treatment in the intermediate term. |
| **Adverse events/harms** | Study authors reported that no adverse events occurred (86 participants total). | |  | (1 RCT)^1,a^ | ⨁◯◯◯ Very low^b,c,d,e^ |  |
| ***The risk in the intervention group** (and its 95% confidence interval) is based on the assumed risk in the comparison group and the **relative effect** of the intervention (and its 95% CI).  **CI:** confidence interval; **MD:** mean difference | | | | | | |
| **GRADE Working Group grades of evidence** **High certainty:** we are very confident that the true effect lies close to that of the estimate of the effect. **Moderate certainty:** we are moderately confident in the effect estimate: the true effect is likely to be close to the estimate of the effect, but there is a possibility that it is substantially different. **Low certainty:** our confidence in the effect estimate is limited: the true effect may be substantially different from the estimate of the effect. **Very low certainty:** we have very little confidence in the effect estimate: the true effect is likely to be substantially different from the estimate of effect. | | | | | | |

#### Explanations

a. Miyamoto 2013: participants received individual supervised exercise; 12 60-minute sessions.

b. Risk of bias: We downgraded once due to potential risk of bias (performance domain).

c. Inconsistency: We did not downgrade; however, there are no other trials with which to compare findings.

d. Indirectness: We downgraded once. The trial was conducted in one trial (high-income).

e. Imprecision: We downgraded twice. The sample size was small (OIS would not have been reached).

f. Miyamoto 2013: function was also measured using the Patient-Specific Functional Scale (PSFS, 0-10); MD = 1.10, 95% CI 0.23 to 1.97.

g. Miyamoto 2013: function was also measured using the Patient-Specific Functional Scale (PSFS, 0-10); MD = 0.80, 95% CI -0.00 to 1.60.

#### References

1.Miyamoto GC, Costa LO, Galvanin T, Cabral CM. Efficacy of the addition of modified Pilates exercises to a minimal intervention in patients with chronic low back pain: a randomized controlled trial. 2013.

Summary of Findings table 7: ***What are the benefits and harms of stretching, flexibility or mobilizing exercises in the management of community-dwelling adults (including older adults aged 60 years and over) with chronic primary low back pain (with or without leg pain) compared with no treatment/no additional treatment?***

| Outcomes | **Anticipated absolute effects^*^** (95% CI) | | Relative effect (95% CI) | № of participants (studies) | Certainty of the evidence (GRADE) | Comments |
| --- | --- | --- | --- | --- | --- | --- |
|  | **Risk with no intervention** | **Risk with stretching, flexibility or mobilizing exercises** |  |  |  |  |
| **ALL ADULTS** | | | | | | |
| **Pain**  0 to 10; 0 = no pain follow-up: closest to 2 weeks | The mean pain was **2.607** | MD **0.18 lower** (1.61 lower to 1.25 higher) | - | 30 (1 RCT)^1,a^ | ⨁◯◯◯ Very low^b,c,d,e^ | Stretching, flexibility or mobilizing exercises do not reduce pain more than no treatment in the immediate term. |
| **Function** 0 to 100; 0 = no disability follow-up: closest to 2 weeks | The mean function was **19.433** | MD **3.97 lower** (13.14 lower to 5.19 higher) | - | 30 (1 RCT)^1,a^ | ⨁◯◯◯ Very low^b,c,d,e^ | Stretching, flexibility or mobilizing exercises do not improve function more than no treatment in the immediate term. |
| ***The risk in the intervention group** (and its 95% confidence interval) is based on the assumed risk in the comparison group and the **relative effect** of the intervention (and its 95% CI).  **CI:** confidence interval; **MD:** mean difference | | | | | | |
| **GRADE Working Group grades of evidence** **High certainty:** we are very confident that the true effect lies close to that of the estimate of the effect. **Moderate certainty:** we are moderately confident in the effect estimate: the true effect is likely to be close to the estimate of the effect, but there is a possibility that it is substantially different. **Low certainty:** our confidence in the effect estimate is limited: the true effect may be substantially different from the estimate of the effect. **Very low certainty:** we have very little confidence in the effect estimate: the true effect is likely to be substantially different from the estimate of effect. | | | | | | |

#### Explanations

a. Shamsi 2022: participants had individual supervised exercise; 12 sessions (session duration not reported).

b. Risk of bias: We downgraded once due to potential risk of bias (performance and detection domains).

c. Inconsistency: We did not downgrade; however, there are no other trials with which to compare findings.

d. Indirectness: We downgraded once. The trial was conducted in one country (low or lower-middle income).

e. Imprecision: We downgraded twice. The sample size is small (OIS would not have been met).

#### References

1.Shamsi M, Ahmadi A, Mirzaei M, Jaberzadeh S. Effects of static stretching and strengthening exercises on flexion relaxation ratio in patients with LBP: A randomized clinical trial. 2022.

Summary of Findings table 8: ***What are the benefits and harms of yoga exercise in the management of community-dwelling adults (including older adults aged 60 years and over) with chronic primary low back pain (with or without leg pain) compared with usual care?***

| Outcomes | **Anticipated absolute effects^*^** (95% CI) | | Relative effect (95% CI) | № of participants (studies) | Certainty of the evidence (GRADE) | Comments |
| --- | --- | --- | --- | --- | --- | --- |
|  | **Risk with usual care** | **Risk with yoga** |  |  |  |  |
| **ALL ADULTS** | | | | | | |
| **Pain**  0 to 100; 0 = no pain follow-up: closest to 2 weeks | Yoga vs. usual care: difference in mean change -2.42, 95% CI -4.97 to 0.12 (313 participants total). | |  | (1 RCT)^1,a,b^ | ⨁◯◯◯ Very low^c,d,e,f^ | Yoga does not reduce pain more than usual care in the immediate term. |
| **Pain**  0 to 100; 0 = no pain follow-up: closest to 6 months | Yoga vs. usual care: difference in mean change -1.74, 95% CI -4.32 to 0.84 (313 participants total). | |  | (1 RCT)^1,a,b^ | ⨁◯◯◯ Very low^c,d,e,f^ | Yoga does not reduce pain more than usual care in the intermediate term. |
| **Pain**  0 to 100; 0 = no pain follow-up: closest to 12 months | Yoga vs. usual care: difference in mean change -0.73, 95% CI -3.30 to 1.84 (313 participants total). | |  | (1 RCT)^1,a,b^ | ⨁◯◯◯ Very low^c,d,e,f^ | Yoga does not reduce pain more t han usual care in the long term. |
| **Function** 0 to 24; 0 = no disability follow-up: closest to 2 weeks | Yoga vs. usual care: difference in mean change -2.17, 95% CI -3.31 to -1.03 (313 participants total). | |  | (1 RCT)^1,a,b^ | ⨁◯◯◯ Very low^c,d,e,f^ | Yoga improves function more than usual care in the immediate term (amount may not reach threshold for what may be considered clinically important). |
| **Function** 0 to 24; 0 = no disability follow-up: closest to 6 months | Yoga vs. usual care: difference in mean change -1.48, 95% CI -2.62 to -0.33 (313 participants total). | |  | (1 RCT)^1,a,b^ | ⨁◯◯◯ Very low^c,d,e,f^ | Yoga improves function more than usual care in the intermediate term (may not reach clinically important threshold). |
| **Function** 0 to 24; 0 = no disability follow-up: closest to 12 months | Yoga vs. usual care: difference in mean change -1.57, 95% CI -2.71 to -0.42 (313 participants total). | |  | (1 RCT)^1,a,b^ | ⨁◯◯◯ Very low^c,d,e,f^ | Yoga improves function more than usual care in the long term (may not reach clinically important threshold). |
| **Health-related quality of life** 0 to 100; 0 = poor QofL follow-up: closest to 2 weeks | Yoga vs. usual care: difference in mean change 1.36, 95% CI -0.70 to 3.41 (PCS); 2.02, 95% CI -0.31 to 4.35 (MCS) (313 participants total). | |  | (1 RCT)^1,a,b^ | ⨁◯◯◯ Very low^c,d,e,f^ | Yoga does not improve health-related quality of life more than usual care in the immediate term. |
| **Health-related quality of life** 0 to 100; 0 = poor QofL follow-up: closest to 6 months | Yoga vs. usual care: difference in mean change 1.24, 95% CI -0.83 to 3.33 (PCS); 2.02, 95% CI -0.34 to 4.37 (MCS) (313 participants total). | |  | (1 RCT)^1,a,b^ | ⨁◯◯◯ Very low^c,d,e,f^ | Yoga does not improve health-related quality of life more than usual care in the intermediate term. |
| **Health-related quality of life** 0 to 100; 0 = poor QofL follow-up: closest to 12 months | Yoga vs. usual care: difference in mean change 0.80, 95% CI -1.28 to 2.87 (PCS); 0.42, 95% CI -1.92 to 2.77 (MCS) (313 participants total). | |  | (1 RCT)^1,a,b^ | ⨁◯◯◯ Very low^c,d,e,f^ | Yoga does not improve health-related quality of life more than usual care in the long term. |
| **Self-efficacy** 0 to 60; 0 = poor self-efficacy follow-up: closest to 2 weeks | Yoga vs. usual care: difference in mean change 2.96, 95% CI 0.35 to 5.58 (313 participants total). | |  | (1 RCT)^1,a,b^ | ⨁◯◯◯ Very low^c,d,e,f^ | Yoga improves self-efficacy more than usual care in the immediate term (may not reach clinically important threshold). |
| **Self-efficacy** 0 to 60; 0 = poor self-efficacy follow-up: closest to 6 months | Yoga vs. usual care: difference in mean change 3.33, 95% CI 0.68 to 5.97 (313 participants total). | |  | (1 RCT)^1,a,b^ | ⨁◯◯◯ Very low^c,d,e,f^ | Yoga improves self-efficacy more than usual care in the intermediate term (may not reach clinically important threshold). |
| **Self-efficacy** 0 to 60; 0 = poor self-efficacy follow-up: closest to 12 months | Yoga vs. usual care: difference in mean change 1.75, 95% CI -0.87 to 4.38 (313 participants total). | |  | (1 RCT)^1,a,b^ | ⨁◯◯◯ Very low^c,d,e,f^ | Yoga does not improve self-efficacy more than usual care in the long term. |
| **Minor adverse events/harms** | 0 per 1,000 | **81 per 1,000** (0 to 0) | **OR 25.77** (1.50 to 441.85) | 274 (1 RCT)^1,a,g^ | ⨁◯◯◯ Very low^c,d,e,h^ | Yoga increases minor adverse events/harms more than usual care. |
| **Serious adverse events/harms** | 14 per 1,000 | **7 per 1,000** (1 to 77) | **OR 0.51** (0.05 to 5.70) | 274 (1 RCT)^1,a,i^ | ⨁◯◯◯ Very low^c,d,e,h^ | Yoga does not increase serious adverse events/harms more than usual care. |
| ***The risk in the intervention group** (and its 95% confidence interval) is based on the assumed risk in the comparison group and the **relative effect** of the intervention (and its 95% CI).  **CI:** confidence interval; **OR:** odds ratio | | | | | | |
| **GRADE Working Group grades of evidence** **High certainty:** we are very confident that the true effect lies close to that of the estimate of the effect. **Moderate certainty:** we are moderately confident in the effect estimate: the true effect is likely to be close to the estimate of the effect, but there is a possibility that it is substantially different. **Low certainty:** our confidence in the effect estimate is limited: the true effect may be substantially different from the estimate of the effect. **Very low certainty:** we have very little confidence in the effect estimate: the true effect is likely to be substantially different from the estimate of effect. | | | | | | |

#### Explanations

a. Tilbrook 2011: Participants had group supervised exercise; 12 75-minute sessions.

b. Tilbrook 2011: only reported within-group changes; follow-up scores were not provided.

c. Risk of bias: We downgraded once due to potential risk of bias (performance and detection domains).

d. Inconsistency: We did not downgrade; however, there are no other trials with which to compare findings.

e. Indirectness: We downgraded once because the trial was conducted in one country (high-income).

f. Imprecision: We downgraded once. The sample size was small (OIS would not have been achieved).

g. Minor adverse events: included increased pain.

h. Imprecision: We downgraded twice due to small sample size and number of events.

i. Major adverse event: 1 participant experienced severe pain (possibly associated with yoga). In usual care group, 1 participant died; 1 had severe accident/injury.

#### References

1.Tilbrook HE, Cox H, Hewitt CE, et al. Yoga for chronic low back pain: a randomized trial. 2011.

Summary of Findings table 9: ***What are the benefits and harms of motor control exercise in the management of community-dwelling adults (including older adults aged 60 years and over) with chronic primary low back pain (with or without leg pain) compared with sham?***

| Outcomes | **Anticipated absolute effects^*^** (95% CI) | | Relative effect (95% CI) | № of participants (studies) | Certainty of the evidence (GRADE) | Comments |
| --- | --- | --- | --- | --- | --- | --- |
|  | **Risk with sham** | **Risk with motor control exercise** |  |  |  |  |
| **ALL ADULTS** | | | | | | |
| **Pain**  0 to 10; 0 = no pain follow-up: closest to 2 weeks | The mean pain was **5.6** | MD **1 lower** (1.85 lower to 0.15 lower) | - | 154 (1 RCT)^1,a^ | ⨁◯◯◯ Very low^b,c,d,e^ | Motor control exercise reduces pain more than sham in the immediate term. |
| **Pain**  0 to 10; 0 = no pain follow-up: closest to 6 months | The mean pain was **5.6** | MD **0.6 lower** (1.46 lower to 0.26 higher) | - | 154 (1 RCT)^1,a^ | ⨁◯◯◯ Very low^b,c,d,e^ | Motor control exercise does not reduce pain more than sham in the intermediate term. |
| **Pain**  0 to 10; 0 = no pain follow-up: closest to 12 months | The mean pain was **6.3** | MD **1.3 lower** (2.13 lower to 0.47 lower) | - | 154 (1 RCT)^1,a^ | ⨁◯◯◯ Very low^b,c,d,e^ | Motor control exercise reduces pain more than sham in the long term. |
| **Function** 0 to 24; 0 = no disability follow-up: closest to 2 weeks | The mean function was **11.9** | MD **2.3 lower** (4.26 lower to 0.34 lower) | - | 154 (1 RCT)^1,a,f^ | ⨁◯◯◯ Very low^b,c,d,e^ | Motor control exercise improves function more than sham in the immediate term (amount may not reach threshold for what may be considered clinically important). |
| **Function** 0 to 24; 0 = no disability follow-up: closest to 6 months | The mean function was **12.2** | MD **1.9 lower** (4.06 lower to 0.26 higher) | - | 154 (1 RCT)^1,a,g^ | ⨁◯◯◯ Very low^b,c,d,e^ | Motor control exercise does not improve function more than sham in the intermediate term. |
| **Function** 0 to 24; 0 = no disability follow-up: closest to 12 months | The mean function was **12.3** | MD **0.9 lower** (3.15 lower to 1.35 higher) | - | 154 (1 RCT)^1,a,h^ | ⨁◯◯◯ Very low^b,c,d,e^ | Motor control exercise does not improve function more than sham in the long term. |
| **Adverse events/harms** | 26 per 1,000 | **39 per 1,000** (7 to 200) | **OR 1.52** (0.25 to 9.36) | 154 (1 RCT)^1,a,i^ | ⨁◯◯◯ Very low^b,c,d,j^ | Motor control exercise does not increase adverse events/harms more than sham. |
| ***The risk in the intervention group** (and its 95% confidence interval) is based on the assumed risk in the comparison group and the **relative effect** of the intervention (and its 95% CI).  **CI:** confidence interval; **MD:** mean difference; **OR:** odds ratio | | | | | | |
| **GRADE Working Group grades of evidence** **High certainty:** we are very confident that the true effect lies close to that of the estimate of the effect. **Moderate certainty:** we are moderately confident in the effect estimate: the true effect is likely to be close to the estimate of the effect, but there is a possibility that it is substantially different. **Low certainty:** our confidence in the effect estimate is limited: the true effect may be substantially different from the estimate of the effect. **Very low certainty:** we have very little confidence in the effect estimate: the true effect is likely to be substantially different from the estimate of effect. | | | | | | |

#### Explanations

a. Costa 2009: participants had individual supervised exercise; 12 30-minute sessions.

b. Risk of bias: We did not downgrade. The study was rated as overall low risk of bias.

c. Inconsistency: We did not downgrade; however, there are no other trials with which to compare findings.

d. Indirectness: We downgraded once because the trial was conducted in one country (high or upper-middle income).

e. Imprecision: We downgraded twice due to small sample size (OIS would not have been achieved).

f. Costa 2009: function was also measured using the Patient-Specific Functional Scale (PSFS, 0-10); MD = 1.10, 95% CI 0.36 to 1.84.

g. Costa 2009: function was also measured using the Patient-Specific Functional Scale (PSFS, 0-10); MD = 1.00, 95% CI 0.16 to 1.84.

h. Costa 2009: function was also measured using the Patient-Specific Functional Scale (PSFS, 0-10); MD = 1.50, 95% CI 0.68 to 2.32.

i. Adverse events: temporary exacerbations of pain.

j. Imprecision: We downgraded twice due to small sample size and number of events.

#### References

1.Costa LO, Maher CG, Latimer J ,et al. Motor control exercise for chronic low back pain: a randomized placebo-controlled trial. 2009.

Summary of Findings table 10: ***What are the benefits and harms of exercise in the management of community-dwelling adults (including older adults aged 60 years and over) with chronic primary low back pain (with or without leg pain) compared with no treatment/no additional treatment?***

| Outcomes | **Anticipated absolute effects^*^** (95% CI) | | Relative effect (95% CI) | № of participants (studies) | Certainty of the evidence (GRADE) | Comments |
| --- | --- | --- | --- | --- | --- | --- |
|  | **Risk with no treatment** | **Risk with exercise** |  |  |  |  |
| **Pain**  follow-up: closest to 2 weeks | The mean pain was **2.9 (VAS, 0-10)** to **36.83 (VAS, 0-100)** | SMD **0.33 lower** (0.58 lower to 0.08 lower) | - | 619 (8 RCTs)^1,2,3,4,5,6,7,8,a,b,c^ | ⨁⨁⨁◯ Moderate^d,e,f,g^ | Exercise reduces pain more than no treatment in the immediate term. |
| **Pain**  0 to 10; 0 = no pain follow-up: closest to 3 months | The mean pain (unclassified presence of leg pain, high or upper-middle income country) was **4.092 (VAS, 0-10)** to **4.74 (VAS, 0-10)** | MD **0.68 lower** (1.82 lower to 0.46 higher) | - | 97 (2 RCTs)^5,7^ | ⨁◯◯◯ Very low^d,i,p,t^ | Exercise does not reduce pain more than no treatment in the short term. |
| **Pain**  0 to 10; 0 = no pain follow-up: closest to 6 months | The mean pain (high or upper-middle income country) was **3.9 (VAS, 0-10)** to **41.32 (VAS, 0-100)** | SMD **0.08 lower** (0.29 lower to 0.13 higher) | - | 404 (4 RCTs)^1,2,7,8,b,u^ | ⨁⨁◯◯ Low^d,p,q,v^ | Exercise does not reduce pain more than no treatment in the intermediate term. |
| **Pain**  0 to 10; 0 = no pain follow-up: closest to 12 months | Between-group MD graded activity with problem solving training alone vs. combination treatment = 8.88, 95% CI -0.36 to 18.13 (119 participants total). | |  | (1 RCT)^9,y^ | ⨁◯◯◯ Very low^i,l,m,z^ | Exercise does not reduce pain more than no treatment in the long term. |
| **Function**  follow-up: closest to 2 weeks | The mean function was **4.3 (RMDQ, 0-24)** to **75.1 (Hannover, 0-100)** | SMD **0.31 lower** (0.57 lower to 0.05 lower) | - | 619 (8 RCTs)^1,2,3,4,5,6,7,8,a,aa,b^ | ⨁⨁⨁◯ Moderate^ab,d,f,g^ | Exercise improves function more than no treatment in the immediate term. |
| **Function**  follow-up: closest to 3 months | The mean function (high or upper-middle income country, unclassified presence of leg pain) **was 5.58 (WI, 0-9)** to **74.9 (Hannover, 0-100)** | SMD **0.26 lower** (0.67 lower to 0.14 higher) | - | 97 (2 RCTs)^5,7^ | ⨁◯◯◯ Very low^d,h,i,p^ | Exercise does not improve function more than no treatment in the short term. |
| **Function**  follow-up: closest to 6 months | The mean function (high or upper-middle income country) was **5.7 (RMDQ, 0-24)** to **8.4 (RMDQ, 0-24)** | SMD **0.16 lower** (0.39 lower to 0.07 higher) | - | 404 (4 RCTs)^1,2,7,8,af,b^ | ⨁⨁◯◯ Low^ag,d,p,v^ | Exercise does not improve function more than no treatment in the intermediate term. |
| **Function** 0 to 24; 0 = no disability follow-up: closest to 12 months | Between-group MD (RMDQ 0-24) graded activity with problem solving training alone vs. combination treatment = 1.62, 95% CI -0.06 to 3.31 (119 participants total). | |  | (1 RCT)^9,c^ | ⨁◯◯◯ Very low^i,l,m,z^ | Exercise does not improve function more than no treatment in the long term. |
| **Health-related quality of life**  (physical component) 0 to 100; 0 = poor QofL follow-up: closest to 2 weeks | The mean health-related quality of life (high or upper-middle income country) was **39.9 (SF-36 PCS, 0-100)** to **62.2 (SF-36 PCS, 0-100)** | MD **2.31 lower** (10.36 lower to 5.75 higher) | - | 248 (2 RCTs)^5,8,b^ | ⨁◯◯◯ Very low^ah,d,k,p^ | Exercise does not improve health-related quality of life (physical) more than no treatment in the immediate term. |
| **Health-related quality of life**  mental component) 0 to 100; 0 = poor QofL follow-up: closest to 2 weeks | The mean health-related quality of life (high or upper-middle income country) was **0** | MD **1.11 lower** (3.67 lower to 1.45 higher) | - | 248 (2 RCTs)^5,8,b^ | ⨁⨁◯◯ Low^d,h,k,p^ | Exercise does not improve health-related quality of life (mental) more than no treatment in the immediate term. |
| **Adverse events** | 0 per 1,000 | **20 to 55 per 1,000** (0.12 to 76.95 – 0.37 to 146.73) | **OR 4.24** (0.69 to 25.95) | 310 (2 RCTs)^8,9,ai,b^ | ⨁◯◯◯ Very low^aj,d,h,p^ | Exercise does not lead to adverse events more than no treatment. |
| ***The risk in the intervention group** (and its 95% confidence interval) is based on the assumed risk in the comparison group and the **relative effect** of the intervention (and its 95% CI).  **CI:** confidence interval; **MD:** mean difference; **OR:** odds ratio; **SMD:** standardised mean difference | | | | | | |
| **GRADE Working Group grades of evidence** **High certainty:** we are very confident that the true effect lies close to that of the estimate of the effect. **Moderate certainty:** we are moderately confident in the effect estimate: the true effect is likely to be close to the estimate of the effect, but there is a possibility that it is substantially different. **Low certainty:** our confidence in the effect estimate is limited: the true effect may be substantially different from the estimate of the effect. **Very low certainty:** we have very little confidence in the effect estimate: the true effect is likely to be substantially different from the estimate of effect. | | | | | | |

#### Explanations

a. Shamsi 2022: had 2 comparisons (both included in meta-analysis).

b. Weiner 2008: had 2 comparisons (both included in meta-analysis).

c. Smeets 2008 was not included in the meta-analysis (provided within-group mean changes; no follow-up scores). 119 participants, rated as overall low risk of bias. Participants performed combination treatment (active physical treatment [aerobic and core strengthening exercises] + graded activity with problem solving training) vs. graded activity with problem solving training alone. Between-group MD (VAS 0-100) graded activity with problem solving training alone vs. combination treatment = 5.35, 95% CI -3.73 to 14.42.

d. Risk of bias: We downgraded once. Most or all trial(s) rated as overall unclear risk of bias.

e. Inconsistency: We did not downgrade. Most of the point estimates are in the same direction and confidence intervals overlap. Statistical heterogeneity is between 30% and 60% (i.e., I2 = 55%). This could not be explained due to small subgroups and may represent moderate heterogeneity.

f. Indirectness: We did not downgrade. The trials were conducted in different countries (high- and low-income).

g. Imprecision: We did not downgrade. The point estimate reached the pre-specified threshold for what may be considered clinically important (MD ≥ 1 or SMD ≥ 0.2 ). The confidence interval does not cross the null.

h. Inconsistency: We did not downgrade. Most or all the point estimates are in the same direction and confidence intervals overlap. Statistical heterogeneity is between 0% and 40%, which might not be important (i.e., I2 = 0%).

i. Imprecision: We downgraded twice. The sample size was small (OIS would not have been achieved).

j. Inconsistency: We downgraded once. Most of the point estimates are in the same direction and confidence intervals overlap. Statistical heterogeneity is between 50% and 90% (i.e., I2 = 67%). This could not be explained due to small subgroups and may represent substantial heterogeneity.

k. Imprecision: We downgraded once. The sample size was small (OIS would not have been achieved).

l. Inconsistency: We did not downgrade; however, there are no other trials with which to compare findings.

m. Indirectness: We downgraded once. The trial was conducted in one country (high or upper-middle income).

n. Imprecision: We downgraded twice. The point estimate did not reach the pre-specified threshold for what may be considered clinically important (MD ≥ 1 or SMD ≥ 0.2 ). The boundaries of the confidence interval cross the thresholds for what may be considered appreciable benefit and harm (MD ≥ 1 or SMD ≥ 0.2 ).

o. Inconsistency: We downgraded once. Most of the point estimates are in the same direction and confidence intervals overlap. Statistical heterogeneity is between 50% and 90% (i.e., I2 = 65%). This could not be explained due to small subgroups and may represent substantial heterogeneity.

p. Indirectness: We did not downgrade. The trials were conducted in different countries (high or upper-middle income).

q. Inconsistency: We did not downgrade. Most or all the point estimates are in the same direction and confidence intervals overlap. Statistical heterogeneity is between 0% and 40%, which might not be important (i.e., I2 = 11%).

r. Indirectness: We downgraded once; trial(s) conducted in one country (low or lower-middle income).

s. Inconsistency: We did not downgrade. Most of the point estimates are in the same direction and confidence intervals overlap. Statistical heterogeneity is between 0% and 40%, which might not be important (i.e., I2 = 34%).

t. Inconsistency: We did not downgrade. Most or all of the point estimates are in the same direction and confidence intervals overlap. Statistical heterogeneity is between 0% and 40%, which might not be important (i.e., I2 = 40%).

u. Smeets 2008 was not included in the meta-analysis (provided within-group mean changes; no follow-up scores). 119 participants, rated as overall low risk of bias. Participants performed combination treatment (active physical treatment [aerobic and core strengthening exercises] + graded activity with problem solving training) vs. graded activity with problem solving training alone. Between-group MD (VAS 0-100) graded activity with problem solving training alone vs. combination treatment = 6.25, 95% CI -2.94 to 15.44.

v. Imprecision: We downgraded once. The point estimate did not reach the pre-specified threshold for what may be considered clinically important (MD ≥ 1 or SMD ≥ 0.2). One of the boundaries of the confidence interval crosses this threshold.

w. Inconsistency: We downgraded once. The point estimates are in different directions with some overlap of confidence intervals. Statistical heterogeneity is between 30% and 60% (i.e., I2 = 44%). This could not be explained due to small subgroups and may represent moderate heterogeneity.

x. Inconsistency: We did not downgrade. Most or all of the point estimates are in the same direction and confidence intervals overlap. Statistical heterogeneity is between 0% and 40%, which might not be important (i.e., I2 = 6%).

y. Smeets 2008 provided within-group mean changes, no follow-up scores.

z. Risk of bias: We did not downgrade because the study was rated as overall low risk of bias.

aa. Smeets 2008 was not included in the meta-analysis (provided within-group mean changes; no follow-up scores). 119 participants, rated as overall low risk of bias. Participants performed combination treatment (active physical treatment [aerobic and core strengthening exercises] + graded activity with problem solving training) vs. graded activity with problem solving training alone. Between-group MD (RMDQ 0-24) graded activity with problem solving training alone vs. combination treatment = 0.58, 95% CI -1.08 to 2.24.

ab. Inconsistency: We did not downgrade. Most of the point estimates are in the same direction with some overlap of confidence intervals. Statistical heterogeneity is between 30% and 60% (i.e., I2 = 58%). This could not be explained due to small subgroups and may represent moderate heterogeneity.

ac. Inconsistency: We did not downgrade. Most of the point estimates are in the same direction and confidence intervals overlap. Statistical heterogeneity is between 30% and 60% (i.e., I2 = 44%). This could not be explained due to small subgroups and may represent moderate heterogeneity.

ad. Imprecision: We downgraded once. The point estimate reached the pre-specified threshold for what may be considered clinically important (MD ≥ 1 or SMD ≥ 0.2). One of the boundaries of the confidence interval crosses this threshold.

ae. Inconsistency: We downgraded once. Most of the point estimates are in the same direction and confidence intervals overlap. Statistical heterogeneity is between 30% and 60% (i.e., I2 = 40%). This could not be explained due to small subgroups and may represent moderate heterogeneity.

af. Smeets 2008 was not included in the meta-analysis (provided within-group mean changes; no follow-up scores). 119 participants, rated as overall low risk of bias. Participants performed combination treatment (active physical treatment [aerobic and core strengthening exercises] + graded activity with problem solving training) vs. graded activity with problem solving training alone. Between-group MD (RMDQ 0-24) graded activity with problem solving training alone vs. combination treatment = 1.11, 95% CI -0.56 to 2.79.

ag. Inconsistency: We did not downgrade. Most of the point estimates are in the same direction and confidence intervals overlap. Statistical heterogeneity is between 0% and 40%, which might not be important (i.e., I2 = 26%).

ah. Inconsistency: We downgraded once. Most of the point estimates are in the same direction and confidence intervals overlap. Statistical heterogeneity is between 50% and 90% (i.e., I2 = 74%). This could not be explained due to small subgroups and may represent substantial heterogeneity.

ai. Adverse events: were mainly minor and included back and knee pain.

aj. Imprecision: We downgraded twice due to small sample size and number of events.

#### References

1.Fukuda TY, Aquino LM, Pereira P, et al. Does adding hip strengthening exercises to manual therapy and segmental stabilization improve outcomes in patients with nonspecific low back pain? A randomized controlled trial. 2021.

2.Miyamoto GC, Costa LO, Galvanin T, Cabral CM. Efficacy of the addition of modified Pilates exercises to a minimal intervention in patients with chronic low back pain: a randomized controlled trial. 2013.

3.Nardin DMK, Stocco MR, Aguiar AF, Machado FA, de Oliveira RG, Andraus RAC. Effects of photobiomodulation and deep water running in patients with chronic non-specific low back pain: a randomized controlled trial. 2022.

4.Rahbar M, Salekzamani Y, Jahanjou F, Eslamian F, Niroumand A, Dolatkhah N. Effect of hippotherapy simulator on pain, disability, and range of motion of the spinal column in subjects with mechanical low back pain: A randomized single-blind clinical trial. 2018.

5.Rotter G, Ortiz M, Binting S, et al. Mindful Walking in Patients with Chronic Low Back Pain: A Randomized Controlled Trial. 2022.

6.Shamsi M, Ahmadi A, Mirzaei M,J aberzadeh S. Effects of static stretching and strengthening exercises on flexion relaxation ratio in patients with LBP: A randomized clinical trial. 2022.

7.Z, Takinaci. Clinical efficacy of spa therapy (balneotherapy) for chronic low back pain: A randomized single-blind trial. 2019.

8.Weiner DK, Perera S, Rudy TE, Glick RM,S henoy S, Delitto A. Efficacy of percutaneous electrical nerve stimulation and therapeutic exercise for older adults with chronic low back pain: a randomized controlled trial. 2008.

9.Smeets R, Vlaeyen JWS, Hidding A, Kester ADM, van der Heijden G, Knottnerus AJ. Chronic low back pain: physical training, graded activity with problem solving training, or both? The one-year post-treatment results of a randomized controlled trial. 2008.

**Online Resource 7.** Meta-analyses

**References**

**Online Resource 8.** Supplementary evidence synthesis (Table of included RCTs, GRADE Evidence Profile tables, meta-analyses)

Characteristics of RCTs included in the supplementary evidence synthesis (n=68)

| **First author, year**  **(ref ID)** | **Methods** | **Participants** | **Interventions/Comparisons** | **Outcomes** | **Follow-up (post-intervention)** |
| --- | --- | --- | --- | --- | --- |
| Abadi 2019 (46204) | Country: Malaysia  ROB: high | Number of participants: n=39 (E: 19; C: 20)  % female: 100  Mean age, years (SD): E: 37.6 (5.8), C: 40.8 (5.3) | Intervention: aerobic (in water)  Comparison: no treatment | Pain: ODI (first question) (0-5)  Function: ODI (0-100) | immediate term (closest to 2 weeks) |
| Abdel-Azeim 2021 (53158) | Country: Egypt  ROB: high | Number of participants: n=48 (E: 21; C: 22)  % female: 100  Mean age, years: E: 36.7 (6.2), C: 38.1 (5.9) | Intervention: motor control exercise (core strengthening, motor control exercise (pelvic floor exercise, stabilization exercise, physical therapy agents) + TENS)  Comparison: no treatment (motor control exercise; stabilization exercise, physical therapy agents) + TENS | Pain: NPRS (0-10)  Function: ODI (0-100) | immediate term (closest to 2 weeks) |
| Afzal 2022 (53165) | Country: Pakistan  ROB: high | Number of participants: n=84 (E: 42; C: 42)  % female: E: 64.3; C: 69  Mean age, years: E: 37.5 (12.5); C: 38.2 (11.8) | Intervention: general strength training (aerobic, core strengthening, stretching/flexibility/mobilizing exercises + heat)  Comparison: no treatment (core strengthening, stretching/flexibility/mobilizing exercises) + heat | Pain: VAS (0-10)  Function: ODI (0-100) | immediate term (closest to 2 weeks) |
| Ahmadizadeh 2019 (46220) | Country: Iran  ROB (high/low): high | Number of participants: n=32 (E: 16; C: 16)  % female: 100  Mean age, years: E: 31.1 (8.3); C: 34.2 (8.4) | Intervention: core strengthening, stretching/flexibility/mobilizing exercises + education with self-management  Comparison: no treatment (education with self-management) | Pain: VAS (0-10)  Function: RMDQ (0-18) | immediate term (closest to 2 weeks) |
| Ain 2019 (46853) | Country: Pakistan  ROB: high | Number of participants: n=40 (E: 20; C: 20)  % female: 100  Mean age, years: E: 41.3 (10.5); C: 35.1 (9.0) | Intervention: stretching/flexibility/mobilizing exercises + electrotherapy, heat, mobilization/manipulation  Comparison: no treatment  (electrotherapy, heat, mobilization/manipulation) | Pain: NPRS (0-10)  Function: ODI (0-50) | immediate term (closest to 2 weeks) |
| Alikhajeh 2020 (46957) | Country: Iran  ROB: high | Number of participants: n=24 (E: 12; C: 12)  % female: 100  Mean age, years: E: 51.6 (3.8); C: 50.7 (3.3) | Intervention: aerobic (in water)  Comparison: no treatment/wait list | Pain: VAS (0-100)  Function: ODI (0-50) | immediate term (closest to 2 weeks) |
| Alvani 2021 (1776) | Country: Iran  ROB: high | Number of participants: n=30 (E: 15; C: 15)  % female: 0  Mean age, years: E: 40.6 (6.0); C: 30.7 (7.8) | Intervention: core strengthening, stretching/flexibility/mobilizing exercises + daily routine physical and exercise activities  Comparison: no treatment (daily routine physical and exercise activities) | Pain: VAS (0-10)  Function: ODI (0-100) | immediate term (closest to 2 weeks) |
| Ansari 2021 (46984) | Country: Iran  ROB: high | Number of participants: n=20 (E: 10; C: 10)  % female: 100  Mean age, years: E: 58.8 (3.73); C: 57.0 (4.1) | Intervention: aerobic (in water)  Comparison: no treatment/wait list | Function: Quebec Back Pain Disability Scale (0-100) | immediate term (closest to 2 weeks) |
| Barni 2018 (43368) | Country: Italy  ROB: high | Number of participants: n=22 (E: 11; C: 11)  % female: NR  Mean age, years: E: 61.4 (5.3); C: 60.9 (7.3) | Intervention: aerobic (aerobic, core strengthening, general strength training, stretching/flexibility/mobilizing exercises + education)  Comparison: no treatment (core strengthening, general strength training, stretching/flexibility/mobilizing exercises + education) | Pain: NPRS (0-10)  Function: RMDQ (0-24) | immediate term (closest to 2 weeks) |
| Bruehl 2020 (47043) | Country: United States  ROB: high | Number of participants: n= 82 (E: 38; C: 44)  % female: E: 55.3; C: 65.9  Mean age, years: E: 40.0 (10.0); C: 41.9 (9.5) | Intervention: aerobic  Comparison: no treatment | Pain: NPRS (0-10)  Function: PROMIS Short Form (Standardized T scores) | immediate term (closest to 2 weeks) |
| Chhabra 2018 (43412) | Country: India  ROB: unclear | Number of participants: n=93 (E: 45, C: 48)  % female: NR  Mean age, years: E: 41.4 (14.2), C: 41.0 (14.2) | Intervention: mixed (aerobic, core strengthening)  Comparison: usual care (conventional therapy; written prescription from the physician, containing a list of prescribed medicines and dosages, and stating the recommended level of physical activity (including home exercises) | Pain: NPRS (0-10)  Function: ODI (0-100) | immediate term (closest to 2 weeks) |
| Costa 2009 (9861) | Country: Australia  ROB: low | Number of participants: n=154 (E: 77; C: 77)  % female: E:58; C: 62  Mean age, years: E: 54.6 (13.0), C: 52.8 (12.7) | Intervention: motor control exercise  Comparison: placebo  (detuned shortwave diathermy and detuned ultrasound) | Pain: NPRS (0-10)  Function: RMDQ (0-24)  Adverse events: participants asked at immediate follow-up | immediate term (closest to 2 weeks)  long term (closest to 12 months) |
| da Luz 2019 (46326) | Country: Brazil  ROB: high | Number of participants: n= 20 (E: 10; C: 10)  % female: 100  Mean age, years (SD): E: 25.5 (5.3); C: 27.1 (5.0) | Intervention: core strengthening + neuromuscular electrical stimulation  Comparison: no treatment (neuromuscular electrical stimulation alone) | Pain: VAS (0-10)  Function: RMDQ (0-24) | immediate term (closest to 2 weeks) |
| da Silva 2014 (46060) | Country: Brazil  ROB: high | Number of participants: n= 18 (E: 9; C: 9)  % female: E: 100; C: 89  Mean age, years: E: 70.1 (2.7); 64.6 (8.5) | Intervention: mixed (core strengthening, general strength training, stretching or flexibility/mobilizing exercise + pamphlets)  Comparison: no treatment (pamphlets alone) | Pain: VAS (0-10)  Function: RMDQ (0-24) | immediate term (closest to 2 weeks) |
| Dalichau 2003 (1621) | Country: Germany  ROB: high | Number of participants: n= 90 (E1: 18; E2: 19; E3: 20; E4: 17; C: 16)  % female: 0  Mean age, years (SD): E1: 37.3 (3.1); E2: 38.1 (3.4); E3: 37.4 (3.4); E4: 38.2 (4.2); C: 39.8 (4.5) | Intervention 1: mixed (aerobic, general strength training; stretching or flexibility/mobilizing exercises)  Intervention 2: aerobic, core strengthening, motor control exercises, stretching or flexibility/mobilizing exercise  Intervention: aerobic, general strength training; stretching or flexibility/mobilizing  Intervention: aerobic, general strength training; stretching or flexibility/mobilizing  Comparison: no treatment | Pain: VAS (0-10)  Function: ODI (0-100) | immediate term (closest to 2 weeks) |
| Dalichau 2005 (1865) | Country: Germany  ROB: high | Number of participants: n= 102 (E1: 25; E2: 27; E3: 25; C: 25)  % female: 0  Mean age, years: NR | Intervention 1: mixed (general strength training, stretching or flexibility/mobilizing exercises, Pilates)  Intervention 2: mixed (general strength training, stretching or flexibility/mobilizing exercises)  Intervention 3: mixed (general strength training, stretching or flexibility/mobilizing exercises)  Comparison: no treatment | Pain: VAS (0-10)  Function: ODI (0-100) | immediate term (closest to 2 weeks) |
| Dineshkumar 2015 (46063) | Country: India  ROB: high | Number of participants: n= 60 (E: 30; C: 30)  % female: E: 50; C: 47  Mean age, years (SD): E: 28.7 (6.9); C: 28.7 (5.5) | Intervention: motor control exercise; visual feedback with resisted dorsiflexion  Comparison: no treatment (visual feedback alone) | Pain: NPRS (0-10)  Function: RMDQ (0-24) | immediate term (closest to 2 weeks) |
| Fouda 2021 (1625) | Country: Egypt  ROB: high | Number of participants: n= 60 (E: 20 ; C1: 20; C2: 20)  % female: E: 45, C1: 44, C2: 50  Mean age, years: E: 38.75 (7.45), C1: 39.11 (9.60), C2: 38.09 (8.80) | Intervention: core strengthening, isotonic exercises  Comparison 1: no treatment (core strengthening)  Comparison 2: no treatment (isotonic exercises) | Function: ODI (0-100) | immediate term (closest to 2 weeks) |
| Fukuda 2021 (53055) | Country: Brazil  ROB: unclear | Number of participants: n=70 (E: 35; C: 35)  % female: 53%  Mean age, years: E: 40.2 (12.4), C: 35.2 (12.5) | Intervention: general strength training, lumbar stabilization + manual therapy  Comparison: no treatment (lumbar stabilization + manual therapy) | Pain: VAS (0-10)  Function: RMDQ (0-24) | immediate term (closest to 2 weeks)  long term (closest to 12 months) |
| Gao 2018 (45037) | Country: China  ROB: high | Number of participants: n= 60 (E: 30; C: 30)  % female: E: 43.3, C: 30  Mean age, years: E: 35.68 (12.56), C: 36.45 (12.38) | Intervention: Qigong  Comparison: no treatment | Pain: VAS (0-10)  Function: ODI (0-45) | immediate term (closest to 2 weeks) |
| Ge 2022 (53137) | Country: China  ROB: high | Number of participants: n= 31 (E: 15; C: 16)  % female: 100  Mean age, years: E: 64.60 (3.71), C: 64.12 (2.96) | Intervention: core strengthening  Comparison: no treatment | Pain: VAS (0-10)  Function: ODI (0-100) | immediate term (closest to 2 weeks) |
| Gupta 2019 (44157) | Country: India  ROB: high | Number of participants: n= 30 (E:15 ; C: 15)  % female: 40  Mean age, years: E: 48.06 (4.97), C: 49.2 (5.528) | Intervention: aerobic  Comparison: usual care (conventional treatment: mobilization, stretching and exercises) | Function: ODI (0-100) | immediate term (closest to 2 weeks) |
| Hatefi 2021 (1879) | Country: Iran  ROB: high | Number of participants: n= 30 (E: 15; C: 15)  % female: 100  Mean age, years: E: 26.27 (2.13), C: 26.43 (2.57) | Intervention: stretching, or flexibility/mobilizing exercises  Comparison: no treatment | Pain: VAS (0-10)  Function: ODI (0-50) | immediate term (closest to 2 weeks) |
| Heidari 2018 (43527) | Country: Iran  ROB: high | Number of participants: n= 32 (E1: 11; E2: 11; C: 10)  % female: 100  Mean age, years: E1: 36.3 (7.2), E2: 35.9 (8.1), C: 32.9 (7.6) | Intervention: core strengthening, stretching/flexibility/mobilizing exercises)  Comparison: no treatment | Function: Quebec pain disability scale (0-100) | immediate term (closest to 2 weeks) |
| Hemmati 2011 (45008) | Country: Iran  ROB: high | Number of participants: n= 24 (E: 12; C: 12)  % female: 100  Mean age, years: E: 21.3, C: 22.72 | Intervention: core strengthening  Comparison: no treatment | Pain: VAS (0-10)  Function: ODI (0-50) | immediate term (closest to 2 weeks) |
| Idowu 2020 (1650) | Country: Nigeria  ROB: high | Number of participants: n= 51(E: 25; C: 26)  % female: E: 68, C: 62  Mean age, years: E: 48.28 (9.41), C: 48.27 (9.56) | Intervention: aerobic (aerobic, general strength training (graded activity + walking))  Comparison: no treatment (general strength training; graded activity) | Pain: VAS (0-10) | immediate term (closest to 2 weeks) |
| Jinnouchi 2020 (47336) | Country: Japan  ROB: unclear | Number of participants: n=52 (E: 26; C: 26)  % female: E: 65.4; C: 61.5  Mean age, years: E: 65 (62-70), C: 66 (64-71) | Intervention: mixed (aerobic, core strengthening, stretching, or flexibility/mobilizing exercises + education)  Comparison: no treatment (education only) | Pain: NPRS (0-10)  Function: RMDQ (0-24) | immediate term (closest to 2 weeks)  short term (closest to 3 months) |
| Joseph 2018 (43560) | Country: Thailand  ROB: high | Number of participants: n= 16 (E:16; C:16 )  % female: 100  Mean age, years: 20.4 | Intervention: core strengthening (core strengthening (using Pilates reformer device) + massage therapy)  Comparison: no treatment (massage therapy) | Pain: VAS (0-10) | immediate term (closest to 2 weeks) |
| Kanwal 2021 (1937) | Country: Pakistan  ROB: high | Number of participants: n=24 (E:14; C:10)  % female: 100  Mean age, years: 54.5 | Intervention: core strengthening (core strengthening + physical therapy (moist heat, TENS, strengthening exercise))  Comparison: no treatment (physical therapy; moist heat, TENS, strengthening exercise) | Pain: NPRS (0-10)  Function: ODI (0-50) | immediate term (closest to 2 weeks) |
| Karimi 2014 (1845) | Country: Iran  ROB: high | Number of participants: n=29 (E:15; C:14)  % female: 100  Mean age, years: E:21.1, C:25.9 | Intervention: motor control exercise  Comparison: no treatment | Pain: VAS (0-10)  Function: ODI (0-100)  Adverse events: N | immediate term (closest to 2 weeks) |
| Karimzadeh 2016 (41261) | Country: Iran  ROB: high | Number of participants: n=26 (E:13; C:13)  % female: 100  Mean age, years: E:43.6; C:41.7 | Intervention: core strengthening  Comparison: no treatment (not specified; assumed no treatment) | Pain: VAS (0-10)  Function: ODI (0-100) | immediate term (closest to 2 weeks) |
| Kell 2011 (45010) | Country: Canada (high)  ROB: high | Number of participants: n= 240 (E1:60; E2:60; E3:60; C:60)  % female: 33  Mean age, years: 42 | Intervention: general strength training (muscle strength training *3 exercise groups differed by dose of training)  Comparison: usual care | Pain: VAS (0-10)  Function: ODI (0-100) | immediate term (closest to 2 weeks) |
| Kim 2020 (47380) | Country: South Korea  ROB: high | Number of participants: n=66 (E1:24; E2:22; C:20)  % female: 48  Mean age, years: 47.4 | Intervention 1: stretching or flexibility/mobilizing  Intervention 2: muscle strength training  Comparison  Intervention: placebo/sham (gentle skin palpation) | Pain: VAS (0-10)  Function: RMDQ (0-24) | immediate term (closest to 2 weeks) |
| Kim 2020 (47385) | Country: Republic of Korea  ROB: high | Number of participants: n=29 (E:14; C:15)  % female: 100  Mean age, years: 21.1 | Intervention: core strengthening on a moving platform  Comparison: no treatment (same core strengthening on a non-operating platform) | Pain: VAS (0-10) | immediate term (closest to 2 weeks) |
| Lang 2021 (1942) | Country: Canada  ROB: high | Number of participants: n=174 (E:117; C:57)  % female: 40  Mean age, years: 46 | Intervention: aerobic (walking) + education and advice  Comparison: no treatment (education and advice) | Pain: ODI first question (0-5)  Function: ODI (0-50)  Adverse events: number of minor and major adverse events | immediate term (closest to 2 weeks)  short term (closest to 3 months) |
| Li 2015 (45015) | Country: China  ROB: high | Number of participants: n= 60 (E: 30 ; C: 30)  % female: E: 40; C: 47  Mean age, years (SD): E: 46.4 (2.3); C: 45.8 (2.1) | Intervention: Qigong + core strengthening (sling exercise)  Comparison: no treatment (core strengthening; sling exercise) | Pain: VAS 0-10  Function:  ODI (scale not reported) | immediate term (closest to 2 weeks) |
| Li 2021 (1892) | Country: China  ROB: high | Number of participants: n= 34 (E1: 11; E2: 12; C: 11)  % female: E1: 72.7; E2: 90; C: 63.6  Mean age, years (SD): E1: 21.9 (2,4); E2: 23.8 (4,1); C: 25.4 (3.7) | Intervention 1: motor control exercise (virtual reality exercise + thermal magnetic therapy)  Intervention 2: motor control exercise (motor control + thermal magnetic therapy)  Comparison: no treatment (thermal magnetic therapy) | Pain: VAS (0-10)  Function: ODI (0-50) | immediate term (closest to 2 weeks) |
| Liu 2018 (45038) | Country: China  ROB: high | Number of participants: n= 40 (E1: 14; E2: 13; C: 13)  % female: NR  Mean age, years (SD): E1: 58.9 (3.7); E2: 55.2 (2.0); C: 57.4 (3.0) | Intervention 1: Tai chi  Intervention 2: core strengthening  Comparison: no treatment/waitlist | Function: ODI (0-50) | short term (closest to 3 months) |
| Liu 2019 (44259) | Country: China  ROB: high | Number of participants: n= 43 (E1: 15; E2: 15; C: 13)  % female: E1: 73.3; E2: 73.3; C: 76.9  Mean age, years (SD): E1: 58.1 (5.4); E2: 58.4 (5.1); C: 60.7 (2.6) | Intervention 1: Tai chi  Intervention 2: core strengthening  Comparison: no treatment | Pain: VAS (0-10) | immediate term (closest to 2 weeks) |
| Madadi-Shad 2020 (47447) | Country: Iran  ROB: high | Number of participants: n= 36 (E: 18; C: 18 )  % female: 0  Mean age, years (SD): E: 68.0 (2.4); C: 68.9 (2.6) | Intervention: mixed (core strengthening, general strength training, stretching or flexibility/mobilizing exercise)  Comparison: no treatment/waitlist | Pain: VAS (0-100)  Function: RMDQ (0-24) | immediate term (closest to 2 weeks) |
| McIlveen 1998 (52017) | Country: Australia  ROB: high | Number of participants: n= 95 (E: 45; C: 50)  % female: E: 64; C: 60  Mean age, years (SD): E: 57.2 (15.2); C: 58.4 (15.0) | Intervention: mixed (aerobic, general strength training in water)  Comparison: no treatment/waitlist | Pain:  McGill Pain Questionnaire (1-5)  Function: ODI (0-100) | immediate term (closest to 2 weeks) |
| Mendes 2022 (53045) | Country: Brazil  ROB: high | Number of participants: n= 14 (E: 7; C: 7)  % female: NR  Mean age, years (SD): E: 27 (2); C: 27 (1) | Intervention: core strengthening (stabilization exercise; core strengthening; motor control exercise)  Comparison: usual care (regular exercise) | Pain: NPRS (0-10)  Function: ODI (0-100) | immediate term (closest to 2 weeks) |
| Miyamoto 2013 (9791) | Country: Brazil  ROB: unclear | Number of participants: n=86 (E: 43; C: 43)  % female: E: 83.7, C: 79.1  Mean age, years: E: 40.7 (11.8), C: 38.3 (11.4) | Intervention: Pilates (Pilates + education)  Comparison: no treatment (education) | Pain: NPRS (0-10)  Function: RMDQ (0-24)  Adverse events: methods not reported | immediate term (closest to 2 weeks) |
| Nardin 2022 (53154) | Country: Brazil  ROB: unclear | Number of participants: n=60 (E1: 20; E2: 20; C: 20)  % female: E1: 60, E2: 75, C: 56.9  Mean age, years: E1: 42.2 (9.1), E2: 42.8 (8.4), C: 43.1 (10.7) | Intervention: aerobic (deep water running) + photobiomodulation)  Comparison: no treatment (photobiomodulation) | Pain: VAS (0-10)  Function: ODI (0-100) | immediate term (closest to 2 weeks) |
| Noormohammadpour 2018 (43701) | Country: Iran  ROB: high | Number of participants: n=20 (E: 10; C: 10)  % female: 100%  Mean age, years: E: 43.3 (7.5), C: 41.3 (6.4) | Intervention: core strengthening  Comparison: no treatment/wait list | Pain: VAS (0-100)  Function: RMDQ (0-24) | immediate term (closest to 2 weeks) |
| Park 2020 (47631) | Country: Korea  ROB: high | Number of participants: n=80 (E: 40; C: 40)  % female: 100  Mean age, years: E: 72.1 (6.8), C: 71.5 (6.3) | Intervention: core strengthening (equine simulator riding)  Comparison: placebo/sham (sat on the horse and watched the video from the monitor) | Pain: VAS (0-10)  Function: ODI (0-100) | immediate term (closest to 2 weeks) (immediately post-intervention) |
| Prado 2019 (44352) | Country: Brazil  ROB: high | Number of participants: n=54 (E: 27; C: 27)  % female: E: 70.4, C: 63.0  Mean age, years: E: 35 (9.8), C: 33 (11.3) | Intervention: stretching/flexibility/mobilizing exercises  Comparison: no treatment/wait list | Pain: VAS (0-10)  Function: RMDQ (0-24) | immediate term (closest to 2 weeks) |
| Rahbar 2018 (43745) | Country: Iran  ROB: unclear | Number of participants: n=80 (E: 40; C: 40)  % female: E: 32.5, C: 27.5  Mean age, years: E: 46.25 (7.97), C: 46.22 (7.83) | Intervention: core strengthening, stretching/flexibility/mobilizing exercises + standardized conventional physical therapy  Comparison: no treatment (standardized conventional physical therapy) | Pain: VAS (0-100)  Function: RMDQ (0-100)  Adverse events: methods not reported | immediate term (closest to 2 weeks) |
| Raoul 2019 (46752) | Country: France  ROB: high | Number of participants: n=67 (E: 35; C: 32)  % female: 0  Mean age, years: E: 44.8 (11); C: 43.1 (11.6) | Intervention: core strengthening  Comparison: usual care (continue usual training) | Pain: NPRS 0-10 | immediate term (closest to 2 weeks) |
| Rathi 2013 (52020) | Country: India  ROB: high | Number of participants: n=30 (E: 15; C: 15)  % female: 100  Mean age, years: NR (only included 20 to 40 years based on inclusion criteria) | Intervention: core strengthening (core strengthening + conventional therapy)  Comparison: usual care (conventional therapy) | Pain: VAS 0-10  Function: ODI 0-100 | immediate term (closest to 2 weeks) |
| Raza 2020 (1601) | Country: Pakistan  ROB: high | Number of participants: n=40 (E: 20; C: 20)  % female: NR  Mean age, years: NR (age range in inclusion criteria was 20 to 60 years) | Intervention: stretching/flexibility/mobilizing exercises (core strengthening, stretching/flexibility/mobilizing exercises)  Comparison: no treatment (core strengthening) | Pain: NPRS (scale not reported)  Function: ODI (scale not reported) | immediate term (closest to 2 weeks) |
| Rotter 2022 (53220) | Country: Germany  ROB: unclear | Number of participants: n=55 (E: 29; C: 26)  % female: E: 82.8, C: 84.6  Mean age, years: E: 52.5 (8.7), C: 54.8 (7.5) | Intervention: aerobic, stretching/flexibility/mobilizing exercises (mindful walking)  Comparison: no treatment | Pain: VAS (0-100)  Function: Hannover Functional Ability Questionnaire (0-100)  Adverse events: measured across study period | immediate term (closest to 2 weeks)  short term (closest to 3 months) |
| Sedaghati 2017 (40972) | Country: Iran  ROB: high | Number of participants: n=68 (E1: 17; E2: 17; E3: 17; C: 17)  % female: 100  Mean age, years: E1: 24.1, E2: 24.5; E3: 23.5; C: 25.7 | Intervention 1: mixed (general strength training, stretching/flexibility/mobilizing exercise)  Intervention 2: stretching or flexibility/mobilizing (McKenizie)  Intervention 3: mixed (core strengthening, general strength training, stretching or flexibility/mobilizing exercise – William’s exercise)  Comparison: no treatment | Function: Quebec Back Pain Disability Scale (0-100) | immediate term (closest to 2 weeks) |
| Shamsi 2022 (53159) | Country: Iran  ROB: unclear | Number of participants: n=45 (E1: 15; E2: 15; C: 15)  % female: E1: 33.3, E2: 26.7, C: 33.3  Mean age, years: E1: 37.67 (8.96), E2: 37.07 (13.39), C: 39.12 (11.61) | Intervention 1: stretching or flexibility/mobilizing (+ physiotherapy)  Intervention 2: general strength training (+ physiotherapy)  Comparison: no treatment (physiotherapy) | Pain: VAS (0-100)  Function: ODI (0-100) | immediate term (closest to 2 weeks) |
| Shariat 2019 (46789) | Country: Iran  ROB: high | Number of participants: n=76 (E1: 19; E2: 19; C1: 19; C2: 19)  % female: NR  Mean age, years: NR | Intervention 1: stretching or flexibility/mobilizing  Intervention 2: stretching or flexibility/mobilizing (+ relaxation therapy)  Comparison 1: no treatment  Comparison 2: no treatment (relaxation therapy) | Pain: Functional Rating Test (0-40) | short term (closest to 3 months) |
| Shu 2021 (1603) | Country: China  ROB: high | Number of participants: n=94 (E: 47; C: 47)  % female: E: 38.3; C: 40.4  Mean age, years: E: 36.2 (7.5); C: 38.6 (7.7) | Intervention: core strength training (+ manipulation)  Comparison: no treatment (manipulation) | Pain: VAS (0-10)  Function: ODI (0-50) | short term (closest to 3 months) |
| Smeets 2008 (16226) | Country: Netherlands  ROB: low | Number of participants: n=119 (E: 61, C: 58)  % female: E: 37.7, C: 58.6  Mean age, years: E: 40.7 (10.1), C: 42.5 (9.7) | Intervention: mixed (aerobic, core strengthening (active physical treatment + graded activity with problem solving training))  Comparison: no treatment (graded activity with problem solving training) | Pain: VAS (0-100)  Function: RMDQ (0-24)  Adverse events: recorded when reported by participants | immediate term (closest to 2 weeks)  long term (closest to 12 months) |
| Sokhangoi 2017 (40938) | Country: Iran  ROB: high | Number of participants: n=34 (E: 17; C: 17)  % female: 100  Mean age, years: E: 49.7; C: 51.4 | Intervention: Pilates  Comparison: no treatment | Pain: VAS (0-100) | immediate term (closest to 2 weeks) |
| Srivastav 2018 (43827) | Country: Iran  ROB: high | Number of participants: n=30 (E: 15; C: 15)  % female: NR  Mean age, years: NR | Intervention: motor control exercise (+ electrotherapy and muscle stretching and strengthening)  Comparison: no treatment (electrotherapy and stretching and strengthening) | Pain: NPRS (scale not reported)  Function: modified ODI (0-50) | immediate term (closest to 2 weeks) |
| Suh 2019 (44414) | Country: South Korea  ROB: high | Number of participants: n=45 (E1: 15; C1: 15; C2: 15)  % female: E1: 85; E2: 60; C: 67%  Mean age, years: E1: 54.2 (13.9); E2: 57.4 (15.9); C: 54.8 (15.9) | Intervention: core strengthening; stretching or flexibility/mobilizing exercise + aerobic (walking)  Comparison 1: no treatment (core strengthening; stretching or flexibility/mobilizing exercise alone)  Comparison 2: no treatment (walking alone) | Pain: VAS (0-100)  Function: ODI (0-100) | immediate term (closest to 2 weeks)  short term (closest to 3 months): (pain only) |
| Takinaci 2019 (44427) | Country: Turkey  ROB: unclear | Number of participants: n=60 (E: 30; C: 30)  % female: E: 69.2, C: 75  Mean age, years: E: 64.5 (11.9), C: 60.33 (9.6) | Intervention: mixed (general strength training; stretching/flexibility/mobilizing exercises) + balneotherapy  Comparison: no treatment (balneotherapy) | Pain: VAS (0-100)  Function: WDI (0-9) | immediate term (closest to 2 weeks)  short term (closest to 3 months) |
| Tilbrook 2011 (17167) | Country: United Kingdom  ROB: unclear | Number of participants: n=313 (E: 156; C: 157)  % female: E: 68, C: 73  Mean age, years: E: 46.4 (11.3), C: 46.3 (11.5) | Intervention: yoga (Iyengar yoga + back book + usual care)  Comparison: usual care (back book + usual care) | Pain: Aberdeen Back Pain Scale (0-100)  Function: RMDQ (0-24)  Adverse events: recorded when reported by participants | immediate term (closest to 2 weeks)  long term (closest to 12 months): Y |
| Wattamwar 2012 (45028) | Country: India  ROB: high | Number of participants: n= 24 (E:12; C:12)  % female: NR  Mean age, years: E: 36; C: 32 | Intervention: yoga, core strengthening + conventional occupational therapy  Comparison: no treatment (conventional occupational therapy) | Function: ODI (0-50) | immediate term (closest to 2 weeks) |
| Weiner 2008 (6068) | Country: United states  ROB: unclear | Number of participants: n=200 (E1: 50; E2: 50; C1: 50; C2: 50)  % female: E1: 56, E2: 60, C1: 54, C2: 58  Mean age, years: E1: 73.9 (5.2), E2: 73.3 (6.0), C1: 74.3 (6.4); C2: 74.1 (5.6) | Intervention 1: mixed (aerobic; general strength training; stretching/flexibility/mobilizing exercises) + PENS  Intervention 2: mixed (aerobic; general strength training; stretching/flexibility/mobilizing exercises) + sham PENS  Comparison 1: no treatment (PENS)  Comparison 2: no treatment (sham PENS) | Pain: MPQ (0-45)  Function: RMDQ (0-24)  Adverse events: methods not reported | immediate term (closest to 2 weeks) |
| Xu 2021 (1814) | Country: China  ROB: high | Number of participants: n= 44 (E1:15; E2:14; C: 15)  % female: E1: 53.3; E2: 42.9, C: 46.7  Mean age, years: E1: 22.7 (2.8), E2: 22.4(1.9), C: 22.5 (2.0) | Intervention 1: motor control exercise (high intensity)  Intervention 2: motor control exercise (low intensity)  Comparison: sham | Pain: VAS (0-10) | immediate term (closest to 2 weeks) |
| Yalfani 2022 (53132) | Country: Iran  ROB: high | Number of participants: n= 25 (E: 13; C:12)  % female: 100  Mean age, years: E: 68 (2.9); C: 67.1 (2.9) | Intervention: mixed (aerobic; core strengthening; general strength training; stretching, or flexibility/mobilizing exercises; other – general exercises simulating sports, balance)  Comparison: no treatment | Pain: VAS (0-10) | immediate term (closest to 2 weeks) |
| Zadro 2019 (44491) | Country: Australia  ROB: high | Number of participants: n= 60 (E: 30; C: 30)  % female: 51.7  Mean age, years: 68.3 (5.7); E: 68.8 (5.5); C: 67.8 (6.0) | Intervention: mixed (aerobic; general strength training, stretching, or flexibility/mobilizing exercise; yoga; other - balance)  Comparison: usual care | Pain: NPRS (0-10)  Function: RMDQ (0-24)  Adverse events: reported by participants | immediate term (closest to 2 weeks) |
| Zakari 2019 (46901) | Country: Nigeria  ROB: high | Number of participants: n= 62 (E1:21; E2:21:, C:20)  % female: NR  Mean age, years: E1: 40.7 (10.9); E2: 35.6 (8.6); C: 33.1 (7.7) | Intervention 1: stretching/flexibility/mobilizing exercise  Intervention 2: core strengthening  Comparison: no treatment | Pain: VAS (0-10)  Function: ODI (0-100) | immediate term (closest to 2 weeks) |

First author last name and year (reference ID)

^†^We used the terms ‘female or male’ to describe gender because these were the terms used by study authors; however, we recognize that gender is a social construct and sex is a biological construct.2013

**C1:** Comparison treatment group 1, **E1:** Exercise treatment group 1, **MPQ:** McGill Pain Questionnaire, **NPRS:** numeric pain rating scale, **NR:** not reported, **ODI:** Oswestry Disability Index, **PENS:** Percutaneous electrical nerve stimulation, **PROMIS:** Patient-Reported Outcomes Measurement Information System, **RMDQ:** Roland-Morris Disability Questionnaire, **ROB:** risk of bias, **SD:** standard deviation, **TENS:** Transcutaneous electrical nerve stimulation, **VAS:** Visual Analogue Scale, **WDI:** Waddell Disability Index

**GRADE Evidence Profile tables**

GRADE Evidence Profile table 1: ***What are the benefits and harms of exercise in the management of community-dwelling adults (including older adults aged 60 years and over) with chronic primary low back pain (with or without leg pain) compared with no treatment/no additional treatment?***

| **Certainty assessment** | | | | | | | **№ of patients** | | **Effect** | | **Certainty** | **Importance** |
| --- | --- | --- | --- | --- | --- | --- | --- | --- | --- | --- | --- | --- |
| **№ of studies** | **Study design** | **Risk of bias** | **Inconsistency** | **Indirectness** | **Imprecision** | **Other considerations** | **exercise** | **no treatment** | **Relative (95% CI)** | **Absolute (95% CI)** |
| **Pain (follow-up: closest to 2 weeks; assessed with: VAS, NRS, ODI, MPQ; benefit indicated by lower values; Scale from: 0 to 10)** | | | | | | | | | | | | |
| 41^1,2,3,4,5,6,7,8,9,10,11,12,13,14,15,16,17,18,19,20,21,22,23,24,25,26,27,28,29,30,31,32,33,34,35,36,37,38,39,40,41,a,b,c,d,e,f,g,h^ | randomised trials | very serious^i^ | not serious^j^ | not serious^k^ | not serious^l^ | none | 1109 | 959 | - | MD **1.32 lower** (1.8 lower to 0.85 lower) | ⨁⨁◯◯ Low | CRITICAL |
| **Pain in adults (excluding aged 60+ years) (follow-up: closest to 2 weeks; assessed with: VAS, NRS, ODI; benefit indicated by lower values; Scale from: 0 to 10)** | | | | | | | | | | | | |
| 35^1,2,3,4,5,6,7,9,10,12,13,14,15,17,18,19,20,21,22,23,24,25,26,28,29,30,31,32,33,34,35,36,37,40,41,a,b,c,d,e,f,g,h^ | randomised trials | very serious^i^ | not serious^j^ | not serious^k^ | not serious^l^ | none | 943 | 793 | - | MD **1.2 lower** (1.7 lower to 0.69 lower) | ⨁⨁◯◯ Low | CRITICAL |
| **Pain in older adults (aged 60+ years) (follow-up: closest to 2 weeks; assessed with: VAS, NRS, MPQ; benefit indicated by lower values; Scale from: 0 to 10)** | | | | | | | | | | | | |
| 6^8,11,16,27,38,39^ | randomised trials | very serious^i^ | serious^m^ | not serious^k^ | serious^n^ | none | 166 | 166 | - | MD **2.31 lower** (3.37 lower to 1.24 lower) | ⨁◯◯◯ Very low | CRITICAL |
| **Pain in adults in high or upper-middle income countries (follow-up: closest to 2 weeks; assessed with: VAS, NRS, ODI, MPQ; benefit indicated by lower values; Scale from: 0 to 10)** | | | | | | | | | | | | |
| 22^1,8,9,10,11,12,14,15,16,20,22,23,24,25,26,28,29,31,33,36,37,38,a,b,c,d^ | randomised trials | very serious^i^ | not serious^o^ | not serious^p^ | not serious^l^ | none | 708 | 595 | - | MD **1.23 lower** (1.57 lower to 0.89 lower) | ⨁⨁◯◯ Low | CRITICAL |
| **Pain in adults in low or lower-middle income countries (follow-up: closest to 2 weeks; assessed with: VAS, NRS; benefit indicated by lower values; Scale from: 0 to 10)** | | | | | | | | | | | | |
| 19^2,3,4,5,6,7,13,17,18,19,21,27,30,32,34,35,39,40,41,a,e,f,g,h^ | randomised trials | very serious^i^ | serious^q^ | not serious^r^ | not serious^l^ | none | 401 | 364 | - | MD **1.41 lower** (2.23 lower to 0.59 lower) | ⨁◯◯◯ Very low | CRITICAL |
| **Pain (aerobic exercise) (follow-up: closest to 2 weeks; assessed with: VAS, NRS, ODI; benefit indicated by lower values; Scale from: 0 to 10)** | | | | | | | | | | | | |
| 9^1,6,8,9,19,23,29,33,36,a^ | randomised trials | very serious^i^ | serious^s^ | not serious^k^ | serious^t^ | none | 253 | 214 | - | MD **1.61 lower** (3.41 lower to 0.19 higher) | ⨁◯◯◯ Very low | CRITICAL |
| **Pain (core strengthening) (follow-up: closest to 2 weeks; assessed with: VAS; benefit indicated by lower values; Scale from: 0 to 10)** | | | | | | | | | | | | |
| 12^4,7,10,16,18,20,21,22,26,30,32,40,a,f,h^ | randomised trials | very serious^i^ | serious^u^ | not serious^k^ | serious^n^ | none | 196 | 177 | - | MD **1.52 lower** (2.02 lower to 1.01 lower) | ⨁◯◯◯ Very low | CRITICAL |
| **Pain (general/muscle strength training) (follow-up: closest to 2 weeks; assessed with: VAS; benefit indicated by lower values; Scale from: 0 to 10)** | | | | | | | | | | | | |
| 3^3,14,34,a^ | randomised trials | serious^v^ | serious^w^ | not serious^k^ | very serious^x^ | none | 92 | 84 | - | MD **0.61 higher** (1.62 lower to 2.84 higher) | ⨁◯◯◯ Very low | CRITICAL |
| **Pain (mixed exercise) (follow-up: closest to 2 weeks; assessed with: VAS, MPQ; benefit indicated by lower values; Scale from: 0 to 10)** | | | | | | | | | | | | |
| 7^11,12,27,36,37,38,39,a,b,c,d^ | randomised trials | very serious^i^ | serious^y^ | not serious^k^ | not serious^l^ | none | 250 | 203 | - | MD **1.52 lower** (2.58 lower to 0.47 lower) | ⨁◯◯◯ Very low | CRITICAL |
| **Pain (motor control exercise) (follow-up: closest to 2 weeks; assessed with: VAS, NRS; benefit indicated by lower values; Scale from: 0 to 10)** | | | | | | | | | | | | |
| 5^2,13,25,35,41,a^ | randomised trials | very serious^i^ | serious^z^ | not serious^k^ | very serious^x^ | none | 104 | 92 | - | MD **0.78 lower** (1.79 lower to 0.23 higher) | ⨁◯◯◯ Very low | CRITICAL |
| **Pain (Pilates) (follow-up: closest to 2 weeks; assessed with: NRS; benefit indicated by lower values; Scale from: 0 to 10)** | | | | | | | | | | | | |
| 1^28,e^ | randomised trials | serious^v^ | not serious^aa^ | serious^ab^ | very serious^x^ | none | 43 | 43 | - | MD **2.1 lower** (3.07 lower to 1.13 lower) | ⨁◯◯◯ Very low | CRITICAL |
| **Pain (Qigong) (follow-up: closest to 2 weeks; assessed with: VAS; benefit indicated by lower values; Scale from: 0 to 10)** | | | | | | | | | | | | |
| 2^15,24^ | randomised trials | very serious^i^ | not serious^ac^ | serious^ab^ | very serious^x^ | none | 60 | 60 | - | MD **0.93 lower** (1.45 lower to 0.4 lower) | ⨁◯◯◯ Very low | CRITICAL |
| **Pain (stretching or flexibility/mobilizing exercise) (follow-up: closest to 2 weeks; assessed with: VAS, NRS; benefit indicated by lower values; Scale from: 0 to 10)** | | | | | | | | | | | | |
| 5^5,17,31,34,40,a,g^ | randomised trials | very serious^i^ | not serious^ad^ | not serious^k^ | very serious^x^ | none | 96 | 79 | - | MD **1.52 lower** (2.08 lower to 0.95 lower) | ⨁◯◯◯ Very low | CRITICAL |
| **Pain (Tai Chi) (follow-up: closest to 2 weeks; assessed with: VAS; benefit indicated by lower values; Scale from: 0 to 10)** | | | | | | | | | | | | |
| 1^26^ | randomised trials | very serious^i^ | not serious^aa^ | serious^ab^ | very serious^x^ | none | 15 | 7 | - | MD **2.38 lower** (3.16 lower to 1.6 lower) | ⨁◯◯◯ Very low | CRITICAL |
| **Pain (low ROB trials) (follow-up: closest to 2 weeks; assessed with: VAS 0 to 100; benefit indicated by lower values)** | | | | | | | | | | | | |
| 1^42^ | randomised trials | not serious^ae^ | not serious^aa^ | serious^ab^ | very serious^x^ | none | Smeets 2008: 119 participants total. Mixed exercise vs. no/no additional treatment. Participants performed combination treatment (active physical treatment [aerobic and core strengthening exercises] + graded activity with problem solving training) vs. graded activity with problem solving training alone. Between-group MD (VAS 0-100) graded activity with problem solving training alone vs. combination treatment = 5.35, 95% CI -3.73 to 14.42. | | | | ⨁◯◯◯ Very low | CRITICAL |
| **Pain (follow-up: closest to 3 months; assessed with: VAS, ODI; benefit indicated by lower values; Scale from: 0 to 10)** | | | | | | | | | | | | |
| 5^23,33,36,37,43,a^ | randomised trials | very serious^i^ | not serious^af^ | not serious^p^ | serious^n^ | none | 191 | 156 | - | MD **0.54 lower** (0.88 lower to 0.2 lower) | ⨁◯◯◯ Very low | CRITICAL |
| **Pain in older adults (aged 60+ years) (follow-up: closest to 3 months)** | | | | | | | | | | | | |
| 0 |  |  |  |  |  |  |  |  |  |  |  | CRITICAL |
| **Pain in adults in low or lower-middle income countries (follow-up: closest to 3 months)** | | | | | | | | | | | | |
| 0 |  |  |  |  |  |  |  |  |  |  |  | CRITICAL |
| **Pain (aerobic exercise) (follow-up: closest to 3 months; assessed with: VAS, ODI; benefit indicated by lower values; Scale from: 0 to 10)** | | | | | | | | | | | | |
| 3^23,33,36,a^ | randomised trials | very serious^i^ | not serious^af^ | not serious^p^ | serious^n^ | none | 111 | 70 | - | MD **0.73 lower** (1.35 lower to 0.11 lower) | ⨁◯◯◯ Very low | CRITICAL |
|  | | | | | | | | | | | | |
|  | | | | | | | | | | | | |
| **Pain (core strengthening) (follow-up: closest to 3 months; assessed with: VAS; benefit indicated by lower values; Scale from: 0 to 10)** | | | | | | | | | | | | |
| 1^43^ | randomised trials | very serious^i^ | not serious^aa^ | serious^ab^ | very serious^x^ | none | 47 | 47 | - | MD **0.53 lower** (0.97 lower to 0.09 lower) | ⨁◯◯◯ Very low | CRITICAL |
| **Pain (mixed exercise) (follow-up: closest to 3 months; assessed with: VAS; benefit indicated by lower values; Scale from: 0 to 10)** | | | | | | | | | | | | |
| 2^36,37,a^ | randomised trials | serious^v^ | not serious^af^ | not serious^p^ | very serious^x^ | none | 33 | 39 | - | MD **0.05 lower** (1.13 lower to 1.02 higher) | ⨁◯◯◯ Very low | CRITICAL |
| **Pain (low ROB trials) (follow-up: closest to 3 months)** | | | | | | | | | | | | |
| 0 |  |  |  |  |  |  |  |  |  |  |  | CRITICAL |
| **Pain (follow-up: closest to 12 months; assessed with: VAS; benefit indicated by lower values; Scale from: 0 to 10)** | | | | | | | | | | | | |
| 1^14,ag^ | randomised trials | serious^v^ | not serious^aa^ | serious^ab^ | very serious^x^ | none | 35 | 35 | - | MD **0.1 lower** (1.32 lower to 1.12 higher) | ⨁◯◯◯ Very low | CRITICAL |
| **Pain in older adults (aged 60+ years) (follow-up: closest to 12 months)** | | | | | | | | | | | | |
| 0 |  |  |  |  |  |  |  |  |  |  |  | CRITICAL |
| **Pain in adults in low or lower-middle income countries (follow-up: closest to 12 months)** | | | | | | | | | | | | |
| 0 |  |  |  |  |  |  |  |  |  |  |  | CRITICAL |
|  | | | | | | | | | | | | |
|  | | | | | | | | | | | | |
|  | | | | | | | | | | | | |
|  | | | | | | | | | | | | |
| **Pain (general/muscle strength training) (follow-up: closest to 12 months; assessed with: benefit indicated by lower values; Scale from: 0 to 10)** | | | | | | | | | | | | |
| 1^14^ | randomised trials | serious^v^ | not serious^aa^ | serious^ab^ | very serious^x^ | none | 35 | 35 | - | MD **0.1 lower** (1.32 lower to 1.12 higher) | ⨁◯◯◯ Very low | CRITICAL |
| **Pain (mixed exercise, low ROB trial) (follow-up: closest to 12 months; assessed with: VAS 0-100; benefit indicated by lower values)** | | | | | | | | | | | | |
| 1^42^ | randomised trials | not serious^ae^ | not serious^aa^ | serious^ab^ | very serious^x^ | none | Smeets 2008 (119 participants). Participants performed combination treatment (active physical treatment [aerobic and core strengthening exercises] + graded activity with problem solving training) vs. graded activity with problem solving training alone. Between-group MD (VAS 0-100) graded activity with problem solving training alone vs. combination treatment = 6.25, 95% CI -2.94 to 15.44. | | | | ⨁◯◯◯ Very low | CRITICAL |
| **Function (follow-up: closest to 2 weeks; assessed with: RMDQ, ODI, modified ODI, Quebec Back Pain Disability Scale, Hannover, PROMIS, WI; benefit indicated by lower values)** | | | | | | | | | | | | |
| 39^1,2,3,4,5,6,7,8,9,10,12,13,14,15,16,17,18,19,21,23,24,25,27,28,29,30,31,32,33,34,35,36,37,38,40,41,44,45,46,a,ah,ai,aj,ak,al,am,an,ao,ap,aq^ | randomised trials | very serious^i^ | serious^ar^ | not serious^k^ | not serious^l^ | none | 1077 | 956 | - | SMD **0.8 lower** (1.07 lower to 0.53 lower) | ⨁◯◯◯ Very low | CRITICAL |
|  | | | | | | | | | | | | |
|  | | | | | | | | | | | | |
|  | | | | | | | | | | | | |
|  | | | | | | | | | | | | |
|  | | | | | | | | | | | | |
| **Function in adults (excluding aged 60+ years) (follow-up: closest to 2 weeks; assessed with: RMDQ, ODI, modified ODI, Quebec Back Pain Disability Scale, Hannover, PROMIS, WI; benefit indicated by lower values)** | | | | | | | | | | | | |
| 35^1,2,3,4,5,6,7,9,10,12,13,14,15,17,18,19,21,23,24,25,28,29,30,31,32,33,34,35,36,37,40,41,44,45,46,a,ah,ai,aj,ak,al,am,an,ao,ap,aq^ | randomised trials | very serious^i^ | serious^ar^ | not serious^k^ | not serious^l^ | none | 933 | 811 | - | SMD **0.8 lower** (1.1 lower to 0.5 lower) | ⨁◯◯◯ Very low | CRITICAL |
| **Function in older adults (aged 60+ years) (follow-up: closest to 2 weeks; assessed with: RMDQ, ODI; benefit indicated by lower values)** | | | | | | | | | | | | |
| 4^8,16,27,38,a^ | randomised trials | very serious^i^ | serious^as^ | not serious^k^ | serious^n^ | none | 144 | 145 | - | SMD **0.85 lower** (1.66 lower to 0.04 lower) | ⨁◯◯◯ Very low | CRITICAL |
| **Function in adults in high or upper-middle income countries (follow-up: closest to 2 weeks; assessed with: RMDQ, ODI, Hannover, PROMIS, WI; benefit indicated by lower values)** | | | | | | | | | | | | |
| 18^1,8,9,10,12,14,15,16,23,24,25,28,29,31,33,36,37,38,a,ah,ai,aj,am,ap^ | randomised trials | very serious^i^ | not serious^o^ | not serious^p^ | not serious^l^ | none | 637 | 544 | - | SMD **0.48 lower** (0.7 lower to 0.27 lower) | ⨁⨁◯◯ Low | CRITICAL |
| **Function in adults in low or lower-middle income countries (follow-up: closest to 2 weeks; assessed with: RMDQ, ODI, modified ODI, Quebec Back Pain Disability Scale; benefit indicated by lower values)** | | | | | | | | | | | | |
| 21^2,3,4,5,6,7,13,17,18,19,21,27,30,32,34,35,40,41,44,45,46,a,ak,al,an,ao,aq^ | randomised trials | very serious^i^ | not serious^at^ | not serious^r^ | not serious^l^ | none | 440 | 412 | - | SMD **1.19 lower** (1.74 lower to 0.64 lower) | ⨁⨁◯◯ Low | CRITICAL |
|  | | | | | | | | | | | | |
|  | | | | | | | | | | | | |
| **Function (aerobic exercise) (follow-up: closest to 2 weeks; assessed with: RMDQ, ODI, Quebec Back Pain Disability Scale, Hannover, PROMIS; benefit indicated by lower values)** | | | | | | | | | | | | |
| 10^1,6,8,9,19,23,29,33,36,44,a^ | randomised trials | very serious^i^ | not serious^au^ | not serious^k^ | not serious^l^ | none | 263 | 224 | - | SMD **0.98 lower** (1.51 lower to 0.45 lower) | ⨁⨁◯◯ Low | CRITICAL |
| **Function (core strengthening) (follow-up: closest to 2 weeks; assessed with: RMDQ, ODI; benefit indicated by lower values)** | | | | | | | | | | | | |
| 10^4,7,10,16,18,21,30,32,40,45,a,ak,ap,aq^ | randomised trials | very serious^i^ | not serious^av^ | not serious^k^ | serious^n^ | none | 186 | 178 | - | SMD **1.08 lower** (1.47 lower to 0.69 lower) | ⨁◯◯◯ Very low | CRITICAL |
| **Function (general/muscle strength training) (follow-up: closest to 2 weeks; assessed with: RMDQ, ODI; benefit indicated by lower values)** | | | | | | | | | | | | |
| 3^3,14,34,a^ | randomised trials | serious^v^ | serious^aw^ | not serious^k^ | very serious^x^ | none | 92 | 84 | - | SMD **1.09 higher** (0.99 lower to 3.17 higher) | ⨁◯◯◯ Very low | CRITICAL |
| **Function (mixed exercise) (follow-up: closest to 2 weeks; assessed with: RMDQ, ODI, WI; benefit indicated by lower values)** | | | | | | | | | | | | |
| 6^12,27,36,37,38,46,a,ah,ai,aj,am,an,ao^ | randomised trials | very serious^i^ | serious^ax^ | not serious^k^ | not serious^l^ | none | 233 | 196 | - | SMD **0.83 lower** (1.38 lower to 0.29 lower) | ⨁◯◯◯ Very low | CRITICAL |
|  | | | | | | | | | | | | |
|  | | | | | | | | | | | | |
| **Function (motor control exercise) (follow-up: closest to 2 weeks; assessed with: RMDQ, ODI, modified ODI; benefit indicated by lower values)** | | | | | | | | | | | | |
| 5^2,13,25,35,41,a^ | randomised trials | very serious^i^ | serious^ay^ | not serious^k^ | very serious^x^ | none | 104 | 92 | - | SMD **0.82 lower** (1.65 lower to 0.02 higher) | ⨁◯◯◯ Very low | CRITICAL |
| **Function (Pilates) (follow-up: closest to 2 weeks; assessed with: RMDQ; benefit indicated by lower values)** | | | | | | | | | | | | |
| 1^28^ | randomised trials | serious^v^ | not serious^aa^ | serious^ab^ | very serious^x^ | none | 43 | 43 | - | SMD **0.74 lower** (1.18 lower to 0.3 lower) | ⨁◯◯◯ Very low | CRITICAL |
| **Function (Qigong) (follow-up: closest to 2 weeks; assessed with: ODI; benefit indicated by lower values)** | | | | | | | | | | | | |
| 2^15,24^ | randomised trials | very serious^i^ | not serious^az^ | serious^ab^ | very serious^x^ | none | 60 | 60 | - | SMD **1.16 lower** (1.87 lower to 0.45 lower) | ⨁◯◯◯ Very low | CRITICAL |
| **Function (stretching or flexibility/mobilizing exercise) (follow-up: closest to 2 weeks; assessed with: RMDQ, ODI; benefit indicated by lower values)** | | | | | | | | | | | | |
| 5^5,17,31,34,40,a,al,ao^ | randomised trials | very serious^i^ | serious^ba^ | not serious^k^ | very serious^x^ | none | 96 | 79 | - | SMD **0.62 lower** (1.36 lower to 0.13 higher) | ⨁◯◯◯ Very low | CRITICAL |
|  | | | | | | | | | | | | |
|  | | | | | | | | | | | | |
| **Function (Tai Chi) (follow-up: closest to 2 weeks; assessed with: ODI 0-50; benefit indicated by lower values)** | | | | | | | | | | | | |
| 1^47^ | randomised trials | very serious^i^ | serious^aa^ | serious^ab^ | very serious^x^ | none | Liu 2018: 43 participants total. Authors reported the average ODI score in each domain of Tai Chi group decreased significantly compared to comparison group (overall scores not reported). | | | | ⨁◯◯◯ Very low |  |
| **Function (low ROB trials) (follow-up: closest to 2 weeks)** | | | | | | | | | | | | |
| 1^42^ | randomised trials | not serious^ae^ | not serious^aa^ | serious^ab^ | very serious^x^ | none | Smeets 2008 (119 participants). Participants performed combination treatment (active physical treatment [aerobic and core strengthening exercises] + graded activity with problem solving training) vs. graded activity with problem solving training alone. Between-group MD (RMDQ 0-24) graded activity with problem solving training alone vs. combination treatment = 0.58, 95% CI -1.08 to 2.24. | | | | ⨁◯◯◯ Very low | CRITICAL |
| **Function (follow-up: closest to 3 months; assessed with: ODI, Hannover, Functional Rating Test, WI; benefit indicated by lower values)** | | | | | | | | | | | | |
| 5^23,33,37,43,48,a^ | randomised trials | very serious^i^ | serious^as^ | not serious^k^ | serious^n^ | none | 211 | 163 | - | SMD **0.99 lower** (1.69 lower to 0.3 lower) | ⨁◯◯◯ Very low | CRITICAL |
| **Function in older adults (aged 60+ years) (follow-up: closest to 3 months)** | | | | | | | | | | | | |
| 0 |  |  |  |  |  |  |  |  |  |  |  |  |
|  | | | | | | | | | | | | |
|  | | | | | | | | | | | | |
|  | | | | | | | | | | | | |
|  | | | | | | | | | | | | |
| **Function in adults in high or upper-middle income countries (follow-up: closest to 3 months; assessed with: ODI, Hannover, WI; benefit indicated by lower values)** | | | | | | | | | | | | |
| 4^23,33,37,43^ | randomised trials | very serious^i^ | not serious^af^ | not serious^p^ | serious^n^ | none | 173 | 129 | - | SMD **0.43 lower** (0.66 lower to 0.19 lower) | ⨁◯◯◯ Very low | CRITICAL |
| **Function in adults in low or lower-middle income countries (follow-up: closest to 3 months; assessed with: Functional Rating Test; benefit indicated by lower values)** | | | | | | | | | | | | |
| 1^48,a^ | randomised trials | very serious^i^ | not serious^aa^ | serious^bb^ | very serious^x^ | none | 38 | 34 | - | SMD **2.87 lower** (6.68 lower to 0.93 higher) | ⨁◯◯◯ Very low | CRITICAL |
| **Function (aerobic exercise) (follow-up: closest to 3 months; assessed with: ODI, Hannover; benefit indicated by lower values)** | | | | | | | | | | | | |
| 2^23,33^ | randomised trials | very serious^i^ | not serious^af^ | not serious^p^ | very serious^x^ | none | 102 | 56 | - | SMD **0.27 lower** (0.6 lower to 0.07 higher) | ⨁◯◯◯ Very low | CRITICAL |
| **Function (core strengthening) (follow-up: closest to 3 months; assessed with: ODI; benefit indicated by lower values)** | | | | | | | | | | | | |
| 1^43^ | randomised trials | very serious^i^ | not serious^aa^ | serious^ab^ | very serious^x^ | none | 47 | 47 | - | SMD **0.66 lower** (1.07 lower to 0.24 lower) | ⨁◯◯◯ Very low | CRITICAL |
|  | | | | | | | | | | | | |
|  | | | | | | | | | | | | |
| **Function (mixed exercise) (follow-up: closest to 3 months; assessed with: WI; benefit indicated by lower values)** | | | | | | | | | | | | |
| 1^37^ | randomised trials | serious^v^ | not serious^aa^ | serious^ab^ | very serious^x^ | none | 24 | 26 | - | SMD **0.44 lower** (1.01 lower to 0.12 higher) | ⨁◯◯◯ Very low | CRITICAL |
| **Function (stretching or flexibility/mobilizing exercise) (follow-up: closest to 3 months; assessed with: Functional Rating Scale (unspecified scale range); benefit indicated by lower values)** | | | | | | | | | | | | |
| 1^48,a^ | randomised trials | very serious^i^ | not serious^aa^ | serious^bb^ | very serious^x^ | none | 38 | 34 | - | SMD **2.87 lower** (6.68 lower to 0.93 higher) | ⨁◯◯◯ Very low | CRITICAL |
| **Function (low ROB trials) (follow-up: closest to 3 months)** | | | | | | | | | | | | |
| 0 |  |  |  |  |  |  |  |  |  |  |  | CRITICAL |
| **Function (follow-up: closest to 12 months; assessed with: RMDQ; benefit indicated by lower vales; Scale from: 0 to 24)** | | | | | | | | | | | | |
| 1^14,bc^ | randomised trials | serious^v^ | not serious^aa^ | serious^ab^ | very serious^x^ | none | 35 | 35 | - | MD **0.2 lower** (2.73 lower to 2.33 higher) | ⨁◯◯◯ Very low | CRITICAL |
| **Function in older adults (aged 60+ years) (follow-up: closest to 12 months)** | | | | | | | | | | | | |
| 0 |  |  |  |  |  |  |  |  |  |  |  | CRITICAL |
| **Function in adults in low or lower-middle income countries (follow-up: closest to 12 months)** | | | | | | | | | | | | |
| 0 |  |  |  |  |  |  |  |  |  |  |  | CRITICAL |
|  | | | | | | | | | | | | |
|  | | | | | | | | | | | | |
|  | | | | | | | | | | | | |
| **Function (general strength training) (follow-up: closest to 12 months; assessed with: RMDQ; benefit indicated by lower values; Scale from: 0 to 24)** | | | | | | | | | | | | |
| 1^14^ | randomised trials | serious^v^ | not serious^aa^ | serious^ab^ | very serious^x^ | none | 35 | 35 | - | MD **0.2 lower** (2.73 lower to 2.33 higher) | ⨁◯◯◯ Very low | CRITICAL |
| **Function (mixed exercise, low ROB trial) (follow-up: closest to 12 months; assessed with: RMDQ 0-24; benefit indicated by lower values)** | | | | | | | | | | | | |
| 1^42^ | randomised trials | not serious^ae^ | not serious^aa^ | serious^ab^ | very serious^x^ | none | Smeets 2008: 119 participants. Participants performed combination treatment (active physical treatment [aerobic and core strengthening exercises] + graded activity with problem solving training) vs. graded activity with problem solving training alone. Between-group MD (RMDQ 0-24) graded activity with problem solving training alone vs. combination treatment = 1.11, 95% CI -0.56 to 2.79. | | | | ⨁◯◯◯ Very low | CRITICAL |
|  | | | | | | | | | | | | |
|  | | | | | | | | | | | | |
|  | | | | | | | | | | | | |
|  | | | | | | | | | | | | |
|  | | | | | | | | | | | | |
|  | | | | | | | | | | | | |
|  | | | | | | | | | | | | |
|  | | | | | | | | | | | | |
|  | | | | | | | | | | | | |
|  | | | | | | | | | | | | |
|  | | | | | | | | | | | | |
| **Harms** | | | | | | | | | | | | |
| 6^23,28,32,33,38,42^ | randomised trials | serious^v^ | not serious | not serious | not serious | none | Lang 2021 (aerobic exercise; 174 participants total): no harms reported. Miyamoto 2013 (Pilates; 86 participants total): no harms reported. Rahbar 2018 (core strengthening; 80 participants total): no harms reported. Rotter 2022 (aerobic exercise; 55 participants total): no harms reported. Smeets 2008 (mixed exercise; 119 participants total): 3 (5%) of participants in exercise group had increased back pain. Weiner 2008 (older adults) (mixed exercise; 200 participants total): no significant intervention-associated adverse events reported. One participant (2%) had increased back pain. One participant (2%) had decreased functional status. | | | | ⨁⨁⨁◯ Moderate | CRITICAL |

**CI:** confidence interval; **Hannover:** Hannover Functional Ability Questionnaire; **MD:** mean difference; **MPQ:** McGill Pain Questionnaire; **NRS:** Numerical Rating Scale; **ODI:** Oswestry Disability Index; **PROMIS:** Patient-Reported Outcomes Measurement Information System; **PSFS:** Patient-Specific Functional Scale; **RMDQ:** Roland Morris Disability Questionnaire; **SMD:** standardised mean difference; **VAS:** Visual Analog Scale; **WI:** Waddell Disability Index

#### Explanations

a. Comparison groups were split for trials with multiple comparisons.

b. Dalichau 2003: not included in meta-analysis due to missing data. Rated as high overall risk of bias; 90 participants total. Mixed exercise vs. no/no additional treatment: authors reported greater pain reduction in exercise group (unclear effect estimates).

c. McIlveen 1998: not included in meta-analysis due to missing data. Rated as high overall risk of bias; 95 participants total. Mixed exercise vs. no/no additional treatment: no significant difference in the number of participants who improved more than 1 point between exercise and comparison; p=0.13 (McGill Pain Questionnaire 1-5, benefit indicated by lower values).

d. Smeets 2008: not included in meta-analysis due to missing data. Rated as low overall risk of bias; 119 participants total. Mixed exercise vs. no/no additional treatment. Participants performed combination treatment (active physical treatment [aerobic and core strengthening exercises] + graded activity with problem solving training) vs. graded activity with problem solving training alone. Between-group MD (VAS 0-100, benefit indicated by lower values) graded activity with problem solving training alone vs. combination treatment = 5.35, 95% CI -3.73 to 14.42.

e. Sokhanguei 2017: not included in meta-analysis due to missing data. Rated as high overall risk of bias; 34 participants total. Pilates exercise vs. no/no additional treatment. Authors reported greater pain reduction in Pilates group; mean difference (SEM): -2.3 (0.72); p=0.003.

f. Kanwal 2021: not included in meta-analysis due to missing data. Rated as high overall risk of bias; 24 participants total. Core strengthening vs. no/no additional treatment. Authors reported no significant difference in pain between groups; p=0.317.

g. Raza 2020: not included in meta-analysis due to missing data. 40 participants, rated as overall high risk of bias, stretching, or flexibility/mobilizing exercise. Authors reported no significant difference in median pain between groups; p=0.112.

h. Rathi 2013: not included in meta-analysis due to missing data. 30 participants, rated as overall high risk of bias, core strengthening. Authors reported significantly greater mean pain reduction in exercise group (3.8, SD 1.0) than in no treatment group (2.9, SD 0.8); p < 0.05 (VAS 0-10, benefit indicated by lower values).

i. Risk of bias: We downgraded twice. Most or all trials were rated as overall high risk of bias.

j. Inconsistency: We did not downgrade. There is similarity in the majority of point estimates with overlapping confidence intervals. Statistical heterogeneity is between 75% and 100% (i.e., I2 = 97%); this could not be explained due to small subgroups and may represent considerable heterogeneity.

k. Indirectness: We did not downgrade. Trials conducted in different countries both high and low income.

l. Imprecision: We did not downgrade. OIS would have been reached. The point estimate reached the pre-specified threshold for what may be considered appreciable benefit (MD = -1 or SMD = -0.2); the confidence interval does not cross the null.

m. Inconsistency: We downgraded once. There is similarity in some of the point estimates with overlapping confidence intervals. Statistical heterogeneity is between 75% and 100% (i.e., I2 = 97%); this could not be explained due to small subgroups and may represent considerable heterogeneity.

n. Imprecision: We downgraded once due to low sample size (OIS would not have been reached).

o. Inconsistency: We did not downgrade. There is similarity in the majority of point estimates with overlapping confidence intervals. Statistical heterogeneity is between 50% and 90% (i.e., I2 = 65%); this could not be explained due to small subgroups and may represent substantial heterogeneity.

p. Indirectness: We did not downgrade. Trials conducted in different high-income countries.

q. Inconsistency: We downgraded once. There is similarity in some of the point estimates with overlapping confidence intervals. Statistical heterogeneity is between 75% and 100% (i.e., I2 = 98%); this could not be explained due to small subgroups and may represent considerable heterogeneity.

r. Indirectness: We did not downgrade. Trials conducted in different low-income countries.

s. Inconsistency: We downgraded once. There is similarity in some of the point estimates with overlapping confidence intervals. Statistical heterogeneity is between 75% and 100% (i.e., I2 = 99%); this could not be explained due to small subgroups and may represent considerable heterogeneity.

t. Imprecision: We downgraded once. OIS would have been reached. The point estimate reached the pre-specified threshold for what may be considered appreciable benefit (MD = -1 or SMD = -0.2); the confidence interval crosses the null.

u. Inconsistency: We downgraded once. There is similarity in some of the point estimates with overlapping confidence intervals. Statistical heterogeneity is between 50% and 90% (i.e., I2 = 73%); this could not be explained due to small subgroups and may represent substantial heterogeneity.

v. Risk of bias: We downgraded once. Some of the weight (>50%) comes from trials with unclear risk of bias.

w. Inconsistency: We downgraded once. There is similarity in some of the point estimates with overlapping confidence intervals. Statistical heterogeneity is between 75% and 100% (i.e., I2 = 95%); this could not be explained due to small subgroups and may represent considerable heterogeneity.

x. Imprecision: We downgraded twice due to low sample size (OIS would not have been reached).

y. Inconsistency: We downgraded once. There is similarity in some of the point estimates with overlapping confidence intervals. Statistical heterogeneity is between 75% and 100% (i.e., I2 = 82%); this could not be explained due to small subgroups and may represent considerable heterogeneity.

z. Inconsistency: We downgraded once. There is similarity in some of the point estimates with overlapping confidence intervals. Statistical heterogeneity is between 50% and 90% (i.e., I2 = 90%); this could not be explained due to small subgroups and may represent substantial heterogeneity.

aa. Inconsistency: We did not downgrade; however, there are no additional trials with which to compare findings.

ab. Indirectness: We downgraded once. Trial(s) conducted in one country (high income).

ac. Inconsistency: We did not downgrade. There is similarity in the point estimates with overlapping confidence intervals. Statistical heterogeneity is between 30% and 60% (i.e., I2 = 43%); this could not be explained due to small subgroups and may represent moderate heterogeneity.

ad. Inconsistency: We did not downgrade. There is similarity in the point estimates with overlapping confidence intervals. Statistical heterogeneity is between 30% and 60% (i.e., I2 = 32%); this could not be explained due to small subgroups and may represent moderate heterogeneity.

ae. Risk of bias: We did not downgrade. Trial(s) rated as overall low risk of bias.

af. Inconsistency: We did not downgrade. The point estimates are similar with overlapping confidence intervals; statistical heterogeneity is between 0% and 40%, which might not be important (i.e., I2 = 0%).

ag. Smeets 2008 was not included in the meta-analysis (provided within-group mean changes; no follow-up scores). 119 participants, rated as overall low risk of bias. Participants performed combination treatment (active physical treatment [aerobic and core strengthening exercises] + graded activity with problem solving training) vs. graded activity with problem solving training alone. Between-group MD (VAS 0-100, benefit indicated by lower values) graded activity with problem solving training alone vs. combination treatment = 6.25, 95% CI -2.94 to 15.44.

ah. Smeets 2008 was not included in the meta-analysis (provided within-group mean changes; no follow-up scores). 119 participants, rated as overall low risk of bias. Participants performed combination treatment (active physical treatment [aerobic and core strengthening exercises] + graded activity with problem solving training) vs. graded activity with problem solving training alone. Between-group MD (RMDQ 0-24, benefit indicated by lower values) graded activity with problem solving training alone vs. combination treatment = 0.58, 95% CI -1.08 to 2.24.

ai. Dalichau 2003: not included in meta-analysis due to missing data. Rated as high overall risk of bias; 90 participants total. Mixed exercise vs. no/no additional treatment: authors reported greater disability improvement in exercise group (unclear effect estimates).

aj. McIlveen 1998: not included in meta-analysis due to missing data. Rated as high overall risk of bias; 95 participants total. Mixed exercise vs. no/no additional treatment: authors reported significantly greater number of participants improved more than 10 points in the exercise group (27%) than in the no treatment group (8%); p=0.04 (ODI 0-100).

ak. Kanwal 2021: not included in meta-analysis due to missing data. Rated as high overall risk of bias; 24 participants total. Core strengthening vs. no/no additional treatment. Authors reported no significant difference in disability between groups; p=0.692.

al. Raza 2020: not included in meta-analysis due to missing data. 40 participants, rated as overall high risk of bias, stretching, or flexibility/mobilizing exercise. Authors reported significantly lower median item scores in the exercise group for personal care (exercise: median 1, IQR 0; no treatment: median 1, IQR 1; p=0.041) and travelling (exercise: median 1, IQR 0; no treatment: median 1, IQR 0; p=0.027); no significant difference for other items (ODI individual items; 0-5).

am. Da Silva 2014: not included in meta-analysis due to missing data. 18 participants total, rated as overall high risk of bias, mixed exercise. Authors reported significantly greater mean % improvement from baseline in exercise group (45% improvement) vs. no exercise (2% worsening); p=0.008 (RMDQ 0-24, benefit indicated by lower values).

an. Wattamwar 2012: not included in meta-analysis due to missing data. 24 participants total, rated as overall high risk of bias, yoga exercise. Authors reported no significant difference in change scores between groups; p=0.146.

ao. Sedaghati 2017: not included in meta-analysis due to missing data. 34 participants total, rated as overall high risk of bias, mixed exercise (in and out of water) and stretching or flexibility/mobilizing exercise. Authors reported a significant difference in follow-up scores between mixed exercise (mean 23.0, SD 3.0) and no treatment (mean 27.5, SD 3.0) (Quebec Back Pain Disability Scale 0-100, benefit indicated by lower values). No significant difference in follow-up scores between stretching or flexibility/mobilizing group and no treatment.

ap. Liu 2018: not included in meta-analysis due to missing data. 43 participants total, rated as overall high risk of bias, Tai Chi and core strengthening. Authors reported the average ODI score in each domain of both exercise groups decreased significantly compared to comparison group (overall scores not reported) (ODI 0-50, benefit indicated by lower values).

aq. Rathi 2013: not included in meta-analysis due to missing data. 30 participants total, rated as overall high risk of bias, core strengthening. Authors reported significantly greater mean disability improvement in exercise group (24.1, SD 3.2) than in no treatment group (19.73, SD 3.58); p < 0.05 (ODI 0-100; benefit indicated by lower values).

ar. Inconsistency: We downgraded once. There is similarity in some of the point estimates with overlapping confidence intervals. Statistical heterogeneity is between 75% and 100% (i.e., I2 = 87%); this could not be explained due to small subgroups and may represent considerable heterogeneity.

as. Inconsistency: We downgraded once. There is similarity in some of the point estimates with overlapping confidence intervals. Statistical heterogeneity is between 75% and 100% (i.e., I2 = 89%); this could not be explained due to small subgroups and may represent considerable heterogeneity.

at. Inconsistency: We did not downgrade. There is similarity in the majority of point estimates with overlapping confidence intervals. Statistical heterogeneity is between 75% and 100% (i.e., I2 = 92%); this could not be explained due to small subgroups and may represent considerable heterogeneity.

au. Inconsistency: We did not downgrade. There is similarity in the majority of point estimates with overlapping confidence intervals. Statistical heterogeneity is between 75% and 100% (i.e., I2 = 84%); this could not be explained due to small subgroups and may represent considerable heterogeneity.

av. Inconsistency: We did not downgrade. There is similarity in the majority of point estimates with overlapping confidence intervals. Statistical heterogeneity is between 50% and 90% (i.e., I2 = 63%); this could not be explained due to small subgroups and may represent substantial heterogeneity.

aw. Inconsistency: We downgraded once. There is similarity in some of the point estimates with overlapping confidence intervals. Statistical heterogeneity is between 75% and 100% (i.e., I2 = 97%); this could not be explained due to small subgroups and may represent considerable heterogeneity.

ax. Inconsistency: We downgraded once. There is similarity in some of the point estimates with overlapping confidence intervals. Statistical heterogeneity is between 75% and 100% (i.e., I2 = 83%); this could not be explained due to small subgroups and may represent considerable heterogeneity.

ay. Inconsistency: We downgraded once. There is similarity in some of the point estimates with overlapping confidence intervals. Statistical heterogeneity is between 75% and 100% (i.e., I2 = 88%); this could not be explained due to small subgroups and may represent considerable heterogeneity.

az. Inconsistency: We did not downgrade. There is similarity in the majority of point estimates with overlapping confidence intervals. Statistical heterogeneity is between 50% and 90% (i.e., I2 = 70%); this could not be explained due to small subgroups and may represent substantial heterogeneity.

ba. Inconsistency: We downgraded once. There is similarity in some of the point estimates with overlapping confidence intervals. Statistical heterogeneity is between 75% and 100% (i.e., I2 = 82%); this could not be explained due to small subgroups and may represent considerable heterogeneity.

bb. Indirectness: We downgraded once. Trial(s) conducted in one country (low income).

bc. Smeets 2008 was not included in the meta-analysis (provided within-group mean changes; no follow-up scores). 119 participants, rated as overall low risk of bias. Participants performed combination treatment (active physical treatment [aerobic and core strengthening exercises] + graded activity with problem solving training) vs. graded activity with problem solving training alone. Between-group MD (RMDQ 0-24, benefit indicated by lower values) graded activity with problem solving training alone vs. combination treatment = 1.11, 95% CI -0.56 to 2.79.

#### References

1.Abadi. The effect of aquatic exercise program on low-back pain disability in obese women. 2019.

2.Abdel-Aziem. The Effects of Stabilization Exercises Combined With Pelvic Floor Exercise in Women With Nonspecific Low Back Pain: A Randomized Clinical Study. 2021.

3.Afzal. Effects of virtual reality exercises and routine physical therapy on pain intensity and functional disability in patients with chronic low back pain. 2022.

4.Ahmadizadeh. The Effect of Stabilization Exercises Along With Self-care Training on Transverse Abdominal Activity, Pain, and Disability in Mothers With Low Back Pain Having Children With Cerebral Palsy: A Randomized Controlled Trial. 2020.

5.Ain. Effects of Sustained Natural Apophyseal Glides with and without thoracic posturecorrection techniques on mechanical back pain: a randomized control trial. 2019.

6.Alikhajeh. A Comparison of 6 Weeks of Aquatic Exercise and Kinesio Taping in Patients With Chronic Nonspecific Low Back Pain. 2021.

7.Alvani. Neuromuscular exercises on pain intensity, functional disability, proprioception, and balance of military personnel with chronic low back pain. 2021.

8.Barni. Does an Aerobic Exercise Improve Outcomes in Older Sedentary Nonspecific Low Back Pain Subjects? A Randomized Controlled Study. 2018.

9.Bruehl. Are endogenous opioid mechanisms involved in the effects of aerobic exercise training on chronic low back pain? A randomized controlled trial. 2020.

10.da Luz. Neuromuscular electrical stimulation associated with core stability exercises in nonspecific postural low back pain: a randomized clinical trial. 2019.

11.da Silva. Back school program for back pain: education or physical exercise? 2014.

12.Dalichau. Quantification of spinal configuration and postural capacity by ultrasound topometry for evaluation of different muscle strengthening programs in the therapy of back pain. 2005.

13.Dineshkumar. Effect of abdominal drawing-in maneuver along with resisted ankle dorsi-flexion to activate transverse abdominis muscle in chronic non-specific low back pain. 2015.

14.Fukuda. Does adding hip strengthening exercises to manual therapy and segmental stabilization improve outcomes in patients with nonspecific low back pain? A randomized controlled trial. 2021.

15.Gao. Effect observation of Baduanjin training on chronic nonspecific low back pain. 2018.

16.Ge. Effects of core stability training on older women with low back pain: a randomized controlled trial. 2022.

17.Hatefi. The effect of static stretching exercises on hip range of motion, pain, and disability in patients with non-specific low back pain. 2021.

18.Hemmati. Effects of consecutive supervised core stability training on pain and disability in women with nonspecific chronic low back pain. 2011.

19.Idowu. Efficacy of Graded Activity with and without Daily-Monitored-Walking on Pain and Back Endurance among Patients with Concomitant Low-Back Pain and Type-2 Diabetes: A Randomized Trial. 2020.

20.Joseph. Effects of Massage as a Combination Therapy with Lumbopelvic Stability Exercises as Compared to Standard Massage Therapy in Low Back Pain: a Randomized Cross-Over Study. 2018.

21.Karimzadeh. The effect of 8 weeks core stabilization exercises on pain and functional disability induced by low back pain in the mothers of children with cerebral palsy. 2016.

22.Kim. Effects of 3D Moving Platform Exercise on Physiological Parameters and Pain in Patients with Chronic Low Back Pain. 2020.

23.Lang. A randomized controlled trial investigating effects of an individualized pedometer driven walking program on chronic low back pain. 2021.

24.Li. Clinical observation of treating chronic nonspecific low back pain with Baduanjin combining sling exercise therapy. 2015.

25.Li. The Effect of Virtual Reality Training on Anticipatory Postural Adjustments in Patients with Chronic Nonspecific Low Back Pain: A Preliminary Study. 2021.

26.Liu. Chen-Style Tai Chi for Individuals (Aged 50 Years Old or Above) with Chronic Non-Specific Low Back Pain: A Randomized Controlled Trial. 2019.

27.Madadi-Shad. Effect of a corrective exercise program on gait kinetics and muscle activities in older adults with both low back pain and pronated feet: A double-blind, randomized controlled trial. 2020.

28.Miyamoto. Efficacy of the addition of modified Pilates exercises to a minimal intervention in patients with chronic low back pain: a randomized controlled trial. 2013.

29.Nardin. Effects of photobiomodulation and deep water running in patients with chronic non-specific low back pain: a randomized controlled trial. 2022.

30.Noormohammadpour. The Role of a Multi-Step Core Stability Exercise Program in the Treatment of Nurses with Chronic Low Back Pain: A Single-Blinded Randomized Controlled Trial. 2018.

31.Prado. Influence of isostretching on patients with chronic low back pain. A randomized controlled trial. 2019.

32.Rahbar. Effect of hippotherapy simulator on pain, disability and range of motion of the spinal column in subjects with mechanical low back pain: A randomized single-blind clinical trial. 2018.

33.Rotter. Mindful Walking in Patients with Chronic Low Back Pain: A Randomized Controlled Trial. 2022.

34.Shamsi. Effects of static stretching and strengthening exercises on flexion relaxation ratio in patients with LBP: A randomized clinical trial. 2022.

35.Srivastav. Comparison between Effectiveness of Lumbar Stabilization Exercises and Conventional Physical Therapy in the Management of Mechanical Low Back Pain . 2018.

36.Suh. The effect of lumbar stabilization and walking exercises on chronic low back pain: A randomized controlled trial. 2019.

37.Takinaci. Clinical efficacy of spa therapy (balneotherapy) for chronic low back pain: A randomized single-blind trial. 2019.

38.Weiner. Efficacy of percutaneous electrical nerve stimulation and therapeutic exercise for older adults with chronic low back pain: a randomized controlled trial. 2008.

39.Yalfani. Effects of an 8-Week Virtual Reality Training Program on Pain, Fall Risk, and Quality of Life in Elderly Women with Chronic Low Back Pain: Double-Blind Randomized Clinical Trial. 2022.

40.Zakari. Comparison of the Effects of Positional Release Therapy and Lumbar Stabilization Exercises in the Management of Chronic Mechanical Low Back Pain—Randomized Controlled Trial. 2019.

41.Karimi. The effects of consecutive supervised functional lumbar stabilizing exercises on the postural balance and functional disability in low back pain. 2014.

42.Smeets. Chronic low back pain: physical training, graded activity with problem solving training, or both? The one-year post-treatment results of a randomized controlled trial. 2008.

43.Shu. Observation on the Curative Effect of Massage Manipulation Combined with Core Strength Training in Patients with Chronic Nonspecific Low Back Pain. 2021.

44.Ansari. The effect of aquatic exercise on functional disability, flexibility and function of trunk muscles in postmenopausal women with chronic non-specific low back pain: Randomized controlled trial. 2020.

45.Fouda. Effects of proprioceptive neuromuscular facilitation techniques in treating chronic nonspecific low back pain patients. 2021.

46.Heidari. Comparison of the Effects of 8 Weeks of Core Stability Exercise on Ball and Sling Exercise on The Quality of Life and Pain in The Female With Non-Specific Chronic Low Back Pain (NSLBP). 2018.

47.Liu. Effects of Tai Chi on the event-related potential of patients with chronic non-specific low back pain. 2018.

48.Shariat. The impact of modified exercise and relaxation therapy on chronic lower back pain in office workers: a randomized clinical trial. 2019.

GRADE Evidence Profile table 2: ***What are the benefits and harms of exercise in the management of community-dwelling adults (including older adults aged 60 years and over) with chronic primary low back pain (with or without leg pain) compared with sham?***

| **Certainty assessment** | | | | | | | **№ of patients** | | **Effect** | | **Certainty** | **Importance** |
| --- | --- | --- | --- | --- | --- | --- | --- | --- | --- | --- | --- | --- |
| **№ of studies** | **Study design** | **Risk of bias** | **Inconsistency** | **Indirectness** | **Imprecision** | **Other considerations** | **exercise** | **sham** | **Relative (95% CI)** | **Absolute (95% CI)** |  |  |
| **Pain (follow-up: closest to 2 weeks; assessed with: VAS, NRS; benefit indicated by lower values; Scale from: 0 to 10)** | | | | | | | | | | | | |
| 4^1,2,3,4,a^ | randomised trials | very serious^b^ | serious^c^ | not serious^d^ | serious^e^ | none | 192 | 152 | - | MD **1.51 lower** (3.02 lower to 0 ) | ⨁◯◯◯ Very low | CRITICAL |
| **Pain in adults (excluding those aged 60+ years) (follow-up: closest to 2 weeks; assessed with: VAS, NRS; benefit indicated by lower values; Scale from: 0 to 10)** | | | | | | | | | | | | |
| 3^1,2,4,a^ | randomised trials | very serious^b^ | not serious^f^ | not serious^d^ | serious^e^ | none | 152 | 112 | - | MD **0.61 lower** (0.91 lower to 0.31 lower) | ⨁◯◯◯ Very low | CRITICAL |
| **Pain in older adults (aged 60+ years) (follow-up: closest to 2 weeks; assessed with: VAS; benefit indicated by lower values; Scale from: 0 to 10)** | | | | | | | | | | | | |
| 1^3^ | randomised trials | very serious^b^ | not serious^g^ | serious^h^ | very serious^i^ | none | 40 | 40 | - | MD **5.54 lower** (6.43 lower to 4.65 lower) | ⨁◯◯◯ Very low | CRITICAL |
| **Pain in adults in low or lower-middle income countries (follow-up: closest to 2 weeks)** | | | | | | | | | | | | |
| 0 |  |  |  |  |  |  |  |  |  |  |  | CRITICAL |
| **Pain (core strengthening) (follow-up: closest to 2 weeks; assessed with: VAS; benefit indicated by lower values; Scale from: 0 to 10)** | | | | | | | | | | | | |
| 1^3^ | randomised trials | very serious^b^ | not serious^g^ | serious^h^ | very serious^i^ | none | 40 | 40 | - | MD **5.54 lower** (6.43 lower to 4.65 lower) | ⨁◯◯◯ Very low | CRITICAL |
| **Pain (general/muscle strength training) (follow-up: closest to 2 weeks; assessed with: VAS; benefit indicated by lower values; Scale from: 0 to 10)** | | | | | | | | | | | | |
| 1^1,a^ | randomised trials | very serious^b^ | not serious^g^ | serious^h^ | very serious^i^ | none | 22 | 10 | - | MD **0.55 lower** (1.03 lower to 0.07 lower) | ⨁◯◯◯ Very low | CRITICAL |
| **Pain (motor control exercise) (follow-up: closest to 2 weeks; assessed with: VAS, NRS; benefit indicated by lower values; Scale from: 0 to 10)** | | | | | | | | | | | | |
| 2^2,4,a^ | randomised trials | very serious^b^ | serious^j^ | not serious^d^ | serious^e^ | none | 106 | 92 | - | MD **0.87 lower** (1.66 lower to 0.09 lower) | ⨁◯◯◯ Very low | CRITICAL |
| **Pain (stretching or flexibility/mobilizing exercise) (follow-up: closest to 2 weeks; assessed with: VAS; benefit indicated by lower values; Scale from: 0 to 10)** | | | | | | | | | | | | |
| 1^1,a^ | randomised trials | very serious^b^ | not serious^g^ | serious^h^ | very serious^i^ | none | 24 | 10 | - | MD **0.55 lower** (1.01 lower to 0.09 lower) | ⨁◯◯◯ Very low | CRITICAL |
| **Pain (low ROB) (follow-up: closest to 2 weeks; assessed with: NRS; benefit indicated by lower values; Scale from: 0 to 10)** | | | | | | | | | | | | |
| 1^2,a^ | randomised trials | not serious^k^ | not serious^g^ | serious^h^ | very serious^i^ | none | 77 | 77 | - | MD **1 lower** (1.85 lower to 0.15 lower) | ⨁◯◯◯ Very low | CRITICAL |
| **Pain (high or unclear ROB) (follow-up: closest to 2 weeks; assessed with: VAS; benefit indicated by lower values; Scale from: 0 to 10)** | | | | | | | | | | | | |
| 3^1,3,4,a^ | randomised trials | very serious^b^ | serious^l^ | not serious^d^ | serious^e^ | none | 115 | 75 | - | MD **1.6 lower** (3.44 lower to 0.24 higher) | ⨁◯◯◯ Very low | CRITICAL |
| **Pain (motor control exercise, low ROB trial) (follow-up: closest to 12 months; assessed with: NRS; benefit indicated by lower values; Scale from: 0 to 10)** | | | | | | | | | | | | |
| 1^2,a^ | randomised trials | not serious^k^ | not serious^g^ | serious^h^ | very serious^i^ | none | 77 | 77 | - | MD **1.3 lower** (2.13 lower to 0.47 lower) | ⨁◯◯◯ Very low | CRITICAL |
| **Pain in older adults (aged 60+ years) (follow-up: closest to 12 months)** | | | | | | | | | | | | |
| 0 |  |  |  |  |  |  |  |  |  |  |  | CRITICAL |
| **Pain in adults in low or lower-middle income countries (follow-up: closest to 12 months)** | | | | | | | | | | | | |
| 0 |  |  |  |  |  |  |  |  |  |  |  | CRITICAL |
| **Function (follow-up: closest to 2 weeks; assessed with: RMDQ, ODI; benefit indicated by lower values; Scale from: 0 to 24)** | | | | | | | | | | | | |
| 3^1,2,3,a^ | randomised trials | very serious^b^ | not serious^m^ | not serious^d^ | serious^e^ | none | 163 | 137 | - | MD **3.29 lower** (6.22 lower to 0.36 lower) | ⨁◯◯◯ Very low | CRITICAL |
| **Function in adults (excluding those aged 60+ years) (follow-up: closest to 2 weeks; assessed with: RMDQ; benefit indicated by lower values; Scale from: 0 to 24)** | | | | | | | | | | | | |
| 2^1,2,a^ | randomised trials | very serious^b^ | not serious^f^ | not serious^d^ | serious^e^ | none | 123 | 97 | - | MD **2.04 lower** (2.86 lower to 1.22 lower) | ⨁◯◯◯ Very low | CRITICAL |
| **Function in older adults (aged 60+ years) (follow-up: closest to 2 weeks; assessed with: ODI; benefit indicated by lower values; Scale from: 0 to 24)** | | | | | | | | | | | | |
| 1^3^ | randomised trials | very serious^b^ | not serious^g^ | serious^h^ | very serious^i^ | none | 40 | 40 | - | MD **6.69 lower** (7.38 lower to 6 lower) | ⨁◯◯◯ Very low | CRITICAL |
| **Function in adults in low or lower-middle income countries (follow-up: closest to 2 weeks)** | | | | | | | | | | | | |
| 0 |  |  |  |  |  |  |  |  |  |  |  | CRITICAL |
| **Function (core strengthening) (follow-up: closest to 2 weeks; assessed with: ODI; benefit indicated by lower values; Scale from: 0 to 24)** | | | | | | | | | | | | |
| 1^3^ | randomised trials | very serious^b^ | not serious^g^ | serious^h^ | very serious^i^ | none | 40 | 40 | - | MD **6.69 lower** (7.38 lower to 6 lower) | ⨁◯◯◯ Very low | CRITICAL |
| **Function (general/muscle strength training) (follow-up: closest to 2 weeks; assessed with: RMDQ; benefit indicated by lower values; Scale from: 0 to 24)** | | | | | | | | | | | | |
| 1^1,a^ | randomised trials | very serious^b^ | not serious^g^ | serious^h^ | very serious^i^ | none | 22 | 10 | - | MD **2.01 lower** (3.32 lower to 0.7 lower) | ⨁◯◯◯ Very low | CRITICAL |
|  | | | | | | | | | | | | |
|  | | | | | | | | | | | | |
|  | | | | | | | | | | | | |
| **Function (motor control exercise) (follow-up: closest to 2 weeks; assessed with: RMDQ; benefit indicated by lower values; Scale from: 0 to 24)** | | | | | | | | | | | | |
| 1^2,a^ | randomised trials | not serious^k^ | not serious^g^ | serious^h^ | very serious^i^ | none | 77 | 77 | - | MD **2.3 lower** (4.26 lower to 0.34 lower) | ⨁◯◯◯ Very low | CRITICAL |
| **Function (stretching, or flexibility/mobilizing exercise) (follow-up: closest to 2 weeks; assessed with: RMDQ; benefit indicated by lower values; Scale from: 0 to 24)** | | | | | | | | | | | | |
| 1^1,a^ | randomised trials | very serious^b^ | not serious^g^ | serious^h^ | very serious^i^ | none | 24 | 10 | - | MD **1.97 lower** (3.22 lower to 0.72 lower) | ⨁◯◯◯ Very low | CRITICAL |
| **Function (low ROB) (follow-up: closest to 2 weeks; assessed with: RMDQ; benefit indicated by lower values; Scale from: 0 to 24)** | | | | | | | | | | | | |
| 1^2^ | randomised trials | not serious^k^ | not serious^g^ | serious^h^ | very serious^i^ | none | 77 | 77 | - | MD **2.3 lower** (4.26 lower to 0.34 lower) | ⨁◯◯◯ Very low | CRITICAL |
| **Function (high or unclear ROB) (follow-up: closest to 2 weeks; assessed with: RMDQ; ODI; benefit indicated by lower values; Scale from: 0 to 24)** | | | | | | | | | | | | |
| 2^1,3,a^ | randomised trials | very serious^b^ | not serious^n^ | serious^h^ | very serious^i^ | none | 86 | 60 | - | MD **3.59 lower** (7.11 lower to 0.07 lower) | ⨁◯◯◯ Very low | CRITICAL |
| **Function (motor control exercise, low ROB trial) (follow-up: closest to 12 months; assessed with: RMDQ; benefit indicated by lower values; Scale from: 0 to 24)** | | | | | | | | | | | | |
| 1^2,a^ | randomised trials | not serious^k^ | not serious^g^ | serious^h^ | very serious^i^ | none | 77 | 77 | - | MD **0.9 lower** (3.15 lower to 1.35 higher) | ⨁◯◯◯ Very low | CRITICAL |
| **Function in older adults (aged 60+ years) (follow-up: closest to 12 months)** | | | | | | | | | | | | |
| 0 |  |  |  |  |  |  |  |  |  |  |  | CRITICAL |
| **Function in adults in low or lower-middle income countries (follow-up: closest to 12 months)** | | | | | | | | | | | | |
| 0 |  |  |  |  |  |  |  |  |  |  |  | CRITICAL |
| **Harms** | | | | | | | | | | | | |
| 1^2,o^ | randomised trials | not serious^k^ | not serious^g^ | serious^h^ | very serious^i^ | none | 3/77 (3.9%) | 2/77 (2.6%) | **OR 1.52** (0.25 to 9.36) | **13 more per 1,000** (from 19 fewer to 174 more) | ⨁◯◯◯ Very low | CRITICAL |

**CI:** confidence interval; **MD:** mean difference; **NRS:** Numerical Rating Scale; **ODI:** Oswestry Disability Index; **OR:** odds ration; **PSFS:** Patient-Specific Functional Scale; **RMDQ:** Roland Morris Disability Questionnaire; **VAS:** Visual Analog Scale

#### Explanations

a. Comparison groups were split in half for trials with multiple comparisons.

b. Risk of bias: We downgraded twice. Most or all trials were rated as overall high risk of bias.

c. Inconsistency: We downgraded once. There is similarity in some of the point estimates with overlapping confidence intervals. Statistical heterogeneity is between 75% and 100% (i.e., I2 = 95%); this could not be explained due to small subgroups and may represent considerable heterogeneity.

d. Indirectness: We did not downgrade. Trials conducted in different high-income countries.

e. Imprecision: We downgraded once due to low sample size (OIS would not have been reached).

f. Inconsistency: We did not downgrade. The point estimates are similar with overlapping confidence intervals; statistical heterogeneity is between 0% and 40%, which might not be important (i.e., I2 = 0%).

g. Inconsistency: We did not downgrade; however, there are no additional trials with which to compare findings.

h. Indirectness: We downgraded once. Trial(s) conducted in one country (high income).

i. Imprecision: We downgraded twice due to low sample size (OIS would not have been reached).

j. Inconsistency: We downgraded once. The point estimates are similar with overlapping confidence intervals; statistical heterogeneity is between 0% and 40%, which might not be important (i.e., I2 = 6%).

k. Risk of bias: We did not downgrade. Trial(s) rated as overall low risk of bias.

l. Inconsistency: We downgraded once. There is similarity in some of the point estimates with overlapping confidence intervals. Statistical heterogeneity is between 75% and 100% (i.e., I2 = 96%); this could not be explained due to small subgroups and may represent considerable heterogeneity.

m. Inconsistency: We did not downgrade. There is similarity in some of the point estimates with overlapping confidence intervals. Statistical heterogeneity is between 75% and 100% (i.e., I2 = 96%); this could not be explained due to small subgroups and may represent considerable heterogeneity.

n. Inconsistency: We did not downgrade. There is similarity in some of the point estimates with overlapping confidence intervals. Statistical heterogeneity is between 75% and 100% (i.e., I2 = 99%); this could not be explained due to small subgroups and may represent considerable heterogeneity.

o. Costa 2009: motor control exercise. Does not include older adults (60+ years). All adverse events were temporary exacerbations of pain.

#### References

1.Kim. Core Stability and Hip Exercises Improve Physical Function and Activity in Patients with Non-Specific Low Back Pain: A Randomized Controlled Trial. 2020.

2.Costa. Motor control exercise for chronic low back pain: a randomized placebo-controlled trial. 2009.

3.Park. A Randomized Controlled Trial Investigating the Effects of Equine Simulator Riding on Low Back Pain, Morphological Changes, and Trunk Musculature in Elderly Women. 2020.

4.Xu. Effect of Transversus abdominis muscle training on pressure-pain threshold in patients with chronic low Back pain. 2021.

GRADE Evidence Profile table 3: ***What are the benefits and harms of exercise in the management of community-dwelling adults (including older adults aged 60 years and over) with chronic primary low back pain (with or without leg pain) compared with usual care?***

| **Certainty assessment** | | | | | | | **№ of patients** | | **Effect** | | **Certainty** | **Importance** |
| --- | --- | --- | --- | --- | --- | --- | --- | --- | --- | --- | --- | --- |
| **№ of studies** | **Study design** | **Risk of bias** | **Inconsistency** | **Indirectness** | **Imprecision** | **Other considerations** | **exercise** | **usual care** | **Relative (95% CI)** | **Absolute (95% CI)** |  |  |
| **Pain (follow-up: closest to 2 weeks; assessed with: VAS, NRS; benefit indicated by lower values; Scale from: 0 to 10)** | | | | | | | | | | | | |
| 5^1,2,3,4,5,a,b,c^ | randomised trials | very serious^d^ | not serious^e^ | not serious^f^ | serious^g^ | none | 288 | 166 | - | MD **0.89 lower** (1.27 lower to 0.5 lower) | ⨁◯◯◯ Very low | CRITICAL |
| **Pain in adults (excluding those aged 60+ years) (follow-up: closest to 2 weeks; assessed with: VAS, NRS; benefit indicated by lower values; Scale from: 0 to 10)** | | | | | | | | | | | | |
| 3^1,3,4,a,b,c^ | randomised trials | very serious^d^ | not serious^h^ | not serious^f^ | serious^g^ | none | 232 | 115 | - | MD **0.93 lower** (1.4 lower to 0.45 lower) | ⨁◯◯◯ Very low | CRITICAL |
| **Pain in older adults (aged 60+ years) (follow-up: closest to 2 weeks; assessed with: NRS; benefit indicated by lower values; Scale from: 0 to 10)** | | | | | | | | | | | | |
| 2^2,5^ | randomised trials | very serious^d^ | not serious^i^ | not serious^j^ | very serious^k^ | none | 56 | 51 | - | MD **0.65 lower** (1.5 lower to 0.19 higher) | ⨁◯◯◯ Very low | CRITICAL |
| **Pain (high or upper-middle income countries) (follow-up: closest to 2 weeks; assessed with: VAS, NRS; benefit indicated by lower values; Scale from: 0 to 10)** | | | | | | | | | | | | |
| 4^2,3,4,5,a,b,c^ | randomised trials | very serious^d^ | not serious^l^ | not serious^j^ | serious^g^ | none | 243 | 118 | - | MD **1.01 lower** (1.32 lower to 0.7 lower) | ⨁◯◯◯ Very low | CRITICAL |
| **Pain (low or lower-middle income countries) (follow-up: closest to 2 weeks; assessed with: NRS; benefit indicated by lower values; Scale from: 0 to 10)** | | | | | | | | | | | | |
| 1^1^ | randomised trials | serious^m^ | not serious^n^ | serious^o^ | very serious^k^ | none | 45 | 48 | - | MD **0.1 higher** (0.81 lower to 1.01 higher) | ⨁◯◯◯ Very low | CRITICAL |
| **Pain (core strengthening) (follow-up: closest to 2 weeks; assessed with: NRS; benefit indicated by lower values; Scale from: 0 to 10)** | | | | | | | | | | | | |
| 1^4,c^ | randomised trials | very serious^d^ | not serious^n^ | serious^p^ | very serious^k^ | none | 7 | 7 | - | MD **2.3 lower** (3.96 lower to 0.64 lower) | ⨁◯◯◯ Very low | CRITICAL |
| **Pain (general/muscle strength training) (follow-up: closest to 2 weeks; assessed with: VAS; benefit indicated by lower values; Scale from: 0 to 10)** | | | | | | | | | | | | |
| 1^3,a^ | randomised trials | very serious^d^ | not serious^n^ | serious^p^ | serious^g^ | none | 180 | 60 | - | MD **1.01 lower** (1.36 lower to 0.65 lower) | ⨁◯◯◯ Very low | CRITICAL |
| **Pain (mixed exercise) (follow-up: closest to 2 weeks; assessed with: NRS; benefit indicated by lower values; Scale from: 0 to 10)** | | | | | | | | | | | | |
| 3^1,2,5^ | randomised trials | very serious^d^ | not serious^i^ | not serious^f^ | serious^g^ | none | 101 | 99 | - | MD **0.31 lower** (0.93 lower to 0.31 higher) | ⨁◯◯◯ Very low | CRITICAL |
| **Pain (yoga) (follow-up: closest to 2 weeks; assessed with: Aberdeen Back Pain Scale, 0-100; benefit indicated by lower values)** | | | | | | | | | | | | |
| 1^6,q^ | randomised trials | serious^m^ | not serious^n^ | serious^p^ | serious^g^ | none | Yoga vs. usual care: difference in mean change -2.42, 95% CI -4.97 to 0.12 (313 participants total). | | | | ⨁◯◯◯ Very low | CRITICAL |
| **Pain (low ROB trials) (follow-up: closest to 2 weeks)** | | | | | | | | | | | | |
| 0 |  |  |  |  |  |  |  |  | - | **0**  (0 to 0 ) | - | CRITICAL |
|  | | | | | | | | | | | | |
| **Pain (older adults aged 60+ years, mixed exercise, unclear ROB trial) (follow-up: closest to 3 months; assessed with: NRS; benefit indicated by lower values; Scale from: 0 to 10)** | | | | | | | | | | | | |
| 1^2^ | randomised trials | serious^m^ | not serious^n^ | serious^p^ | very serious^k^ | none | 26 | 22 | - | MD **0.3 lower** (1.66 lower to 1.06 higher) | ⨁◯◯◯ Very low | CRITICAL |
| **Pain (low or lower-middle income countries) (follow-up: closest to 3 months)** | | | | | | | | | | | | |
| 0 |  |  |  |  |  |  |  |  |  |  |  | CRITICAL |
| **Pain (yoga exercise) (follow-up: closest to 12 months; assessed with: Aberdeen Back Pain Scale, 0-100; benefit indicated by lower values)** | | | | | | | | | | | | |
| 1^6,q^ | randomised trials | serious^m^ | not serious^n^ | serious^p^ | serious^g^ | none | Yoga vs. usual care: difference in mean change -0.73, 95% CI -3.30 to 1.84 (313 participants total). | | | | ⨁◯◯◯ Very low | CRITICAL |
| **Pain in older adults (aged 60+ years) (follow-up: closest to 12 months)** | | | | | | | | | | | | |
| 0 |  |  |  |  |  |  |  |  |  |  |  | CRITICAL |
| **Pain (low or lower-middle income countries) (follow-up: closest to 12 months)** | | | | | | | | | | | | |
| 0 |  |  |  |  |  |  |  |  |  |  |  | CRITICAL |
| **Pain (low ROB trials) (follow-up: closest to 12 months)** | | | | | | | | | | | | |
| 0 |  |  |  |  |  |  |  |  |  |  |  | CRITICAL |
| **Function (follow-up: closest to 2 weeks; assessed with: RMDQ, ODI, modified ODI; benefit indicated by lower values; Scale from: 0 to 100)** | | | | | | | | | | | | |
| 6^1,2,3,4,5,7,a,r^ | randomised trials | very serious^d^ | not serious^s^ | not serious^f^ | not serious^t^ | none | 303 | 181 | - | MD **9.72 lower** (13.72 lower to 5.72 lower) | ⨁⨁◯◯ Low | CRITICAL |
| **Function in adults (excluding those aged 60+ years) (follow-up: closest to 2 weeks; assessed with: RMDQ, ODI, modified ODI; benefit indicated by lower values; Scale from: 0 to 100)** | | | | | | | | | | | | |
| 4^1,3,4,7,a,r^ | randomised trials | very serious^d^ | not serious^u^ | not serious^f^ | serious^g^ | none | 247 | 130 | - | MD **9.72 lower** (14.37 lower to 5.07 lower) | ⨁◯◯◯ Very low | CRITICAL |
|  | | | | | | | | | | | | |
| **Function in older adults (aged 60+ years) (follow-up: closest to 2 weeks; assessed with: RMDQ; benefit indicated by lower values; Scale from: 0 to 100)** | | | | | | | | | | | | |
| 2^2,5^ | randomised trials | very serious^d^ | not serious^i^ | not serious^j^ | very serious^k^ | none | 56 | 51 | - | MD **9.81 lower** (16.11 lower to 3.52 lower) | ⨁◯◯◯ Very low | CRITICAL |
| **Function (high or upper-middle income countries) (follow-up: closest to 2 weeks; assessed with: RMDQ, ODI; benefit indicated by lower values; Scale from: 0 to 100)** | | | | | | | | | | | | |
| 4^2,3,4,5,a,r^ | randomised trials | very serious^d^ | not serious^v^ | not serious^j^ | serious^g^ | none | 243 | 118 | - | MD **8.13 lower** (10.69 lower to 5.58 lower) | ⨁◯◯◯ Very low | CRITICAL |
| **Function (low or lower-middle income countries) (follow-up: closest to 2 weeks; assessed with: ODI, modified ODI; benefit indicated by lower values; Scale from: 0 to 100)** | | | | | | | | | | | | |
| 2^1,7^ | randomised trials | very serious^d^ | not serious^w^ | serious^o^ | very serious^k^ | none | 60 | 63 | - | MD **14.02 lower** (19.75 lower to 8.3 lower) | ⨁◯◯◯ Very low | CRITICAL |
| **Function (aerobic exercise) (follow-up: closest to 2 weeks; assessed with: ODI; benefit indicated by lower values; Scale from: 0 to 100)** | | | | | | | | | | | | |
| 1^7^ | randomised trials | very serious^d^ | not serious^n^ | serious^o^ | very serious^k^ | none | 15 | 15 | - | MD **16 lower** (17.59 lower to 14.41 lower) | ⨁◯◯◯ Very low | CRITICAL |
| **Function (core strengthening) (follow-up: closest to 2 weeks; assessed with: ODI; benefit indicated by lower values; Scale from: 0 to 100)** | | | | | | | | | | | | |
| 1^4^ | randomised trials | very serious^d^ | not serious^n^ | serious^p^ | very serious^k^ | none | 7 | 7 | - | MD **4.3 lower** (9.64 lower to 1.04 higher) | ⨁◯◯◯ Very low | CRITICAL |
|  | | | | | | | | | | | | |
|  | | | | | | | | | | | | |
| **Function (general/muscle strength training) (follow-up: closest to 2 weeks; assessed with: ODI; benefit indicated by lower values; Scale from: 0 to 100)** | | | | | | | | | | | | |
| 1^3,a^ | randomised trials | very serious^d^ | not serious^n^ | serious^p^ | serious^g^ | none | 180 | 60 | - | MD **8.95 lower** (11.96 lower to 5.93 lower) | ⨁◯◯◯ Very low | CRITICAL |
| **Function (mixed exercise) (follow-up: closest to 2 weeks; assessed with: RMDQ, modified ODI; benefit indicated by lower values; Scale from: 0 to 100)** | | | | | | | | | | | | |
| 3^1,2,5^ | randomised trials | very serious^d^ | not serious^i^ | not serious^f^ | serious^g^ | none | 101 | 99 | - | MD **9.77 lower** (14.64 lower to 4.89 lower) | ⨁◯◯◯ Very low | CRITICAL |
| **Function (yoga) (follow-up: closest to 2 weeks; assessed with: RMDQ, 0-24; benefit indicated by lower values)** | | | | | | | | | | | | |
| 1^6^ | randomised trials | serious^m^ | not serious^n^ | serious^p^ | serious^g^ | none | Yoga vs. usual care: difference in mean change -2.17, 95% CI -3.31 to -1.03 (313 participants total). | | | | ⨁◯◯◯ Very low | CRITICAL |
| **Function (low ROB trials) (follow-up: closest to 2 weeks)** | | | | | | | | | | | | |
| 0 |  |  |  |  |  |  |  |  | - | **0**  (0 to 0 ) | - | CRITICAL |
| **Function (older adults aged 60+ years, mixed exercise, unclear ROB trial) (follow-up: closest to 3 months; assessed with: RMDQ; benefit indicated by lower values; Scale from: 0 to 24)** | | | | | | | | | | | | |
| 1^2^ | randomised trials | serious^m^ | not serious^n^ | serious^p^ | very serious^k^ | none | 26 | 22 | - | MD **2.3 lower** (4.92 lower to 0.32 higher) | ⨁◯◯◯ Very low | CRITICAL |
| **Function (low or lower-middle income countries) (follow-up: closest to 3 months)** | | | | | | | | | | | | |
| 0 |  |  |  |  |  |  |  |  |  |  |  | CRITICAL |
| **Function (yoga) (follow-up: closest to 12 months; assessed with: RMDQ 0 to 24; benefit indicated by lower values)** | | | | | | | | | | | | |
| 1^6^ | randomised trials | serious^m^ | not serious^n^ | serious^p^ | serious^g^ | none | Yoga vs. usual care: difference in mean change -1.57, 95% CI -2.71 to -0.42 (313 participants total). | | | | ⨁◯◯◯ Very low | CRITICAL |
|  | | | | | | | | | | | | |
| **Function in older adults (aged 60+ years) (follow-up: closest to 12 months)** | | | | | | | | | | | | |
| 0 |  |  |  |  |  |  |  |  |  |  |  | CRITICAL |
| **Function (low or lower-middle income countries) (follow-up: closest to 12 months)** | | | | | | | | | | | | |
| 0 |  |  |  |  |  |  |  |  |  |  |  | CRITICAL |
| **Function (low ROB trials) (follow-up: closest to 12 months)** | | | | | | | | | | | | |
| 0 |  |  |  |  |  |  |  |  |  |  |  | CRITICAL |
| **Harms** | | | | | | | | | | | | |
| 2^5,6^ | randomised trials | serious^m^ | not serious | not serious^j^ | serious^g^ | none | Tilbrook 2011: yoga vs. usual care; 313 participants total:  Minor adverse events: 11 of 156 (7.1%) yoga participants events were classified as nonserious and mostly related to increased pain. Major adverse events. 1 yoga participant experienced severe pain (possibly associated with yoga). In usual care group, 1 participant died; 1 had severe accident/injury.  Zadro 2019: mixed exercise vs. usual care; 60 older participants total: no adverse events reported. | | | | ⨁⨁◯◯ Low | CRITICAL |

**CI:** confidence interval; **MD:** mean difference; **NRS:** Numerical Rating Scale; **ODI:** Oswestry Disability Index; **RMDQ:** Roland Morris Disability Questionnaire; **VAS:** Visual Analog Scale

#### Explanations

a. Comparison groups were split for trials with multiple comparisons.

b. Tilbrook 2011: not included in meta-analysis (only reported within-group changes; follow-up scores not provided). Rated as unclear overall risk of bias. Yoga vs. usual care: difference in mean change -2.42, 95% CI -4.97 to 0.12 (313 participants total; Aberdeen Back Pain Scale 0-100, benefit indicated by lower values).

c. Raoul 2019: not included in meta-analysis due to missing data. Rated as high overall risk of bias. Core strengthening vs. usual care: greater mean pain reduction in exercise group (3.91, SD 2.88) than in comparison group (1.83, SD 2.80), p<0.01(67 participants total; NRS 0-10, benefit indicated by lower values).

d. Risk of bias: We downgraded twice. Most or all trials were rated as overall high risk of bias.

e. Inconsistency: We did not downgrade. There is similarity in the point estimates with overlapping confidence intervals. Statistical heterogeneity is between 30% and 60% (i.e., I2 = 50%); this could not be explained due to small subgroups and may represent moderate heterogeneity.

f. Indirectness: We did not downgrade. Trials conducted in different countries both high and low income.

g. Imprecision: We downgraded once due to low sample size (OIS would not have been reached).

h. Inconsistency: We did not downgrade. There is similarity in the majority of point estimates with overlapping confidence intervals. Statistical heterogeneity is between 50% and 90% (i.e., I2 = 65%); this could not be explained due to small subgroups and may represent substantial heterogeneity.

i. Inconsistency: We did not downgrade. The point estimates are similar with overlapping confidence intervals; statistical heterogeneity is between 0% and 40%, which might not be important (i.e., I2 = 0%).

j. Indirectness: We did not downgrade. Trials conducted in different high-income countries.

k. Imprecision: We downgraded twice due to low sample size (OIS would not have been reached).

l. Inconsistency: We did not downgrade. The point estimates are similar with overlapping confidence intervals; statistical heterogeneity is between 0% and 40%, which might not be important (i.e., I2 = 26%).

m. Risk of bias: We downgraded once. Some (>50%) or all weight comes from trials with unclear risk of bias.

n. Inconsistency: We did not downgrade; however, there are no additional trials with which to compare findings.

o. Indirectness: We downgraded once. Trial(s) conducted in one country (low income).

p. Indirectness: We downgraded once. Trial(s) conducted in one country (high income).

q. Tillbrook 2011: not included in meta-analysis (only reported within-group changes; follow-up scores not provided).

r. Tillbrook 2011: not included in meta-analysis (only reported within-group changes; follow-up scores not provided). Rated as unclear overall risk of bias. Yoga vs. usual care: difference in mean change -2.17, 95% CI -3.31 to -1.03 (313 participants total; RMDQ 0-24, benefit indicated by lower values).

s. Inconsistency: We did not downgrade. There is similarity in some of the point estimates with overlapping confidence intervals. Statistical heterogeneity is between 75% and 100% (i.e., I2 = 80%); this could not be explained due to small subgroups and may represent considerable heterogeneity.

t. Imprecision: We did not downgrade. OIS would have been reached. The point estimate did not reach the pre-specified threshold for what may be considered appreciable benefit (MD = -10 or SMD = -0.2); the confidence interval does not cross the null.

u. Inconsistency: We did not downgrade. There is similarity in some of the point estimates with overlapping confidence intervals. Statistical heterogeneity is between 75% and 100% (i.e., I2 = 85%); this could not be explained due to small subgroups and may represent considerable heterogeneity.

v. Inconsistency: We did not downgrade. The point estimates are similar with overlapping confidence intervals; statistical heterogeneity is between 0% and 40%, which might not be important (i.e., I2 = 9%).

w. Inconsistency: We did not downgrade. There is similarity in the majority of point estimates with overlapping confidence intervals. Statistical heterogeneity is between 50% and 90% (i.e., I2 = 59%); this could not be explained due to small subgroups and may represent substantial heterogeneity.

#### References

1.Chhabra. Smartphone app in self-management of chronic low back pain: a randomized controlled trial. 2018.

2.Jinnouchi. Effects of brief self-exercise education on the management of chronic low back pain: A community-based, randomized, parallel-group pragmatic trial. 2020.

3.Kell. The response of persons with chronic nonspecific low back pain to three different volumes of periodized musculoskeletal rehabilitation. 2011.

4.Mendes. Core stabilisation exercises reduce chronic low back pain in Air Force fighter pilots: a randomised controlled trial. 2022.

5.Zadro. Video-Game-Based Exercises for Older People With Chronic Low Back Pain: A Randomized Controlledtable Trial (GAMEBACK). 2019.

6.Tillbrook. Yoga for chronic low back pain: a randomized trial. 2011.

7.Gupta. The Effectiveness of Aerobic Exercise Program for Improving Functional Performance and Quality of Life in Chronic Low Back Pain. 2019.

**Meta-analyses**
